# Disentangling cation–polyanion coupling reveals which anion motion dominates cation transport in solid electrolytes

**DOI:** 10.1038/s41467-026-77273-x

**Published:** 2026-09-02

**Authors:** Ke Li, Jitai Yang, Yu Zhai, Hui Li

**Affiliations:** 1 State Key Laboratory of Supramolecular Structure and Materials, Institute of Theoretical Chemistry, College of Chemistry, Jilin University, Changchun, China; 2 Beijing National Laboratory for Molecular Sciences, Institute of Theoretical and Computational Chemistry, College of Chemistry and Molecular Engineering, Peking University, Beijing, China

**Keywords:** Batteries, Atomistic models, Batteries, Molecular dynamics

## Abstract

Polyanion dynamics, notably rotation, have long been linked to fast cation transport in solid electrolytes, yet their contribution to ionic conductivity remains unquantified, and even rotation’s role, disputed. Here, we quantify the contributions of polyanion translation, rotation, and vibration to conductivity in plastic crystals by combining a constraint algorithm and machine learning molecular dynamics. Contrary to prior belief that rotation primarily facilitates transport, we reveal that each motion mode can dominate: rotation when its frequency matches the cation hopping frequency, translation at higher rotation frequencies and vibration at lower rotation frequencies. We find that rotation and translation each can enhance conductivity by over two orders of magnitude by inducing cation disorder; rotation can further enhance cooperative cation transport; and polyanion rotation, translation, and vibration all modulate the migration barrier and prefactor. These findings demonstrate that polyanion dynamics are crucial to superionicity and offer design principles for superionic conductors.

## Abstract (web summary)

Fast ion transport in solid electrolytes is often attributed to anion rotation. Simulations of six materials show that rotation, translation or vibration can dominate transport, depending on how rapidly anions reorient relative to cation hopping.

## Introduction

All-solid-state batteries, which offer high energy density and improved safety, have received considerable attention and extensive research in recent years^1,2^. One of their key components, the solid electrolytes (SEs), is crucial to the device’s overall performance, and much effort has been devoted to searching for SEs with high ionic conductivity. The rational design of advanced conductive materials largely hinges on a fundamental understanding of their nature at the atomic level^3^.

From a static perspective, thermally activated cation hops occur by overcoming the energy barrier defined by the rigid host lattice; specific anion packing^4^, polyanion mixing^5,6^, and site disorder^7^ can facilitate cation diffusion. In parallel, dynamic mechanisms of ion conduction have been actively investigated. From a phonon–ion coupling perspective, specific phonon modes have been shown to contribute directly to ion hopping, and their effects on ionic conductivity have been quantified by both theory and experiment^8^. From a local perspective, correlations between polyanion dynamics and cation hops have been identified in close spatiotemporal proximity^9–12^. However, in contrast to the phonon–ion coupling, their contribution to conductivity remains unquantified. Among polyanion dynamics, rotation has received the most attention. It is commonly observed in various inorganic superionic conductors, including sulfates^13^, phosphates^14,15^, hydrides^16^, closo-borates^17–21^, antiperovskites^22,23^, and thiophosphates^9,24–26^. Its role is most often described through the paddle-wheel effect^13,27^, a hypothesis proposed over thirty years ago and since refined, that polyanion rotation facilitates cation migration^28^. Over the years, some studies have supported its role in enhancing cation conduction^9,10,12,23,26,29^, while a comparable body of work has challenged its existence^11,30–32^.

Despite the ongoing debate, several inorganic plastic crystals have been reported in which fast superionic conduction is accompanied by polyanion rotation. In 2007, Matsuo et al.^16^ discovered that the ionic conductivity of lithium borohydride surged by three orders of magnitude to 10^−3^ S cm^−1^ near 390 K. This increase was accompanied by a structural transition from the orthorhombic to hexagonal phase and the onset of quasi-free rotation of ${{{{\rm{BH}}}}}_{4}^{-}$. Incorporating lithium halide or nanoconfining can stabilize the high-temperature phase and maintain the anion rotation near room temperature, resulting in fast Li^+^ conduction^33,34^. Despite extensive exploitation of the superionic phase, the mechanism behind the conductivity surge in host material, LiBH_4_, and the specific role of ${{{{\rm{BH}}}}}_{4}^{-}$ rotation in this surge remain unclear. In the dehydrogenation process of LiBH_4_, an intermediate, Li_2_B_12_H_12_, is formed and exhibits high conductivity in its superionic phase. The related electrolyte family spans an extremely wide chemical space, including closo-type compounds^17–21^ (e.g. M_2_B_*n*_H_*n*_ (*n* = 10,12), MCB_*n*_H_*n*_ (*n* = 9,11), M = Li, Na; Na_2_B_12_H_12−*x*_I_*x*_), nido-type compounds^35^ (e.g. NaB_11_H_14_, Na-7-CB_10_H_13_, Li-7-CB_10_H_13_, Na-7,8-C_2_B_9_H_12_, and Na-7,9-C_2_B_9_H_12_). These materials exhibit remarkable conductivity, reaching up to 70 mS cm^−1^ through anion mixing at ambient temperature^36^. Studies show that in this material class, anion orientational disorder is linked to fast cation transport^12,37–39^. Another newly developed sodium SE, Na_3_OBH_4_, shows conductivity comparable to that of its liquid counterpart. This antiperovskite-type ionic conductor features a dual-anion structure and a highly rotationally mobile ${{{{\rm{BH}}}}}_{4}^{-}$. The rotation of ${{{{\rm{BH}}}}}_{4}^{-}$ is proposed to aid Na^+^-ion transport^22,23,40^.

Lithium thiophosphate represents another promising SE family and is derived from the Li_2_S–P_2_S_5_ system, with distinct units such as ${{{{\rm{PS}}}}}_{4}^{3-}$ and ${{{{\rm{P}}}}}_{2}{{{{\rm{S}}}}}_{6}^{4-}$, depending on composition^41^. One archetype, Li_3_PS_4_, transitions from the *γ* to *β* phase at intermediate temperature, along with an anomalous increase in conductivity and the onset of anion rotation^26,42^. The *β* further transitions to the *α* phase at higher temperature, which was also proposed to show exceptional conductivity^43^. Efforts to stabilize these two superionic phases at room temperature have included partial substitution^26^ and rapid heating of glass^44^. However, great controversy has arisen on the existence of the paddle-wheel effect in this type of compound^10,11,26,30–32^. Indeed, the ${{{{\rm{PS}}}}}_{4}^{3-}$ anion appears in many systems known for record-high conductivity ^25,45–48^, but the coupling between its dynamics and cation motion remains poorly understood. A novel sodium SE, Na_1_1Sn_2_PS_1_2, displays a three-dimensional chessboard framework of PS_4_ and SnS_4_ tetrahedra, achieving high room-temperature conductivity^47^. Unlike others that transition to a rotor phase at elevated temperatures, this material maintains its phase integrity and anion rotation, which was proposed to aid cation diffusion, down to very low temperatures^9^. Another recently found thiophosphate SE, Na_4_P_2_S_6_, exhibits high conductivity in *γ* phase^24^, where the orientational disorder of the prolate ${{{{\rm{P}}}}}_{2}{{{{\rm{S}}}}}_{6}^{4-}$, with low point-group symmetry compared to other rotors, and the flexibility of its dihedral angle have been reported.

Across these systems, the onset of polyanion rotation typically accompanies a phase transition, making it experimentally challenging to isolate the effects of rotation from structural changes. Moreover, this longstanding focus on rotation has overshadowed the potential contributions of polyanion translation and vibration, whose contributions to ionic conductivity have not been quantitatively addressed. Similarly, while both the migration barrier and the Arrhenius prefactor govern ionic conductivity, SE design has predominantly targeted the former, with the latter receiving comparatively less attention.

The Nernst–Einstein (NE) equation, which is an approximation to the Green–Kubo formula and converges faster, is often employed to estimate conductivity. However, given the high concentration of charge carriers in superionic conductors, ion–ion correlations can play an important role^49–51^ and lead to the failure of this approximation^52^. These correlations may themselves be coupled to anion dynamics, an aspect that has rarely been examined. Capturing them through the Green–Kubo formula, however, requires substantially longer simulations. Significantly longer timescales, even in the millisecond range, can be accessed by employing kinetic Monte Carlo coupled with local cluster expansion; this approach can establish a robust link between experimental and theoretical diffusion results^5,53^. Similarly, machine-learning interatomic potentials extend simulations to larger system sizes and longer timescales while retaining ab initio accuracy^54–56^.

In this study, we combine our recently developed constraint algorithm^57^ and machine learning molecular dynamics (MLMD) simulations to quantify the contributions of polyanion rotation, translation, and vibration to ionic conductivity across six plastic-crystal SEs from three material classes. For each material, we performed MLMD simulations for more than 100 ns at each temperature, in which specific polyanion motion modes were selectively constrained, and evaluated the ionic conductivity using the Einstein–Helfand (EH) relation. We quantify the contribution of the paddle-wheel effect to ionic conductivity and find it always operative. Yet we show that no single polyanion motion mode universally dominates cation transport. The paddle-wheel effect dominates conductivity only when the polyanion reorientation frequency, quantified using the Ivanov model, matches the cation hopping frequency; when reorientation is much faster or much slower than cation hopping, the paddle-wheel effect persists, but conductivity is instead dominated by polyanion translation or vibration, respectively. Beyond identifying the dominant motion modes, we find that anion dynamics modulate ionic conductivity through both the migration barrier, which has been the central focus in SE design, and the prefactor, which can also contribute comparably. Haven ratio analysis further shows cooperative cation transport across all systems. In the borohydrides, this cooperative motion is reinforced by anion rotation. In LiBH_4_ and Na_2_B_1__0_H_1__0_, anion dynamics are decisive for superionicity; ${{{{\rm{BH}}}}}_{4}^{-}$ translation and ${{{{\rm{B}}}}}_{10}{{{{\rm{H}}}}}_{10}^{2-}$ rotation each contribute over two orders of magnitude to the conductivity by inducing disorder within the Li and Na sublattices. These findings provide a design principle for superionic conductors exploiting polyanion dynamics.

## Results

### Quantifying each polyanion motion mode’s contribution to conductivity

**Fig. 1:**
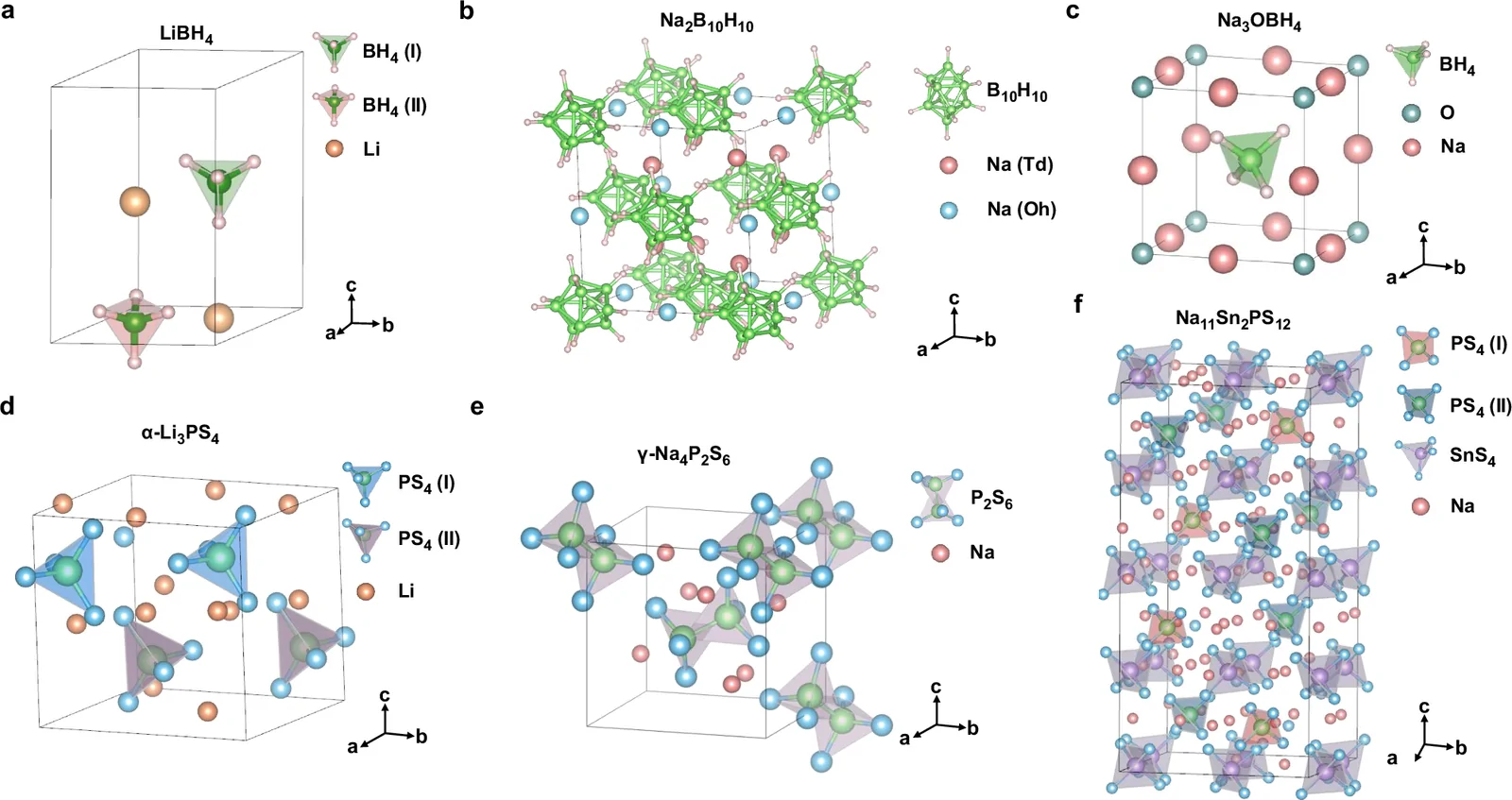
Crystal structures of the studied solid electrolytes. **a** High-temperature hexagonal polymorph of LiBH_4_ in *P*6_3_*mc* space group^93^. **b** Unit cell of disordered phase of Na_2_B_1__0_H_1__0_ with $Fm\bar{3}m$ symmetry^19^. Two types of Na sites based on coordination are shown: 4-fold tetrahedral (Td) and 6-fold octahedral (Oh). **c** Antiperovskite Na_3_OBH_4_ with cubic structure with $Pm\bar{3}m$ symmetry^22^. **d** The *α*-polymorph of Li_3_PS_4_ crystalized in *Cmcm* space group^94^. **e** *γ*-modification of Na_4_P_2_S_6_ with $Im\bar{3}m$ symmetry^24^. **f** Crystal structure of Na_1__1_Sn_2_PS_1__2_ with tetragonal *I*4_1_/acd symmetry^47^. Types I and II in (**a**, **d**, **f**) denote two distinct anion orientations.

**Fig. 2:**
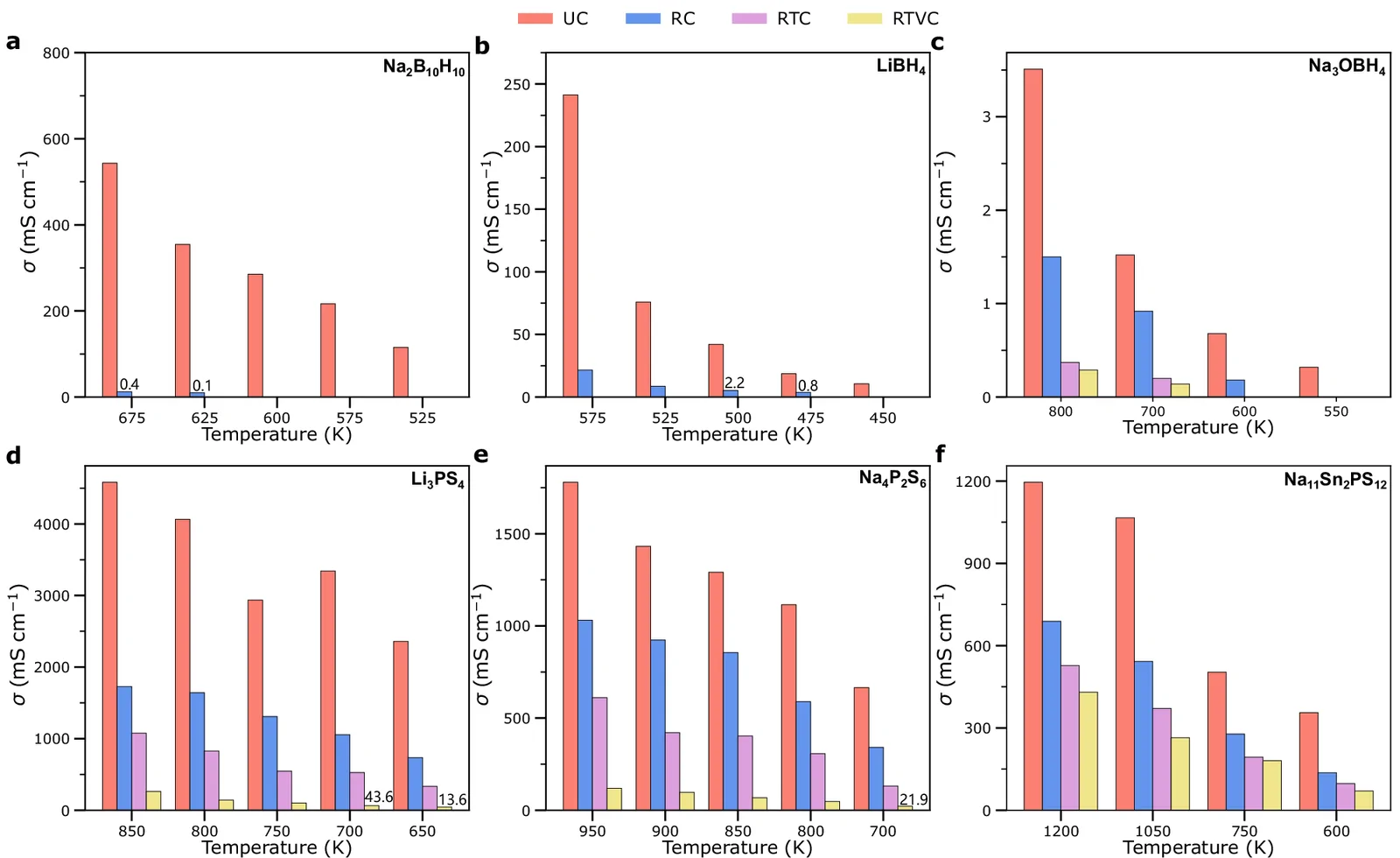
Conductivity of six materials, calculated using the Einstein–Helfand formula. **a** Na_2_B_1__0_H_1__0_, **b** LiBH_4_, **c** Na_3_OBH_4_, **d** *α*-Li_3_PS_4_, **e** *γ*-Na_4_P_2_S_6_, and **f** Na_1__1_Sn_2_PS_1__2_ under unconstrained (UC, red), rotationally constrained (RC, blue), roto-translationally constrained (RTC, purple), and roto-translationally-vibrationally constrained (RTVC, yellow) conditions at various temperatures. Bars are absent where no sufficient cation diffusion was observed within the simulation time. In Na_1__1_Sn_2_PS_1__2_, constraint is applied to PS_4_ only, as a previous study suggests that SnS_4_ exhibits no orientational disorder^9^. Source data are provided as a Source Data file.

Figure 1 shows the crystal structures of the six plastic crystals, which feature polyanions with dynamic orientational disorder, translationally fixed on their lattice sites^24^. To quantify the contribution of polyanion translation, rotation, and vibration to cation transport in SEs, we calculate the ionic conductivity using the Einstein–Helfand formula (equation (1), hereafter the EH conductivity), which is equivalent to the Green–Kubo formula and captures ion–ion cross-correlations. We performed MLMD simulations at various temperatures, with the orientationally disordered polyanions either unconstrained (UC), rotationally constrained (RC), roto-translationally constrained (RTC), or roto-translationally-vibrationally constrained (RTVC). Our constraint algorithm enables selective constraint of each anion motion mode. The detailed validation of the machine learning potential and the constraint method is presented in Sections 1 and 4 of the Supplementary Information. At each temperature, more than 100 ns of MD trajectories were used to compute the EH conductivity, and the results are shown in Fig. 2a–f.

Across the six SEs, anion dynamics enhance the conductivity. The relative contributions of anion translation, rotation, and vibration vary in each SE, and one of the three dominates across all simulated temperatures. For Na_2_B_1__0_H_1__0_, ${{{{\rm{B}}}}}_{10}{{{{\rm{H}}}}}_{10}^{2-}$ rotation governs Na^+^ transport (Fig. 2a). Under the rotational constraint, the conductivity drops by 3.1 orders of magnitude at 675 K; further constraining translation reduces the conductivity by at least 0.6 orders of magnitude. In the high-temperature polymorph of LiBH_4_, ${{{{\rm{BH}}}}}_{4}^{-}$ undergo rapid reorientation, which has been linked to fast Li^+^ diffusion^58,59^. The rotation does enhance the conductivity by an order of magnitude (Fig. 2b). Notably, no Li^+^ diffusion is observed under RTC at any simulated temperature. This reveals that translation, rather than rotation, governs Li^+^ transport. ${{{{\rm{BH}}}}}_{4}^{-}$ translation contributes more than two orders of magnitude to the conductivity at 575 K. In the thiophosphate *α*-Li_3_PS_4_, ${{{{\rm{PS}}}}}_{4}^{3-}$ vibration dominates Li^+^transport, contributing ~1.4 orders of magnitude at 650 K. Its effect on the conductivity is roughly twice that of either rotation or translation across all simulated temperatures. In the remaining three SEs, the most strongly affecting mode contributes less than one order of magnitude to the conductivity. For Na_3_OBH_4_, ${{{{\rm{BH}}}}}_{4}^{-}$ translation dominates Na^+^ transport (~0.6 orders of magnitude). In *γ*-Na_4_P_2_S_6_, ${{{{\rm{P}}}}}_{2}{{{{\rm{S}}}}}_{6}^{4-}$ vibration dominates Na^+^ transport (~0.7 orders of magnitude). Na_1__1_Sn_2_PS_1__2_ differs from the other two thiophosphates; rotation, rather than vibration, dominates Na^+^ transport (~0.4 orders of magnitude).

In the borohydrides LiBH_4_ and Na_2_H_1__0_H_1__0_, cation mobility exhibits the strongest responses to anion dynamics among the six SEs studied. Notably, the rotational constraint reduces the conductivity in every one of the six SEs, demonstrating that the long-debated paddle-wheel effect is universally operative, even though its enhancement is not always the largest among the three motion modes. Across these six SEs, all three motion modes (rotation, translation, and vibration) emerge as dominant for cation transport. This reveals that, in plastic crystals, polyanion reorientation does not necessarily dominate ionic conductivity; no single polyanion motion mode universally dominates cation transport.

### Anion rotation enhances cooperative transport in borohydrides

**Fig. 3:**
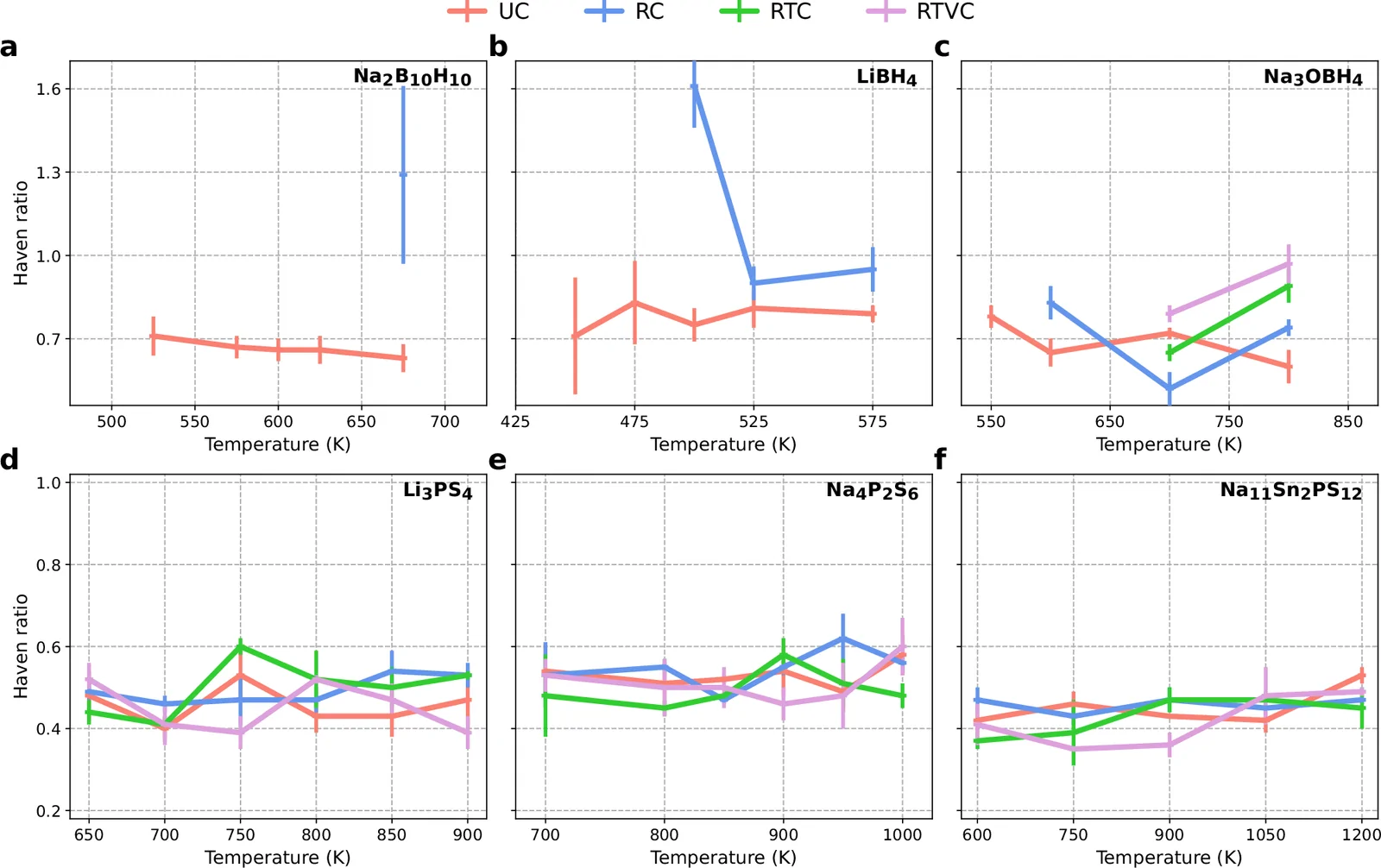
Haven ratios under different constraints for six SEs. **a** Na_2_B_1__0_H_1__0_, **b** LiBH_4_, **c** Na_3_OBH_4_, **d** Li_3_PS_4_, **e** Na_4_P_2_S_6_, and **f** Na_1__1_Sn_2_PS_1__2_ under unconstrained (UC, red), rotationally constrained (RC, blue), roto-translationally constrained (RTC, green), and roto-translationally-vibrationally constrained (RTVC, purple) conditions at various temperatures. Data are absent where no cation diffusion was observed within the simulation time. Error bars represent the standard error of the mean over blocks from multiple independent molecular dynamics trajectories (six trajectories for Na_3_OBH_4_ and three trajectories each for Na_2_B_1__0_H_1__0_, LiBH_4_, Li_3_PS_4_, Na_4_P_2_S_6_, and Na_1__1_Sn_2_PS_1__2_). Source data are provided as a Source Data file.

The conductivities calculated using the Nernst–Einstein approximation (equation (3)) are shown in Supplementary Fig. 20. For every conductor, the NE conductivity differs substantially from the EH conductivity, but both yield the same dominant anion motion mode. The Haven ratio, defined as the ratio of NE conductivity to EH conductivity, quantifies the deviation of EH conductivity from NE conductivity and, more importantly, it characterizes how cations move cooperatively. Most of the Haven ratios shown in Fig. 3 are smaller than 1, indicating the presence of cation–cation correlation and the resulting cooperative transport. Moreover, the three thiophosphates exhibit lower Haven ratios than the two borohydrides and the antiperovskite, indicating stronger cooperative transport in the thiophosphates. For the three thiophosphates and Na_3_OBH_4_, the Haven ratio remains within a narrow range under all four constraint conditions and across the entire simulated temperature range. For Na_2_B_1__0_H_1__0_ and LiBH_4_, the Haven ratio shows similarly weak temperature dependence, but increases when anion rotation is constrained. This trend suggests that anion rotation strengthens the cooperative transport of Li^+^/Na^+^ in LiBH_4_ and Na_2_B_1__0_H_1__0_, thereby enhancing conductivity.

### The paddle-wheel effect dominates only under frequency matching

**Fig. 4:**
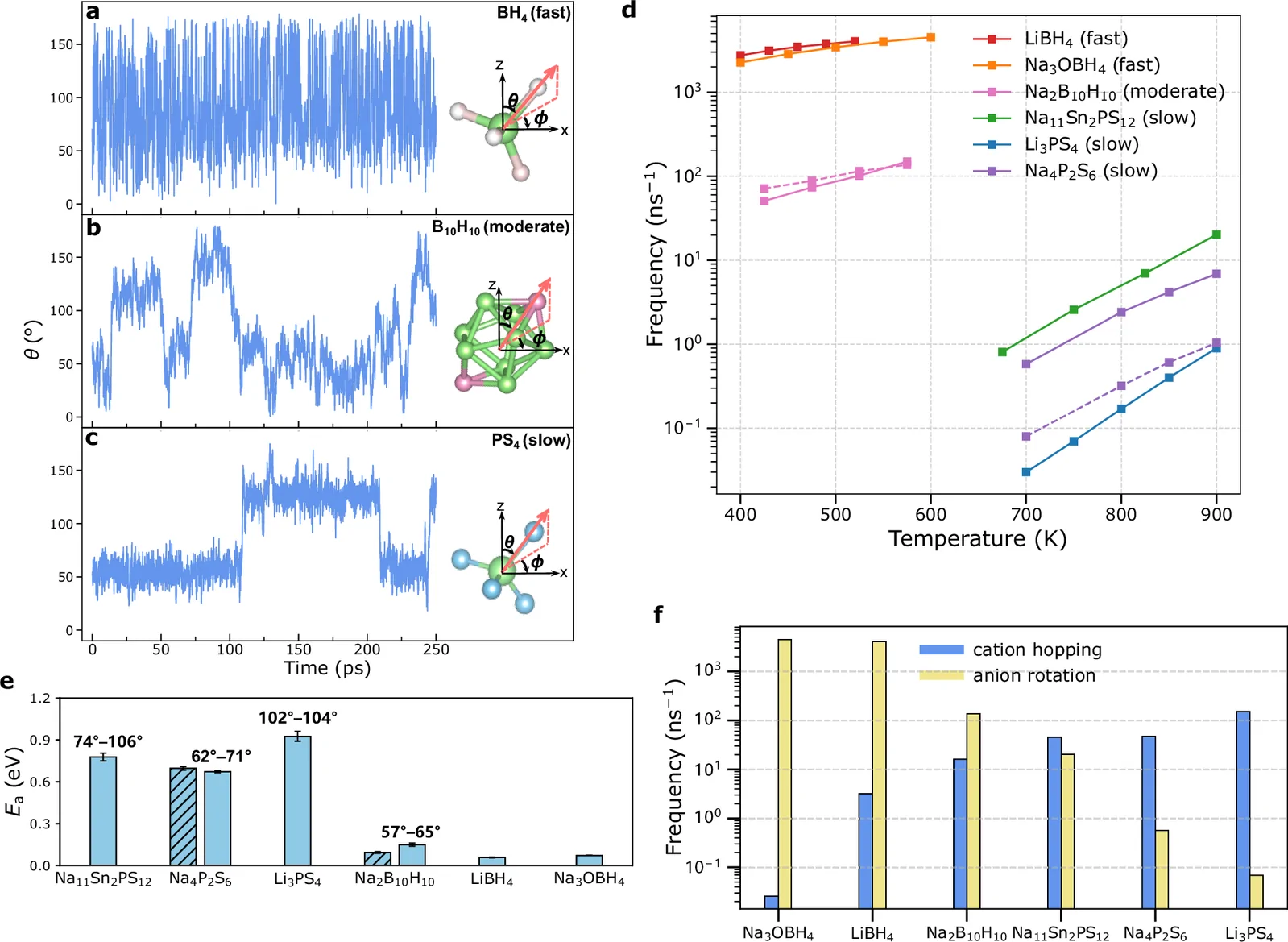
Polyanion rotation dynamics. Polar angle of **a** ${{{{\rm{BH}}}}}_{4}^{-}$ in LiBH_4_ at 500 K, **b** ${{{{\rm{B}}}}}_{10}{{{{\rm{H}}}}}_{10}^{2-}$ in Na_2_B_1_0H_1_0 at 550 K, and **c** ${{{{\rm{PS}}}}}_{4}^{3-}$ in Na_1_1Sn_2_PS_1_2 at 900 K, representing fast, moderate, and slow reorientational dynamics, respectively. Coordinates for the rotors are defined as shown in the insets. In **b**, **m** denotes the center of mass of the ${{{{\rm{B}}}}}_{10}{{{{\rm{H}}}}}_{10}^{2-}$, and the apical and equatorial B atoms are represented by the pink and green spheres, respectively. **d** Anion reorientation frequencies in the six SEs at various temperatures. For Na_4_P_2_S_6_ and Na_2_B_1__0_H_1__0_, solid lines correspond to reorientation of the line connecting the two P atoms in ${{{{\rm{P}}}}}_{2}{{{{\rm{S}}}}}_{6}^{4-}$ and the two apical B atoms in ${{{{\rm{B}}}}}_{10}{{{{\rm{H}}}}}_{10}^{2-}$, respectively. Dashed lines correspond to the internal rotation of ${{{{\rm{P}}}}}_{2}{{{{\rm{S}}}}}_{6}^{4-}$ and rotation about the line connecting the two apical B atoms, respectively. **e** Activation energies (*E*_a_) of anion reorientation, obtained from Arrhenius fits of the reorientation frequencies in **d**. For Na_4_P_2_S_6_ and Na_2_B_1__0_H_1__0_, two reorientation modes are shown. Solid bars correspond to reorientation of the principal axis (P–P in ${{{{\rm{P}}}}}_{2}{{{{\rm{S}}}}}_{6}^{4-}$, apical B–B in ${{{{\rm{B}}}}}_{10}{{{{\rm{H}}}}}_{10}^{2-}$); hatched bars correspond to internal rotation of ${{{{\rm{P}}}}}_{2}{{{{\rm{S}}}}}_{6}^{4-}$ and rotation of ${{{{\rm{B}}}}}_{10}{{{{\rm{H}}}}}_{10}^{2-}$ about this axis. Numbers above the bars indicate the temperature-dependent ranges of reorientation angles fitted using the Ivanov model. No values are reported for LiBH_4_ and Na_3_OBH_4_, because their reorientations are difficult to define as discrete jumps. Error bars represent the standard error of *E*_a_ from the least-squares linear fit (*n* = 4–5 temperature points). **f** Cation hopping frequencies (blue) and anion reorientation frequencies (yellow) for six ionic conductors. Frequencies were evaluated at 520–600 K for the borohydride and antiperovskite systems and at 700–900 K for the thiophosphates. Source data are provided as a Source Data file.

Following the angle-projection approach of Scholz et al.^24^, we track the reorientation dynamics of each rotor by monitoring the polar and azimuthal angles (*θ* and *ϕ*) defined with respect to a reference frame parallel to the lattice vectors (insets of Fig. 4a–c). ${{{{\rm{B}}}}}_{10}{{{{\rm{H}}}}}_{10}^{2-}$ in Na_2_B_1__0_H_1__0_ shows brief residence at preferred orientations on the order of tens of picoseconds, but reorientation remains facile on the picosecond timescale overall (Fig. 4b). ${{{{\rm{PS}}}}}_{4}^{3-}$ in Na_1__1_Sn_2_PS_1__2_, by contrast, exhibits slower transitions among preferred orientations with long residence times ( ~100 ps) (Fig. 4c). ${{{{\rm{BH}}}}}_{4}^{-}$ in LiBH_4_, on the other end, rotates nearly freely, with negligible residence time (Fig. 4a). Based on these observations, we extracted the reorientation frequencies of the anion rotors across all six SEs over a range of temperatures using the Ivanov model (Fig. 4d). Here, reorientation refers to large-amplitude jumps between preferred orientations, not the fast libration within a single orientation. The reorientation frequencies span five orders of magnitude across the six SEs, from ~10^3^ ns^−1^ in the fast rotors (LiBH_4_, Na_3_OBH_4_) down to ~10^−2^ ns^−1^ in the slowest (*α*-Li_3_PS_4_), with Na_2_B_1__0_H_1__0_ falling in between. Two types of rotation in ${{{{\rm{B}}}}}_{10}{{{{\rm{H}}}}}_{10}^{2-}$ were considered separately: rotation about the axis connecting the apical B atoms and reorientation of the axis itself. Both occur at comparable frequencies (pink dashed and solid lines in Fig. 4d). For ${{{{\rm{P}}}}}_{2}{{{{\rm{S}}}}}_{6}^{4-}$ in Na_4_P_2_S_6_, internal rotation occurs at a lower frequency than the P–P bond reorientation (purple dashed and solid lines in Fig. 4d). The angular distribution and its analysis are provided in Section 3 of the Supplementary Information.

We also evaluate the cation hop frequencies in the six SEs (equation (9)). For Na_3_OBH_4_, defect formation energies are first calculated to identify the dominant defects (Supplementary Fig. 6). Under most Na-poor conditions, V_Na_ and V_BH4_ are energetically favored, while Na_i_ has much higher formation energy and is excluded. We thus introduce Na vacancies. Detailed results about defect physics are provided in Section 2 in the Supplementary Information. Hop and reorientation frequencies are shown in Fig. 4f. The two frequencies differ by more than three orders of magnitude in Na_3_OBH_4_ and LiBH_4_, and by at least two in Na_4_P_2_S_6_ and Li_3_PS_4_. In Na_2_B_1__0_H_1__0_ and Na_1__1_Sn_2_PS_1__2_, the large-amplitude reorientations of the rotors (57–65^∘^ for B_1__0_H_1__0_, 74–107^∘^ for PS_4_) occur at frequencies comparable to those of cation hopping (Fig. 4f), in line with the dominant role of rotation in cation transport in these SEs (Fig. 2a, f). The paddle-wheel effect thus dominates conductivity only when these two frequencies coincide.

Figure 4 e shows that the anion reorientation barrier increases from  ~0.06 eV for ${{{{\rm{BH}}}}}_{4}^{-}$ to  ~0.1 eV for ${{{{\rm{B}}}}}_{10}{{{{\rm{H}}}}}_{10}^{2-}$, and reaches 0.7–0.9 eV for the P/S-based rotors. Notably, although ${{{{\rm{B}}}}}_{10}{{{{\rm{H}}}}}_{10}^{2-}$ has a reorientation barrier comparable to that of ${{{{\rm{BH}}}}}_{4}^{-}$, its frequency is ~1.6 orders of magnitude lower, in part due to larger moment of inertia (*I*_*A*_ = 192, *I*_*B*_ = *I*_*C*_ = 249 amu ⋅ Å^2^ for ${{{{\rm{B}}}}}_{10}{{{{\rm{H}}}}}_{10}^{2-}$, versus 4 amu ⋅ Å^2^ for the spherical top ${{{{\rm{BH}}}}}_{4}^{-}$). This points to two design principles for maximizing the paddle-wheel effect’s contribution: the cation hop and anion reorientation frequencies should be matched, which in turn requires balancing the reorientational barrier imposed by the host lattice and the moment of inertia of the rotor itself.

### Anion dynamics tune conductivity through barrier and prefactor

**Fig. 5:**
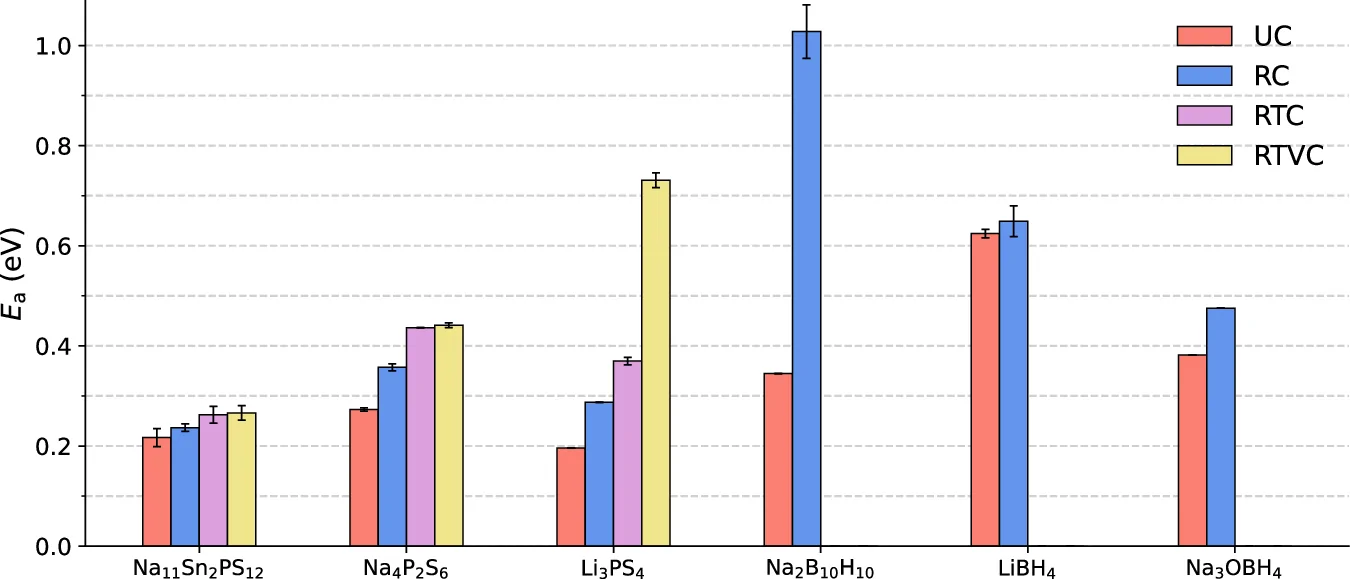
Activation energies for six SEs. Activation energies derived from linear Arrhenius fits of six superionic conductors under unconstrained (UC, red), rotationally constrained (RC, blue), roto-translationally constrained (RTC, purple), and roto-translationally-vibrationally constrained (RTVC, yellow) conditions; some are absent due to the diffusion coefficients over a wide temperature range cannot be obtained within the simulation timescale. Error bars represent the standard error of *E*_a_ from the least-squares linear fit (*n* = 3–5 temperature points). Source data are provided as a Source Data file.

To gain a deeper insight into the mechanism underlying dynamic cation–anion coupling, we calculated the activation energy (Fig. 5). Beyond rotation, polyanion translation and vibration can also contribute to lowering the migration barrier. For Na_2_B_1__0_H_1__0_ and Li_3_PS_4_, the dominant anion motions affect conductivity mainly through the activation barrier; constraining ${{{{\rm{B}}}}}_{10}{{{{\rm{H}}}}}_{10}^{2-}$ rotation and ${{{{\rm{PS}}}}}_{4}^{3-}$ vibration lowers the conductivity by ~3 and ~1 orders of magnitude, respectively, consistent with the corresponding sharp increases in migration activation energy. In contrast, in Na_4_P_2_S_6_ and LiBH_4_, constraining ${{{{\rm{P}}}}}_{2}{{{{\rm{S}}}}}_{6}^{4-}$ vibration and ${{{{\rm{BH}}}}}_{4}^{-}$ rotation, respectively, decreases the conductivity by ~0.8 and ~1 order of magnitude, despite very small increases in *E*_a_ of only 0.0048 and 0.0246 eV. This implies that the conductivity decrease mainly originates from changes in the Arrhenius prefactor rather than in the activation barrier. These results indicate that anion dynamics can influence ionic conductivity by altering both the activation energy and the prefactor, with the latter likely arising from changes in the attempt frequency and migration entropy.

### Anion dynamics drive superionicity via coupling and disorder

**Fig. 6:**
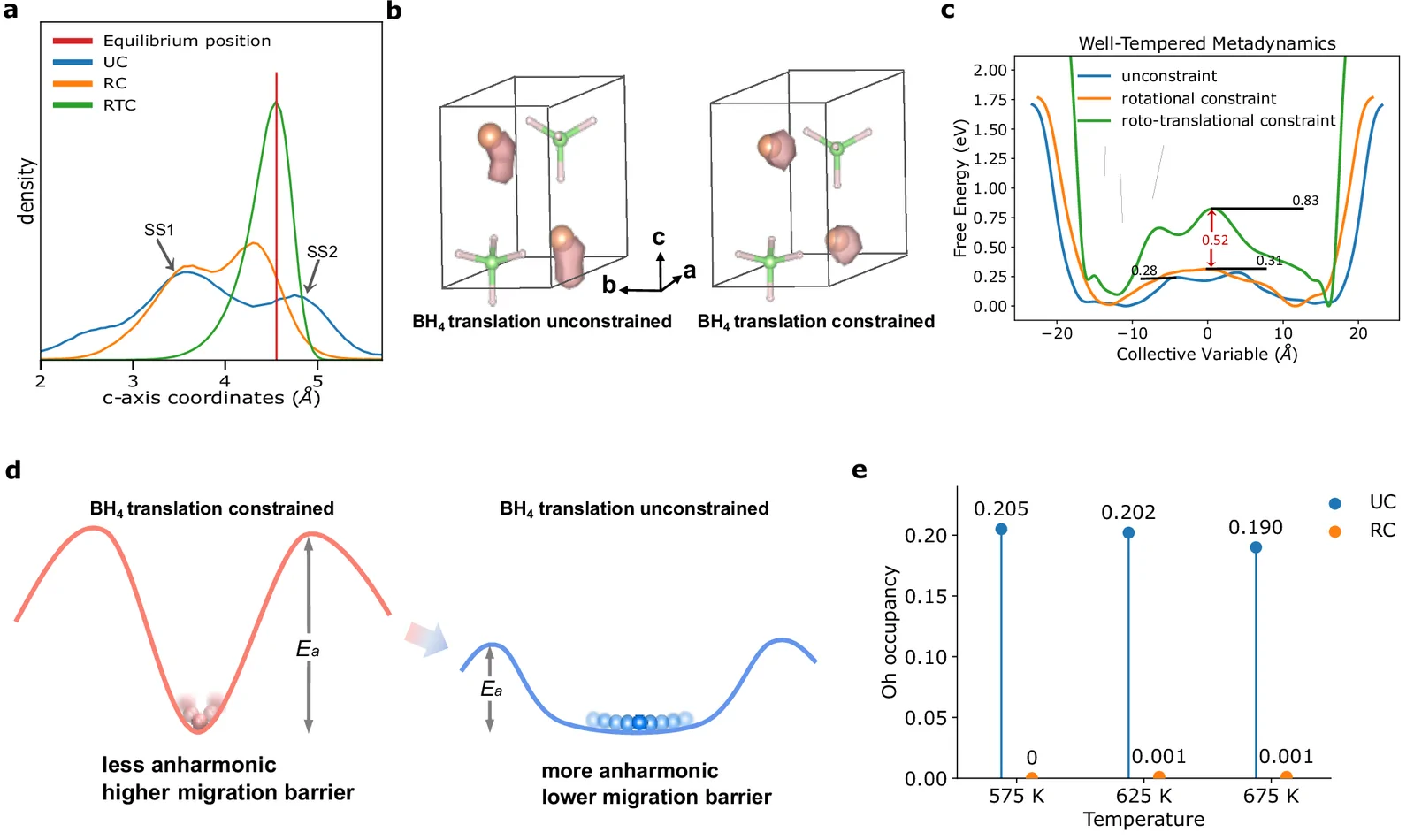
Influence of anion dynamics on the cation energy landscape and on-site occupancy. **a** Statistical distribution of the Li ions along *c* axis in LiBH_4_ under unconstrained (UC, blue), rotationally constrained (RC, orange), and roto-translationally constrained (RTC, green) conditions at 550 K, along with the equilibrium lattice positions (red). SS1 and SS2 denote split sites 1 and 2, respectively. The distribution under RTVC overlapping with that under RTC is not shown. **b** The probability densities of Li ions in LiBH_4_ obtained from MLMD simulations at 550 K, with ${{{{\rm{BH}}}}}_{4}^{-}$ translation (RC) and without ${{{{\rm{BH}}}}}_{4}^{-}$ translation (RTC), shown with an isosurface value of 0.045. **c** Free energy profile along the collective variable obtained from well-tempered metadynamics with UC (blue), RC (orange), and RTC (green) at 300 K for Li^+^ migration in LiBH_4_. The numbers indicate the free energy barriers (in eV) for cation migration. **d** Schematic of the cation migration energy landscape with BH_4_ translation constrained (left) and unconstrained (right). BH_4_ translation modulates the landscape, enhancing the anharmonicity and lowering the migration barrier *E*_a_. **e** Oh site occupancy of Na ions in Na_2_B_1__0_H_1__0_ with ${{{{\rm{B}}}}}_{10}{{{{\rm{H}}}}}_{10}^{2-}$ unconstrained (UC, blue) and rotationally constrained (RC, orange) at various temperatures. Source data are provided as a Source Data file.

Figure 6a shows the characteristic splitting of the Li site along the *c* axis into two split sites, denoted SS1 and SS2^60,61^. This double splitting plays an important role in the creation of the metastable state and migration through the connection path^62^. Under RC, the split undergoes a minor contraction but persists. In contrast, under RTC, the doubly split nature is completely suppressed. Additionally, in Fig. 6b, we visualize the Li probability densities with and without anion translation, clearly illustrating the disappearance of the double splitting when the anion translation is constrained. This demonstrates that BH_4_ translation enables Li to explore off-centered positions over a broader local region, giving rise to a flat-bottomed local free-energy landscape for Li. In contrast to the single-peaked distribution under RTC, the split distribution shows that BH_4_ translation enhances the anharmonicity of the local potential experienced by Li. To quantify this, we evaluate the degree of anharmonicity of the Li sublattice using equation (13), which has also been applied to an SE study^63^, obtaining 2.17 for the unconstrained system, 1.05 under RC, and 0.53 under RTC. The pronounced drop upon constraining BH_4_ translation (1.05 → 0.53) quantitatively confirms that anion translation enhances the anharmonicity of the local potential experienced by Li.

When BH_4_ translation is constrained, the conductivity drops by more than two orders of magnitude and becomes too low to be reliably extracted within the accessible simulation timescale, as discussed above, preventing a fit of the migration barrier. We therefore turn to metadynamics to directly compute the Li migration free-energy barrier. As shown in Fig. 6c, constraining only ${{{{\rm{BH}}}}}_{4}^{-}$ rotation changes the Li migration free-energy barrier by only ~0.03 eV, consistent with the small difference in the activation energies fitted from the UC and RC conductivities (Fig. 5). By contrast, further constraining BH_4_ translation increases the barrier by ~0.5 eV, demonstrating that BH_4_ translation is critical for reducing the Li migration barrier. These results reveal that BH_4_ translation enhances anharmonicity and is critical for Li transport by lowering the migration barrier, as schematically illustrated in Fig. 6d.

**Fig. 7:**
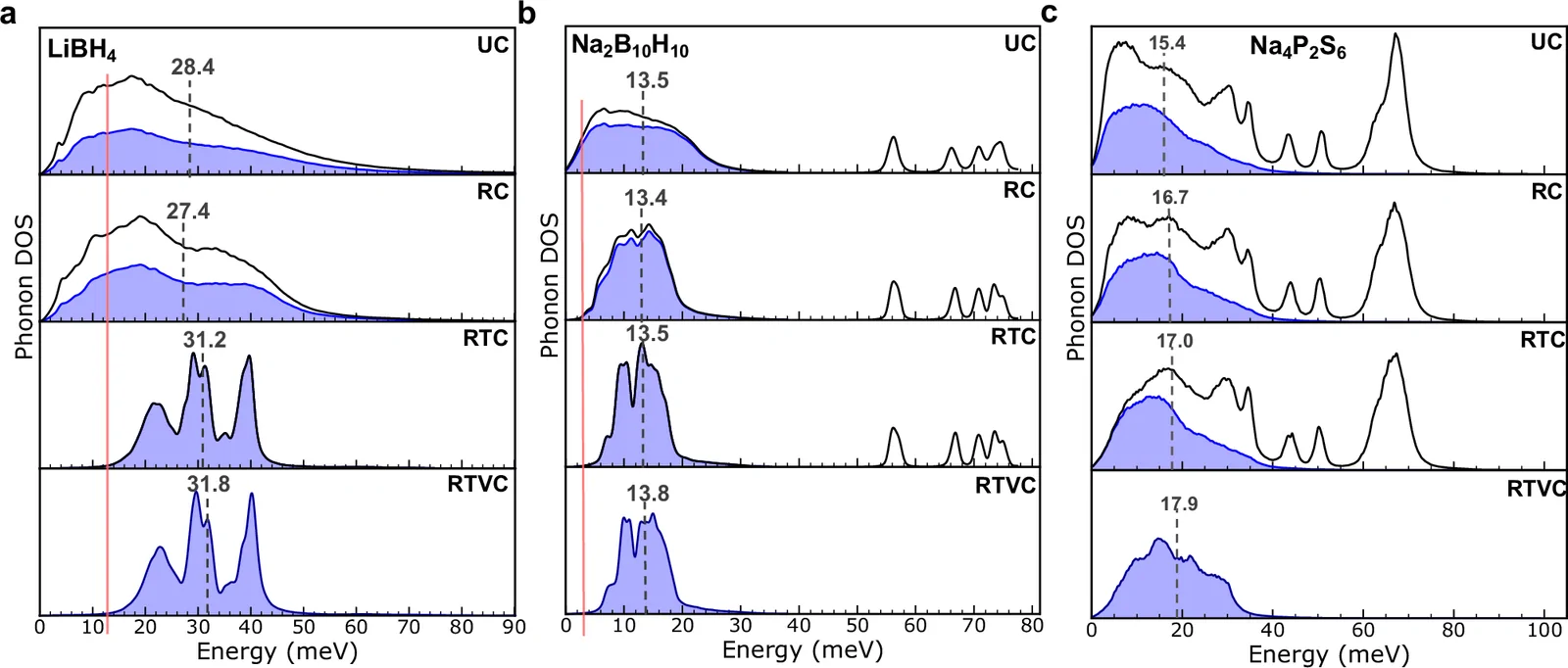
Phonon DOS under different constraints. **a** LiBH_4_ at 550 K, **b** Na_2_B_10_H_10_ at 350 K, and **c** Na_4_P_2_S_6_ at 800 K under unconstrained (UC), rotationally constrained (RC), roto-translationally constrained (RTC), and roto-translationally-vibrationally constrained (RTVC) conditions. The blue shaded areas correspond to the partial phonon DOS of the mobile cation; black curves show the total phonon DOS. The number and dashed line represent the cation band center. The red line indicates the emergence of the low-frequency component of the partial phonon DOS of the cation when anion dynamics are present. Higher frequency peaks are not plotted for clarity. Source data are provided as a Source Data file.

The minor change in the partial phonon DOS of Li from RTVC to RTC indicates that BH_4_ internal vibrations do not substantially alter the partial phonon DOS of Li (Fig. 7a). In contrast, BH_4_ translation induces a drastic change in the partial phonon DOS of Li, making it more diffuse and suppressing the sharp peaks. This indicates a strong coupling between BH_4_ translation and Li vibrational dynamics. This change can in part be attributed to anharmonic effects. Meanwhile, BH_4_ translation shifts the band center of the partial phonon DOS of Li toward lower frequency and increases its low-frequency intensity, reflecting softened Li dynamics that can facilitate large-amplitude local motion.

Partial occupancy of Na sites^19^ and the presence of multiple possible occupation patterns among the eight Td sites surrounding an occupied Oh site^12^ strongly indicate disorder in the Na sublattice. Our occupancy analysis (Fig. 6e) corroborates this picture: ~20% of Na reside on Oh sites while the remainder occupy Td sites when no constraint is applied, consistent with a disordered Na sublattice. In contrast, constraining the rotation of ${{{{\rm{B}}}}}_{10}{{{{\rm{H}}}}}_{10}^{2-}$ reduces the Oh occupancy by more than two orders of magnitude (to ~0.1%). This reveals that anion reorientational disorder directly induces cation disorder, thereby enabling superionicity. As for the partial phonon DOS of Na, anion rotation strongly modifies the partial phonon DOS of Na, making it more diffuse and giving rise to a low-frequency component (Fig. 7b).

For the thiophosphate-based SEs, the partial phonon DOS of the cation shows different degrees of sensitivity to anion dynamics. For Na_4_P_2_S_6_, the partial phonon DOS of Na shows a pronounced response to P_2_S_6_ vibrations, and the overall P_2_S_6_ dynamics shift its band center toward lower frequency (Fig. 7c). For Li_3_PS_4_, the partial phonon DOS of Li is already diffuse within the static anion framework under RTVC (Supplementary Fig. 33a), and PS_4_ dynamics further shift the band center toward lower frequency. The Li–Li PDF shows the gradual opening of Li-ion transport channels by each anion motion mode (Supplementary Fig. 34). In contrast, the partial phonon DOS of Na in Na_1__1_Sn_2_PS_1__2_ changes only weakly under different anion constraints, and its band center remains nearly unchanged (Supplementary Fig. 33b).

## Discussion

Using a constraint algorithm and machine learning interatomic potentials, we quantify the contribution of each anion motion mode to the Einstein–Helfand conductivity across six representative plastic crystals. Although the paddle-wheel effect is universally operative in plastic crystals, no single anion motion mode universally dominates cation transport. Anion translation and vibration can also dominate, and even govern, cation transport. This demands a broader view of cation–anion coupling, beyond the long-standing focus on rotation as the most visually compelling mechanism of cation hopping. More broadly, quantifying the polyanion–cation dynamic coupling provides a complementary perspective to phonon–ion coupling on ion transport.

To understand the origin of these conductivity changes, we examine the activation barrier and the prefactor. Lowering the activation barrier has long been a central strategy in the design of SEs, and we find that anion dynamics can substantially modify this barrier. However, anion dynamics can also alter conductivity substantially without significantly changing the activation barrier, indicating that the prefactor, likely through the attempt frequency and migration entropy, is comparably important. These findings call for SE design strategies that target both the activation barrier and the prefactor. Beyond this single-ion picture, all six conductors exhibit Haven ratios below unity, indicating that cooperative cation motion is common across plastic-crystal SEs. In the borohydrides, anion rotation reinforces this cooperative motion, suggesting another way in which rotation can enhance ionic conductivity.

Finally, anion dynamics can induce dynamic cation disorder, which alone can enhance conductivity by over two orders of magnitude. This disorder takes two distinct forms: anharmonic broadening of cation positions around their lattice sites, and partial occupancy across multiple crystallographic sites. Both differ fundamentally from site disorder. This suggests that SE design should target the dynamic anion–cation couplings that activate such disorder, opening additional pathways for fast ion transport.

## Methods

### Machine learning interatomic potential

Geometry optimizations and single-point calculations were performed using the projector augmented-wave (PAW)^64,65^ method and the Perdew–Burke–Ernzerhof (PBE)^65^ functional in the Vienna ab initio simulation package (VASP) 5.4.4^66,67^. The VASP PAW potentials treat 1, 1, 3, 1, 6, 5, 6, and 4 valence electrons for Li, Na, B, H, O, P, S, and Sn, respectively. The electronic self-consistency convergence criterion was set to 10^−6^ eV. The convergence tests for **k**-point sampling and energy cutoff were carried out to ensure that forces are converged to less than 1 meV/Å. Specifically, the plane-wave cutoffs are 650 eV for Na_2_B_1__0_H_1__0_ and LiBH_4_, 600 eV for Na_3_OBH_4_, 500 eV for Li_3_PS_4_ and Na_4_P_2_S_6_, and 400 eV for Na_1__1_Sn_2_PS_1__2_. The Γ-centered **k**-point meshes are 2 × 2 × 2 for Na_2_B_1__0_H_1__0_, LiBH_4_, and Li_3_PS_4_; 3 × 3 × 3 for Na_3_OBH_4_ and Na_4_P_2_S_6_; and 2 × 2 × 1 for Na_1__1_Sn_2_PS_1__2_. Due to the relatively small number of atoms in the unit cells of LiBH_4_ and Na_3_OBH_4_, we begin with their 2 × 2 × 1 and 2 × 2 × 2 supercells, respectively. Structure manipulation was dealt with pymatgen^68^.

The DP-GEN scheme (version 0.12.1)^69^ was adopted for the deep potential (DP)^56^ model generation. During each iteration, four models are generated, and the *σ*_high_ and *σ*_low_ are set to 0.26 and 0.11, respectively, to evaluate model deviation. A cut-off radii of 7 Å is selected for training, with 400,000 training steps. The exploration phase uses LAMMPS (version 2 Aug 2023)^70^, with MD steps increasing from 1500 to 10,000. Six temperatures are set, with the highest and lowest temperatures being, respectively, higher and lower than the temperature range used for production MD runs. After exploration, hundreds of candidate configurations are chosen for labeling. The exploration of each system is considered converged when the percentage of accurate configurations reaches 99.5%. After the models trained on unit cells (supercells for LiBH_4_ and Na_3_OBH_4_) converged, we expanded Na_2_B_1__0_H_1__0_, LiBH_4_, Na_3_OBH_4_, Li_3_PS_4_, and Na_4_P_2_S_6_ to 2 × 1 × 1, 3 × 3 × 2, 3 × 3 × 3, 2 × 2 × 2, and 2 × 2 × 2 supercells, respectively, repeating the process until the same convergence criteria were met. For Na_1__1_Sn_2_PS_1__2_, which already has a large number of atoms in its unit cell and is highly time-consuming, no expansion was performed. For Na_3_OBH_4_, we first carry out the DP-GEN iterations on the host supercell. After convergence, the MD exploration is performed on a supercell containing one Na vacancy, which converges without triggering any additional labeling. Consequently, the training set consists solely of host supercells. Finally, six model ensembles, each containing four models, were obtained.

To build the test set, configurations are sampled from MD trajectories spanning the temperature range of the production simulations, combining AIMD with longer DP-driven MD, so as to cover a broad and physically representative region of configuration space. All trajectories start from perturbed structures generated using DP-GEN, in which the cell matrix *L* is transformed into (*I* + *P*)*L* with each element of *P* drawn uniformly from [ −0.02, 0.02], and every atomic coordinate is independently displaced by a random value in [−0.2, 0.2] Å.

Taking Na_3_OBH_4_ as an example, its test set is built on the 3 × 3 × 3 supercell and combines an AIMD set with an MLIP-MD set. For the former, 200 perturbed structures were each run for 0.2 ps of AIMD (150–1000 K), and the final frame of each was retained, giving 200 DFT-labeled configurations. For the latter, 30 perturbed structures were each propagated for 100 ps with the current DP model over the same temperature range and sampled every 1 ps, yielding 3000 configurations; following Imbalzano et al.^71^, a global fingerprint was obtained for each by summing the local fingerprints of all atomic environments, reduced by principal component analysis, and 300 representative structures were selected by farthest-point sampling and labeled by DFT single-point calculations. The final test set thus comprises 200 AIMD and 300 MLIP-MD configurations.

### Molecular dynamics simulation

MD simulations were performed with the trained DP models using a modified version of CP2K v2024.2^72^. The selective constraints on rotor anion motion modes were implemented following the constraint algorithm developed in our previous work^57^. In RC and RTC simulations, we explicitly constrain each rotor anion’s 3 rotational degrees of freedom (DOF) and 3 translational+3 rotational DOF, respectively. In RTVC simulations, we constrain all DOF of each rotor anion. For Na_3_OBH_4_, six 20 ns simulations with different initial velocities were performed for each temperature and constraint using a 7 × 7 × 7 supercell containing 3087 atoms, in which 12 Na vacancies were introduced. For Na_2_B_1__0_H_1__0_, LiBH_4_, Li_3_PS_4_, Na_4_P_2_S_6_, and Na_1__1_Sn_2_PS_1__2_, three independent 50 ns simulations were performed in supercells containing 1056, 768, 864, 648, and 832 atoms, respectively. All systems were first equilibrated and then simulated in the canonical (NVT) ensemble using a Nosé–Hoover thermostat^73,74^ with a time constant of 100 fs and a chain length of 3. The equations of motion were integrated using the velocity Verlet algorithm. A time step of 0.5 fs was used for the hydrogen-containing materials and 2 fs for the thiophosphates. The conductivity is calculated through the Einstein–Helfand relation^75^:

1 $${\sigma }_{{{{\rm{HE}}}}}=\frac{1}{3\Omega {{{{\rm{k}}}}}_{{{{\rm{B}}}}}T}{{\lim }_{t\to \infty }}\frac{\langle | {\sum }_{i\in C\cup F}{q}_{i}({{{{\bf{r}}}}}_{i}^{{{{\rm{bar}}}}}(t)-{{{{\bf{r}}}}}_{i}^{{{{\rm{bar}}}}}(0)){| }^{2}\rangle }{2t}$$

where 〈 ⋅ 〉 denotes an equilibrium ensemble average, *Ω* and *T* are the system volume and temperature, k_B_ is the Boltzmann constant, *C* and *F* denote the mobile carrier sublattice and solid-matrix framework, ${{{{\bf{r}}}}}_{i}^{{{{\rm{bar}}}}}$ denotes the position of the *i*-th particle in the barycentric reference frame, and *q*_*i*_ denotes the charge of the *i*-th particle. It can be rigorously shown that *q*_*i*_ can be expressed in terms of integer-valued, scalar, and time-independent atomic oxidation numbers^76^. Thanks to gauge invariance, given that the system is charge-neutral, an equivalent way to evaluate the conductivity is

2 $${\sigma }_{{{{\rm{HE}}}}}=\frac{1}{3\Omega {{{{\rm{k}}}}}_{{{{\rm{B}}}}}T}{{\lim }_{t\to \infty }}\frac{\langle | {\sum }_{i\in C}{q}_{i}({{{{\bf{r}}}}}_{i}^{{{{\rm{mat}}}}}(t)-{{{{\bf{r}}}}}_{i}^{{{{\rm{mat}}}}}(0)){| }^{2}\rangle }{2t}$$

where ${{{{\bf{r}}}}}_{i}^{{{{\rm{mat}}}}}$ denotes the position of particle in the solid-matrix reference frame. Expanding the squared term inside ensemble average gives diagonal and off-diagonal terms. It is common practice to retain only the diagonal terms, which leads to the Nernst-Einstein approximation:

3 $${\sigma }_{{{{\rm{NE}}}}}=\frac{N{q}_{i}^{2}}{\Omega {{{{\rm{k}}}}}_{{{{\rm{B}}}}}T}{D}_{{{{\rm{tr}}}}}$$

where *N* is the number ions in the simulation box. The tracer diffusion coefficient ${D}_{{{{\rm{tr}}}}}$ is defined as

4 $${D}_{{{{\rm{tr}}}}}={{\lim }_{t\to \infty }}\frac{\frac{1}{N}{\sum }_{i}^{N}\langle {\left\vert {{{{\bf{r}}}}}_{i}(t)-{{{{\bf{r}}}}}_{i}(0)\right\vert }^{2}\rangle }{6t}$$

The Haven ratio is then defeined as

5 $${H}_{{{{\rm{R}}}}}=\frac{{\sigma }_{{{{\rm{NE}}}}}}{{\sigma }_{{{{\rm{EH}}}}}}$$

When the tracer diffusion coefficient is around 10^−7^ cm^2^ s^−1^, MD of thousands of atoms over 10 ns typically yield results with minimal error^55^. Here, we adopt 10^−8^ cm^2^ s^−1^ as the lower threshold for a reliable tracer diffusion coefficient, which typically corresponds to an *σ*_NE_ on the order of 0.1 mS cm^−1^. For the conductivity calculated from the EH relation, we also adopt 0.1 mS cm^−1^ as the lower threshold, based on a previous study that reported conductivities on the order of 0.1 mS cm^−1^^30^.

The average residence times and jump amplitudes associated with the reorientation of ${{{{\rm{PS}}}}}_{4}^{3-}$ and ${{{{\rm{P}}}}}_{2}{{{{\rm{S}}}}}_{6}^{4-}$, as well as with the reorientation of the apical B–B axis of ${{{{\rm{B}}}}}_{10}{{{{\rm{H}}}}}_{10}^{2-}$, are extracted by fitting the Ivanov model^77^. Specifically, the first- and second-rank orientational correlation functions were fitted as

6 $${C}_{1}(t)=\exp \left[-\frac{t}{\tau }\left(1-\cos \Delta \right)\right],$$

and

7 $${C}_{2}(t)=\exp \left[-\frac{3t}{2\tau }\left(1-{\cos }^{2}\Delta \right)\right],$$

where *τ* is the average residence time between two jumps and Δ is the jump amplitude. The corresponding reorientation frequencies are then obtained as the inverse of the average residence time. The reorientation frequencies *ν*_rot_ of ${{{{\rm{BH}}}}}_{4}^{-}$, and of ${{{{\rm{B}}}}}_{10}{{{{\rm{H}}}}}_{10}^{2-}$ about its apical B–B axis, are evaluated directly as the inverse of the orientational correlation time^78^

8 $${\nu }_{{{{\rm{rot}}}}}=\left(\int_{0}^{\infty }\left\langle {P}_{1}(\cos [\theta (t)])\right\rangle dt\right)^{-1}.$$

where *P*_1_ is the first-order Legendre polynomial, and *θ*(*t*) is the angle between a given B–H bond at time *t* and at *t* = 0. The cation hop frequency *ν*_hop_ is estimated by

9 $${\nu }_{{{{\rm{hop}}}}}=\frac{6{D}_{{{{\rm{tr}}}}}}{{\lambda }^{2}}$$

*λ* is the distance between nearest cations in the equilibrium structure.

The phonon density of states is calculated by ref. ^79^

10 $${{\mathrm{PDOS}}}\,(\omega )=\int\frac{\left\langle {\sum }_{s}{m}_{s}{{{{\bf{v}}}}}_{s}\left({t}_{0}\right)\cdot {{{{\bf{v}}}}}_{s}\left({t}_{0}+t\right)\right\rangle }{\left\langle {\sum }_{s}{m}_{s}{{{{\bf{v}}}}}_{s}\left({t}_{0}\right)\cdot {{{{\bf{v}}}}}_{s}\left({t}_{0}\right)\right\rangle }{e}^{-i\omega t}dt$$

where *s* runs over all atoms. If it runs over a specific type of atom, the atom-projected PDOS is obtained. The band center is defined as^80^

11 $${\omega }_{{{{\rm{av}}}}}=\frac{\int\omega \times {{\mathrm{PDOS}}}\,(\omega )d\omega }{\int{{\mathrm{PDOS}}}\,(\omega )d\omega }$$

Well-tempered metadynamics^81^ simulations were performed to obtain the free energy profile of Li-ion migration in LiBH_4_. Three nearest-neighbor Li sites along the c-axis were considered and labeled I, II, and III. In the initial configuration, sites II and III are occupied by two Li ions, denoted Li(II) and Li(III) after their starting sites, while site I is vacant. The migration consists of a concerted displacement of Li(II) and Li(III) as a rigid pair, in which Li(II) moves into site I and Li(III) into site II, leaving site III vacant. The two end states are related by symmetry and the process is reversible, so the resulting free energy profile is symmetric. The surrounding ${{{{\rm{BH}}}}}_{4}^{-}$ were used as an internal reference frame. The collective variable was defined as

12 $$d({{{\rm{site(II),\; Li(II)}}}})+d({{{\rm{site(III),\; Li(III)}}}})-d({{{\rm{site(I),\; Li(II)}}}})-d({{{\rm{site(II),\; Li(III)}}}})$$

where *d*(*A*, *B*) denotes the distance between *A* and *B*. The height of Gaussian is 0.0008 Ha, and the bias temperature Δ*T* is 4500 K.

We adopt the degree of anharmonicity introduced by Knoop et al.^82^ For each atom *I*, the anharmonic contribution to the force is defined as the difference between the force *F*_*I*_ in finite-temperature MD and its harmonic approximation ${F}_{I,\alpha }^{(2)}=-{\sum }_{J,\beta }{\Phi }_{\alpha,\beta }^{I,J}\Delta {R}_{J}^{\beta }$, where ${\Phi }_{\alpha,\beta }^{I,J}$ are the second-order force constants and $\Delta {R}_{J}^{\beta }$ is the instantaneous displacement of atom *J* from its equilibrium position at direction *β*. The degree of anharmonicity at temperature *T* is then

13 $${\sigma }^{{{{\rm{A}}}}}(T)=\sqrt{\frac{{\sum }_{I,\alpha }{\left\langle {\left({F}_{I,\alpha }(t)-{F}_{I,\alpha }^{(2)}(t)\right)}^{2}\right\rangle }_{T}}{{\sum }_{I,\alpha }{\left\langle {F}_{I,\alpha }^{2}(t)\right\rangle }_{T}}},$$

where 〈⋅〉_*T*_ denotes the ensemble average. Restricting the sums over *I* to the Li atoms yields the degree of anharmonicity of the Li sublattice reported here.

The second-order force constants ${\Phi }_{\alpha,\beta }^{I,J}$ were obtained from calculations on a 4 × 4 × 4 supercell using the finite displacement method implemented in phonopy^83^, based on the equilibrium structure relaxed until the residual forces fell below 10^−4^ eV/Å. *F*_*I*_ were sampled from MD trajectories of approximately 1.6 ns at 525 K (sampled every 0.5 ps). All force-constant and force evaluations were performed with our deep potential model.

### Defect calculations

Within the supercell approach, the formation energy of a defect *D* in charge state *q* is calculated by

14 $$\Delta {E}_{D,q}=({E}_{D,q}-{E}_{H})+\mathop{\sum}_{i}{n}_{i}{\mu }_{i}+q{E}_{F}+{E}_{{{{\rm{corr}}}}}$$

where *E*_*D*,*q*_ and *E*_*H*_ are the energies of the supercell containing defect *D* in charge state *q* and of the perfect supercell, resepectively. Here, *n*_*i*_ is the number of atoms of species *i* removed (*n*_*i*_ > 0) or added (*n*_*i*_ < 0) from the pristine supercell, *μ*_*i*_ is the corresponding chemical potential, *E*_*F*_ is the Fermi level, and *E*_corr_ is the finite-size charge correction. The native defects considered in Na_3_OBH_4_ include vacancies, antisite defects, and interstitials. Candidate Na and H interstitial sites were identified using the Voronoi tessellation scheme implemented in pylada-defects^84^. For each defect, charge states of *q* = −3, −2, −1, 0, +1, +2, +3 were considered. Supercell total energies were computed using the GGA-PBE functional and the projector augmented-wave (PAW) method as implemented in VASP. The total energies of 243-atom Na_3_OBH_4_ supercells were calculated using a plane-wave energy cutoff of 520 eV and a Γ-centered 3 × 3 × 3 Monkhorst–Pack **k**-point mesh. The atomic positions in all supercells were relaxed until the force on each atom was less than 0.01 eV Å^−1^.

The elemental chemical potentials are written as ${\mu }_{i}={\mu }_{i}^{0}+\Delta {\mu }_{i}$, where ${\mu }_{i}^{0}$ is the reference chemical potential of element *i* under standard conditions, and Δ*μ*_*i*_ is the deviation from this reference. The values of Δ*μ*_*i*_ are bounded by the phase diagram. When total-energy differences are taken between chemically and physically very different systems, the error cancellation is often incomplete^85^. Therefore, we used the fitted elemental chemical potentials ${\mu }_{i}^{0}$ reported by Liu et al., which were obtained by least-squares fitting to experimental formation enthalpies^86^. *E*_corr_ includes: (i) the eFNV correction^87^ for charged defects, including both image-charge correction and potential alignment, (ii) Moss–Burstein type band filling due to shallow defects, (iii) the correction of the band edges for shallow acceptors and donors. The defect calculations were set up and analyzed using doped 3.2.0^88^.

Most xc-functionals, in particular LDA and GGA functionals, severely underestimate the band gap, often by a factor of 2 or more^89^. The gold standard for quasiparticle band structure calculations of solids is the many-body GW method. In passing the partially self-consistent GW_0_, the deviation from experiments can fall below 0.2 eV^90^. The GW quasiparticle energies are calculated using a 6 × 6 × 6 Γ-centered **k**-point grid for primitive Na_3_OBH_4_, with the GW PAW potentials employed in the calculations. The DFT wavefunctions are used as the starting wavefunctions. The quasiparticle energies are iteratively updated until the difference in the band gap between two consecutive iterations is <0.05 eV. Finally, the GGA valence- and conduction-band edges are corrected separately using the corresponding GW quasiparticle energy shifts.

## Supplementary information

Supplementary Information

Transparent Peer Review file

## Source data

Source Data

## Data Availability

The trained DP models, the MSD and mean square dipole displacement data, and the MD configurations are available at https://doi.org/10.5281/zenodo.21831685^91^. The training and test datasets used in this work are available in a public Zenodo repository https://doi.org/10.5281/zenodo.21320507^92^. Source data are provided with this paper.

## Code availability

The source code for the constraint algorithm, the rotor reorientation frequency calculations, and the MSD calculations, together with the input files for the defect calculations, metadynamics simulations and MD simulations, is available at https://doi.org/10.5281/zenodo.21831685^91^.
