# Supplementary material for: Disentangling cation–polyanion coupling reveals which anion motion dominates cation transport in solid electrolytes
Source: Nat Commun. 2026 Sep 2;17:10455. doi: 10.1038/s41467-026-77273-x (PMC13635178; doi:10.1038/s41467-026-77273-x)
Supplement: Supplementary file 1 [file 41467_2026_77273_MOESM1_ESM.pdf]

# Supplementary Information

## Disentangling Cation–Polyanion Coupling Reveals Which Anion Motion Dominates Cation Transport in Solid Electrolytes

Ke Li[1], Jitai Yang[1], Yu Zhai[2], Hui Li[1,*]

[1]State Key Laboratory of Supramolecular Structure and Materials, Institute of Theoretical Chemistry, College of Chemistry, Jilin University, 2519 Jiefang Road, Changchun 130023, P.R. China

[2]Beijing National Laboratory for Molecular Sciences, Institute of Theoretical and Computational Chemistry, College of Chemistry and Molecular Engineering, Peking University, Beijing 100871, China

[*]Corresponding author email: prof_huili@jlu.edu.cn

## Table of Contents

Supplementary Note 1: Machine Learning Interatomic Potential . . . . . . . . . . . . . . . . . . . . . . 2
Supplementary Note 2: Defect physics of $Na_3OBH_4$ . . . . . . . . . . . . . . . . . . . . . . . . . . 13
Supplementary Note 3: Rotation Dynamics Statistics . . . . . . . . . . . . . . . . . . . . . . . . . . 15
Supplementary Note 4: Constrained Algorithm . . . . . . . . . . . . . . . . . . . . . . . . . . . . 20
Supplementary Note 5: Supplementary Figures . . . . . . . . . . . . . . . . . . . . . . . . . . . . 24

## Supplementary Note 1: Machine Learning Interatomic Potential

The number of configurations used for training separate model of each material is listed in the Supplementary Table 1.

**Supplementary Table 1** Sizes and compositions of the training and test datasets for the separate model trained for each material.

| Material | Training data | Test data |
|---|---|---|
| $Na_2B_{10}H_{10}$ | 2666 (1 × 1 × 1); 9547 (2 × 1 × 1) | 500 (2 × 1 × 1) |
| $LiBH_4$ | 7800 (2 × 2 × 1); 19661 (3 × 3 × 2) | 500 (3 × 3 × 2) |
| $Na_3OBH_4$ (host) | 6349 (2 × 2 × 2); 9804 (3 × 3 × 3) | 500 (3 × 3 × 3) |
| $Na_3OBH_4$ (defect) | — | 500 (3 × 3 × 3) |
| $Li_3PS_4$ | 4859 (1 × 1 × 1); 20024 (2 × 2 × 2) | 500 (2 × 2 × 2) |
| $Na_4P_2S_6$ | 5105 (1 × 1 × 1); 3714 (2 × 2 × 2) | 500 (2 × 2 × 2) |
| $Na_{11}Sn_2PS_{12}$ | 4917 (1 × 1 × 1) | 500 (1 × 1 × 1) |

The root mean square errors (RMSE) for energies and forces of the models for each system are listed in Supplementary Table 2. The RMSE for energies is mostly around 0.5–2 meV per atom, and for forces, it is several tens of meV/Å. We also observe that, for some systems, the force RMSE on the test set exceeds that on the training set. We attribute this to the active-learning scheme employed, which preferentially selects hard-to-fit configurations from the candidate pool [4]. Meanwhile, active-learning bias may be actively helpful when training overparameterized neural networks, counteracting the tendency toward higher training RMSEs and leading to test RMSEs comparable to or slightly higher than the training RMSEs [4].

**Supplementary Table 2** RMSE of energies per atom (meV/atom) and forces (meV/Å) on training and new test sets for the DP models of six superionic conductors. The test sets consist of configurations sampled from AIMD and MLIP-MD trajectories, respectively.

| Materials | RMSE in energy | | | RMSE in force | | |
|---|---|---|---|---|---|---|
| | Training | Test | | Training | Test | |
| | | AIMD | MLIP-MD | | AIMD | MLIP-MD |
| $Na_{11}Sn_2PS_{12}$ | 0.68 | 1.88 | 2.22 | 48.3 | 45.5 | 48.6 |
| $Na_4P_2S_6$ | 1.13 | 2.74 | 1.85 | 64.1 | 55.1 | 54.4 |
| $Na_2B_{10}H_{10}$ | 1.23 | 0.92 | 0.99 | 81.5 | 56.7 | 70.2 |
| $Na_3OBH_4$(perfect) | 0.47 | 0.19 | 0.27 | 29.4 | 19.2 | 20.7 |
| $Na_3OBH_4$(defect) | — | 0.62 | 0.46 | — | 29.9 | 28.0 |
| $LiBH_4$ | 1.28 | 0.75 | 0.67 | 57.4 | 32.6 | 38.1 |
| $Li_3PS_4$ | 1.35 | 1.34 | 1.20 | 88.5 | 55.5 | 75.2 |

Furthermore, it is important to ensure that the model performs reasonably well across as much of the configuration space as possible. Therefore, parity plots comparing the DFT and DP models are shown in Supplementary Fig.3 and 9 to visualize the error distribution. These plots demonstrate that our MLIPs are reliable and accurately reproduce the DFT energies and forces over a wide range of configurational space.

In general, a good model should yield low numerical error and reproduce key observations. First, the reorientation of these anions in six SEs was well reproduced. For $\alpha$-$Li_3PS_4$, the reorientation frequency obtained by fitting the Ivanov model (Supplementary Table 5) is $0.17\,ns^{-1}$ per rotor at 800 K. This value is of the same order of magnitude as the $\sim 0.5\,ns^{-1}$ per rotor reported by Jun et al. from a quaternion-based algorithm of ab initio molecular dynamics at the same temperature. [5] The comparison is also reasonable considering that their reported value corresponds to rotation angles up to $\sim 100\,^\circ$, whereas the fitted jump amplitude in our Ivanov model is $105\,^\circ$, for which the frequency is expected to be lower.

For the $Na_2B_{10}H_{10}$ high-temperature polymorph, Udovic et al. reported a QENS-derived $B_{10}H_{10}$ reorientational frequency of $\sim 30\,ns^{-1}$. [6] Our value of $50.8\,ns^{-1}$ (Supplementary Table 6) for apical B–B axis

reorientation at 425 K is reasonably consistent with this experimental timescale. For $\gamma$-$Na_4P_2S_6$, Scholz et al. reported reorientation times of 20–100 ps for the P–P bond from ab initio molecular dynamics, corresponding to rates of 10–50 $ns^{-1}$.[7] This range is consistent with our calculated reorientation rate of 15.42 $ns^{-1}$ at the same temperature (Supplementary Table 7). For $Na_{11}Sn_2PS_{12}$, Zhang et al. combined experimental MEM analysis of neutron powder diffraction with AIMD simulations to show facile $PS_4$ reorientation.[8] Our calculated orientation frequencies, ranging from 0.25 to 93.83 $ns^{-1}$ over 600–1050 K (Supplementary Table 9), are therefore consistent with the reported facile anion reorientation in $Na_{11}Sn_2PS_{12}$. For the HT phase of $LiBH_4$, Remhof et al. reported an activation energy of 6.6±0.5 kJ $mol^{-1}$ (0.068±0.005 eV) for $BH_4$ reorientation.[9] This value agrees with our fitted activation energy of 0.0572 eV (Fig.4e). The very low $BH_4$ rotational barrier observed here is consistent with the nearly free $BH_4$ rotation reported by Sun et al. for $Na_3OBH_4$.[10]

The comparison between AIMD and MLMD in terms of the diffusion properties of $Li_3PS_4$, $Na_4P_2S_6$, and $Na_{11}Sn_2PS_{12}$ is shown in Supplementary Fig.1. Both the conductivity over a wide temperature range and the activation energy, calculated using our MLIP, closely match the AIMD results. For $Na_2B_{10}H_{10}$ and $LiBH_4$, conductivity measurements via impedance spectroscopy are available below 423 K[6;11]. The ionic conductivities of $Na_2B_{10}H_{10}$ and $LiBH_4$ are reported to be 0.0259 S $cm^{-1}$ at 423 K and 0.005 S $cm^{-1}$ at 423 K, respectively. These values are in agreement with our extrapolated/interpolated values (Supplementary Fig.2): 0.0206 S $cm^{-1}$ for $Na_2B_{10}H_{10}$ and 0.0028 S $cm^{-1}$ $LiBH_4$.

For $Na_3OBH_4$, the experimental conductivity does not match the result obtained here, likely because the simulation uses a lower Na-vacancy concentration than that inferred from XRD. To further assess the accuracy of the MLIP, we compared its migration barriers for the vacancy-mediated Na-ion pathway in $Na_3OBH_4$ with those from DFT using the nudged elastic band (NEB) method. First, we compared the migration energy barriers obtained from MLIP-NEB and DFT-NEB calculations. Specifically, we first performed an IT-NEB calculation using the MLIP to obtain an initial path, followed by a CI-NEB calculation yielding 8 images including the endpoints. These images were then used as the starting configurations for a DFT CI-NEB calculation in VASP, using the same settings as those employed in the training set generation. As shown in Supplementary Fig.10, the MLIP and DFT energy profiles are in close agreement, with pointwise deviations on the order of 0.02 eV, demonstrating that the model accurately captures the energetics of the Na migration event.

Second, we computed the model deviation[12], i.e., the error indicator $\epsilon$, for each image along the NEB path. As shown in Supplementary Fig.10 (the lower panel), all images fall within the accurate regime, confirming that these configurations are well within the domain of applicability of the current model.

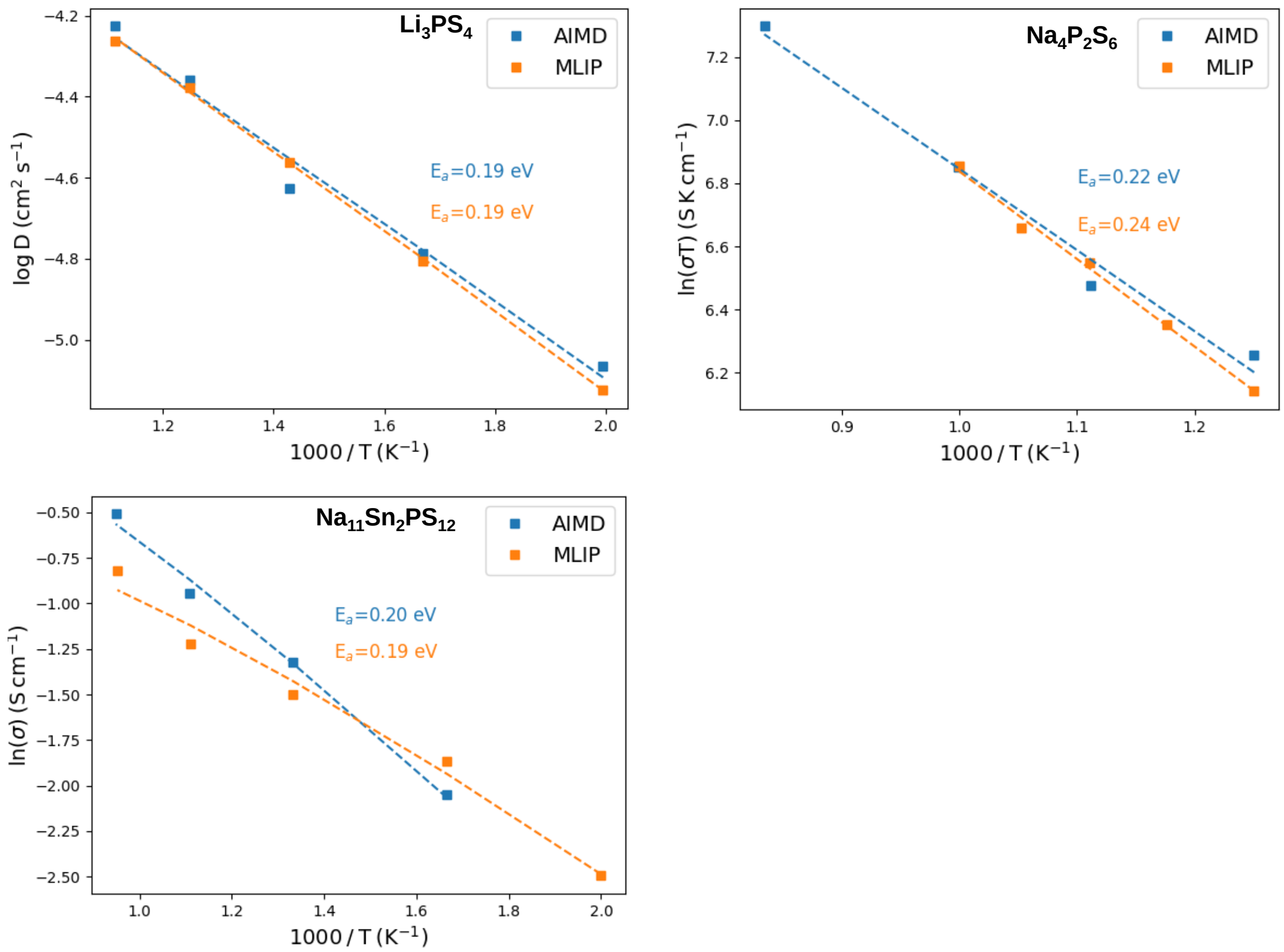


**Supplementary Fig.1** Comparison between AIMD and MLMD results for $Li_3PS_4$[13], $Na_4P_2S_6$[7], and $Na_{11}Sn_2PS_{12}$[14]. Note that we have used the same variable on the y-axis as that in the literature. Our MLMD results closely match those of AIMD.

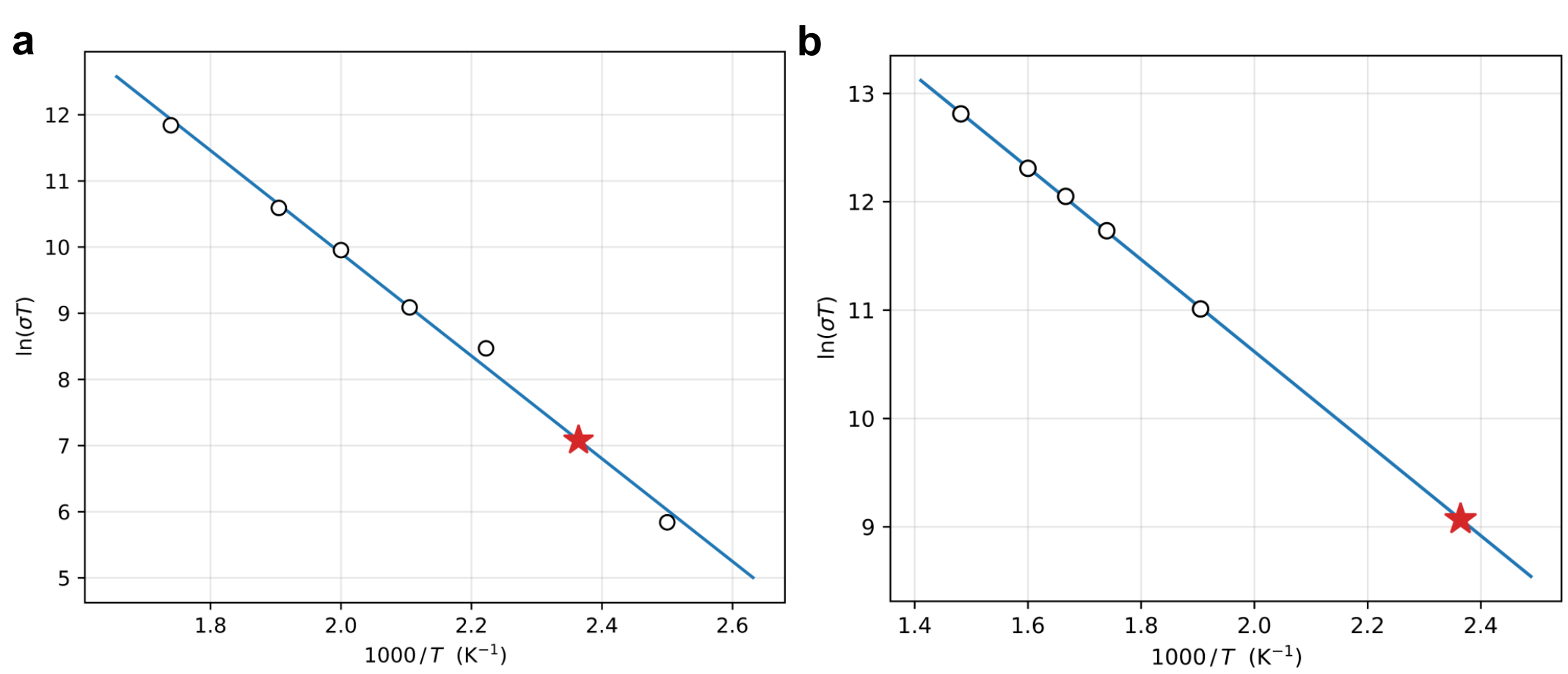


**Supplementary Fig.2** Arrhenius plots of the ionic conductivity computed from the Einstein–Helfand relation, shown as $\ln(\sigma T)$ versus $1000/T$. (a) Interpolation for $LiBH_4$. (b) Extrapolation for $Na_2B_{10}H_{10}$. Open circles denote the values obtained from molecular dynamics simulations, solid lines are linear fits, and red stars mark the inter-/extrapolated conductivities at the target temperature.

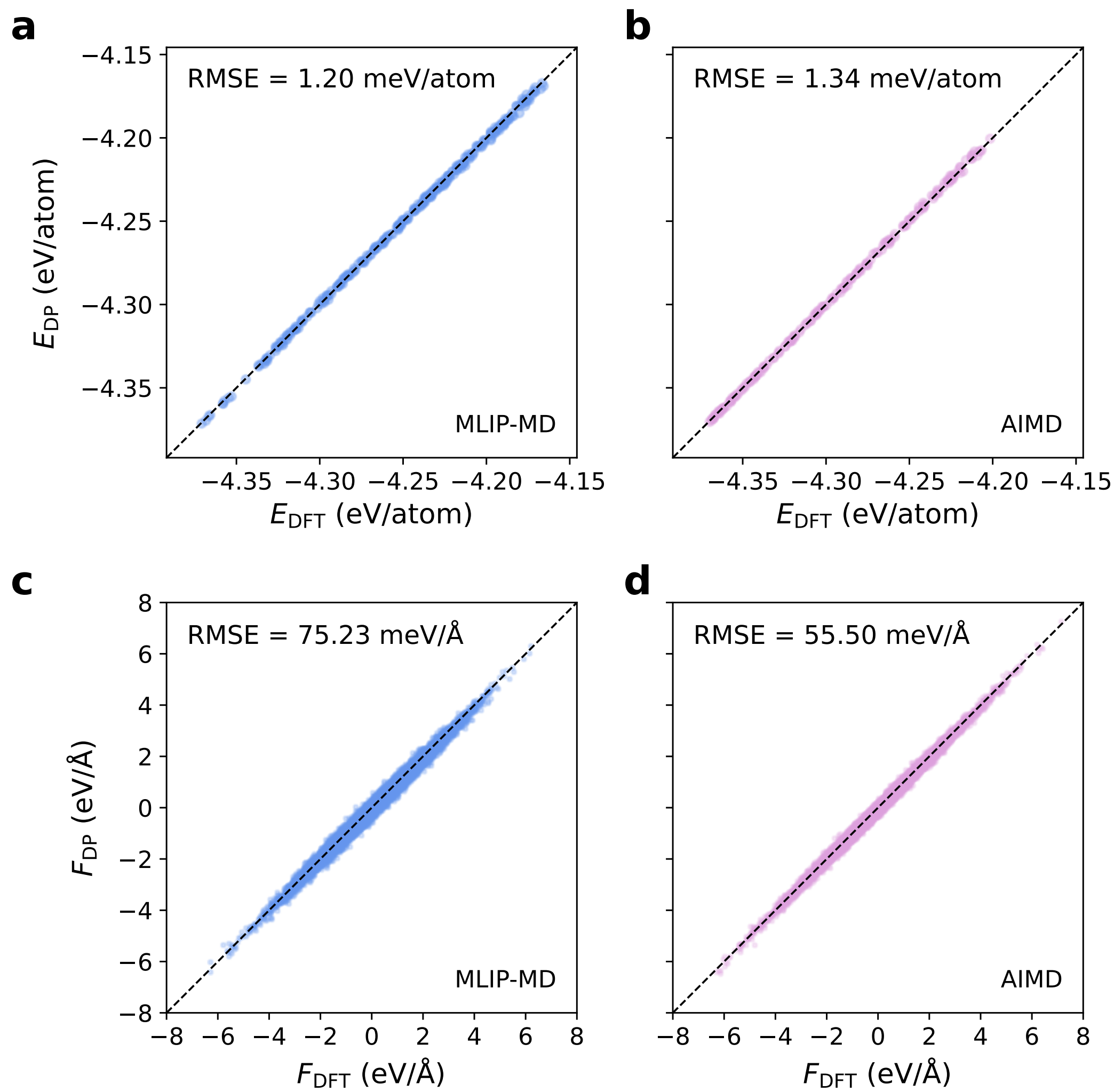


**Supplementary Fig.3** Parity plots of the DP model against DFT for $Li_3PS_4$ on the test set. (a, b) Per-atom energies and (c, d) atomic force components. The configurations are sampled either from AIMD or MLIP-MD.

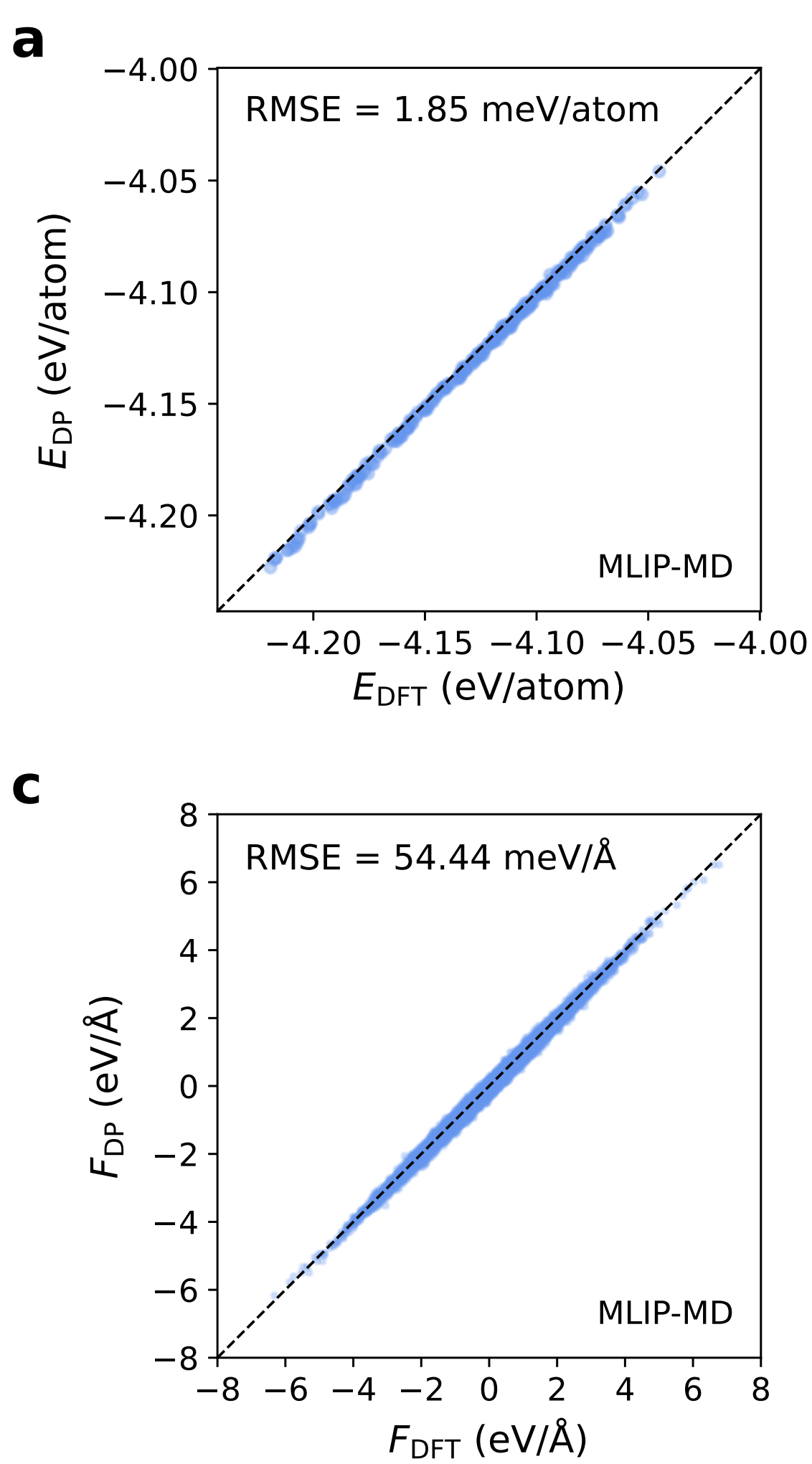


**Supplementary Fig.4** Parity plots of the DP model against DFT for $Na_4P_2S_6$ on the test set. (a, b) Per-atom energies and (c, d) atomic force components. The configurations are sampled either from AIMD or MLIP-MD.

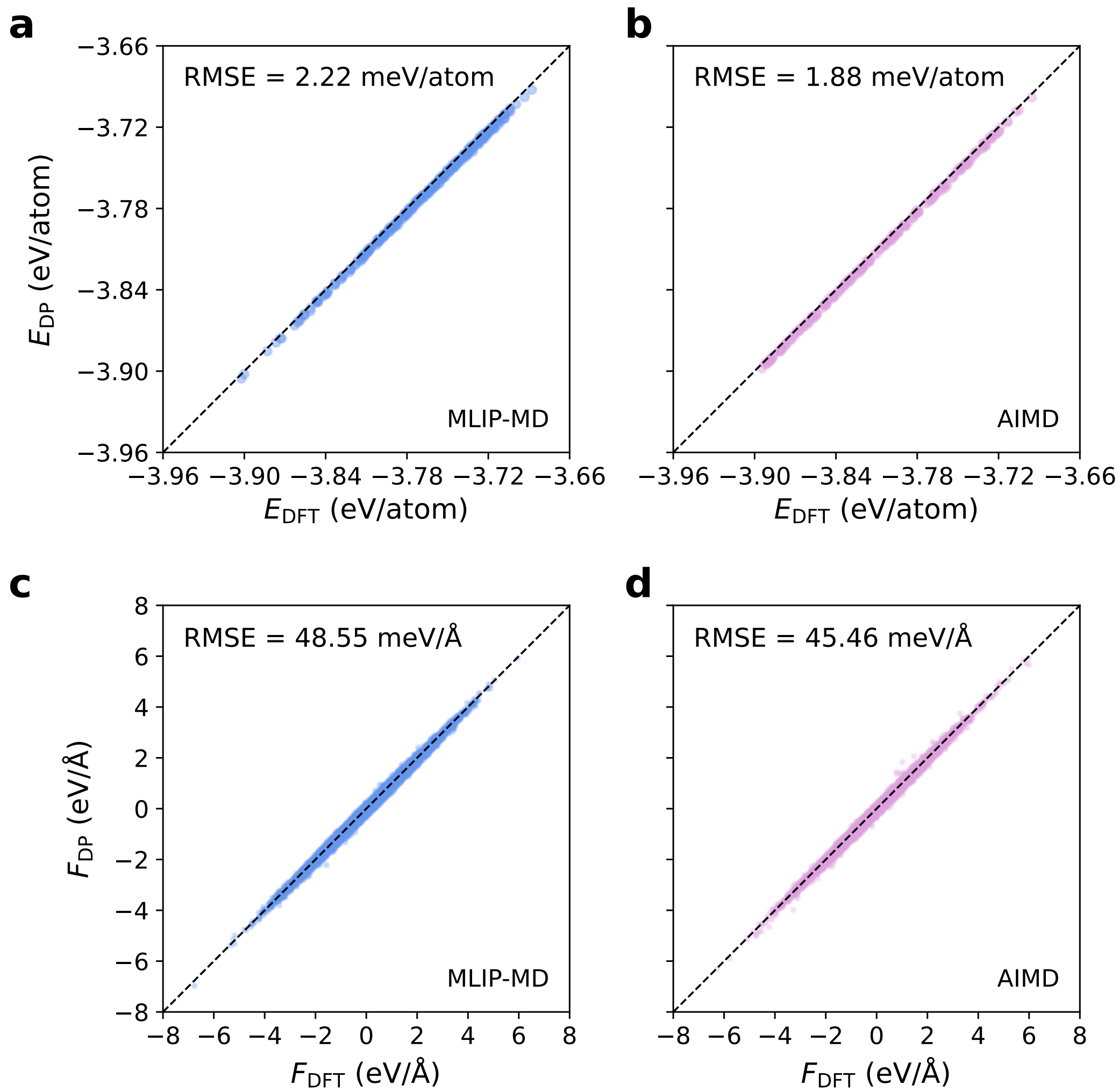


**Supplementary Fig.5** Parity plots of the DP model against DFT for $Na_{11}Sn_2PS_{12}$ on the test set. (a, b) Per-atom energies and (c, d) atomic force components. The configurations are sampled either from AIMD or MLIP-MD.

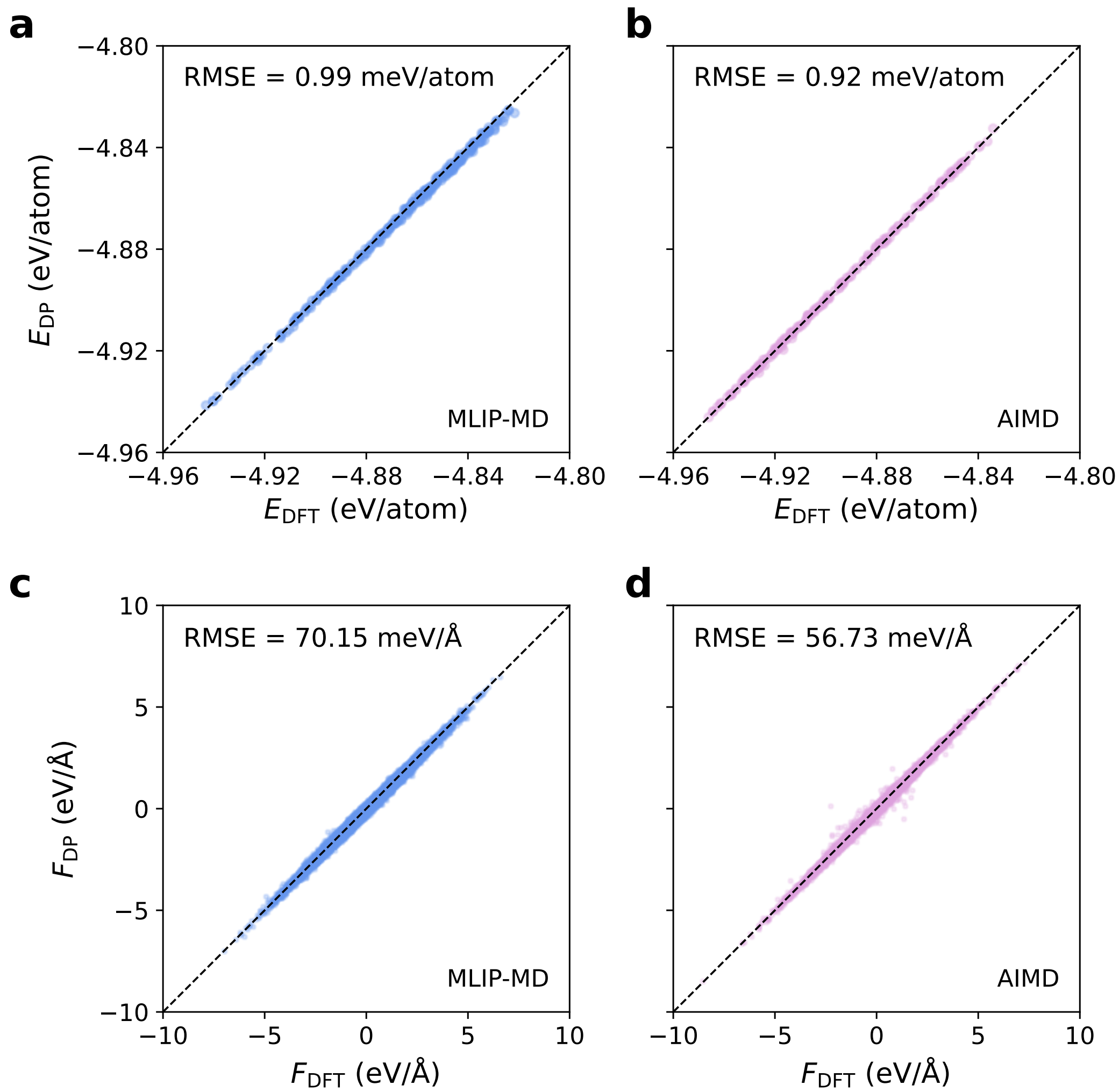


**Supplementary Fig.6** Parity plots of the DP model against DFT for $Na_2B_{10}H_{10}$ on the test set. (a, b) Per-atom energies and (c, d) atomic force components. The configurations are sampled either from AIMD or MLIP-MD.

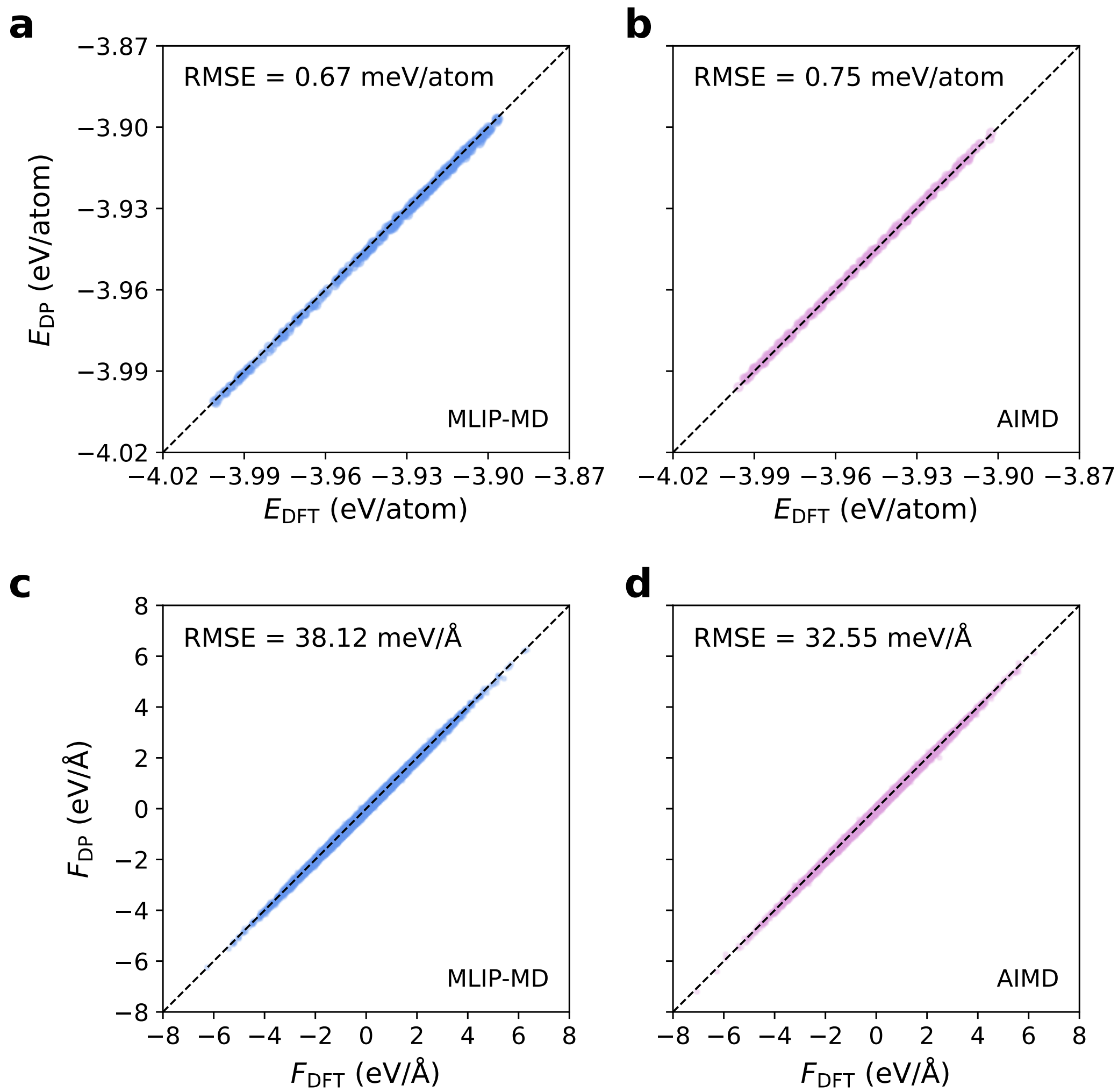


**Supplementary Fig.7** Parity plots of the DP model against DFT for $LiBH_4$ on the test set. (a, b) Per-atom energies and (c, d) atomic force components. The configurations are sampled either from AIMD or MLIP-MD.

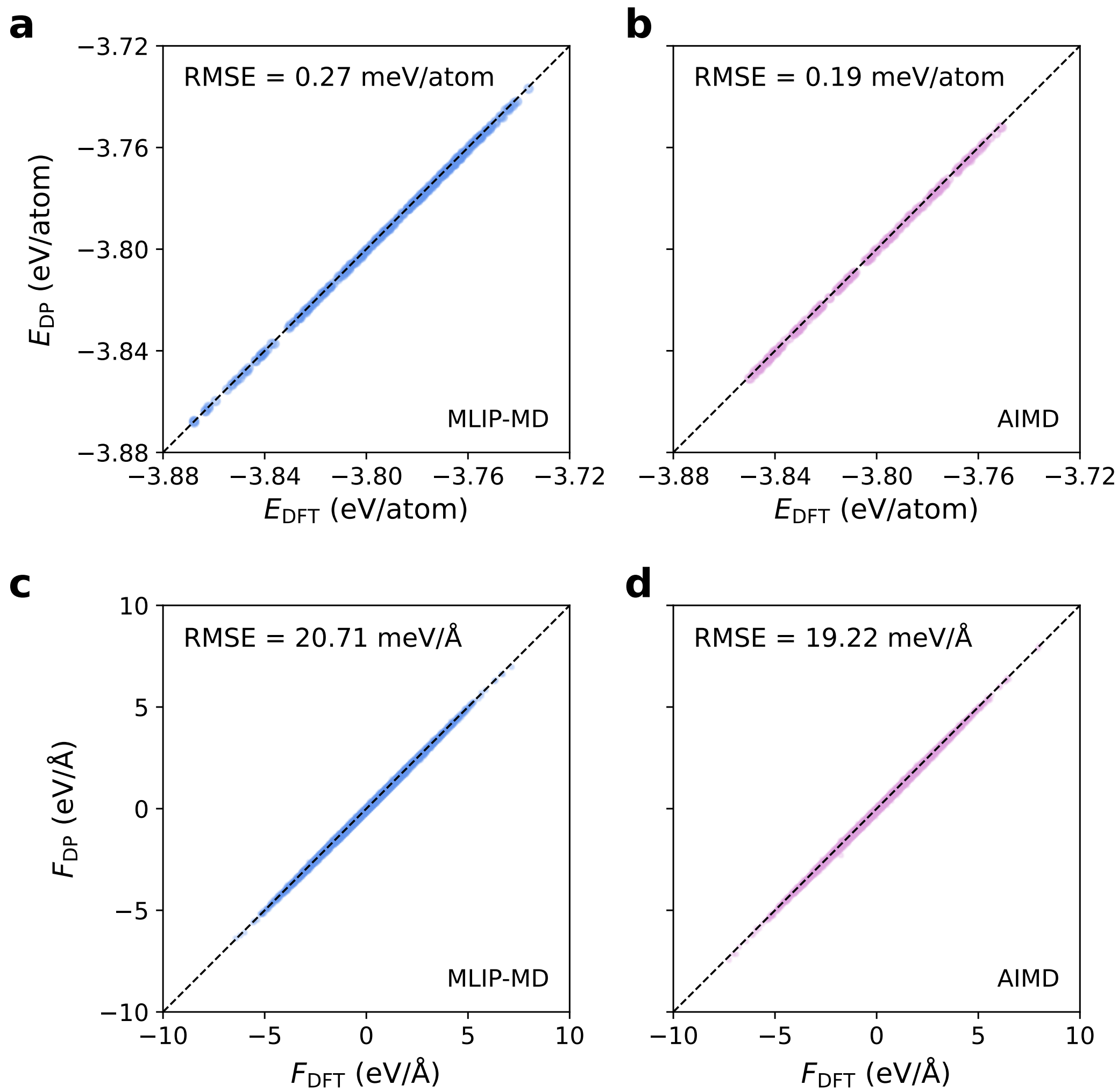


**Supplementary Fig.8** Parity plots of the DP model against DFT for host $Na_3OBH_4$ on the test set. (a, b) Per-atom energies and (c, d) atomic force components. The configurations are sampled either from AIMD or MLIP-MD.

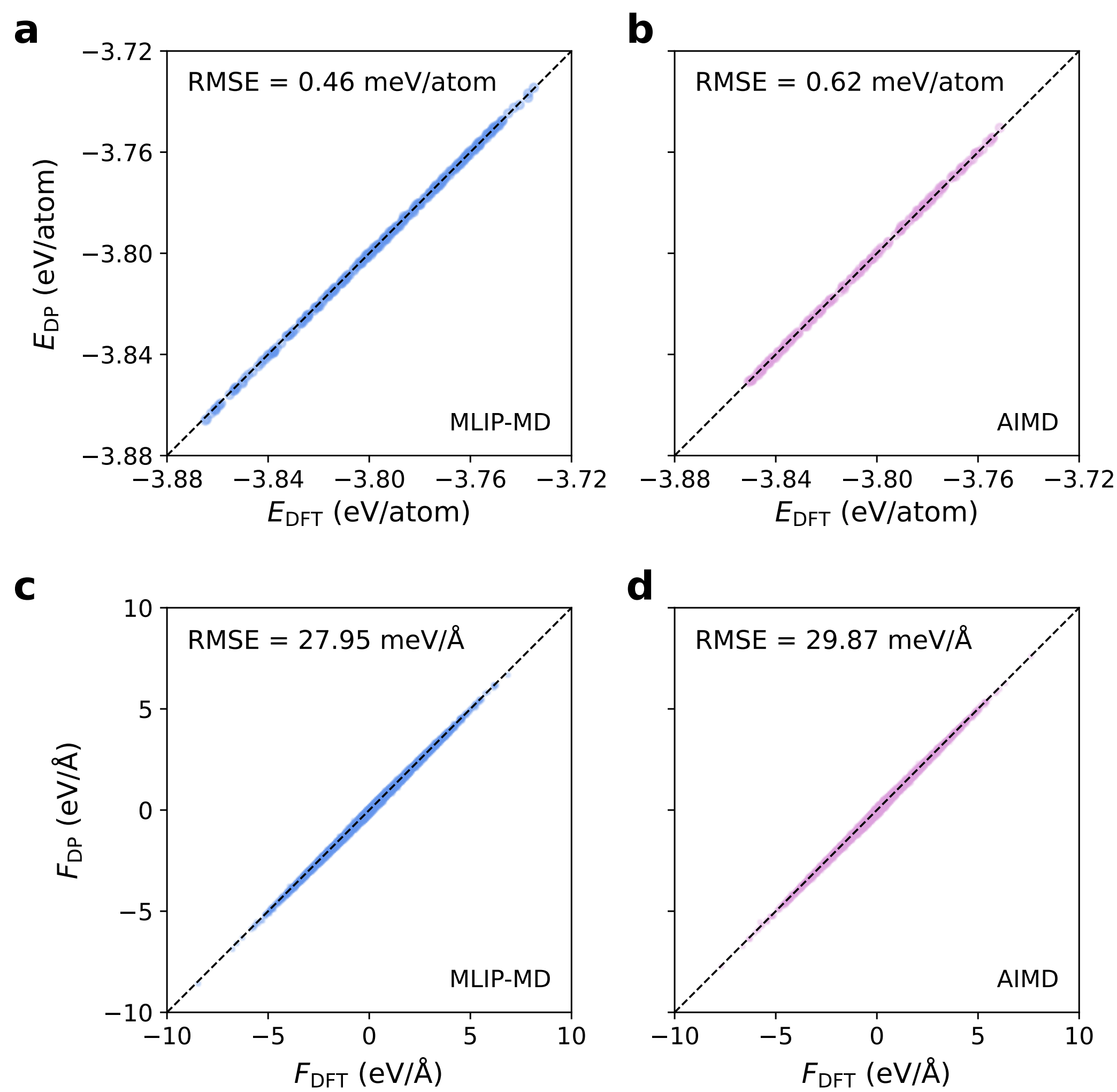


**Supplementary Fig.9** Parity plots of the DP model against DFT for $Na_3OBH_4$ containing one sodium vacancy on the test set. (a, b) Per-atom energies and (c, d) atomic force components. The configurations are sampled either from AIMD or MLIP-MD.

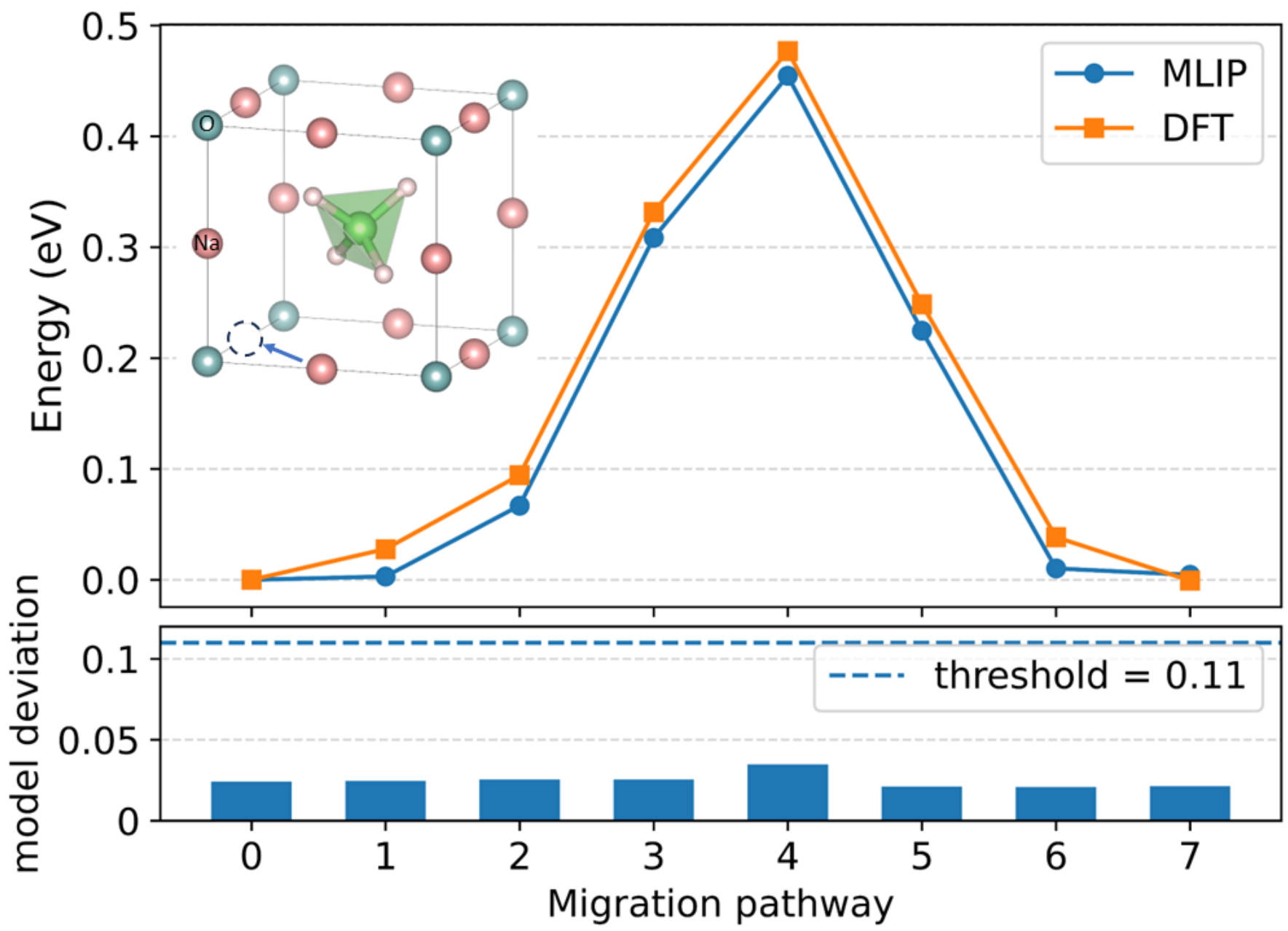


**Supplementary Fig.10** MLIP and DFT comparison for Na migration in $3 \times 3 \times 3$ $Na_3OBH_4$ supercell containing a Na vacancy. (Top) CI-NEB energy profiles computed with MLIP (blue) and DFT (orange) along the migration pathway, with 8 images including the endpoints. The migration barriers are 0.46 eV (MLIP) and 0.48 eV (DFT), with pointwise deviations on the order of 0.02 eV. The inset shows the migration pathway, where the dashed circle indicates the Na vacancy site and the arrow denotes the direction of Na migration. (Bottom) Model deviation for each image along the NEB path. All values fall well below the DP-GEN convergence threshold of 0.11 eV/Å, confirming that these configurations lie within the domain of applicability of the current MLIP.

## Supplementary Note 2: Defect physics of $Na_3OBH_4$

**Supplementary Table 3** Vertices of the phase stability region. Deviations of elemental chemical potentials ($\Delta\mu_i$) are reported in eV.

| Vertex | $\Delta\mu_{Na}$ (eV) | $\Delta\mu_O$ (eV) | $\Delta\mu_B$ (eV) | $\Delta\mu_H$ (eV) | Phases in equilibrium with $Na_3OBH_4$ |
|---|---|---|---|---|---|
| V1 | -0.511 | -4.456 | -1.748 | -0.025 | NaOH, NaH, $NaBH_4$ |
| V2 | -0.514 | -4.449 | -1.731 | -0.028 | NaOH, $Na_3BO_3$, $NaBH_4$ |
| V3 | -0.504 | -4.456 | -1.741 | -0.031 | NaOH, $Na_3BO_3$, NaH |
| V4 | -0.036 | -5.405 | -0.298 | -0.506 | $Na(BH)_6$, $Na_3BO_3$, $NaBH_4$ |
| V5 | -0.027 | -5.424 | -0.297 | -0.509 | $Na(BH)_6$, NaH, $NaBH_4$ |
| V6 | -0.021 | -5.423 | -0.291 | -0.515 | $Na(BH)_6$, $Na_3BO_3$, NaH |

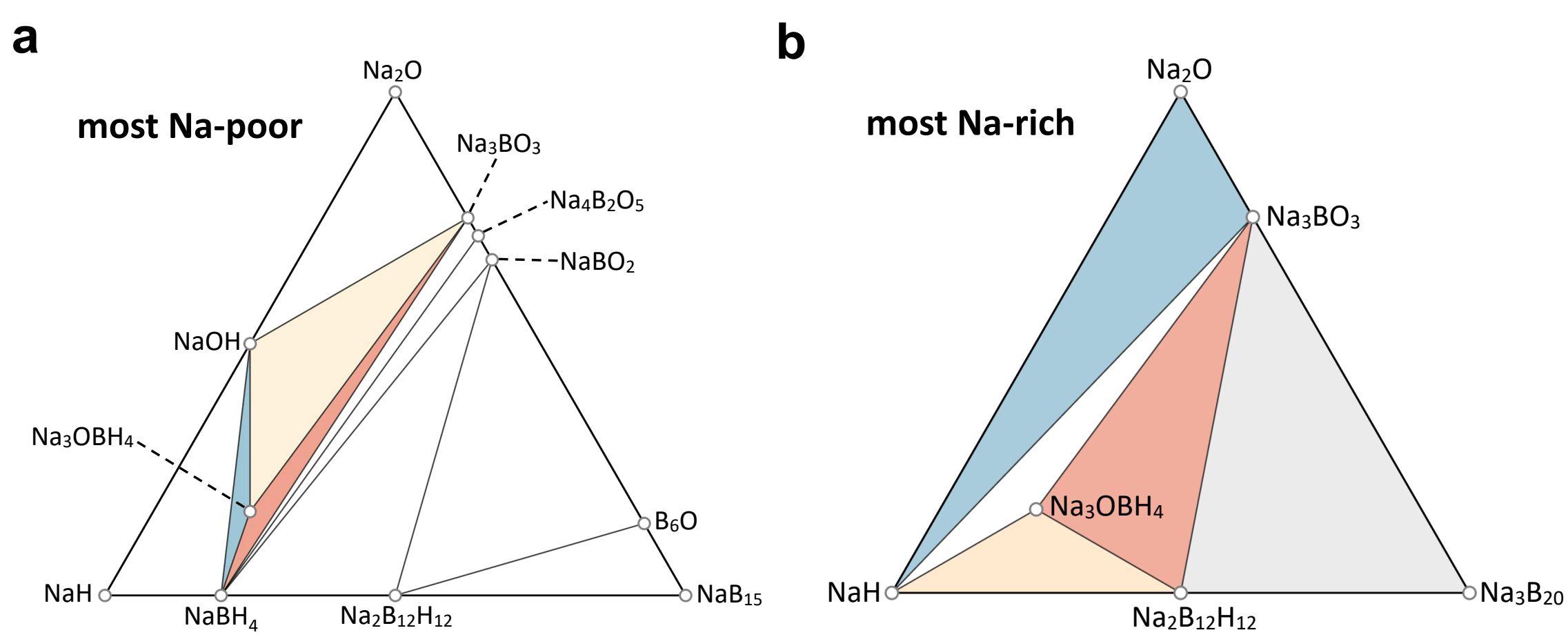


**Supplementary Fig.11** Projected phase diagram of $Na_3OBH_4$ for (a) most Na-poor and (b) most Na-rich conditions.

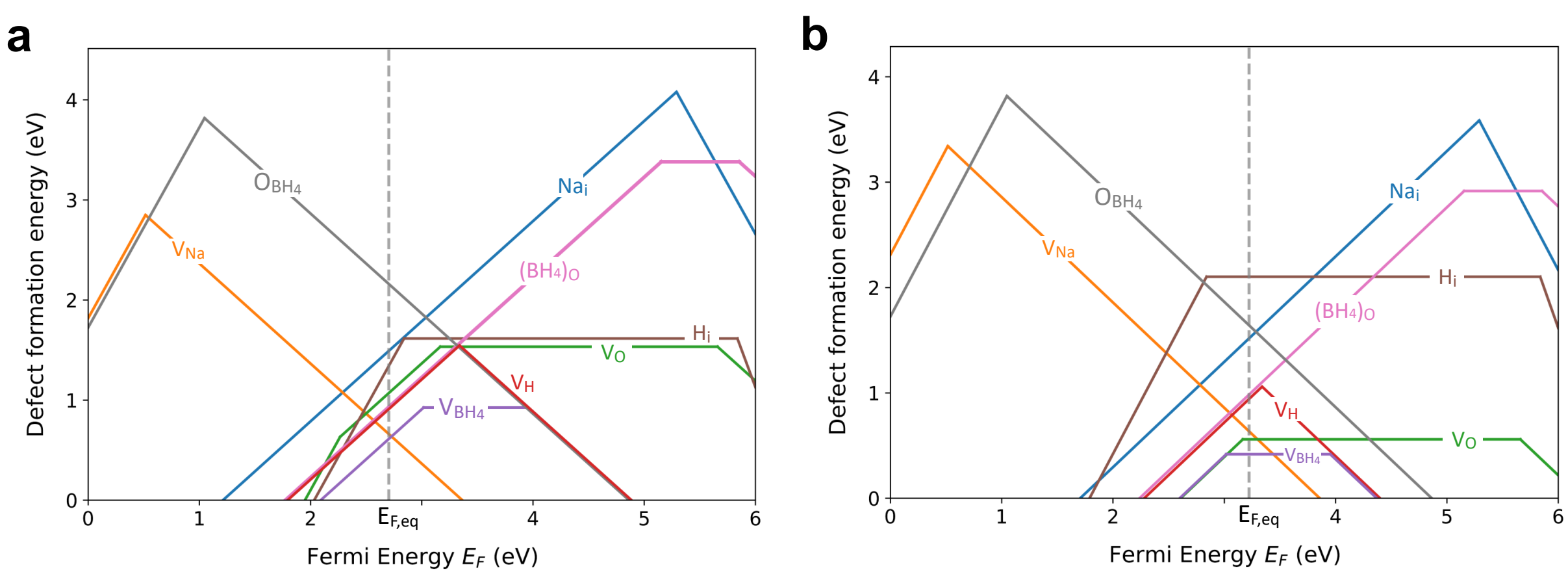


**Supplementary Fig.12** Formation energy ($\Delta E_{D,q}$) of native defects as a function of Fermi energy ($E_F$) in $Na_3OBH_4$ under (a) most Na-poor and (b) most Na-rich conditions. The equilibrium $E_{F,eq}$ is calculated at the synthesis temperature of 523 K and marked by vertical dashed lines.

**Supplementary Table 4** Chemical formula, Material ID in Materials Project and space group numbers of competing phases considered in calculating the phase diagram of $Na_3OBH_4$.

| Compound | Material ID (Materials Project) | Space group |
|---|---|---|
| $B(HO)_3$ | mp-23791 | $P\bar{1}$ |
| $B(HO)_3$ | mp-759069 | $P3_2$ |
| $B_2O_3$ | mp-306 | $P3_121$ |
| $B_6O$ | mp-1346 | $R\bar{3}m$ |
| $BHO_2$ | mp-1198592 | $P2_1/c$ |
| $BHO_2$ | mp-696403 | $Pnma$ |
| $BHO_2$ | mp-721851 | $P\bar{4}3n$ |
| $Na(BH)_6$ | mp-978278 | $P2_1/c$ |
| $Na_{12}B_{26}O_{45}$ | mp-1180710 | $Iba2$ |
| $Na_2B_3HO_6$ | mp-706631 | $P2_1/c$ |
| $Na_2B_4H_8O_{11}$ | mp-706655 | $P2_1/c$ |
| $Na_2B_4O_7$ | mp-17941 | $P\bar{1}$ |
| $Na_2B_4O_7$ | mp-557423 | $P\bar{1}$ |
| $Na_2B_5H_3O_{10}$ | mp-720312 | $P2_1/c$ |
| $Na_2B_8O_{13}$ | mp-542300 | $P2_1/c$ |
| $Na_2BHO_3$ | mp-556125 | $Pnma$ |
| $Na_2H_{16}O_9$ | mp-505185 | $P2_1/c$ |
| $Na_2O_2$ | mp-2340 | $P\bar{6}2m$ |
| $Na_2O$ | mp-2352 | $Fm\bar{3}m$ |
| $Na_3B_{20}$ | mp-262 | $Cmmm$ |
| $Na_3B_3(HO_2)_4$ | mp-1203588 | $Cc$ |
| $Na_3B_3H_2O_7$ | mp-720583 | $Pnma$ |
| $Na_3B_5(HO_5)_2$ | mp-722369 | $Pca2_1$ |
| $Na_3B_5H_4O_{11}$ | mp-720296 | $Pbca$ |
| $Na_3B_7O_{12}$ | mp-31073 | $P\bar{1}$ |
| $Na_3BO_3$ | mp-30975 | $P2_1/c$ |
| $Na_4B_2O_5$ | mp-27564 | $C2/c$ |
| $NaB(HO)_4$ | mp-707419 | $P2_1/c$ |
| $NaB_{15}$ | mp-2315 | $Imma$ |
| $NaB_2H_5O_6$ | mp-706629 | $R32$ |
| $NaB_3H_4O_7$ | mp-720642 | $C2/c$ |
| $NaB_3O_5$ | mp-556226 | $P2_1/c$ |
| $NaB_3O_5$ | mp-557406 | $P2_1/c$ |
| $NaB_5(H_2O_5)_2$ | mp-721294 | $P2_1/c$ |
| $NaB_5H_{10}O_{13}$ | mp-540721 | $C2/c$ |
| $NaBH_8O_7$ | mp-721727 | $P\bar{1}$ |
| $NaBO_2$ | mp-3889 | $R\bar{3}c$ |
| $NaH_3O_2$ | mp-24193 | $Pbca$ |
| $NaH_8O_5$ | mp-733637 | $C2/c$ |
| NaHO | mp-23891 | $Cmcm$ |
| NaHO | mp-23940 | $P2_1/m$ |
| NaHO | mp-626778 | $Pbcm$ |
| $NaO_2$ | mp-1901 | $Pnnm$ |

## Supplementary Note 3: Rotation Dynamics Statistics

The rotor can adopt different orientations in some SEs; for example, $PS_4$(I) and $PS_4$(II) in $\alpha-Li_3PS_4$ (Fig.1d). Accordingly, to obtain meaningful rotational statistics, we compute $\theta$ and $\phi$ by tracking the same individual bond in rotors sharing the same orientation. The resulting angular distributions for all six SEs are shown in Supplementary Fig.13–21. All distributions exhibit multiple maxima, implying that the rotors undergo rotational motion between preferred orientations. For $\alpha$-$Li_3PS_4$, $PS_4$(I) and $PS_4$(II) each exhibit four maxima at distinct angular positions from 700 to 900 K (Supplementary Fig.13 and 14); $Na_{11}Sn_2PS_{12}$ shows a similar behaviour at 600 K (Supplementary Fig.15 and 16). However, at 900 K, the distributions of $PS_4$(I) and $PS_4$(II) show eight maxima, which is the combination of the two four-peak patterns observed at 600 K for $PS_4$(I) and $PS_4$(II). This increase in accessible orientational states raises the orientational entropy and may further stabilize the plastic phase[15]. For $Na_3OBH_4$ and $Na_2B_{10}H_{10}$, the angular distributions are consistent with the cubic symmetry of the crystals (Supplementary Fig.19 and 20). For $LiBH_4$, $BH_4$(I) and $BH_4$(II) show different distributions, which are also consistent with the hexagonal symmetry of the crystal (Supplementary Fig.21).

**Supplementary Table 5** Reorientation frequency $\nu$ and jump amplitude $\Delta$ for $PS_4$ in $Li_3PS_4$, obtained by fitting the Ivanov model.

| | 700 K | 750 K | 800 K | 850 K | 900 K |
|---|---|---|---|---|---|
| $\nu$ ($ns^{-1}$) | 0.03 | 0.07 | 0.17 | 0.4 | 0.9 |
| $\Delta$ (°) | 102.2 | 105.3 | 105.2 | 104.9 | 104.3 |

**Supplementary Table 6** Reorientation frequency $\nu$ and jump amplitude $\Delta$ for apical B–B axis of $B_{10}H_{10}$ in $Na_2B_{10}H_{10}$, obtained by fitting the Ivanov model.

| | 425 K | 475 K | 525 K | 575 K |
|---|---|---|---|---|
| $\nu$ ($ns^{-1}$) | 50.78 | 73.70 | 101.54 | 149.72 |
| $\Delta$ (°) | 65.4 | 63.0 | 63.1 | 57.2 |

**Supplementary Table 7** Reorientation frequency $\nu$ and jump amplitude $\Delta$ for P–P bond of $P_2S_6$ in $\gamma$-$Na_4P_2S_6$, obtained by fitting the Ivanov model.

| | 700 K | 800 K | 850 K | 900 K | 950 K | 1000 K |
|---|---|---|---|---|---|---|
| $\nu$ ($ns^{-1}$) | 0.58 | 2.41 | 4.18 | 6.89 | 10.86 | 15.42 |
| $\Delta$ (°) | 71.4 | 68.6 | 66.2 | 64.5 | 63.3 | 62.0 |

**Supplementary Table 8** Frequency $\nu$ and jump amplitude $\Delta$ for the internal rotation of $P_2S_6$ in $\gamma$-$Na_4P_2S_6$, obtained by fitting the Ivanov model.

| | 700 K | 800 K | 850 K | 900 K | 950 K | 1000 K |
|---|---|---|---|---|---|---|
| $\nu$ ($ns^{-1}$) | 0.08 | 0.32 | 0.61 | 1.04 | 1.77 | 2.8 |
| $\Delta$ (°) | 105.67 | 105.0 | 104.5 | 103.4 | 103.1 | 102.5 |

**Supplementary Table 9** Reorientation frequency $\nu$ and jump amplitude $\Delta$ for $Na_{11}Sn_2PS_{12}$, obtained by fitting the Ivanov model.

| | 600 K | 675 K | 750 K | 825 K | 900 K | 1050 K |
|---|---|---|---|---|---|---|
| $\nu$ ($ns^{-1}$) | 0.25 | 0.81 | 2.57 | 6.99 | 20.21 | 93.83 |
| $\Delta$ (°) | 106.7 | 106.0 | 103.8 | 100.1 | 91.3 | 74.1 |

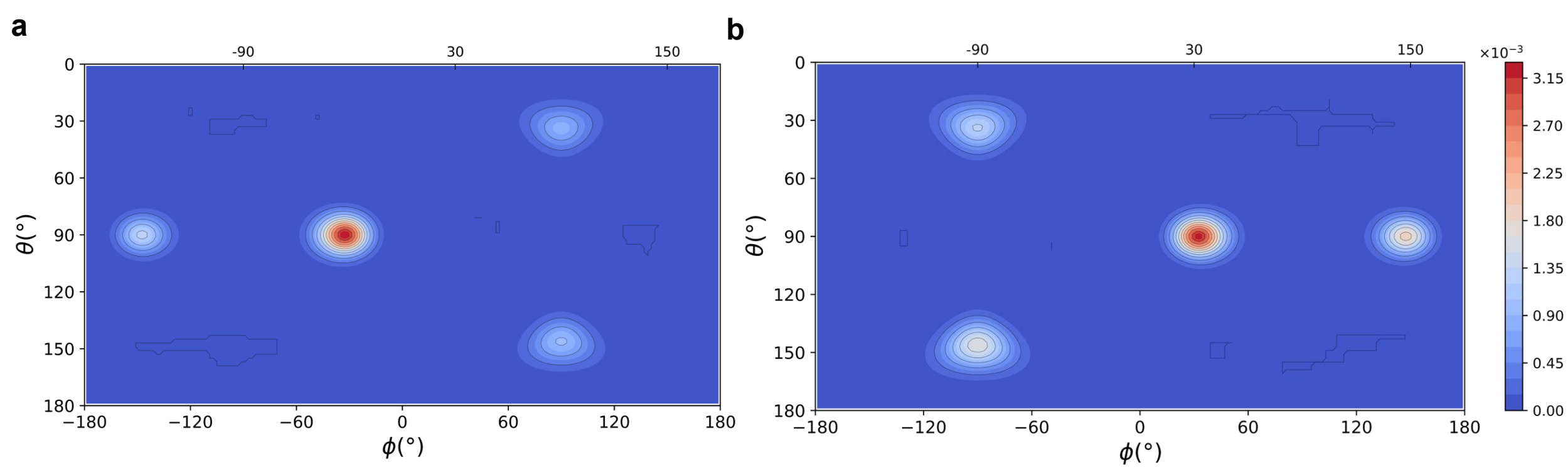

**Supplementary Fig.13** Angular distribution of one P-S bond (the same bond tracked across all $PS_4$ units) for (a) $PS_4$(I) and (b) $PS_4$(II) in $Li_3PS_4$ at 700 K.

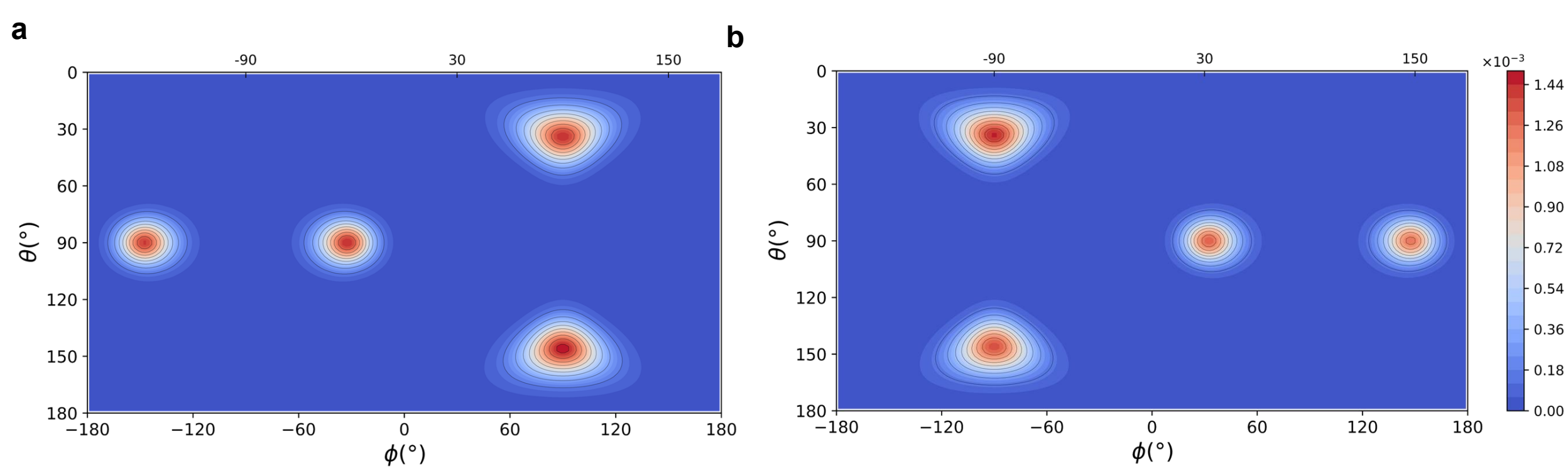

**Supplementary Fig.14** Angular distribution of one P-S bond (the same bond tracked across all $PS_4$ units) for (a) $PS_4$(I) and (b) $PS_4$(II) in $Li_3PS_4$ at 900 K.

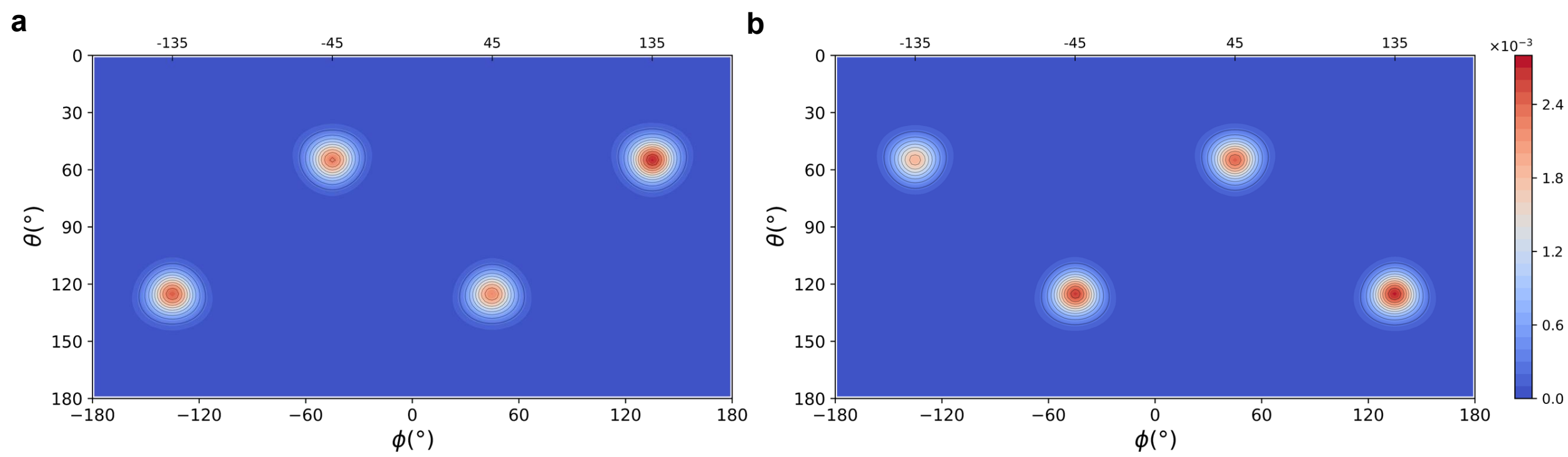


**Supplementary Fig.15** Angular distribution of one P-S bond (the same bond tracked across all $PS_4$ units) for (a) $PS_4$(I) and (b) $PS_4$(II) in $Na_{11}Sn_2PS_{12}$ at 600 K.

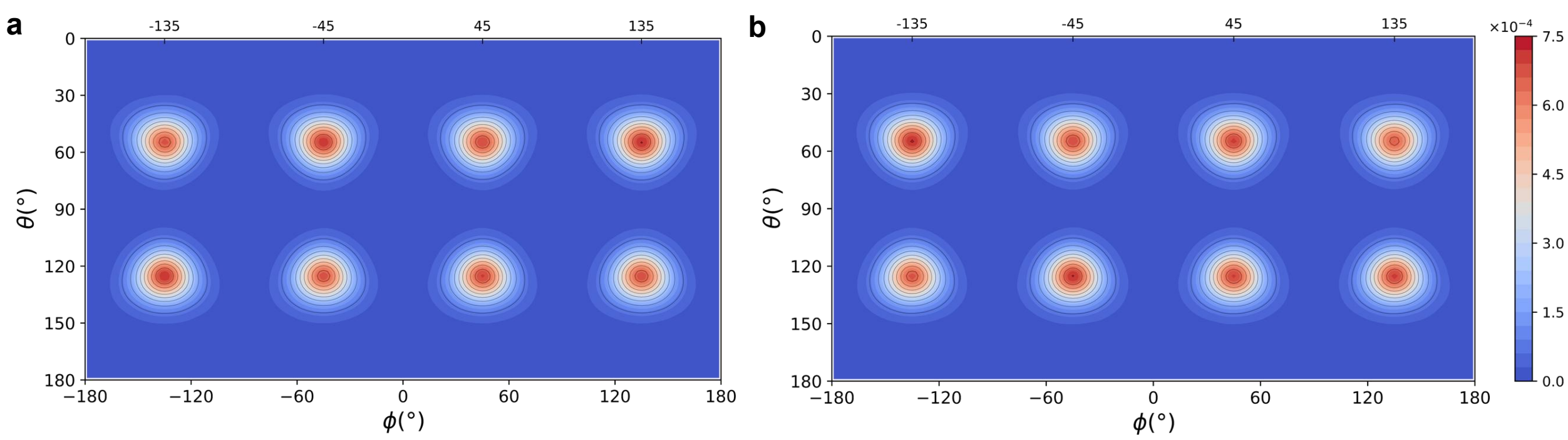


**Supplementary Fig.16** Angular distribution of one P-S bond (the same bond tracked across all $PS_4$ units) for (a) $PS_4$(I) and (b) $PS_4$(II) in $Na_{11}Sn_2PS_{12}$ at 900 K.

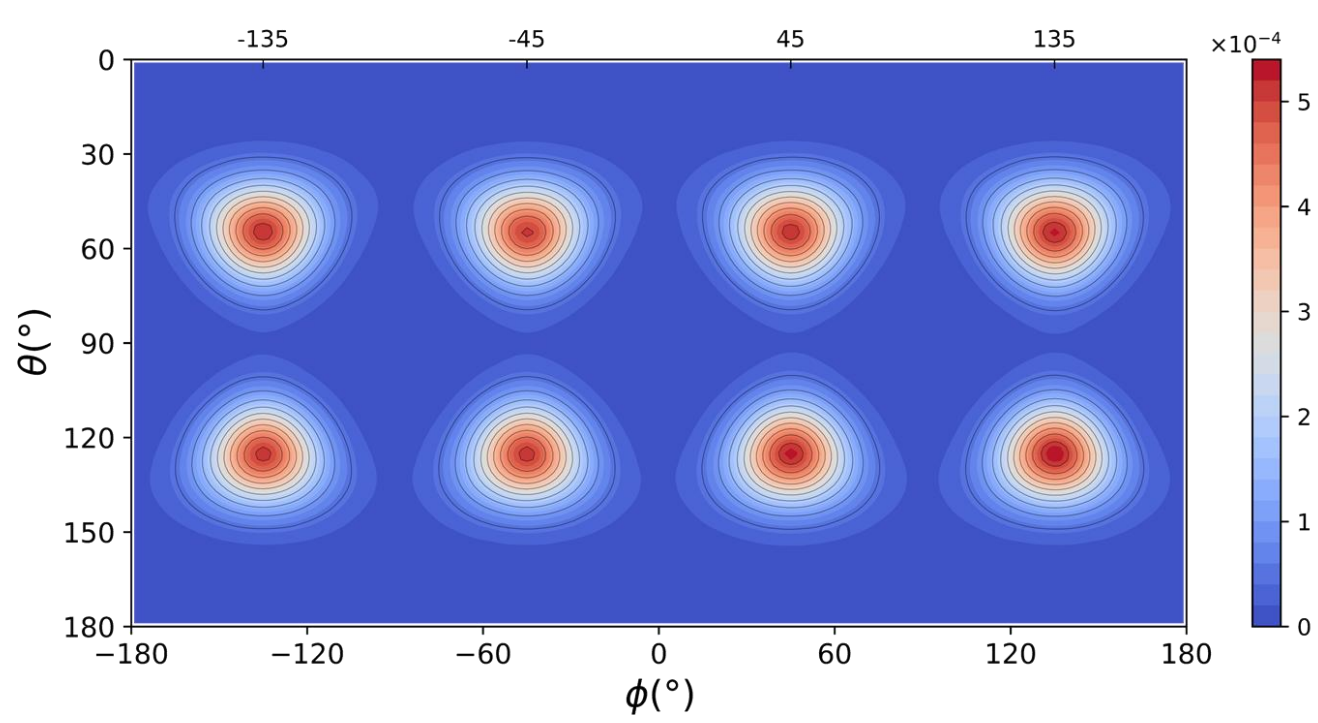


**Supplementary Fig.17** Angular distribution of one P-P bond for $P_2S_6$ in $Na_4P_2S_6$ at 1000 K.

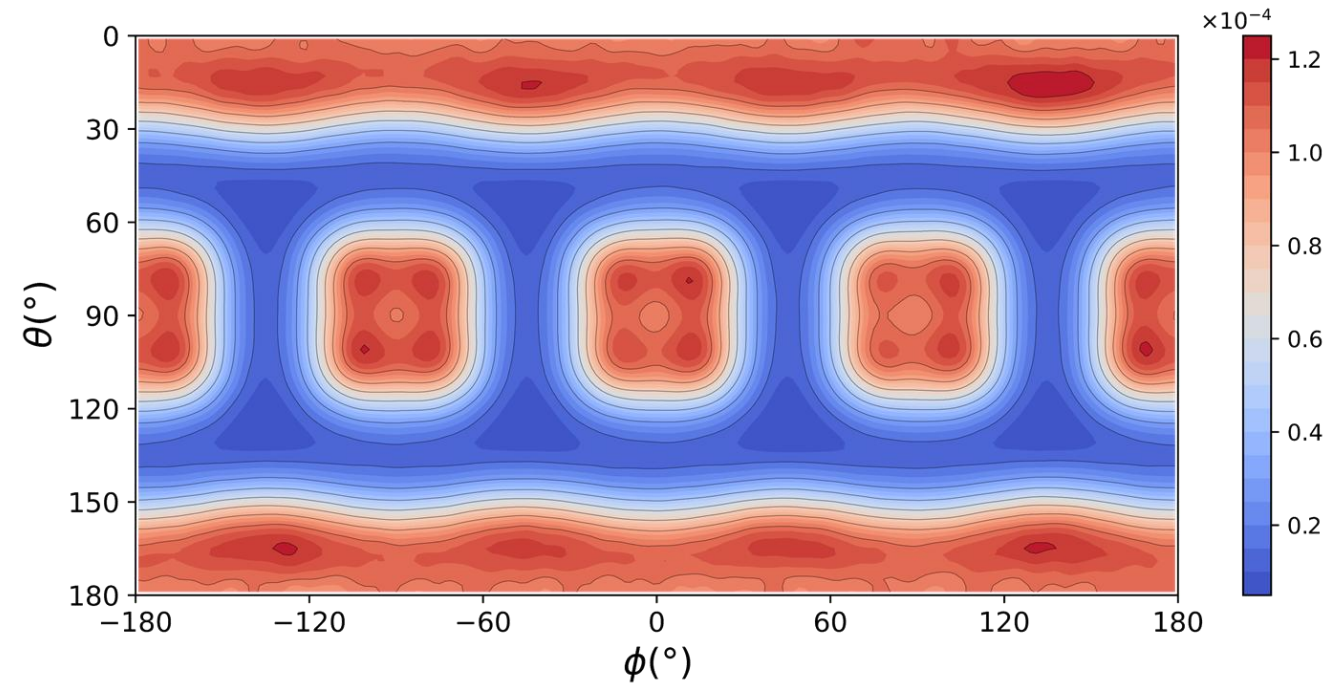


**Supplementary Fig.18** Angular distribution of one P-S bond (the same bond tracked across all $P_2S_6$ units) for $P_2S_6$ in $Na_4P_2S_6$ at 1000 K.

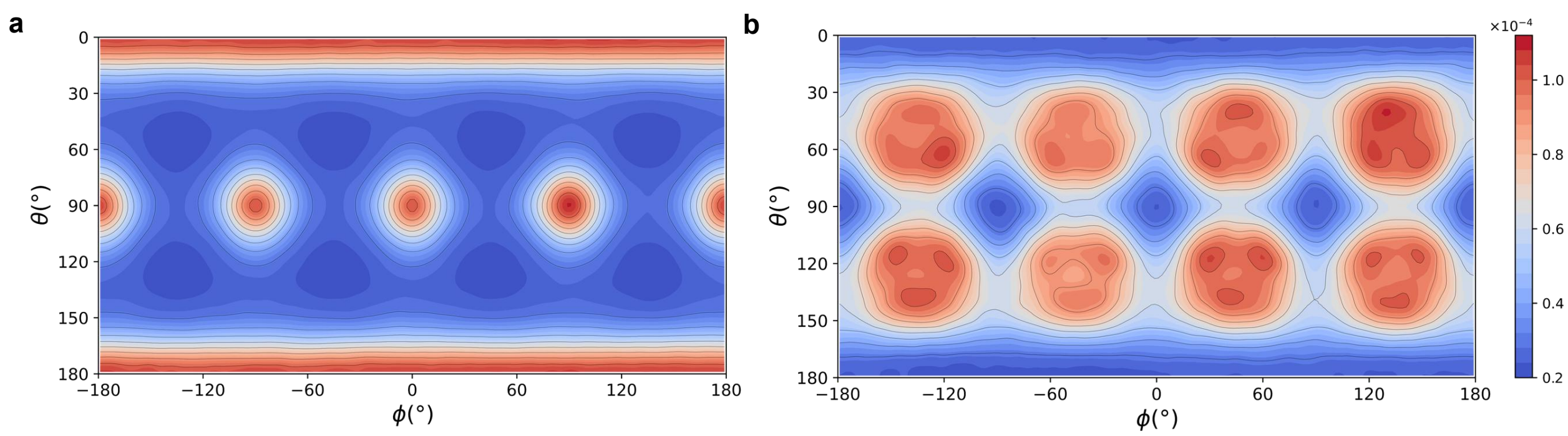


**Supplementary Fig.19** Angular distribution of for vector formed by (a) center of mass and apex B and (b) center of mass and non-apex B in $Na_2B_{10}H_{10}$ at 575 K.

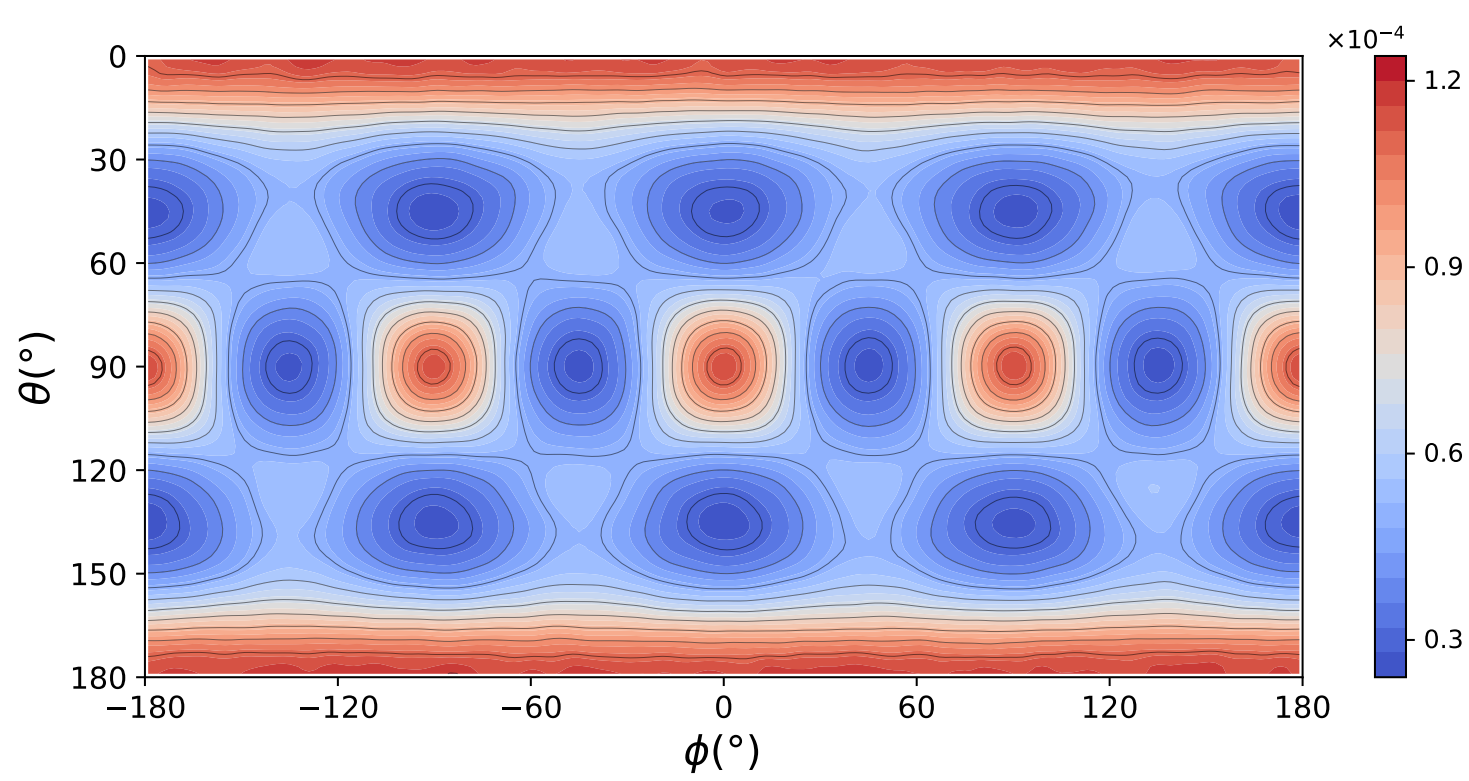


**Supplementary Fig.20** Angular distribution of B-H bond of $BH_4$ in $Na_3OBH_4$ at 600 K.

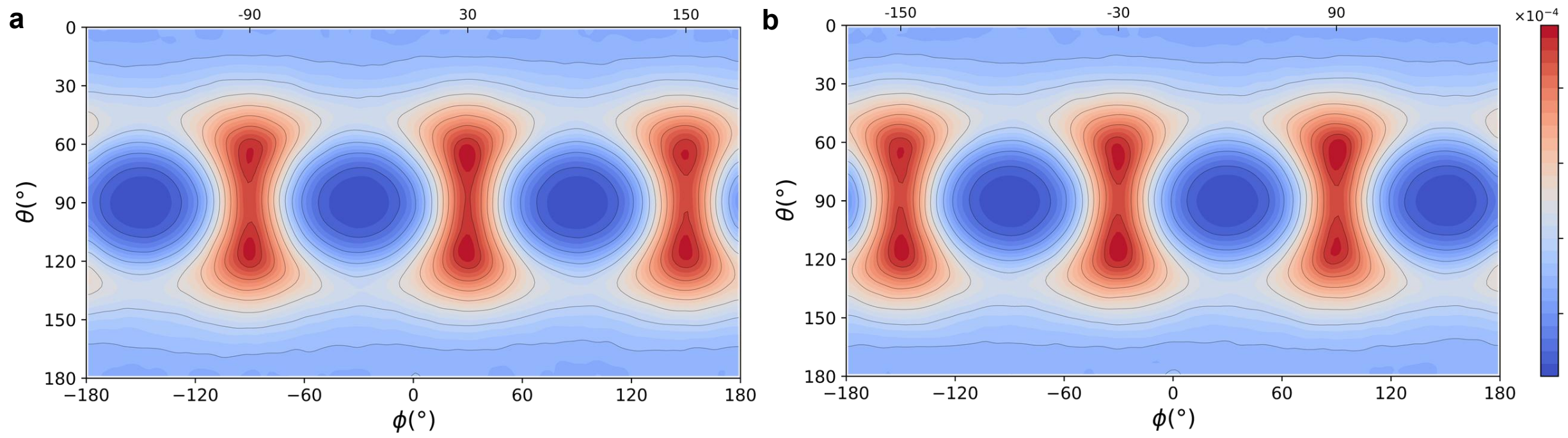


**Supplementary Fig.21** Angular distribution of B-H bond (the same bond tracked across all $BH_4$ units) for (a) $BH_4$(I) and (b) $BH_4$(II) in $LiBH_4$ at 400 K.

## Supplementary Note 4: Constrained Algorithm

### Separation of motion mode

According to normal mode theory, translational and rotational degress of freedom (DOF) are orthogonal to vibrational modes, which provides a theoretical basis for applying constraint methods on special DOF. Our constraint algorithm is similar to one used in biomolecular simulations to control the overall rotation of large molecules[16]. Additionally, the partial density of states of rotor anion in Supplementary Fig.22. We find significant suppression of low-frequency peaks with rotation constraint applied and complete disappearance of low-frequency peaks with roto-translational constraints applied. In constrast, all high-frequency vibrational peaks persisted. This precisely demonstrates that our constraint algorithm can independently and effectively constrain each motion mode. The polar angle ($\theta$) evolution with rotational constraint and without constraint is shown in Supplementary Fig.23.

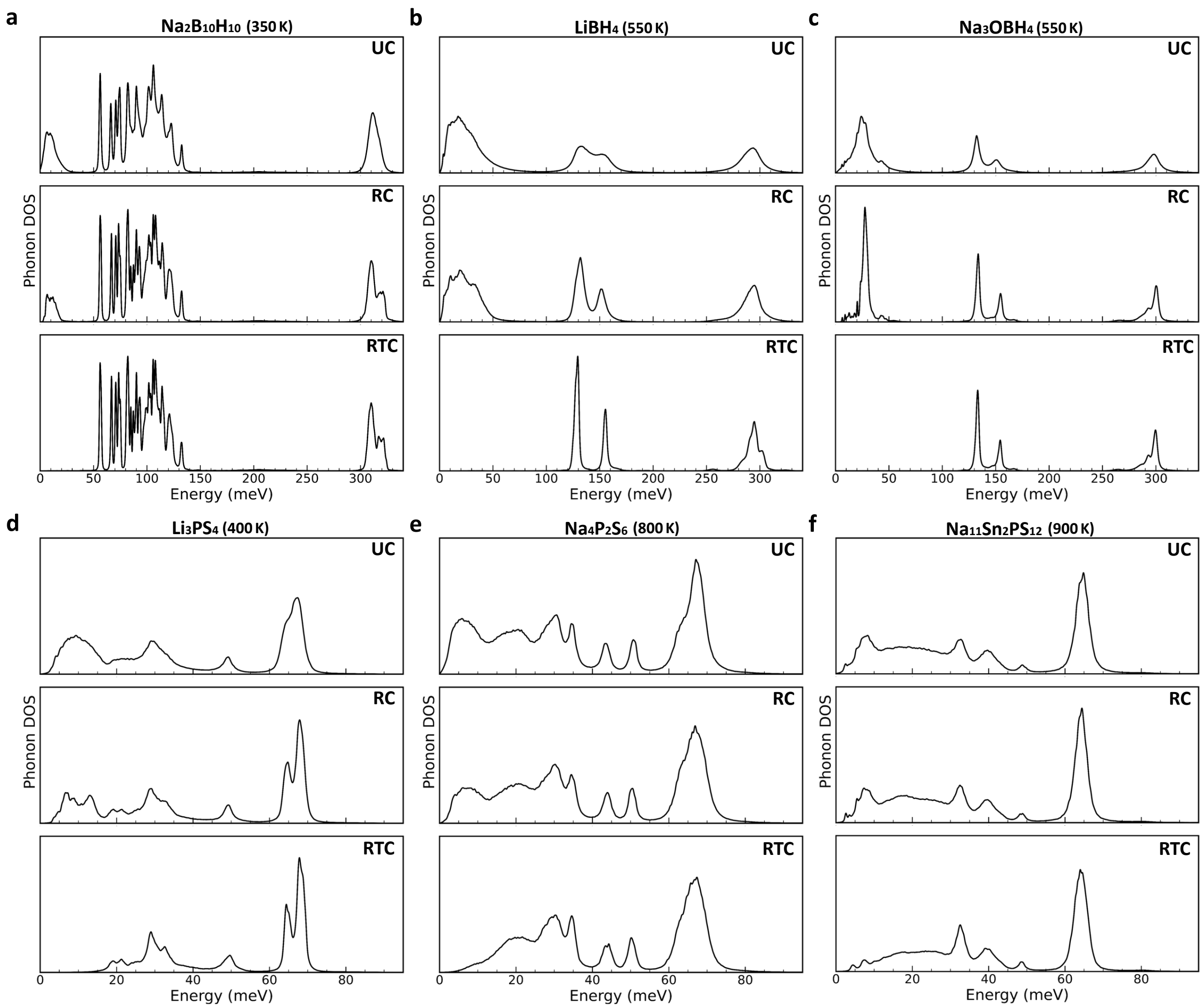


**Supplementary Fig.22** Partial density of states of rotor anions when different constraints are applied for (a) $Na_2B_{10}H_{10}$, (b) $LiBH_4$, (c) $Na_3OBH_4$, (d) $Li_3PS_4$, (e) $Na_4P_2S_6$, and (f) $Na_{11}Sn_2PS_{12}$. The abbreviation is unconstrained (UC), rotationally constrained (RC), and roto-translationally constrained (RTC).

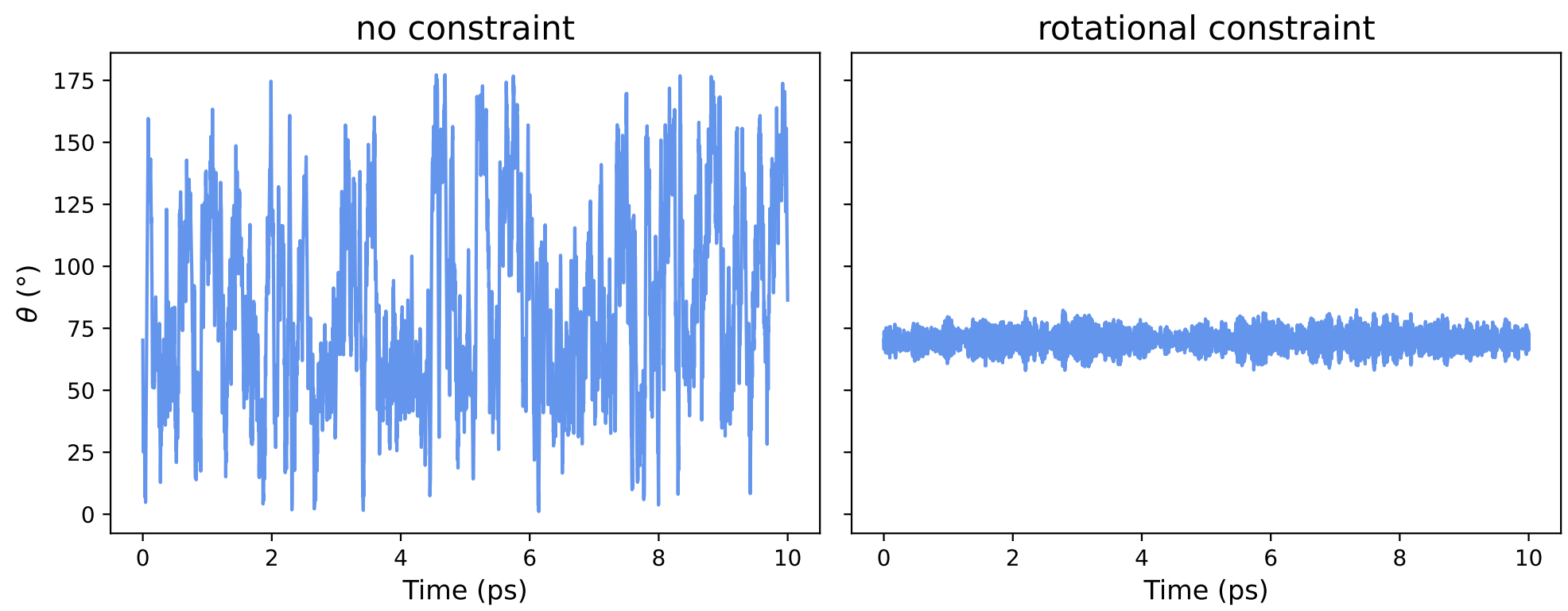


**Supplementary Fig.23** Representative angle projection of $BH_4$ with the rotational constraint algorithm applied and without constraint. The small fluctuations under rotational constraint arise from vibrational wiggling of the atoms.

**Numerical stability**

The time evolution of thermodynamical quantities, including conserved quantity and temperature for six ionic conductors are further provided in Supplementary Fig.24 and 25. The conserved quantity conserved by equations of motion in canonical ensemble should exhibit no significant drift and small fluctuations. Our conserved quantity shows minimal drift and small fluctuations, indicating the presence of rotational/roto-translational constraints is conservative on the constraint surface. Note also that the difference of conserved quantity with/without constraints comes from the DOF changed by constraints. The average temperatures in constrained canonical ensemble simulations remain consistent with those in unconstrained simulations. The temperature fluctuations with rotational (RC) or roto-translational (RTC) constraint applied are similar to those without constraint (UC), while are slightly larger under roto-translational vibrational constraint (RTVC). This occurs because fixing the motion of all anions restricts velocity rescaling to the DOF of only cations. Since the temperature fluctuation is inversely proportional to the number of DOF [17], a relative large fluctuation should be observed. In summary, constrained simulations exhibit numerical stability while maintaining thermodynamical consistency with their unconstrained counterparts.

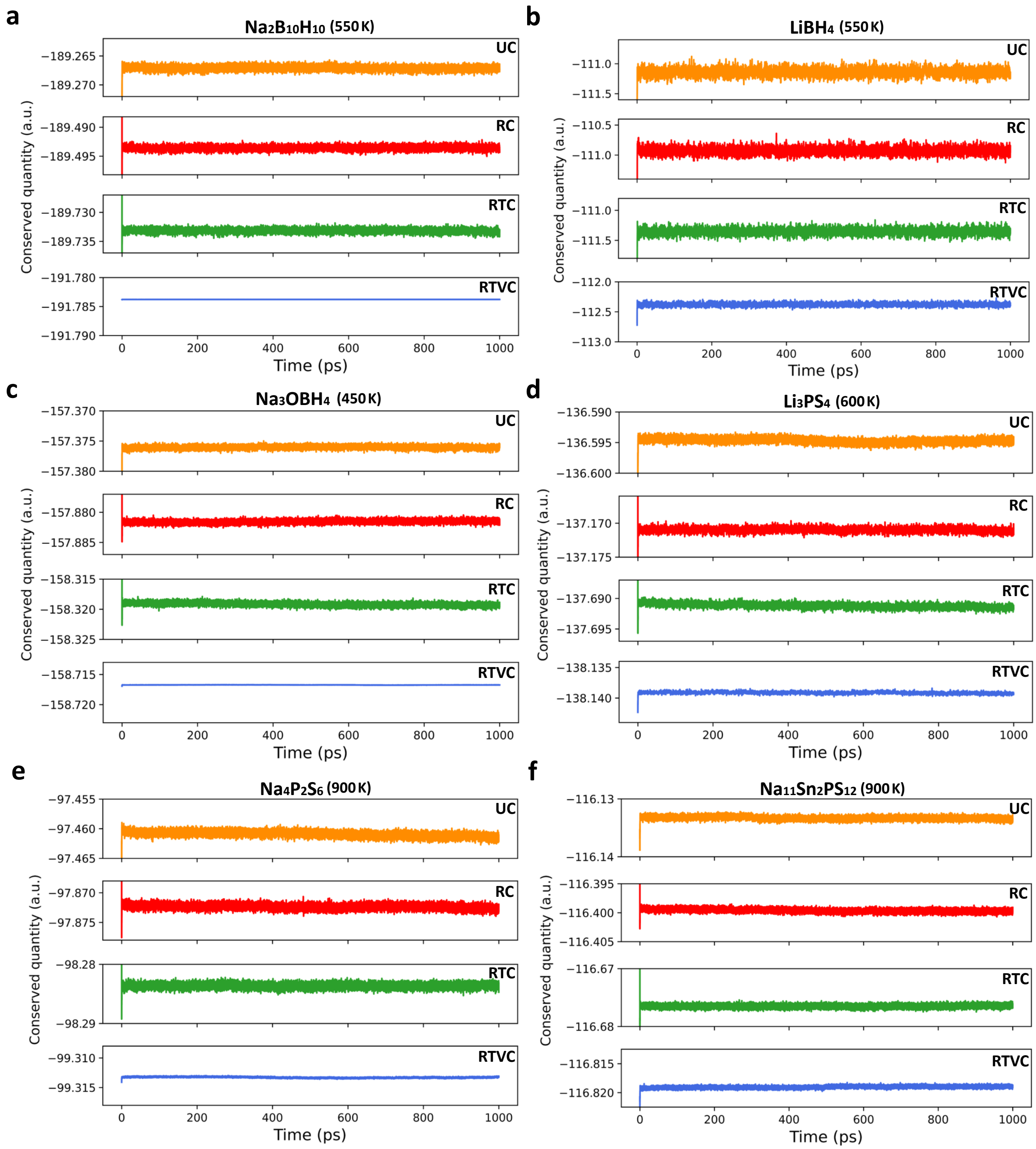


**Supplementary Fig.24** The conserved quantity (in atomic units) of the canonical ensemble fluctuates with time for (a) $Na_2B_{10}H_{10}$, (b) $LiBH_4$, (c) $Na_3OBH_4$, (d) $Li_3PS_4$, (e) $Na_4P_2S_6$, and (f) $Na_{11}Sn_2PS_{12}$. For the NVT ensemble employed here, it corresponds to the total energy of the extended system, including the degrees of freedom of the heat bath. The abbreviation is unconstrained (UC, orange), rotationally constrained (RC, red), roto-translationally constrained (RTC, green), and roto-translationally-vibrationally constrained (RTVC, blue).

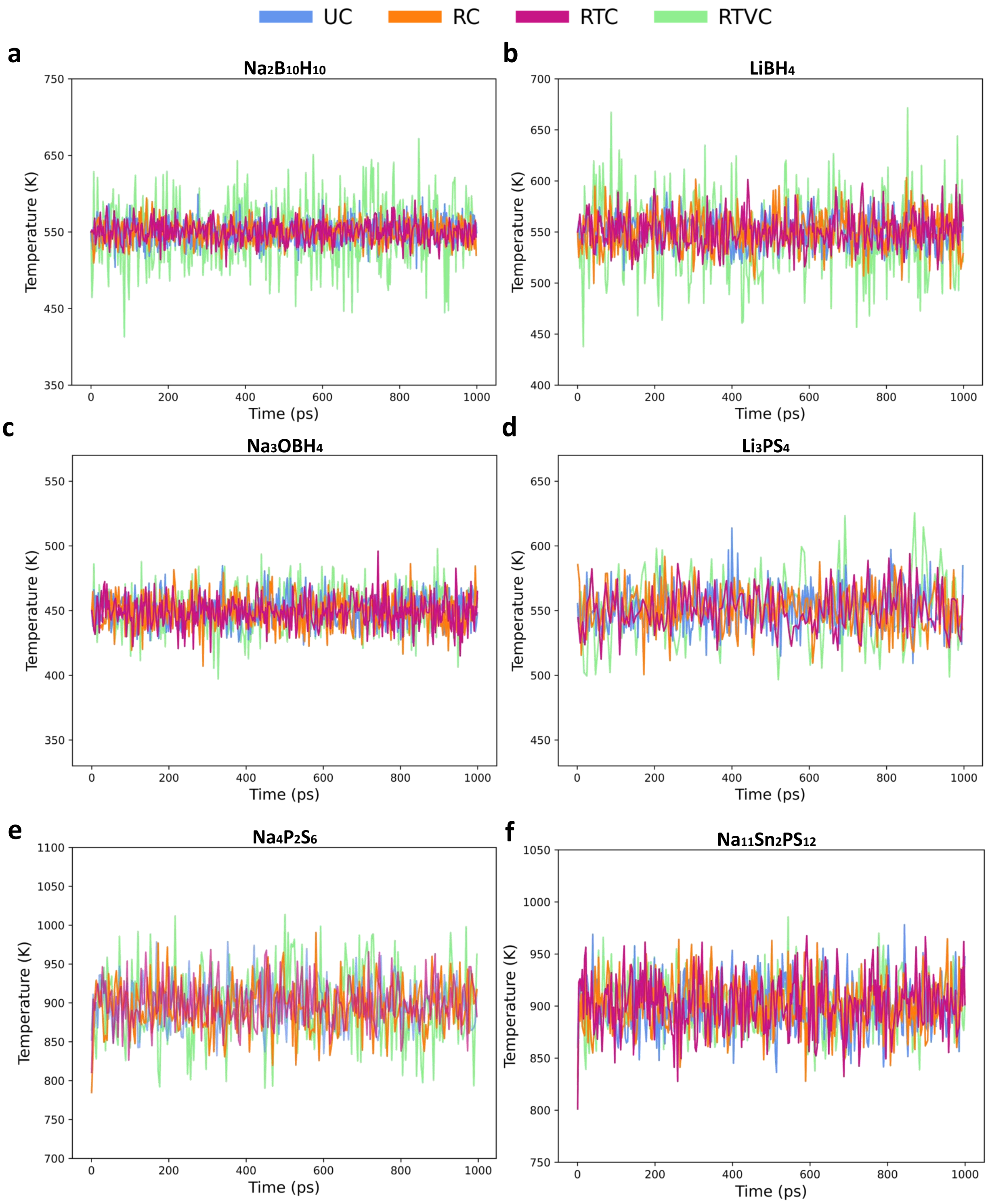


**Supplementary Fig.25** Temperature fluctuations under different constraints for (a) $Na_2B_{10}H_{10}$, (b) $LiBH_4$, (c) $Na_3OBH_4$, (d) $Li_3PS_4$, (e) $Na_4P_2S_6$, and (f) $Na_{11}Sn_2PS_{12}$. The abbreviation is unconstrained (UC, blue), rotationally constrained (RC, orange), roto-translationally constrained (RTC, purple), and roto-translationally-vibrationally constrained (RTVC, green).

## Supplementary Note 5: Supplementary Figures

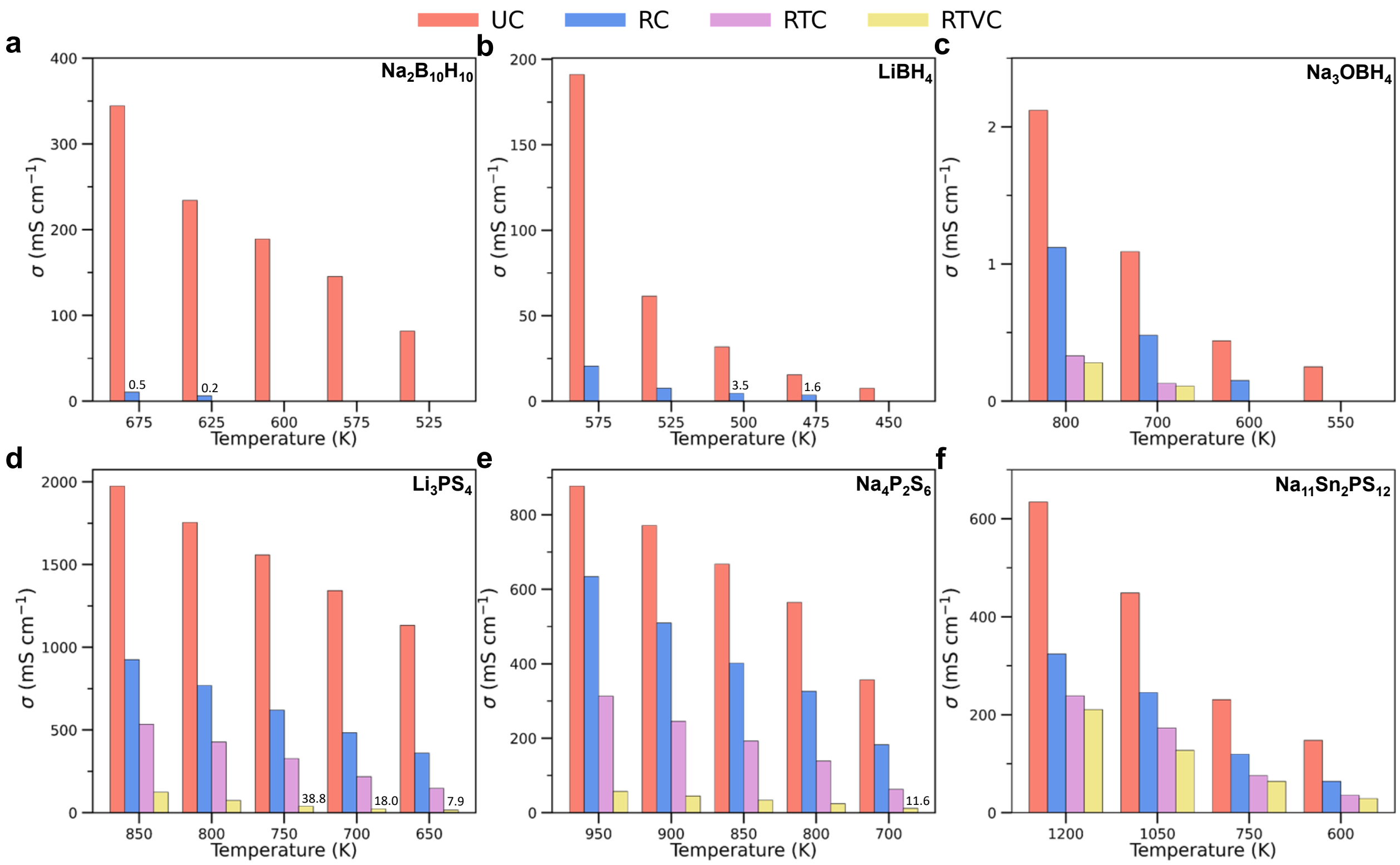


**Supplementary Fig.26** Conductivity calculated using the Nernst–Einstein approximation at various temperatures under four constrained conditions: (a) $Na_2B_{10}H_{10}$, (b) $LiBH_4$ (c) $Na_3OBH_4$, (d) $\alpha$-$Li_3PS_4$, (e) $\gamma$-$Na_4P_2S_6$, and (f) $Na_{11}Sn_2PS_{12}$. Some conductivities are absent because the ions do not exhibit sufficient migration to yield a valid linear fit. The abbreviation is unconstrained (UC, red), rotationally constrained (RC, blue), roto-translationally constrained (RTC, purple), and roto-translationally-vibrationally constrained (RTVC, yellow).

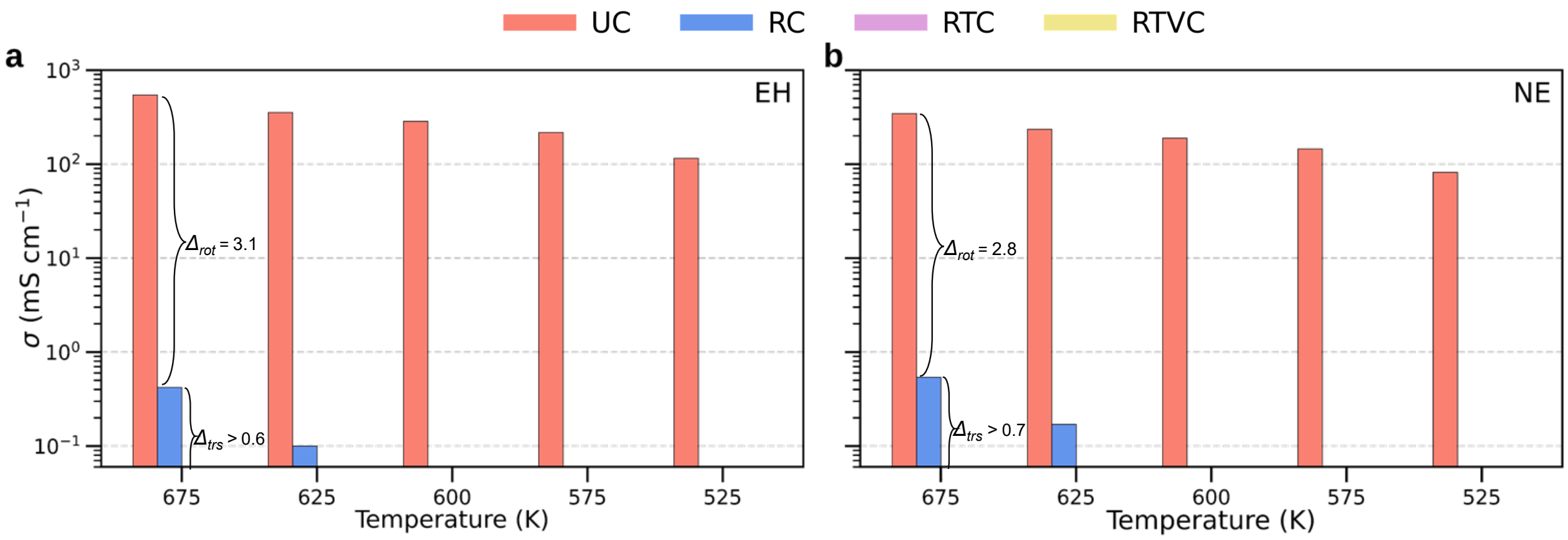


**Supplementary Fig.27** Conductivities of $Na_2B_{10}H_{10}$ calculated using the (a) Einstein–Helfand (EH) relation and (b) Nernst–Einstein (NE) approximation, plotted on logarithmic scales. Both results indicate that $B_{10}H_{10}^{2-}$ rotation plays the dominant role in Na-ion conduction over the simulated temperature range. In the left panel, constraining rotation decreases the conductivity by 3.1 orders of magnitude; further constraining translation reduces the conductivity below the measurable limit (0.1 mS cm $^{-1}$), corresponding to a additional decrease of at least 0.6 orders of magnitude. The abbreviation is unconstrained (UC, red), rotationally constrained (RC, blue), roto-translationally constrained (RTC, purple), and roto-translationally-vibrationally constrained (RTVC, yellow).

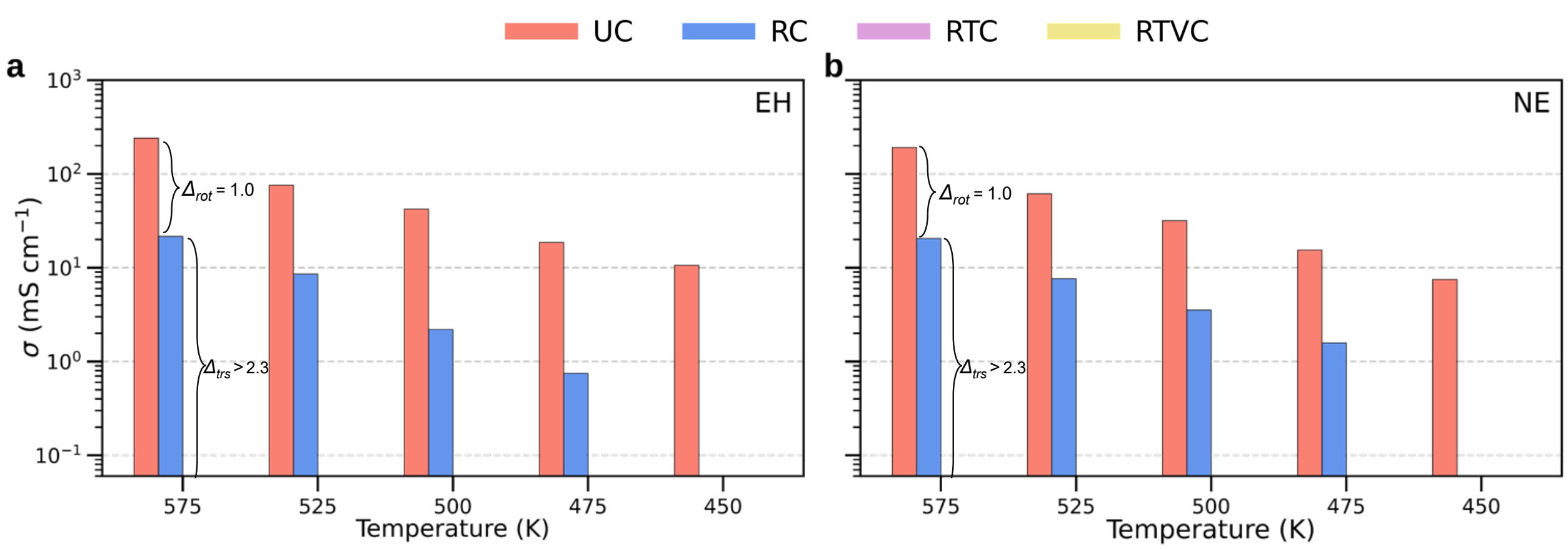


**Supplementary Fig.28** Conductivities of $LiBH_4$ calculated using the (a) Einstein–Helfand (EH) relation and (b) Nernst–Einstein (NE) approximation, plotted on logarithmic scales. Both results indicate that $BH_4^-$ translation plays the dominant role in Li-ion conduction over the simulated temperature range. In both panels, constraining rotation decreases the conductivity by 1.0 orders of magnitude; further constraining translation reduces the conductivity below the measurable limit (0.1 mS cm $^{-1}$), corresponding to a additional decrease of at least 2.3 orders of magnitude. The abbreviation is unconstrained (UC, red), rotationally constrained (RC, blue), roto-translationally constrained (RTC, purple), and roto-translationally-vibrationally constrained (RTVC, yellow).

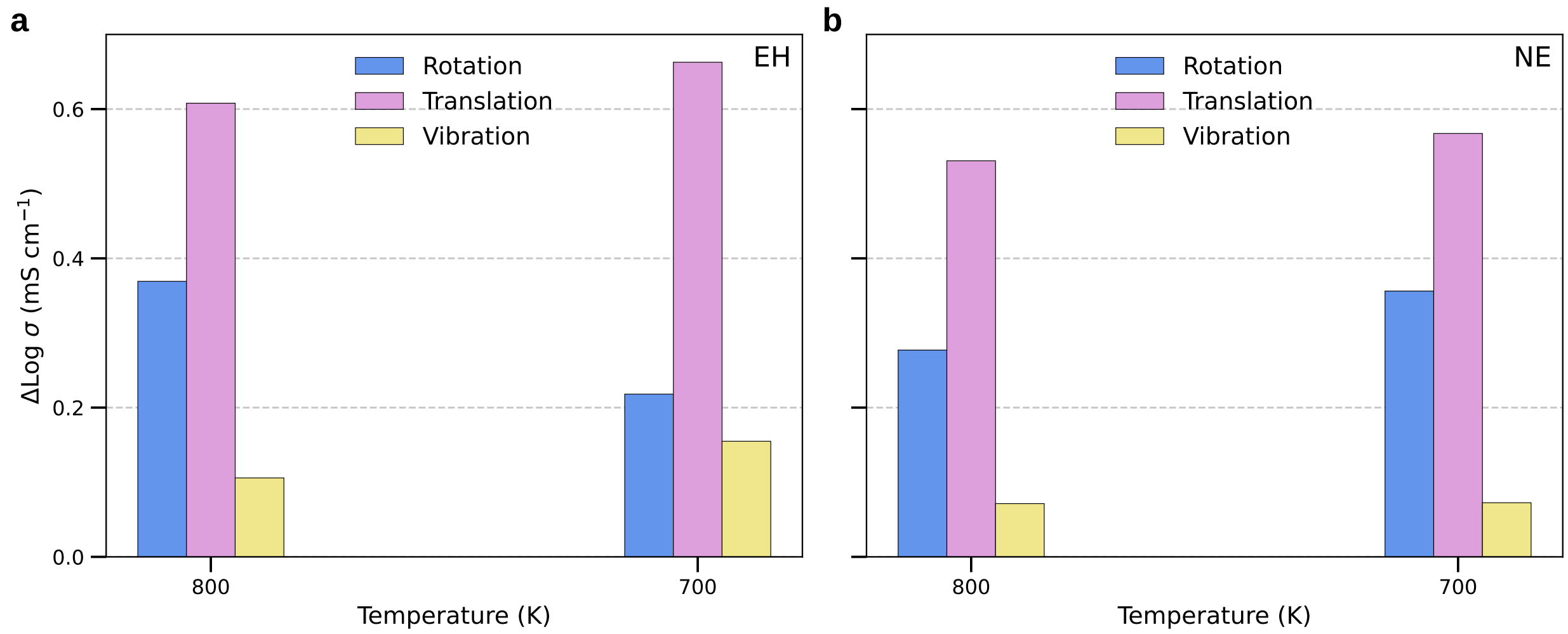


**Supplementary Fig.29** Order-of-magnitude changes in the conductivity of $Na_3OBH_4$ upon constraining anion rotation (blue), translation (purple), and vibration (yellow). Contributions are calculated as $\log_{10}(\sigma_{\mathrm{UC}}/\sigma_{\mathrm{RC}})$ for rotation, $\log_{10}(\sigma_{\mathrm{RC}}/\sigma_{\mathrm{RTC}})$ for translation, and $\log_{10}(\sigma_{\mathrm{RTC}}/\sigma_{\mathrm{RTVC}})$ for vibration. Panels (a) and (b) correspond to conductivities obtained from the Einstein–Helfand (EH) relation and the Nernst–Einstein (NE) approximation, respectively. Both the EH and NE results indicate that $BH_4^-$ translation is the dominant anion motion mode affecting ionic conductivity, followed by rotation and then vibration.

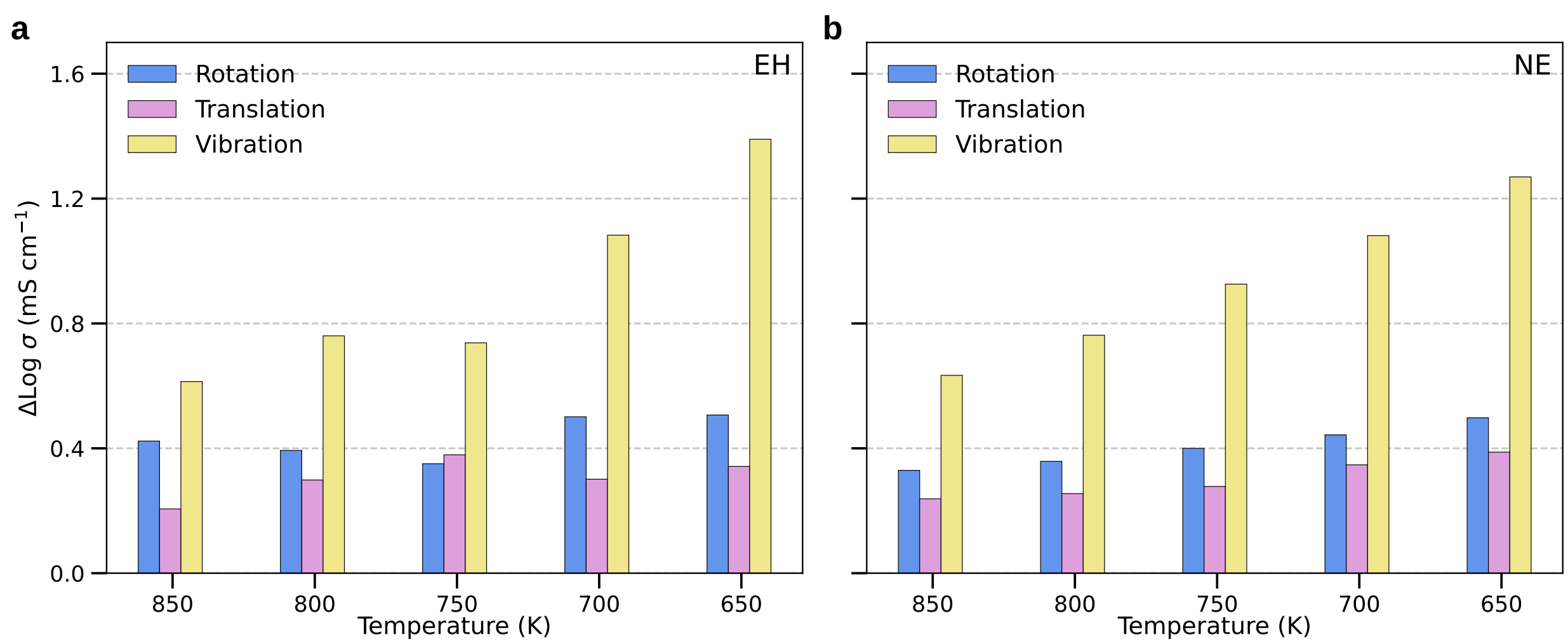


**Supplementary Fig.30** Order-of-magnitude changes in the conductivity of $\alpha$-$Li_3PS_4$ upon constraining anion rotation (blue), translation (purple), and vibration (yellow). Contributions are calculated as $\log_{10}(\sigma_{\mathrm{UC}}/\sigma_{\mathrm{RC}})$ for rotation, $\log_{10}(\sigma_{\mathrm{RC}}/\sigma_{\mathrm{RTC}})$ for translation, and $\log_{10}(\sigma_{\mathrm{RTC}}/\sigma_{\mathrm{RTVC}})$ for vibration. Panels (a) and (b) correspond to conductivities obtained from the Einstein-Helfand (EH) relation and the Nernst-Einstein (NE) approximation, respectively. Both the EH and NE results indicate that, in $Li_3PS_4$, the anion vibration is the dominant motion mode promoting conductivity over the simulated temperature range, followed by rotation and then translation.

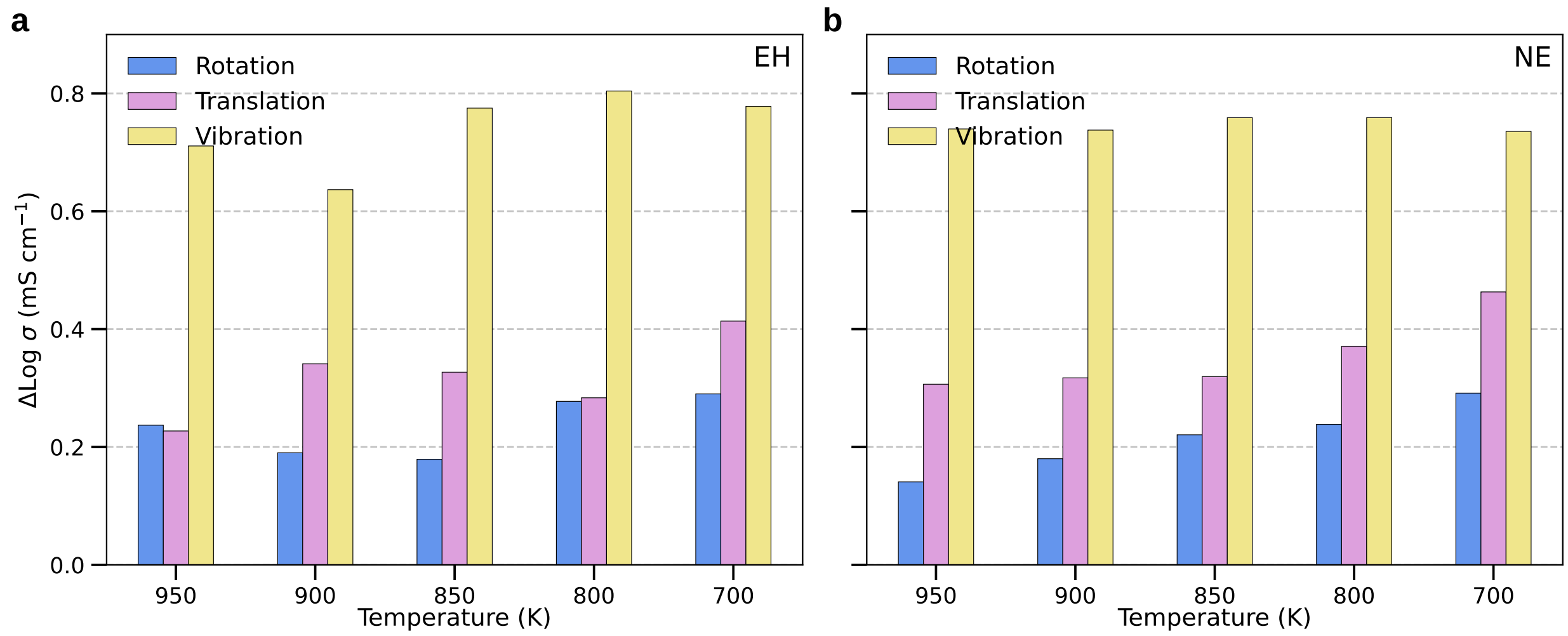


**Supplementary Fig.31** Order-of-magnitude changes in the conductivity of $Na_4P_2S_6$ upon constraining anion rotation (blue), translation (purple), and vibration (yellow). Contributions are calculated as $\log_{10}(\sigma_{\mathrm{UC}}/\sigma_{\mathrm{RC}})$ for rotation, $\log_{10}(\sigma_{\mathrm{RC}}/\sigma_{\mathrm{RTC}})$ for translation, and $\log_{10}(\sigma_{\mathrm{RTC}}/\sigma_{\mathrm{RTVC}})$ for vibration. Panels (a) and (b) correspond to conductivities obtained from the Einstein–Helfand (EH) relation and the Nernst–Einstein (NE) approximation, respectively. Both the EH and NE results indicate that, in $Na_4P_2S_6$, the anion vibration is the dominant motion mode promoting conductivity over the simulated temperature range, followed by translation and then rotation.

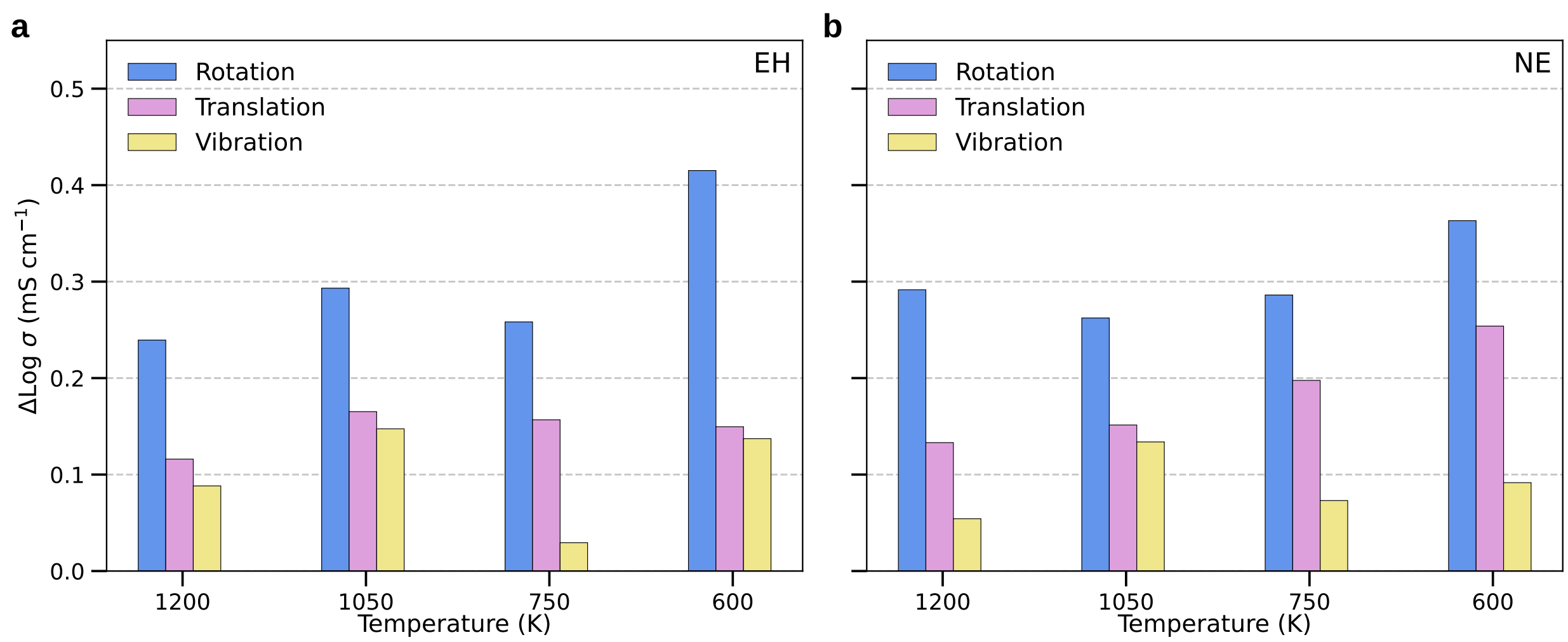


**Supplementary Fig.32** Order-of-magnitude changes in the conductivity of $Na_{11}Sn_2PS_{12}$ upon constraining anion rotation (blue), translation (purple), and vibration (yellow). Contributions are calculated as $\log_{10}(\sigma_{\mathrm{UC}}/\sigma_{\mathrm{RC}})$ for rotation, $\log_{10}(\sigma_{\mathrm{RC}}/\sigma_{\mathrm{RTC}})$ for translation, and $\log_{10}(\sigma_{\mathrm{RTC}}/\sigma_{\mathrm{RTVC}})$ for vibration. Panels (a) and (b) correspond to conductivities obtained from the Einstein–Helfand (EH) relation and the Nernst–Einstein (NE) approximation, respectively. Both the EH and NE results indicate that, in $Na_{11}Sn_2PS_{12}$, $PS_4^{3-}$ rotation has the largest effect on conductivity over the simulated temperature range, followed by translation and then vibration.

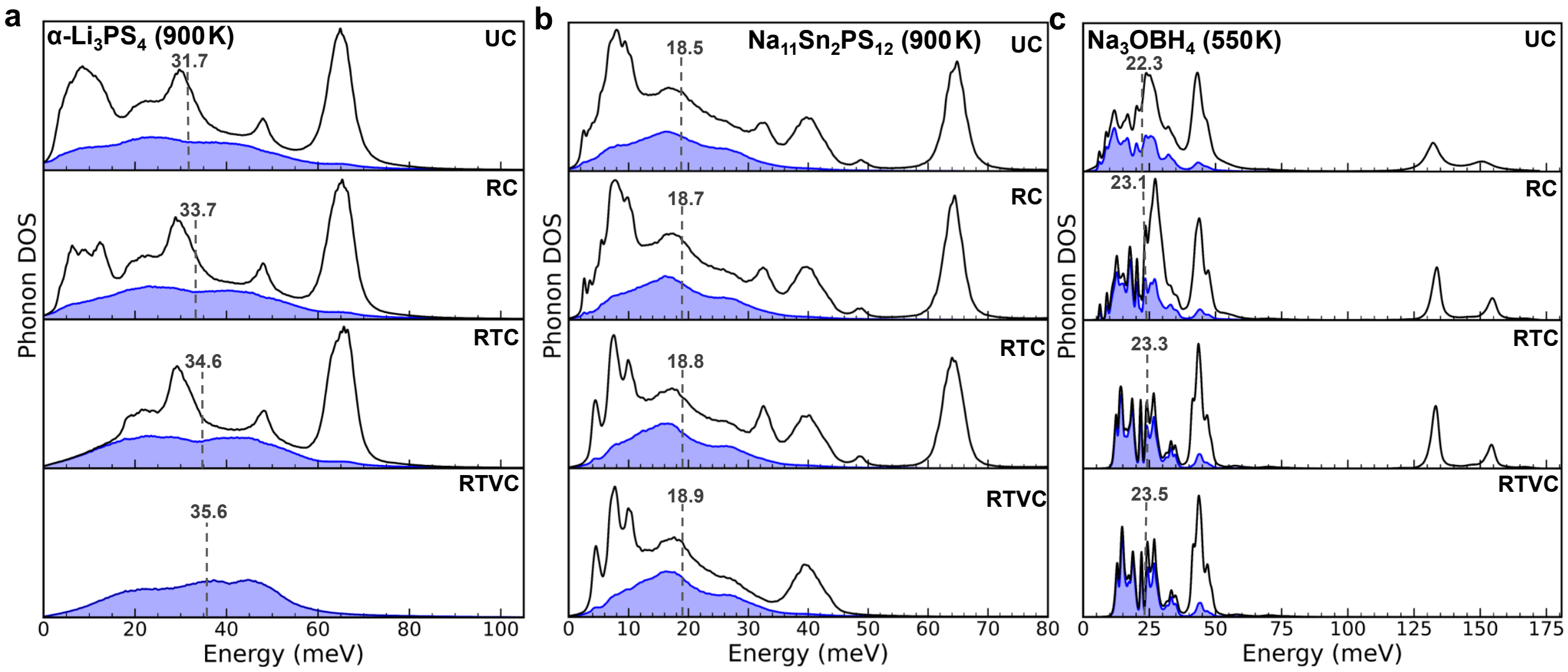


**Supplementary Fig.33** Phonon density of states of (a) $\alpha$-$Li_3PS_4$, (b) $Na_{11}Sn_2PS_{12}$ and (c) $Na_3OBH_4$ under different constraints. The black lines represent the phonon DOS, while the blue shaded areas correspond to the partial phonon DOS of mobile cation. The number and dashed line represent the cation band center. The abbreviation is unconstrained (UC), rotationally constrained (RC), roto-translationally constrained (RTC), and roto-translationally-vibrationally constrained (RTVC).

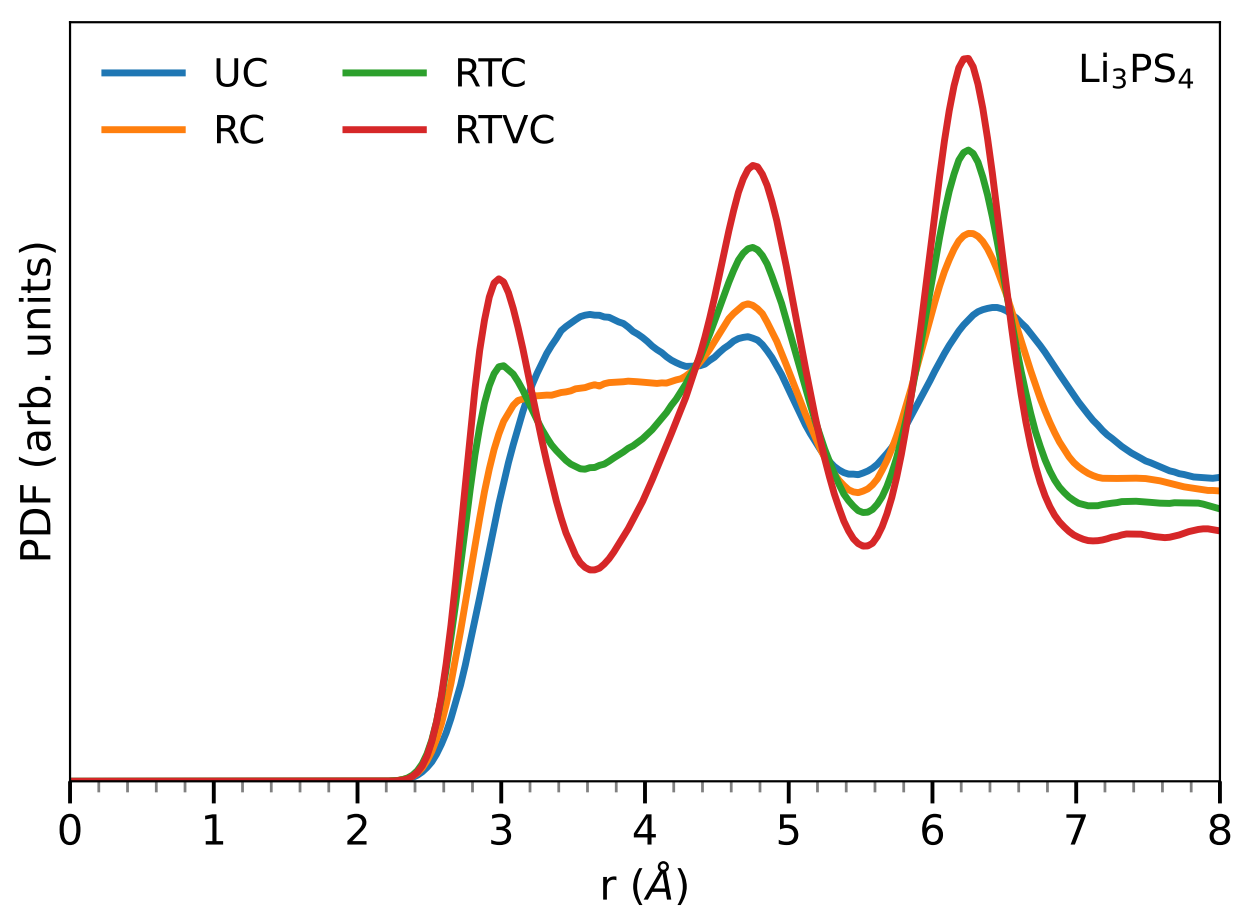


**Supplementary Fig.34** Li–Li pair distribution function (PDF) in $\gamma$-$Li_3PS_4$ under four constraint conditions, calculated from MLMD trajectories at 550 K. The abbreviation is unconstrained (UC, blue), rotationally constrained (RC, orange), roto-translationally constrained (RTC, green), and roto-translationally-vibrationally constrained (RTVC, red).

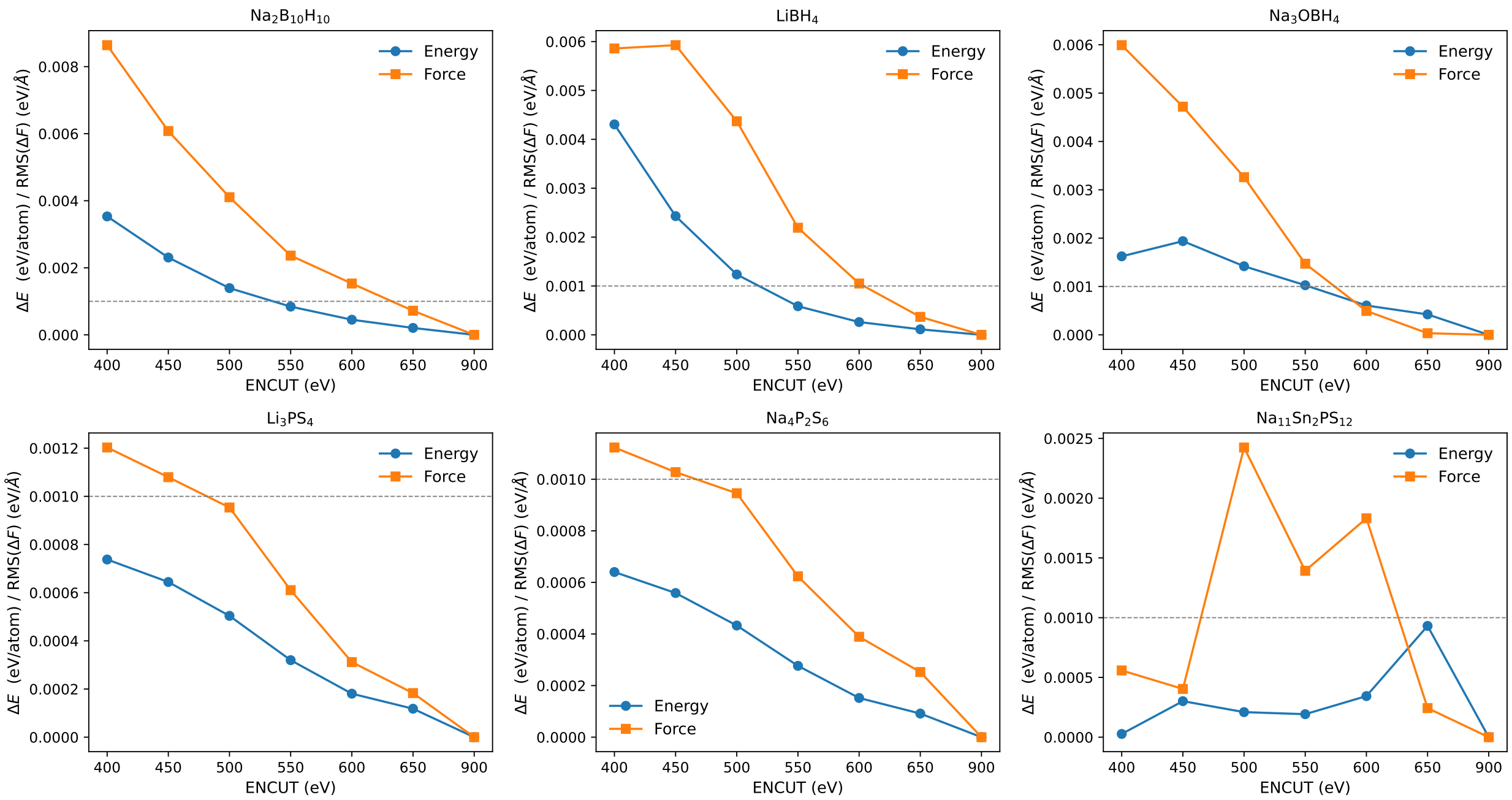


**Supplementary Fig.35** Plane-wave cutoff convergence tests for the six systems. The horizontal axis shows the plane-wave cutoff energy in eV, and the vertical axis shows the absolute changes in total energy and RMS force relative to the results obtained with the highest cutoff energy for each system.

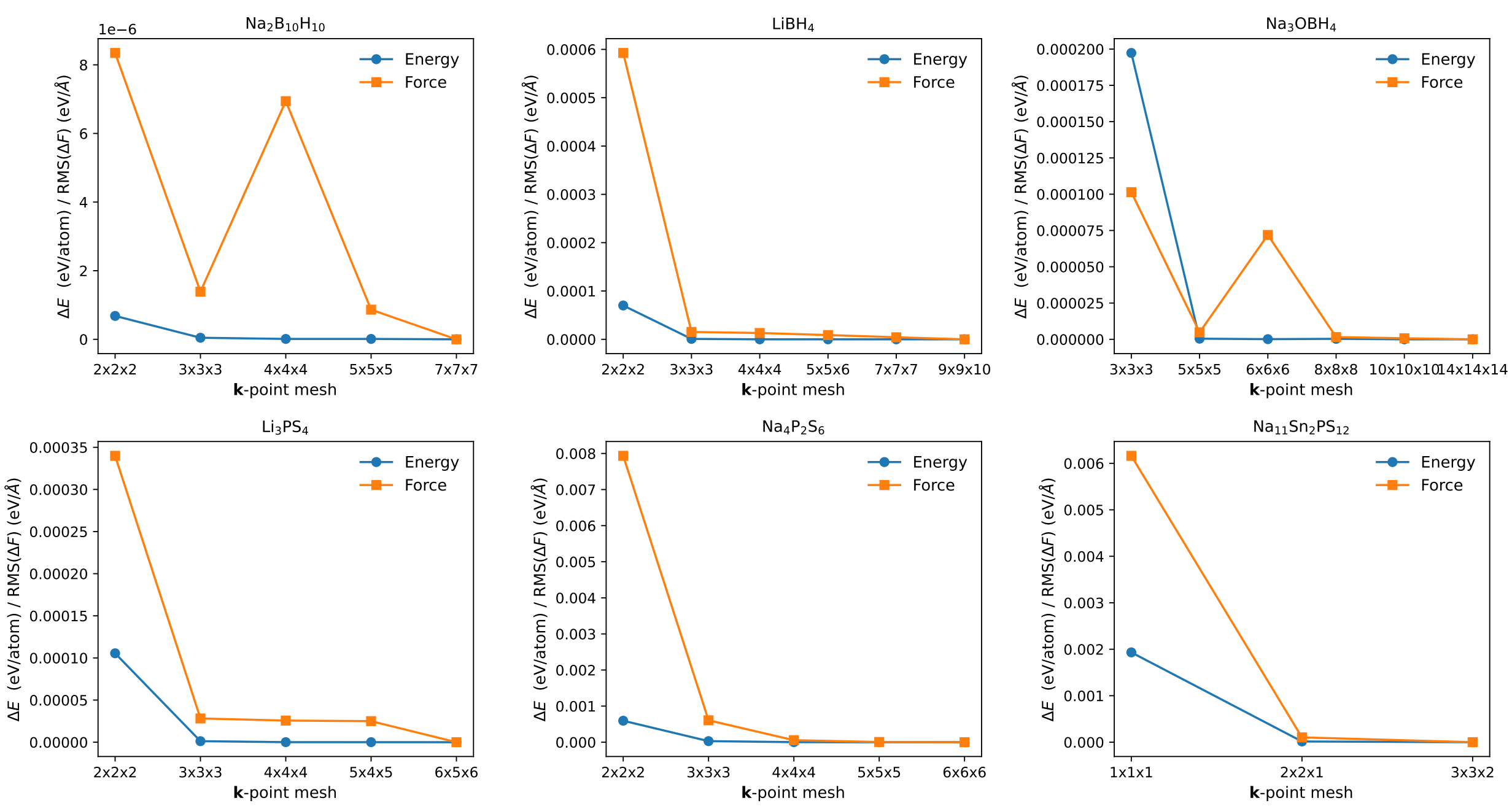


**Supplementary Fig.36 k-**point convergence tests for the six systems. The horizontal axis shows the $k$-point mesh used for Brillouin-zone sampling, and the vertical axis shows the absolute changes in total energy and RMS force relative to the results obtained with the densest $k$-point mesh for each system.

## Supplementary references


[1] Jianxing Huang, Linfeng Zhang, Han Wang, Jinbao Zhao, Jun Cheng, and Weinan E. Deep potential generation scheme and simulation protocol for the $Li_{10}GeP_2S_{12}$-type superionic conductors. *J. Chem. Phys.*, 154(9):094703, March 2021.

[2] Giulio Imbalzano, Andrea Anelli, Daniele Giofré, Sinja Klees, Jörg Behler, and Michele Ceriotti. Automatic selection of atomic fingerprints and reference configurations for machine-learning potentials. *The Journal of Chemical Physics*, 148(24):241730, June 2018.

[3] Joe D. Morrow, John L. A. Gardner, and Volker L. Deringer. How to validate machine-learned interatomic potentials. *J. Chem. Phys.*, 158(12):121501, March 2023.

[4] Sebastian Farquhar, Yarin Gal, and Tom Rainforth. On statistical bias in active learning: How and when to fix it. In *International Conference on Learning Representations (ICLR)*, 2021.

[5] KyuJung Jun, Byungju Lee, Ronald L. Kam, and Gerbrand Ceder. The nonexistence of a paddlewheel effect in superionic conductors. *Proc. Natl. Acad. Sci. U. S. A.*, 121(18):e2316493121, April 2024.

[6] Terrence J. Udovic, Motoaki Matsuo, Wan Si Tang, Hui Wu, Vitalie Stavila, Alexei V. Soloninin, Roman V. Skoryunov, Olga A. Babanova, Alexander V. Skripov, John J. Rush, Atsushi Unemoto, Hitoshi Takamura, and Shin-ichi Orimo. Exceptional Superionic Conductivity in Disordered Sodium Decahydro-*closo* -decaborate. *Adv. Mater.*, 26(45):7622–7626, December 2014.

[7] Tanja Scholz, Christian Schneider, Maxwell W. Terban, Zeyu Deng, Roland Eger, Martin Etter, Robert E. Dinnebier, Pieremanuele Canepa, and Bettina V. Lotsch. Superionic Conduction in the Plastic Crystal Polymorph of $Na_4P_2S_6$. *ACS Energy Lett.*, 7(4):1403–1411, April 2022.

[8] Zhizhen Zhang, Pierre-Nicholas Roy, Hui Li, Maxim Avdeev, and Linda F Nazar. Coupled Cation–Anion Dynamics Enhances Cation Mobility in Room-Temperature Superionic Solid-State Electrolytes. *J. Am. Chem. Soc.*, 141(49):19360–19372, December 2019.

[9] Arndt Remhof, Zbigniew Łodziana, Pascal Martelli, Oliver Friedrichs, Andreas Züttel, Alexander V. Skripov, Jan Peter Embs, and Thierry Strässle. Rotational motion of $BH_4$ units in $MBH_4$ ( M = Li , Na , K ) from quasielastic neutron scattering and density functional calculations. *Phys. Rev. B*, 81(21):214304, June 2010.

[10] Yulong Sun, Yuechao Wang, Xinmiao Liang, Yuanhua Xia, Linfeng Peng, Huanhuan Jia, Hanxiao Li, Liangfei Bai, Jiwen Feng, Hong Jiang, and Jia Xie. Rotational Cluster Anion Enabling Superionic Conductivity in Sodium-Rich Antiperovskite $Na_3OBH_4$. *J. Am. Chem. Soc.*, 141(14):5640–5644, April 2019.

[11] Motoaki Matsuo, Yuko Nakamori, Shin-ichi Orimo, Hideki Maekawa, and Hitoshi Takamura. Lithium superionic conduction in lithium borohydride accompanied by structural transition. *Appl. Phys. Lett.*, 91(22):224103, November 2007.

[12] Yuzhi Zhang, Haidi Wang, Weijie Chen, Jinzhe Zeng, Linfeng Zhang, Han Wang, and Weinan E. DP-GEN: A concurrent learning platform for the generation of reliable deep learning based potential energy models. *Comput. Phys. Commun.*, 253:107206, August 2020.

[13] Ji-Su Kim, Wo Dum Jung, Sungjun Choi, Ji-Won Son, Byung-Kook Kim, Jong-Ho Lee, and Hyoungchul Kim. Thermally Induced S-Sublattice Transition of $Li_3PS_4$ for Fast Lithium-Ion Conduction. *J. Phys. Chem. Lett.*, 9(18):5592–5597, September 2018.

[14] Z. Zhang, E. Ramos, F. Lalère, A. Assoud, K. Kaup, P. Hartman, and Linda F. Nazar. $Na_{11}Sn_2PS_{12}$ : a new solid state sodium superionic conductor. *Energy Environ. Sci.*, 11(1):87–93, 2018.

[15] Ming Zeng, Carlos Escorihuela-Sayalero, Tamio Ikeshoji, Shigeyuki Takagi, Sangryun Kim, Shin-ichi Orimo, María Barrio, Josep-Lluís Tamarit, Pol Lloveras, Claudio Cazorla, and Kartik Sau. Colossal Reversible Barocaloric Effects in a Plastic Crystal Mediated by Lattice Vibrations and Ion Diffusion. *Advanced Science*, 11(26):2306488, July 2024.

[16] A. Amadei, G. Chillemi, M. A. Ceruso, A. Grottesi, and A. Di Nola. Molecular dynamics simulations with constrained roto-translational motions: Theoretical basis and statistical mechanical consistency. *The Journal of Chemical Physics*, 112(1):9–23, January 2000.

[17] J. Hickman and Y. Mishin. Temperature fluctuations in canonical systems: Insights from molecular dynamics simulations. *Physical Review B*, 94(18):184311, November 2016.
