# Supplementary material for: Disentangling cation–polyanion coupling reveals which anion motion dominates cation transport in solid electrolytes
Source: Nat Commun. 2026 Sep 2;17:10455. doi: 10.1038/s41467-026-77273-x (PMC13635178; doi:10.1038/s41467-026-77273-x)
Supplement: Supplementary file 2 [file 41467_2026_77273_MOESM2_ESM.pdf]

nature portfolio

Peer Review File

Disentangling Cation–Polyanion Coupling Reveals Which Anion Motion Dominates Cation Transport in Solid Electrolytes

Corresponding Author: Professor Hui Li

Parts of this Peer Review File have been redacted as indicated to remove third-party material.

This file contains all reviewer reports in order by version, followed by all author rebuttals in order by version.

Attachments originally included by the reviewers as part of their assessment can be found at the end of this file.

Version 0:

Reviewer comments:

Reviewer #1

(Remarks to the Author)
See attached report.

(Remarks on code availability)
The code provided is largely a copy of the code CP2K, https://www.cp2k.org/. As such it works. Other materials, such as the trained models can be found at https://github.com/KeouLee/SE_MLIP, as stated in the Data Availability statement. Honestly, I would suggest avoiding the use of statements such as "Other source data are available from the corresponding authors upon request." in the Data Availability statement, since, in this way, reproducibility may be lost.

Reviewer #2

(Remarks to the Author)
I co-reviewed this manuscript with one of the reviewers who provided the listed reports. This is part of the Nature Communications initiative to facilitate training in peer review and to provide appropriate recognition for Early Career Researchers who co-review manuscripts.

(Remarks on code availability)

Reviewer #3

(Remarks to the Author)
Understanding anion participation in the transport of active ions, lithium/sodium (Li/Na), in solid-state materials has been a great debate. Significant imprecise and far-fetched literature with claims of concerted anion motion, paddlewheel effects has been published in the last 40 years, with a resurgence in recent years with the “rediscovery” of ion conductivity in solid crystalline materials. The manuscript of Li and collaborators investigated the participation of anion motion in the lithium/sodium (Li/Na) ion motion in ionic conductor materials, also known as solid electrolytes. In my opinion, the most interesting part of this paper is how the motion of polyanion groups increases the local disorder, with the global outcome of flattening the potential energy surface. They used a combination of standard techniques, density functional theory, and machine learning potential. The work is technically sound, but with several imprecisions and paradoxical statements.

In summary, the topic is important. However, the analysis of the data is quite simplistic, and many assumptions are made. This study cannot be considered in its present state. Finally, these authors are not aware/up to date on the existing literature on this topic.

Comments:
1. The language of the manuscript is very assertive and borderline overreaching. For example, in the abstract, they state,

“Here, we present a pioneering study…”. What’s so pioneering about this study?

2. One crucial aspect that is never discussed in this study is whether they introduce lithium (sodium) vacancies in the 6 solid electrolytes investigated, or whether they believe that interstitials will provide sufficient Li/Na-ion transport. Furthermore, the vacancy concentration features directly in Eq. 2 in the manuscript.

3. I am very curious about their method for freezing vibrational degrees of freedom. This is an integral part of the manuscript, but they don’t make any effort to describe this in any detail.

4. I am pretty disappointed that they don’t even attempt or define “correlated” center of mass diffusivity. Still, instead, they just used the tracer diffusivity in Eq. 1. Tracer diffusivity is the simplest definition of diffusivity and doesn’t account for any correlation. These authors should revise some essential basics of ion transport in solids, e.g. 10.1103/PhysRevB.64.184307, or more recently 10.1021/acsmaterialsau.2c00057

5. In the intro, they claimed they are doing “ultralong-time” simulations. This is very speculative and incorrect. First of all, their simulations fall short if compared to any classical molecular dynamics simulations. Therefore, they’re unable to capture any correlation properties and even some basic ion transport properties. For example, they didn’t report an Arrhenius plot in Figure 3f for LiBH4, because they could not converge their MSDs for this system. Kinetic Monte Carlo simulation as in 10.1038/s41467-022-32190-7 or 10.1098/rsos . 170824, are the only methods that can reach “ultra-long” and spatially adequate simulations in the millisecond regime. These authors should comment on these publications as gold standards for ion transport.

6. Regarding Figure 3f, some fits do not appear linear; for instance, Li3PS4. Are they seeing a phase transition? Is there a sudden change in slope at higher temperatures? They don’t comment on this important aspect.

7. In Figure 2, the use of octants and the analysis of changes in angle projections over time was introduced in 10.1021/acsenergylett.1c02815, which they cite. It would be appropriate if they acknowledged this idea. In this context, a better treatment of anion rotations can be achieved through the use of quaternions and quaternion coordinates as in 10.1073/pnas.2316493121. Have these authors attempted the implementation of quaternion analysis for their data?

8. Another important layer of disorder that is not discussed but is undoubtedly present in Na11Sn2PS12 is the mixing of SnS4 and PS4 polyanion. This mixing certainly contributes to changes in ion transport. Polyanion mixing/disorder certainly changes the potential energy surface perceived by a traveling ion. This is central in NaSICON structure, where some of these authors have published before. However, molecular dynamics cannot describe these mixed anion species properly, and stochastic methods, such as Monte Carlo are paramount 10.1038/s41467-022-32190-7, and 10.1021/acs.chemmater.0c02695.

9. I am utterly surprised to read that SnS4 units are immobile in Na11Sn2PS12. This could be because they had just chosen a convenient, perhaps unrepresentative, arrangement/ordering of the PS4/SnS4 in their MDs. As explained in point 7., this question can only be addressed by performing MD (or better Monte Carlo) on a significantly varied set of PS4/SnS4 arrangments.

10. In Figure 2c, it’s unclear if P2S6 or PS4. The inset is confusing. Particularly, what data are they currently showing?

11. On page 7 they bring about the concept of moment of inertia. This is important, and they should be more quantitative about this by computing the various moments of inertia of the various polyanion species investigated.

12. The definition of plastic structures is not given.

13. They used the terminology “cation local vibration frequencies”. For me, it’s extremely tough to visualize/understand “cation local vibration frequencies”, as a vibrational mode should always include at least 2 entities. So, how can we define a cation-only local vibrational frequency? To me, this is an ill-defined term. They should revise this part as it doesn’t make much sense.

14. Most of the DFT settings are not available, and therefore, any reader cannot reproduce these simulations. What is the plane-wave cut-off used? And how many valence electrons were treated explicitly with the PAW used? What DFT total energy convergence was used? What integrator did they use to integrate the equation of motion? All these details are vital to any interested reader. Why did they use DP-GEN as opposed to other existing machine learning interatomic potential methods? Likewise, no details are given about the MDs using CP2K, these are ab initio MDs, where they do not say how they choose the basis set and a functional. I had to dig out this information . In summary, these details are extremely important. However, this part appears quite incomplete.

(Remarks on code availability)
The Code is just an input for CP2K, a popular DFT code. The README appears incomplete. While I could install the code, they didn't provide sufficient details and examples.

Version 1:

Reviewer comments:

Reviewer #1

(Remarks to the Author)
See attachment.

(Remarks on code availability)
Yes. The authors now provide the relevant instructions for installation and running the code on the GitHub page linked in the manuscript.
About data availability, see the last point of the review document attached.

Reviewer #2

(Remarks to the Author)
I co-reviewed this manuscript with one of the reviewers who provided the listed reports. This is part of the Nature Communications initiative to facilitate training in peer review and to provide appropriate recognition for Early Career Researchers who co-review manuscripts.

(Remarks on code availability)

Reviewer #3

(Remarks to the Author)
I have read the extensive and carefully drafted reply by these authors. In my opinion this manuscript is ready for publication.

(Remarks on code availability)

Version 2:

Reviewer comments:

Reviewer #1

(Remarks to the Author)
The revised version and the Authors' replies are fully satisfactory and in our opinion the manuscript meets the publication standards of Nature Communications.

As a final remark, the Authors should explicitly state, as in the previous version, that the charges (q_i) employed in the formulae for the calculation of the conductivity are integer oxidation numbers. This assumption is not merely a convenient modelling choice, but is also supported by the modern ab initio theory of charge transport in electrolytes, according to which the dc electrical conductivity can be expressed in terms of integer (topological) charges, due to quantisation of charge transport.

(Remarks on code availability)

Reviewer #2

(Remarks to the Author)
I co-reviewed this manuscript with one of the reviewers who provided the listed reports. This is part of the Nature Communications initiative to facilitate training in peer review and to provide appropriate recognition for Early Career Researchers who co-review manuscripts.

(Remarks on code availability)

Open Access This Peer Review File is licensed under a Creative Commons Attribution 4.0 International License, which permits use, sharing, adaptation, distribution and reproduction in any medium or format, as long as you give appropriate credit to the original author(s) and the source, provide a link to the Creative Commons license, and indicate if changes were made.
In cases where reviewers are anonymous, credit should be given to 'Anonymous Referee' and the source.
The images or other third party material in this Peer Review File are included in the article's Creative Commons license, unless indicated otherwise in a credit line to the material. If material is not included in the article's Creative Commons license and your intended use is not permitted by statutory regulation or exceeds the permitted use, you will need to obtain permission directly from the copyright holder.
To view a copy of this license, visit https://creativecommons.org/licenses/by/4.0/

Table: Mapping from the revised manuscript to the original manuscript.

Revised manuscript	Original manuscript
subsections in the Results section	
Subsec. 1: Quantifying each polyanion motion mode's contribution to conductivity	Subsec. 2: Quantifying cation-anion coupling dynamics
Subsec. 2: Anion rotation enhances cooperative transport in borohydrides (new)	—
Subsec. 3: The paddle-wheel effect dominates only under frequency matching	Subsec. 1: Anion rotation and constraint algorithm
Subsec. 4: Anion dynamics tune conductivity through barrier and prefactor	Subsec. 3: Correlating anion dynamics with migration enthalpy and prefactor
Subsec. 5: Unchanged	Subsec. 4: Anion dynamics drive superionicity via coupling and disorder
Figures	
Fig. 1: crystal structures	Figure 1
Fig. 2: conductivity	Figure 3
Fig. 3: Haven ratio (new)	—
Fig. 4: rotor rotation frequency/barrier; cation hop frequency	Figure 2
Fig. 5: cation migration barrier	Figure 4
Fig. 6: anion translation/rotation modulates cation migration barriers and site occupancy.	Figure 6
Fig. 7: phonon density of states	Figure 5

Contents

Response to Reviewer 1 p. 2
Response to Reviewer 3 p. 8
Additional revisions p. 41
Revision of the Abstract p. 41
Revision of the Introduction p. 43
Revision of the Reults p. 47
Revision of the Discussion p. 61
Revision of the Methods & Supplementary Information p. 63

Reviewer #1 (Remarks to the Author):

The manuscript "Disentangling Cation-Polyanion Coupling in Solid Electrolytes: Which Anion Motion Dominates Cation Transport?" by K. Li and co-authors tackles the problem of understanding the correlation between the anion motion and the ionic conductivity in SSE. The problem is timely and very relevant to the community.

However, the work presents a major critical issue that impedes me from suggesting publication.

The Authors compute the conductivity via the Nernst-Einstein (NE) relation (see Eq. (2), p. 21).
However, this relation is an approximation that discards by construction the correlations:

- among the carriers (in this case, the cations)
- between the carriers and the solid matrix of the SSE.

In order to include these effects, one should compute the ionic conductivity via the full Green-Kubo (GK) relation:

$$\sigma_{GK} = \frac{\Omega}{3k_BT}\int_0^\infty \langle \vec{J}(t)\cdot\vec{J}(0)\rangle dt$$

employing the full charge flux

$$\vec{J}(t) = \frac{e}{\Omega}\sum_{i=1}^{N} q_i\vec{v}_i(t)$$

where the sum is extended over all the N atoms of the simulation cell, of volume Ω. The full GK expression is able to take into account the interactions among the ions. These correlation effects are at the basis of the so-called Haven ratio:

$$H_R = \frac{\sigma_{NE}}{\sigma_{GK}}$$

i.e. the ratio between the NE estimate for the ionic conductivity to its full value, which can indeed substantially deviate from 1. In SSEs it is often just a smaller fraction, since the concerted motion of ions is able to facilitate the motion of charge carriers at an effectively decreased energetic cost. This is also evidenced in energy landscape analysis in concerted migration of charge carriers, such as in He, X., Zhu, Y. & Mo, Y. "Origin of fast ion diffusion in super-ionic conductors." Nat Commun 8, 15893 (2017).
https://doi.org/10.1038/ncomms15893.

Methods that are solely based on atomic diffusivities cannot capture correlations. See, e.g., A. Marcolongo, N. Marzari, Phys. Rev. Materials 1, 025402, DOI:
https://doi.org/10.1103/PhysRevMaterials.1.025402 for more information and examples about this crucial drawback of NE approximation, compared with full GK relation, in SSEs.

Therefore, most of the results the Authors present aim at clarifying the role of interionic correlations, but are based on calculations of the ionic conductivity that neglect these correlations by constructions, which is a severe inconsistency that flaws the reliability of the conclusions drawn. Notice that freezing or constraining the motion of the anionic groups does not resolve these issues, since interionic correlations among charge carriers may still be present, and dependent on the constraints. Once again, the latter cannot be captured by atomic-diffusion based estimates, like the NE relation. The use of carriers' atomic diffusivity alone can depend on interionic correlations only in "mean field", but the explicit, dynamical, time dependence of the correlations (which is what defines, e.g., the paddlewheel effects) are not captured.

We are grateful to the reviewer for the careful and thoughtful reading of our manuscript, and in particular for recognising the timeliness and relevance of the question we set out to address. We also thank the reviewer for raising a fundamental methodological point that goes to the heart of how ionic conductivity should be evaluated in solid electrolytes.

We fully agree with the reviewer that the Nernst–Einstein relation, by construction, neglects inter-ionic correlations that are rigorously captured by the Green–Kubo expression, and that the resulting Haven ratio can deviate substantially from unity in superionic conductors. In the revised manuscript we have accordingly recomputed all conductivities using the Einstein–Helfand (EH) relation—formally equivalent to Green–Kubo but with superior statistical behaviour—which retains all inter-ionic correlations. We further provide, in the Appendix, an explicit proof that in crystalline solid electrolytes with a non-diffusive framework the framework self-term and the carrier–framework cross-term in the EH expression both vanish in the long-time limit, so that the EH conductivity is rigorously given by the mobile-carrier contribution alone, without any approximation regarding cation–framework correlations. We also thank the reviewer for directing us to two key references on this point, both of which have now been cited and discussed in the revised manuscript:

Added citation (*Introduction*, lines 121–123):
"However, given the high concentration of charge carriers in superionic conductors, ion–ion correlations can play an important role[56,57,58] and lead to the failure of this approximation.[59]"

Acting on this comment, we have carried out substantial additional work and added a considerable amount of new content to the revised manuscript. We were pleased to find that engaging seriously with the reviewer's concern led us to several new and, in our view, rather illuminating conclusions, which we believe have strengthened the paper. In what follows we address this comment in detail, with the corresponding revisions to the manuscript listed at the end.

The conductivity is expressed through EH relation, in terms of the slope of the mean square dipole displacement, $\Delta\boldsymbol{\mu}(t)$, as a function of time (*Phys. Rev.* **119**, 1–9 (1960); *Nat. Phys.* **15**, 967–972 (2019)).

$$\sigma = \frac{1}{3\Omega k_B T} \lim_{t\to\infty} \frac{\langle|\Delta\boldsymbol{\mu}(t)|^2\rangle}{2t} \tag{1}$$

$$\Delta\boldsymbol{\mu}(t) = \int_0^t \sum_i \mathbf{Z}_i^*(t') \cdot \mathbf{v}_i(t') dt' \tag{2}$$

where Ω and T are the system volume and temperature, $\mathbf{Z}_i^*$ and $\mathbf{v}_i$ are the Born effective charge and velocity of the ith atom and k_B is the Boltzmann constant. Jiang et al. showed that the oxidation state of ions in insulating solids can be rigorously defined (*Phys. Rev. Lett.* **108**, 166403 (2012)).Grasselli and Baroni further showed that the adiabatic electrical conductivity can be rigorously expressed in terms of integer-valued, scalar, and time-independent atomic oxidation numbers, instead of time-dependent Born effective charges (*Nat. Phys.* **15**, 967–972 (2019)).Therefore, for the six electronically insulating solid electrolytes studied here, eq 1 can be rewritten as

$$\sigma = \frac{1}{3\Omega k_B T} \lim_{t\to\infty} \frac{\langle|\sum_i q_i(\mathbf{r}_i(t) - \mathbf{r}_i(0))|^2\rangle}{2t} \tag{3}$$

where $q_i = +e$ for Na and Li ions and $\mathbf{r}_i$ is the position of the ith ion. It can be shown that, in solid crystalline electrolytes, when eq 3 is used to calculate the conductivity, the contributions associated with framework atoms vanish in the long time limit (see Appendix). Thus the summation in eq 3 can be restricted to the mobile charge carriers only.

Numerical Simulation Results

To calculate both the EH and Nernst–Einstein (NE) conductivities, we ran MD simulations for a total of over 100 ns for each temperature, constraint and material. The EH conductivity and Haven ratio are shown in Figure 1 and Figure 2, respectively. For each conductor, although the conductivities calculated using the EH relation differ substantially from those calculated using the NE approximation (Supplementary Fig.20), the dominant anion motion mode is the same whether the conductivity is evaluated using EH relation or NE approximation (Supplementary Fig.21–26). The anion dynamics facilitate cation diffusion, leading to higher conductivity. Specifically, anion rotation and translation govern ionic conduction in $Na_2B_{10}H_{10}$ and $LiBH_4$, respectively. In Na_3OBH_4, BH_4^- translation is the dominant anion motion mode affecting conductivity. In Li_3PS_4 and $Na_4P_2S_6$, vibration dominates, whereas in $Na_{11}Sn_2PS_{12}$, vibration has only a minor effect and rotation dominates. Note that Na vacancies are introduced into Na_3OBH_4 at a concentration of 1.17% to enable Na migration.

Redacted

Most of the Haven ratios shown in Figure 2 are smaller than 1, indicating the presence of interactions among charge carriers (*Solid State Ionics.* **7**, 177–198 (1982)) and the resulting cooperative motion (*Nat. Commun.* **8**, 15893 (2017)). For the three thiophosphates and Na_3OBH_4, the Haven ratio remains within a narrow range under all four constraint conditions and across the entire simulated temperature range. For $Na_2B_{10}H_{10}$ and $LiBH_4$, the Haven ratio shows weak temperature dependence, but tends to increase when anion rotation is constrained. This trend indicates that anion rotation enhances the cooperative inter-ionic correlations in $LiBH_4$ and $Na_2B_{10}H_{10}$, contributing to the high Li^+/Na^+ conductivity in

these materials. Overall, the three thiophosphates exhibit lower Haven ratios than the two borohydrides and the antiperovskite, indicating stronger interionic correlations.

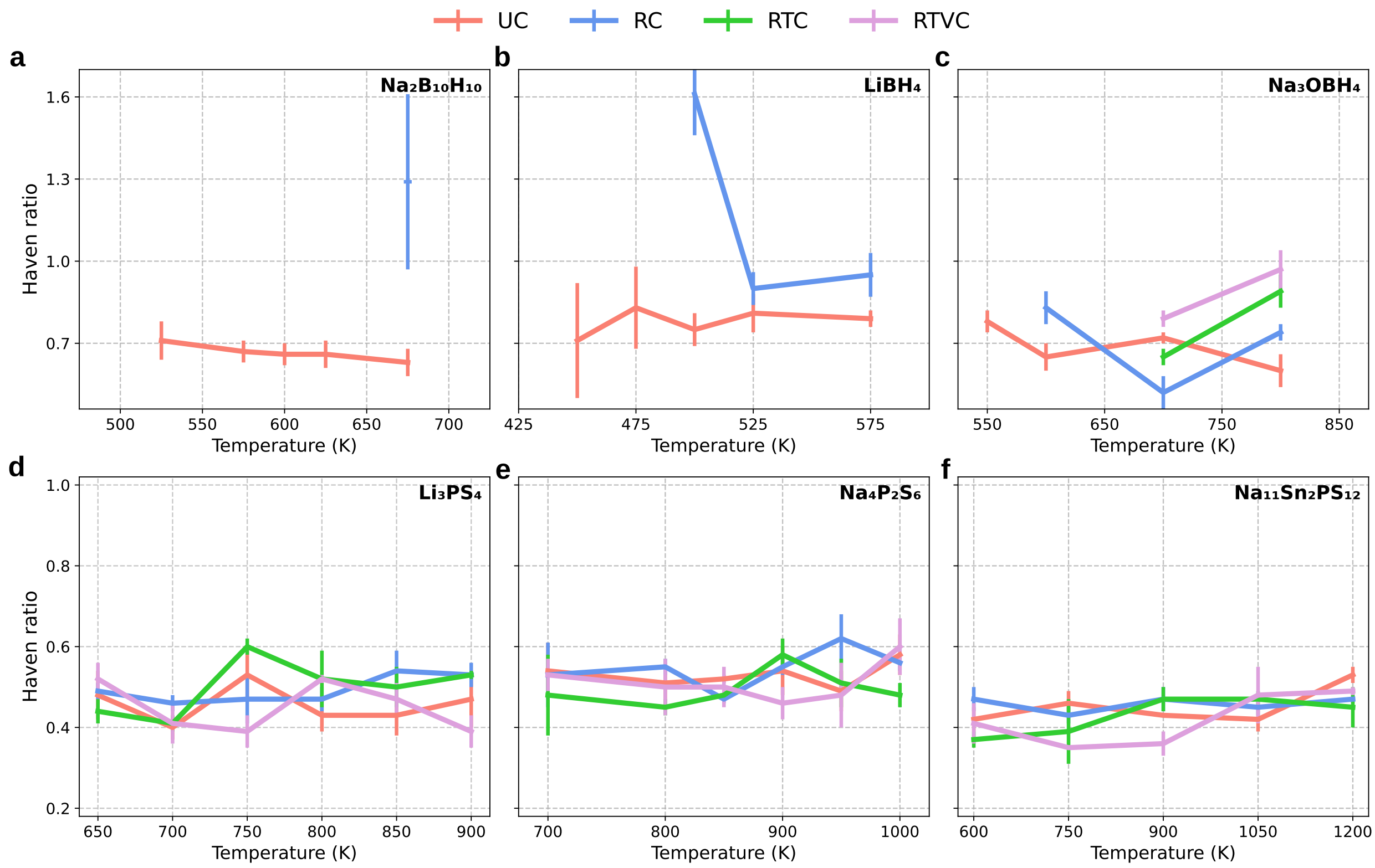

Figure 2. Temperature dependence of Haven ratio for six ionic conductors under four constrained conditions. Error bars computed from independent trajectories are displayed. This figure also appears as Fig.3 in the revised manuscript.

Manuscript revisions

The *Abstract* now mentions the key finding:

Change 1 (*Abstract*, lines 28–29)

- Added: "rotation can further enhance cooperative cation transport."

The *Introduction* now provides the relevant background:

Change 2 (*Introduction*, lines 120–126)

- Added: "The Nernst–Einstein (NE) equation, which is an approximation to the Green–Kubo formula and converges faster, is often employed to estimate conductivity. However, given the high concentration of charge carriers in superionic conductors, ion–ion correlations can play an important role[56–58] and leads to the failure of this approximation.[59] These correlations may themselves be coupled to anion dynamics, an aspect that has rarely been examined. Capturing them through the Green–Kubo formula, however, requires substantially longer simulations."

The *Introduction*'s closing summary now incorporates these results:

Change 3 (*Introduction*, lines 147–149)

- Added: "For each material, we performed MLMD simulations for more than 100 ns at each temperature, in which specific polyanion motion modes were selectively constrained, and evaluated the ionic conductivity using the Einstein–Helfand (EH) relation."

Change 4 (*Introduction*, lines 162–164)

- Added: "Haven ratio analysis further shows cooperative cation transport across all systems. In the borohydrides, this cooperative motion is reinforced by anion rotation."

The *Results* section now introduces the Einstein–Helfand formula:

Change 5 (*Results*, lines 229–233)

- Added: "To quantify the contribution of polyanion translation, rotation and vibration to cation transport in SEs, we calculate the ionic conductivity using the Einstein–Helfand formula (eq 1, hereafter the EH conductivity), which is equivalent to the Green–Kubo formula and captures ion–ion cross-correlations."

The conductivity figure now uses the Einstein–Helfand formula:

Change 6 (*Results*, Fig.2, content & caption)

- Original: Figure 3 showed conductivities calculated using the Nernst–Einstein approximation.
- Revised: (1) Fig.2 now shows conductivities calculated using the Einstein–Helfand formula; the caption is updated accordingly.

  (2) Added in the main text (lines 238–240): "At each temperature, more than 100 ns of MD trajectories were used to compute the EH conductivity, and the results are shown in Fig. 2a–f."

  (3) All numerical values cited in the main text are updated accordingly.

A new subsection discussing the effect of cation–anion dynamic coupling on the Haven ratio was added:

Change 7 (*Results*, new subsection, lines 267–281)

A new subsection, "Anion rotation enhances cooperative transport in borohydrides," is added, together with a new Fig. 3 presenting the Haven ratios.

The *Discussion* now covers cooperative cation transport:

Change 8 (*Discussion*, lines 607–610)

- Added: "Beyond this single-ion picture, all six conductors exhibit Haven ratios below unity, indicating that cooperative cation motion is common across plastic-crystal SEs. In the borohydrides, anion rotation reinforces this cooperative motion, suggesting another way in which rotation can enhance ionic conductivity."

The *Methods* now describe the Einstein-Helfand relation and Haven ratio:

Change 9 (*Methods*, lines 665–670 and 674; equation (2) and (5))

- Added: "The conductivity is calculated through the Einstein-Helfand relation:[102]

$$\sigma_{HE} = \frac{1}{3\Omega k_B T} \lim_{t\to\infty} \frac{\langle | \sum_i q_i(\mathbf{r}_i(t) - \mathbf{r}_i(0))|^2 \rangle}{2t}$$

  The Haven ratio is then defeined as $H_R = \frac{\sigma_{NE}}{\sigma_{EH}}$"

The *Methods* also specify the conductivity threshold applied to the EH relation:

Change 10 (*Methods*, lines 678–680)

- Added: "For the conductivity calculated from the EH relation, we also adopt $0.1\,\mathrm{mS\,cm^{-1}}$ as the lower threshold, based on a previous study that reported conductivities on the order of $0.1\,\mathrm{mS\,cm^{-1}}$.[40]"

Reviewer #1 (Remarks on code availability):

The code provided is largely a copy of the code CP2K, https://www.cp2k.org/. As such it works. Other materials, such as the trained models can be found at https://github.com/KeouLee/SE_MLIP, as stated in the Data Availability statement. Honestly, I would suggest avoiding the use of statements such as "Other source data are available from the corresponding authors upon request." in the Data Availability statement, since, in this way, reproducibility may be lost.

We thank the reviewer for the suggestion. Notification on the code changes have been added to the source code description in Code Ocean. A detailed log of these changes is available in data/changes.md within the Code Ocean capsule.

To improve reproducibility, in addition to the previously uploaded MLIP, we have now also uploaded the MSD and mean square dipole displacement data to https://github.com/KeouLee/Analysis/tree/main/data. Regarding code availability, in addition to the previously uploaded modified CP2K, we have also made available the custom code for trajectory analysis, rotor reorientation frequency calculations, CP2K metadynamics simulations, and VASP defect formation energy calculations at https://github.com/KeouLee/Analysis. We hope these additions further enhance the reproducibility of our work.

The manuscript has been updated accordingly:

Change (*Data availability*, lines 744–749)

- Original: "The data generated in this study are presented in the main article and its Supplementary information. Other source data are available from the corresponding authors upon request."
- Revised: "The MSD and mean square dipole displacement can be found at https://github.com/KeouLee/Analysis/tree/main/data. Source data underlying all figures in the manuscript and Supplementary Information are provided with this paper."

Added (*Code availability*, lines 751–753)

- Added: "Custom code for trajectory analysis, rotor reorientation frequency calculations, CP2K metadynamics simulations, and VASP defect formation energy calculations is available at https://github.com/KeouLee/Analysis."

Reviewer #3 (Remarks to the Author):

Understanding anion participation in the transport of active ions, lithium/sodium (Li/Na), in solid-state materials has been a great debate. Significant imprecise and far-fetched literature with claims of concerted anion motion, paddlewheel effects has been published in the last 40 years, with a resurgence in recent years with the "rediscovery" of ion conductivity in solid crystalline materials. The manuscript of Li and collaborators investigated the participation of anion motion in the lithium/sodium (Li/Na) ion motion in ionic conductor materials, also known as solid electrolytes. In my opinion, the most interesting part of this paper is how the motion of polyanion groups increases the local disorder, with the global outcome of flattening the potential energy surface. They used a combination of standard techniques, density functional theory, and machine learning potential. The work is technically sound, but with several imprecisions and paradoxical statements.

In summary, the topic is important. However, the analysis of the data is quite simplistic, and many assumptions are made. This study cannot be considered in its present state. Finally, these authors are not aware/up to date on the existing literature on this topic.

We sincerely thank the reviewer for carefully reading our manuscript and the constructive comments. We fully share the reviewer's view that the participation of anion motion in Li/Na transport is an important question, and we appreciate their clear explanation of its importance.

The reviewer has identified the connection between polyanion motion, cation dynamic disorder, and the flattening of potential energy surface as the most interesting aspect of our work. This is indeed one of the central physical pictures we aim to convey. On revisiting the manuscript, however, we recognized that our original analysis supporting this picture was not yet sufficiently developed, and the reviewer's emphasis on this mechanism prompted us to substantially extend the analysis. Specifically, we have employed metadynamics to quantify the changes in the cation migration energy barrier, and have additionally analyzed the Na occupational disorder. Together, these new analyses place the connection between polyanion motion, cation dynamic disorder, and energy-barrier flattening on a quantitative footing, rather than leaving it as a qualitative observation as in the original version.

We agree with the reviewer's concern regarding the imprecise and at times overstated claims that have appeared in the literature on concerted anion motion and paddlewheel effects. The present work was in fact motivated by precisely this concern: rather than presupposing a particular role for polyanion dynamics, we developed a constraint-based approach that isolates and quantifies the contribution of each motion mode (translation, rotation, vibration) separately, and our conclusions—including the finding that rotation does not universally dominate transport—follow directly from these quantitative results.

We acknowledge that the original manuscript contained imprecisions, included some statements that may have appeared inconsistent, and presented analyses that could be made more rigorous. We have also expanded our discussion of the relevant literature. These points are addressed in detail in the responses below, and we hope the revisions reflect our shared commitment to a quantitative and cautious treatment of this topic.

1. The language of the manuscript is very assertive and borderline overreaching. For example, in the abstract, they state, “Here, we present a pioneering study...”. What’s so pioneering about this study?

   We sincerely thank the reviewer for raising this point. We agree that “pioneering” was overstated. More broadly, we recognise that the original manuscript contained language that was both overly assertive in tone and, in places, overreaching relative to the evidence presented. We have carried out a manuscript-wide revision, addressing 28 such instances across the *Abstract*, *Introduction*, and *Results*. The changes are detailed below.

   Change 1 (*Abstract*, lines 9–15)

   - Original: “Here, we present a pioneering study ... to quantitatively reveal ...”
   - Revised: “Here, we quantify ...”

   Change 2 (*Abstract*, lines 3–9)

   - Original: “Understanding and quantifying the impact of polyanion dynamics on cations ... no experimental or computational method can directly extract this information”
   - Revised: “Polyanion dynamics ... their contribution to ionic conductivity remains unquantified”
   - Reason: “No method can” is incorrect. The revised sentence states the actual research gap: the contributions of local polyanion dynamics to conductivity remain unquantified.

   Change 3 (*Abstract*, lines 16–17)

   - Original: “..., strong couplings between translation/vibration and diffusion, were revealed for the first time.”
   - Revised: Deleted.
   - Reason: We agree this was overstated, particularly “for the first time”. On further review of the literature, we found that strong coupling between vibration and diffusion has in fact been reported previously (*Adv. Mater.* **34**, 2207411 (2022)). The novelty of our work lies not in identifying such coupling, but in quantifying its specific contribution to the ionic conductivity.

   Change 4 (*Abstract*, line 17)

   - Original: “Each drives superionicity, ...”
   - Revised: Deleted.
   - Reason: We agree this was overreaching. “Each drives superionicity” implied that translation, rotation and vibration can all give rise to superionic behaviour on its own. However, vibration modulates conductivity by at most $\sim$1.4 orders of magnitude, which is not enough to suppress superionic behaviour in the thiophosphates studied.

Change 5 (*Abstract*, lines 24–25 and 27–28)

- Original: "Analyses uncover anion motion modes drive cation migration by inducing disorder in the cation sublattice."
- Revised: "We find that rotation and translation each can enhance conductivity by over two orders of magnitude by inducing cation disorder"
- Reason: "drive" → "can enhance": "can" reflects a capability realized in suitable systems, rather than the universal effect implied by the original.

Change 6 (*Abstract*, lines 25–27 and 29–30; *Introduction*, lines 158–159 and 160–162)

- Original: "They introduce fluctuations in the underlying energy landscape, lowering the energy barrier, and alter cation dynamics, affecting the prefactor."
- Revised: (1) "polyanion rotation, translation, and vibration all modulate the migration barrier and prefactor."

  (2) "we find that anion dynamics modulate ionic conductivity through both the migration barrier, which has been the central focus in SE design, and the prefactor, which can also contribute comparably."
- Reason: The original sentence asserted two causal relationships: "fluctuations lower the energy barrier" and "altering cation dynamics affects the prefactor", without providing adequate evidence to support either. The revised wording states only that the three motion modes (anion dynamics) modulate the barrier and the prefactor.

Change 7 (*Introduction*, lines 113–117)

- Original: "While rotation attracts great attention, other degrees of freedom (DOF) (translation and vibration) are often overlooked and rarely examined for their impact on cation migration."
- Revised: "Moreover, this longstanding focus on rotation has overshadowed the potential contributions of polyanion translation and vibration, whose contributions to ionic conductivity have not been quantitatively addressed."
- Reason: The original wording was overly assertive, as vibration has been examined in prior work, e.g., *Adv. Mater.* **34**, 2207411 (2022). The revised wording narrows the statement to their quantitative contributions to ionic conductivity, which have not been fully addressed.

Change 8 (*Introduction*, lines 141, 157–158, and 164–166)

- Original: "unveil the mechanisms by which anion dynamics enable superionicity. ... anion dynamics can induce cation disorder."
- Revised: "In $LiBH_4$ and $Na_2B_{10}H_{10}$, anion dynamics are decisive for superionicity; BH_4^- translation and $B_{10}H_{10}^{2-}$ rotation each contribute over two orders of magnitude to the conductivity by inducing disorder within the Li and Na sublattices."
- Reason: The original text attributed superionicity to anion-induced cation disorder, but did not provide sufficient evidence for this mechanism in either system: for $Na_2B_{10}H_{10}$, no quantitative evidence for disorder was given; for $LiBH_4$, the data implied disorder but did not explicitly demonstrate it.
  During revision, site-occupancy analysis (Figure 1a) now provides quantitative evidence for Na disorder in $Na_2B_{10}H_{10}$, and a schematic illustration (Figure 1b) explicitly shows how BH_4 translation induces dynamic cation disorder in $LiBH_4$.

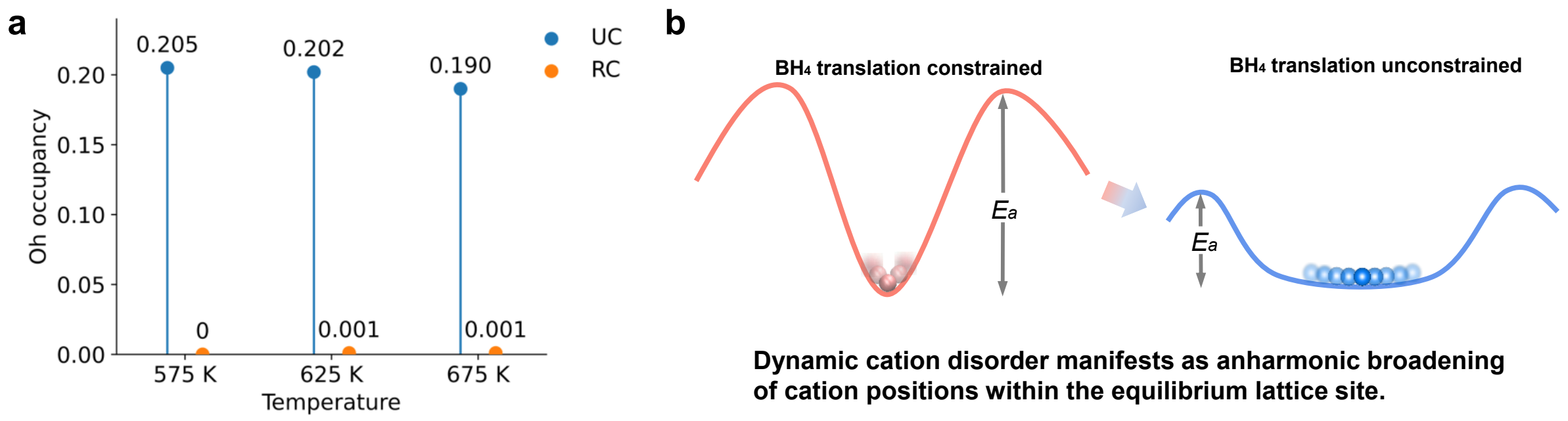

Figure 1. (a) Oh site occupancy of Na ions in $Na_2B_{10}H_{10}$ with $B_{10}H_{10}^{2-}$ unconstrained (UC) and rotationally constrained (RC) at various temperatures. (b) A schematic illustration shows how BH_4 translation modulates the energy landscape and induces dynamic cation disorder in $LiBH_4$. These figures also appear as Fig.6d and 6e in the revised manuscript.

Change 9 (*Introduction*, lines 151–155 and 167)

- Original: "Our work addresses longstanding controversies "
- Revised: "We quantify the contribution of the paddle-wheel effect to ionic conductivity and find it always operative. ... The paddle-wheel effect dominates conductivity only when the polyanion reorientation frequency matches the cation hopping frequency;"
- Reason: Removed overly assertive wording, specifically "addresses longstanding controversies". The conclusion regarding the paddle-wheel controversy is now directly specified.

Change 10 (*Results*, lines 205–210)

- Original: "For thiophosphate-type SEs, ... the conductivity generally stays above 1 mS cm^{-1}, even after applying RTVC. This suggests that the highly polarizable anion sublattice and the relatively weak bonding interactions between cations and anions are also important factors contributing to superionicity in this class of materials[4,66,68]"
- Revised: Deleted.
- Reason: This sentence attributed the remaining conductivity under roto-translational-vibrational constraint (RTVC) to high anion polarizability and weak cation–anion bonding without direct support. While these factors may be relevant according to previous studies, they were not evaluated in the present work, and the RTVC results alone do not establish them as the origin of the observed conductivity.

Change 11 (*Results*, lines 310–313)

- Original: "We observe that anions composed of the same elements with similar point-group symmetry exhibit comparable rotation frequencies, and their rotation frequencies show similar temperature-dependent susceptibility, regardless of variations in lattice symmetries and chemical environments, as illustrated in Figure 2e."
- Revised: Deleted.
- Reason: The original claims were not adequately supported by the previous evidence presented. The new analyses performed during revision further show that these claims are in fact incorrect, for the following specific reasons:

  (1) Reorientation frequencies can differ by more than an order of magnitude even for anions composed of the same elements with the same point-group symmetry; for example, PS_4^{3-} in $Na_{11}Sn_2PS_{12}$ versus Li_3PS_4 (Figure 2a).

  (2) "Similar point-group symmetry" is ill-defined as a classification criterion, making any generalization based on it prone to overreaching. For example, BH_4^- (T_d) and $B_{10}H_{10}^{2-}$ (D_{4d}) could be grouped as having similar point-group symmetry, yet their reorientation frequencies differ by ∼1.6 orders of magnitude (Figure 2a).

  (3) The newly fitted activation energies differ by ∼0.3 eV between Li_3PS_4 and $Na_4P_2S_6$ (Figure 2b), contradicting the claim of "similar temperature-dependent susceptibility" (i.e., similar slopes).

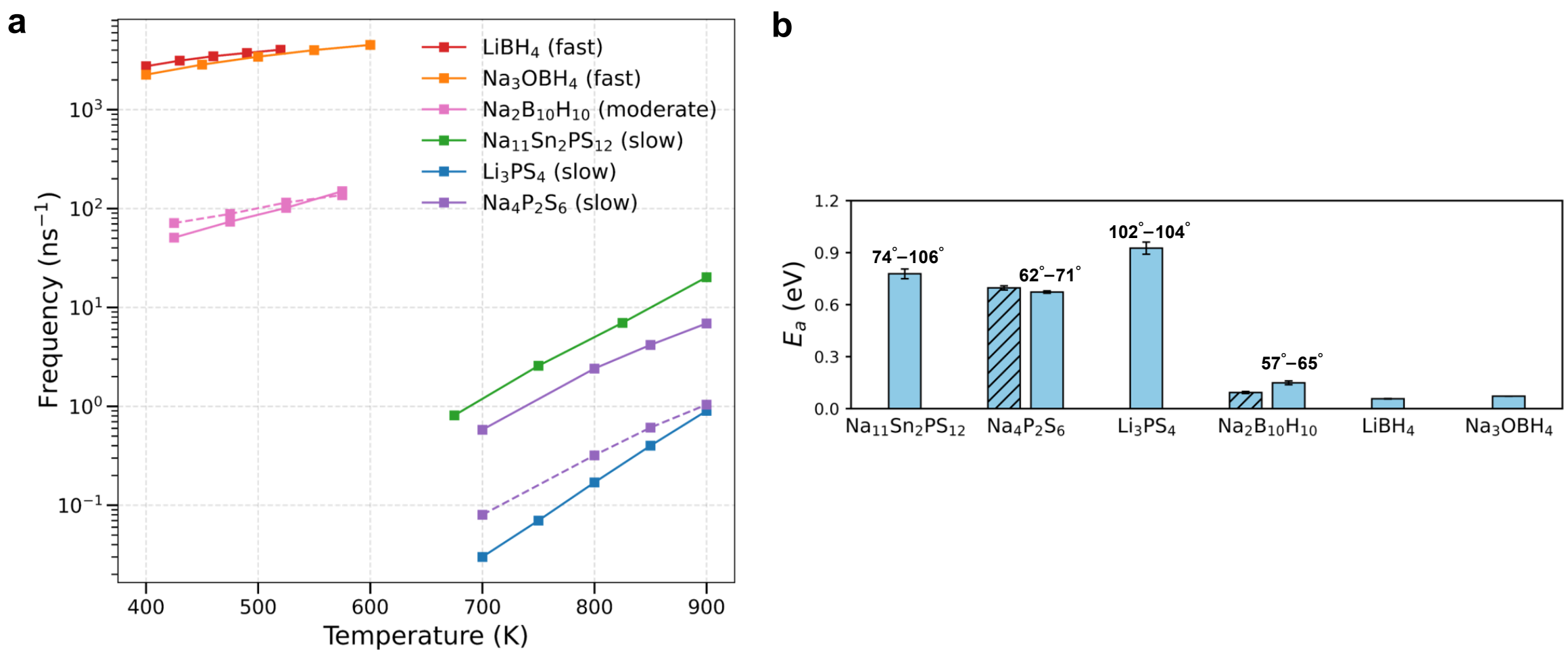

Figure 2. (a) Anion reorientation frequencies in the six SEs at various temperatures. For $Na_4P_2S_6$ and $Na_2B_{10}H_{10}$, solid lines correspond to reorientation of the line connecting the two P atoms in ${P_2S_6}^{4-}$ and the two apical B atoms in ${B_{10}H_{10}}^{2-}$, respectively. Dashed lines correspond to the internal rotation of ${P_2S_6}^{4-}$ and rotation about the line connecting the two apical B atoms, respectively. (b) Activation energies (E_a) of anion reorientation, obtained from Arrhenius fits of the reorientation frequencies in (a). For $Na_4P_2S_6$ and $Na_2B_{10}H_{10}$, two reorientation modes are shown. Solid bars correspond to reorientation of the principal axis (P–P in ${P_2S_6}^{4-}$, apical B–B in ${B_{10}H_{10}}^{2-}$); hatched bars correspond to internal rotation of ${P_2S_6}^{4-}$ and rotation of ${B_{10}H_{10}}^{2-}$ about this axis. Numbers above bars indicate fitted reorientation angle ranges. No values are reported for $LiBH_4$ and Na_3OBH_4, whose reorientations cannot be described as discrete jumps. These figures also appear as Fig.4d and 4e in the revised manuscript.

Change 12 (*Results*, lines 318–320)

- Original: "This difference likely arises from the larger moments of inertia of ${PS_4}^{3-}$ and ${P_2S_6}^{4-}$ compared to ${BH_4}^{-}$, as well as differences in interaction strength between these rotor anions and the cation framework."
- Revised: Deleted.
- Reason: The statement lacks quantitative support. While differences in moments of inertia and rotor-cation framework interactions are plausible factors, they are expected for different anion groups and do not, by themselves, add mechanistic insight.

Change 13 (*Results*, lines 320–323)

- Original: "The last type of rotor is ${B_{10}H_{10}}^{2-}$ with high point-group symmetry D_{4d}. Despite its large moment of inertia, the high intrinsic symmetry lowers the energy barrier for fast reorientational motion, resulting in a moderate rotation frequency."
- Revised: Deleted.

- Reason: (1) The statement was overreaching. It directly claimed that the high intrinsic symmetry of $B_{10}H_{10}{}^{2-}$ lowers the energy barrier, but no evidence was provided to support this conclusion.

  (2) In the original manuscript, the analyzed motion was the reorientation of the axis connecting the two apical B atoms, rather than rotation about this axis. Therefore, this motion is not directly related to the high intrinsic symmetry of the $B_{10}H_{10}{}^{2-}$ anion. Thus this statement is incorrect.

Change 14 (*Results*, lines 397–401)

- Original: "The results indicate that anion rotation (or libration) lowers the energy barrier, in line with previous proposals that anion rotation introduces fluctuations in the local potential energy landscape and reduces the effective energy barrier.[10,31,45] It can also be seen from phonon DOS that the timescales of local potential fluctuations from anion motion align with cation local vibration frequencies (Figure S17)."
- Revised: Deleted.
- Reason: The original text rests on an implied causal chain: certain anion motions share frequencies with cation vibrations → the cation therefore experiences potential fluctuations from these motions → these fluctuations reduce the migration barrier. The first two steps are plausible, but the last step does not follow from the preceding two and no evidence is provided to support it. The claim thus overreaches what the phonon DOS data can establish.

Change 15 (*Results*, line 412)

- Original: "Fitting the prefactor from MD simulations is not feasible due to significant errors."
- Revised: Deleted.
- Reason: This statement is overly assertive. The prefactor can be and has been fitted from MD simulations (*RSC Adv.*, **10**, 10715 (2020)). The actual difficulty is not in fitting the total prefactor but in decomposing it into its individual contributing terms.

Change 16 (*Results*, lines 424–427)

- Original: "Migration entropy also plays a crucial role, being related to the vibrational frequencies of configurations at both equilibrium and the saddle point. The presence of fast anion rotation can alter the local dynamics of the cation in two configurations, thereby influencing the entropy."
- Revised: Deleted.
- Reason: (1) We observed changes in phonon DOS and inferred changes in local cation dynamics for both equilibrium and saddle-point configurations. However, phonon DOS from MD trajectories predominantly reflects equilibrium dynamics, with saddle-point configurations only transiently sampled during migration events. It therefore cannot substantiate the claim for both configurations.

  (2) Changes in local cation dynamics do not directly imply changes in migration entropy without additional quantitative analysis.

Change 17 (*Results*, lines 448–449 and 495–496)

- Original: "When only the vibrations of BH_4^- are present, the Li^+ phonon DOS is hardly affected, indicating that the high-frequency optical modes decouple from Li^+ dynamics."
- Revised: "The minor change in the partial phonon DOS of Li from RTVC to RTC indicates that BH_4 internal vibrations do not substantially alter the partial phonon DOS of Li (Fig. 7a)."
- Reason: A minor change in the overall partial phonon DOS of Li does not demonstrate that BH_4 vibrations and Li dynamics are decoupled; it only shows that BH_4 vibrations do not substantially alter the partial phonon DOS of Li as a whole. The original conclusion overreached.

Change 18 (*Results*, lines 452–453)

- Original: "Anion translation can couple with certain modes of Li^+, which are essential for migration, thereby driving superionicity."
- Revised: Deleted.
- Reason: The original statement claimed coupling with Li modes "essential for migration" without identifying which modes are involved or demonstrating their relevance to migration.

Change 19 (*Results*, lines 453–458 and 497–500)

- Original: "In addition, damping can also result in a more diffuse phonon DOS due to broadening; this causes Li^+ to undergo less well-defined lattice vibrations and exhibit more liquid-like behavior, reminiscent of the breakdown of phonons involving the Cu sublattice in $CuCrSe_2$ during its superionic transition. This has also been observed in several other superionic conductors during their superionic transitions."
- Revised: "BH_4 translation ... making the DOS more diffuse and suppressing the sharp peaks. This change can in part be attributed to anharmonic effects."
- Reason: The original over-associated the diffuse partial phonon DOS of Li with damping, which cannot be concluded from the partial phonon DOS of Li alone and constitutes an overreach. The revised discussion instead attributes the diffuse features in part to anharmonic effects.

Change 20 (*Results*, lines 458–460 and 500–502)

- Original: "Meanwhile, anion translation directly triggers the emergence of low-frequency modes of cation motion (see red line in Figure 5a), leading to large atomic displacements and thereby contributing to superionicity."
- Revised: "Meanwhile, BH_4^- translation shifts the band center of the partial phonon DOS of Li^+ toward lower frequency and increases its low-frequency intensity, reflecting softened Li^+ dynamics that can facilitate large-amplitude local motion."

- Reason: (1) Large-amplitude local motion does not necessarily contribute to superionicity. Rather, the mode and migration pathway should match (*Energy Environ. Sci.* **14**, 6554 (2021)). The original conclusion overreached.

  (2) The original claim that anion rotation "triggers the emergence of low-frequency modes" overreaches, as increased low-frequency intensity can also result from broadening and/or shift rather than new modes. The revised text avoids this unsupported claim and instead states only what is directly observed: a band-center shift toward lower frequency and enhanced low-frequency intensity.

Change 21 (*Results*, lines 470–472 and 490–492)

- Original: "The absence of BH_4^- translation renders the split site highly energetically unfavorable, thereby blocking the migration pathway."
- Revised: "By contrast, further constraining BH_4 translation increases the barrier by $\sim$0.5 eV, demonstrating that BH_4 translation is critical for reducing the Li migration barrier."
- Reason: Without the metadynamics data, the split-site distribution alone could not support the original claim that the migration pathway is completely blocked. During revision we now provide the metadynamics results (Figure 3), which do show a significant barrier increase to 0.83 eV. Even with this large barrier, we still adopt a more measured conclusion that BH_4 translation is critical for Li transport, rather than claiming complete blockage.

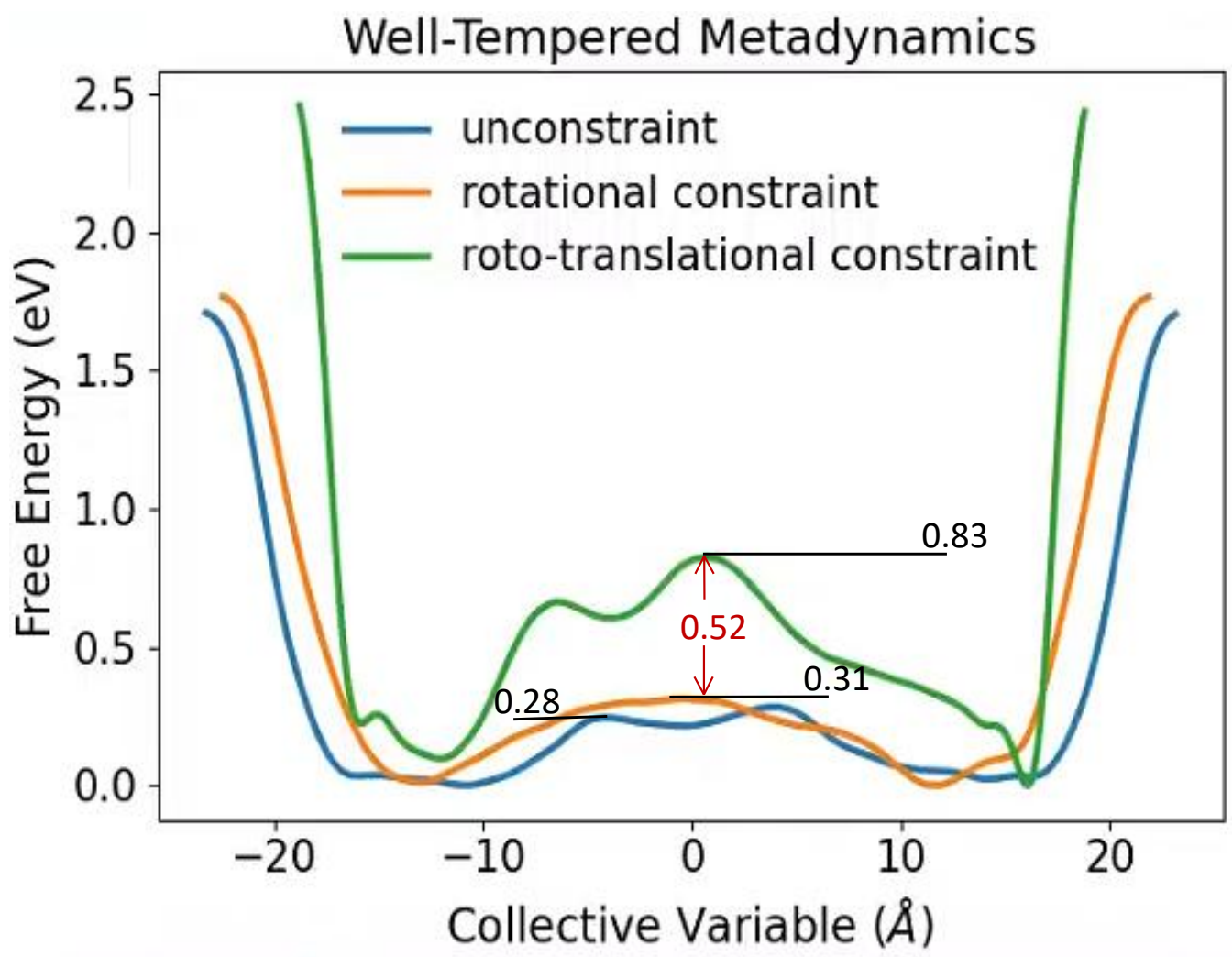

Figure 3. Free-energy profiles of Li^+ migration in $LiBH_4$ obtained from well-tempered metadynamics at 300 K under three constraint conditions: unconstrained (blue), rotationally constrained (orange), and roto-translationally constrained (green). The migration barrier increases from 0.28 eV in the unconstrained case to 0.31 eV under rotational constraint, and further to 0.83 eV when translation is also constrained, corresponding to an additional 0.52 eV barrier increase from translational constraint alone. This figure also appears as Fig.6c in the revised manuscript.

Change 22 (*Results*, lines 505–506 and 519–521)

- Original: "Notably, anion ($B_{10}H_{10}{}^{2-}$) rotation strongly affects phonon modes dominated by sodium ions and triggers the emergence of low-frequency modes of Na^+ motion."
- Revised: "anion rotation strongly modifies the partial phonon DOS of Na^+, making it more diffuse and giving rise to a low-frequency component (Fig. 7b)."
- Reason: The original claim that anion rotation "triggers the emergence of low-frequency modes" overreaches, as increased low-frequency intensity can also result from broadening and/or shift rather than new modes. The revised text avoids this unsupported claim and instead refers only to low-frequency "component."

Change 23 (*Results*, lines 508–512 and 517–519)

- Original: "The Na^+–Na^+ PDF (Figure 6d) and Na^+ probability densities under UC and RC (Figure 6e–f) suggest that anion rotation directly induces cation disorder, leading to the distribution of cations across both tetrahedral and octahedral sites with partial occupancy, thereby opening transport channels."
- Revised: "constraining the rotation of $B_{10}H_{10}{}^{2-}$ reduces the Oh occupancy by more than two orders of magnitude (to $\sim$0.1%). This reveals that anion reorientational disorder directly induces cation disorder, thereby enabling superionicity."
- Reason: The original Na^+–Na^+ PDF and probability density plots were open to interpretation and could serve as auxiliary support for this conclusion at most. "Opening transport channels" further overreached what these data could support. The revised text is based on the quantitative Oh site occupancy analysis (Figure 1a), which directly demonstrates that $B_{10}H_{10}{}^{2-}$ rotation induces Na^+ sublattice disorder.

Change 24 (*Results*, lines 523–526 and 546–547)

- Original: "Strikingly, $PS_4{}^{3-}$ vibrations induce disorder within Li^+ sublattice and cause Li^+ phonon DOS to become diffuse, with the disorder evidenced by the probability densities of Li ions distributing over multiple partial sites (Figure 6h–i) and by the broadening of peaks in the Li^+–Li^+ PDF from RTC to RTVC (Figure 6g)."
- Revised: "$PS_4{}^{3-}$ dynamics further shift the band center toward lower frequency."
- Reason: (1) It overstated the role of $PS_4{}^{3-}$ vibrations by saying they "induce" disorder, because the evidence (PDF & probability densities of Li) is not sufficient to support direct induction of cation disorder in the same sense as the occupancy analysis in Figure 1a.

  (2) No quantitative evidence supports "it becomes diffuse". The revised text only states the lowering of the band center of the partial phonon DOS of Li.

  (3) The original wording was too subjective, especially the use of "Strikingly."

Change 25 (***Results*, lines 533–535**)

- Original: "For the different types of SEs studied here, cation dynamics are strongly coupled only with certain key anion motion modes, which can be identified through selective constraint based on our algorithm."
- Revised: Deleted.
- Reason: A large overall change in the partial phonon DOS of cation upon constraining a given anion motion mode can indicate strong coupling, but a small overall change does not rule out strong coupling. The claim that cation dynamics are strongly coupled "only" with certain modes is therefore not supported by the partial phonon DOS of cation alone.

Change 26 (***Results*, lines 536–537**)

- Original: "The cation phonon DOS undergoes little change when the other modes are constrained, aligning with the conductivity trend in Figure 3."
- Revised: Deleted.
- Reason: Avoid over-associating weak response of partial phonon DOS of cation with weak conductivity influence, since small changes of partial phonon DOS of cation do not necessarily demonstrate weak transport effects.

Change 27 (***Results*, lines 537–541**)

- Original: "From a lattice wave perspective, phonon mode coupling is crucial for cation migration.[71] The constraint on key local anion modes directly affects the phonon modes they dominantly participate in, subsequently influencing cation vibration through coupling. We successfully identify these key local anion modes and quantify their impact on conductivity."
- Revised: Deleted.
- Reason: Establishing a link between local anion modes and cation migration would require showing that these local modes project substantially onto phonon modes aligned with the migration pathway (*Energy Environ. Sci.* **14**, 6554 (2021)). No such analysis was provided, and this is beyond the scope of the present study.

2. One crucial aspect that is never discussed in this study is whether they introduce lithium (sodium) vacancies in the 6 solid electrolytes investigated, or whether they believe that interstitials will provide sufficient Li/Na-ion transport. Furthermore, the vacancy concentration features directly in Eq. 2 in the manuscript.

We thank the reviewer for pointing this out and agree that our original manuscript did not sufficiently address this aspect. This comment prompted us to revisit the role of defects in ion conduction for every system in the study, and in particular motivated a dedicated investigation of defect physics for Na_3OBH_4 that we believe has meaningfully improved the manuscript. We address each of the six electrolytes below.

$Na_{11}Sn_2PS_{12}$

It is not necessary to introduce extra Na vacancies because the sodium ions occupy six distinct sites, three of which are almost fully occupied with the others being partially occupied and providing the all-important cross-over points that enable fast ion conduction. (*Energy Environ. Sci.* **11**, 87–93 (2018); *Chem. Mater.* **30**, 7413–7417 (2018))

Furthermore, an AIMD study suggests that the Na6 site, which has the lowest occupancy among the six, can significantly contribute to Na-ion transport upon the onset of ${PS_4}^{3-}$ rotation (*Mater. Adv.* **1**, 184–196 (2020)).

$Na_2B_{10}H_{10}$

Three partially occupied Na sites have been reported in $Na_2B_{10}H_{10}$, which provide available sites for Na-ion migration (*Adv. Mater.* **26**, 7622–7626 (2014)). Therefore, no extra Na vacancies were introduced.

$\gamma-Na_4P_2S_6$

Two partially occupied Na sites have been reported in γ-$Na_4P_2S_6$, which provide available sites for Na-ion migration (*ACS Energy Lett.* **7**, 1403–1411 (2022)). Therefore, no extra Na vacancies were introduced.

$\alpha-Li_3PS_4$

The Li-ion transport in α-Li_3PS_4 can be related to the availability of three partially occupied Li Wyckoff sites (*J. Mater. Chem. A* **8**, 12446–12456 (2020)). Therefore, no extra Li vacancies were introduced.

$LiBH_4$

The intrinsic defects involved in Li-ion migration are Frenkel pairs. This has been indicated by their low formation energy in previous studies.
(*Phys. Rev. B* **83**, 144301 (2011); *J. Phys. Chem. C* **116**, 13488–13496 (2012))

Therefore, no Li vacancies were introduced.

Na_3OBH_4

For Na_3OBH_4, no successful Na hops were observed in the defect-free model in our previous simulations. We therefore performed a defect physics investigation (Figure 4), which indicates that Na vacancies are the dominant native defects governing ion diffusion. We introduced V_{Na} into the Na_3OBH_4 model, extended the machine learning interatomic potential to the defect-containing system, and verified its convergence and accuracy. We then calculated the ionic conductivity of the defect-containing system and quantified the contributions of different anion motion modes. (Figure 5).

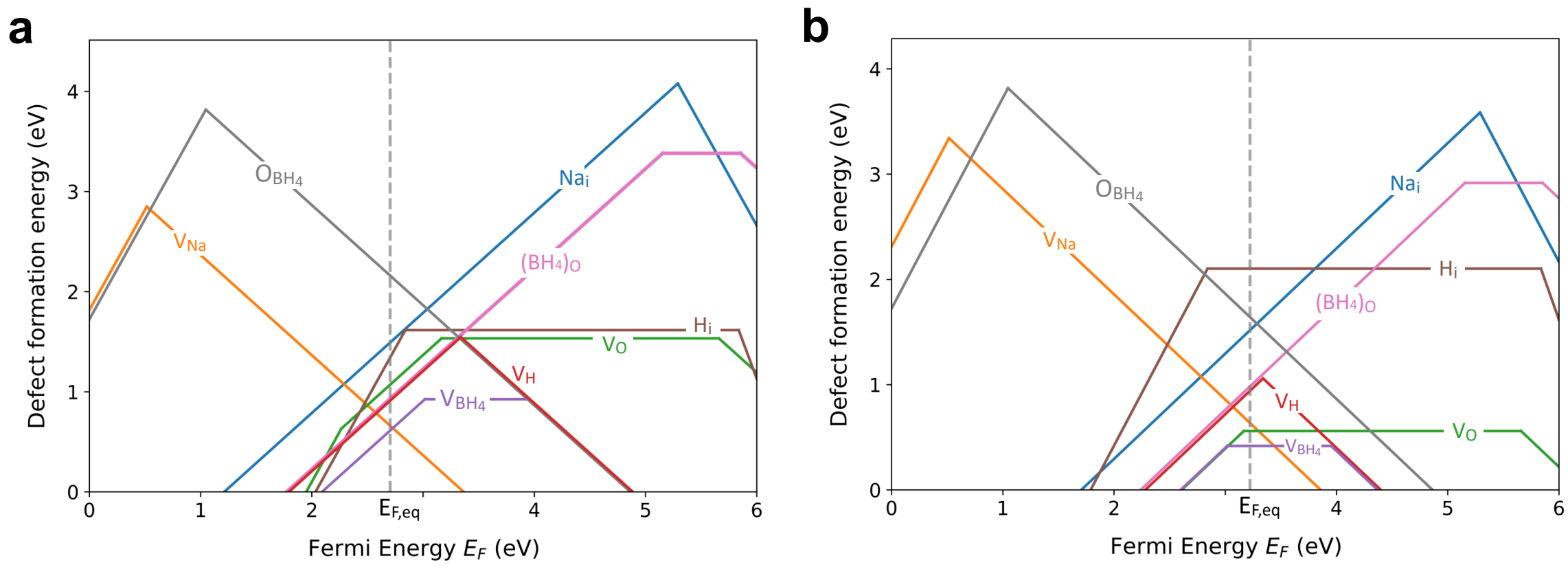

Figure 4: Formation energy ($\Delta E_{D,q}$) of native defects as a function of Fermi energy (E_F) in Na_3OBH_4 under (a) most Na-poor and (b) most Na-rich conditions. The equilibrium $E_{F,eq}$ is calculated at the synthesis temperature of 523 K and marked by vertical dashed lines. These figures also appear as Supplementary Fig.6 in the revised Supplementary Information.

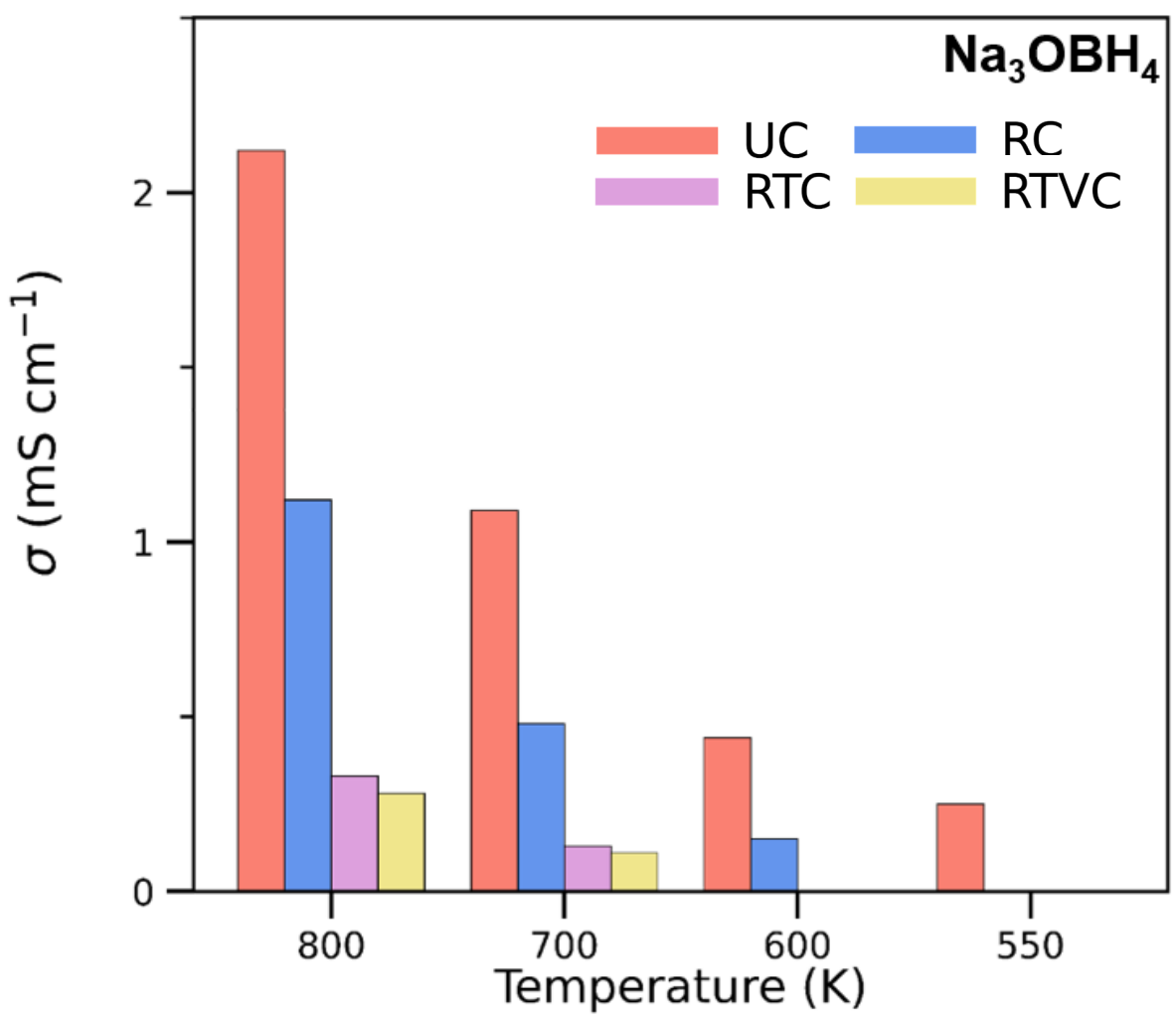

Figure 5: Conductivity of Na_3OBH_4, calculated using the Einstein–Helfand formula under four constrained conditions at various temperatures. Bars are absent where no sufficient Na-ion diffusion was observed within the simulation time. UC, RC, RTC, and RTVC denote unconstrained, rotationally constrained, roto-translationally constrained, and roto-translationally-vibrationally constrained, respectively. This figure also appears as Fig.2c in the revised manuscript.

With the vacancy mechanism confirmed, conductivity of Na_3OBH_4 can now be calculated, and the corresponding results and methods are added in the revised manuscript:

Change 1 (*Results*, Fig.2, content & caption)

- Original: No conductivity avaliable for Na_3OBH_4 in original Figure 3
- Revised: (1) Fig.2c now shows conductivities calculated of Na_3OBH_4 using the Einstein–Helfand formula; the caption is updated accordingly.

  (2) Added in the main text (lines 255–256): "For Na_3OBH_4, BH_4^- translation dominates Na^+ transport (∼0.6 orders of magnitude)."

Change 2 (*Results*, Fig.4f, content)

- Original: No Na^+ hop frequency was reported for Na_3OBH_4 in original Figure 2f.
- Revised: A Na^+ hop frequency for Na_3OBH_4 is now obtained and added to Fig. 4f.

Change 3 (*Results*, lines 336–341, 367–371)

- Original: Interestingly, one of the simulated electrolytes, Na_3OBH_4, with the same fast BH_4^- rotors constituting the sublattice, showed no successful Na^+ hops after simulation at 800 K for 10 ns within a 1125-atom box (see the mean square displacment (MSD) result in Figure S8), contradicting experimental findings.[11] Ahiavi et al.[76] and Tsai et al.[15] suggest that the high conductivity cannot be reproduced neither by experiment nor MD simulation.
- Revised: For Na_3OBH_4, defect formation energies are first calculated to identify the dominant defects (Supplementary Fig. 6). Under most Na-poor conditions, V_{Na} and V_{BH4} are energetically favored, while Na_i has much higher formation energy and is excluded. We thus introduce Na vacancies. Detailed results about defect physics are provided in Section 2 in Supplementary Information.

Change 4 (*Methods*, new subsection, lines 708–742)

- Added: A new subsection describing the computational details of defect physics of Na_3OBH_4 is added.

Change 5 (*Supplementary Information*, new subsection, pages 6–7)

- Added: A new subsection on the defect physics of Na_3OBH_4 is added.

3. I am very curious about their method for freezing vibrational degrees of freedom. This is an integral part of the manuscript, but they don't make any effort to describe this in any detail.

We thank the reviewer very much for this comment. The contribution of vibrations is quantified by comparing two simulations: one in which the translational and rotational degrees of freedom of each rotor anion are constrained, and one in which the positions of all atoms within each rotor anion are fixed at their initial configuration throughout the simulation. The numerical implementation of the constraint algorithm has been described in detail in our previous publication (*J. Chem. Phys.* **161**, 054101, (2024)), which was cited in the *Introduction*.

For clarity, we briefly summarize here the essential construction of the constraint scheme used in the present simulations.

For each rotor anion, the atomic positions were expressed in the center-of-mass (COM) frame. The rotational constraint was defined with respect to a reference geometry of the rotor anion, represented by the reference positions $\mathbf{r}_a^0$ of all atoms in the COM frame. The positional rotational constraint is

$$\boldsymbol{\chi}^{(r)} = \sum_{a=1}^{n_a} \mathbf{r}_a^0 \times m_a \mathbf{r}_a(t) = \mathbf{0},$$

where a indexes atoms in the constrained rotor anion, m_a is the atomic mass, and $\mathbf{r}_a(t)$ is the instantaneous atomic position in the COM frame. Here, $\mathbf{r}_a^0$ is the reference position of atom a in the reference geometry.

Using the velocity Verlet integrator, the constrained position update should satisfy

$$\sum_{a=1}^{n_a} \mathbf{r}_a^0 \times \left[m_a \mathbf{r}'_a(t+\delta t) + \frac{1}{2}\delta t^2 \mathbf{g}_a^{(r)}(t) \right] = \mathbf{0},$$

where $\mathbf{r}'_a(t+\delta t)$ is the unconstrained predicted position and $\mathbf{g}_a^{(r)}(t)$ is the positional constraint force. The constraint force is expressed using Lagrange multipliers as

$$\mathbf{g}_a^{(r)} = -\sum_{i=1}^{n_r} \lambda_i^{(r)} \nabla_{\mathbf{r}_a} \chi_i^{(r)},$$

with $n_r = 3$ for the rotational constraint of a nonlinear rotor. After evaluating the gradient, the Cartesian components of $\mathbf{g}_a^{(r)}$ become linear functions of the three Lagrange multipliers, with coefficients determined only by the atomic masses and the reference positions of the atoms. Substituting these force components back into the rotational constraint equation gives a 3×3 linear system for $\lambda_1^{(r)}$, $\lambda_2^{(r)}$, and $\lambda_3^{(r)}$. Once the Lagrange multipliers are obtained, the corresponding constraint forces are substituted back into the velocity Verlet to update the constrained positions.

The velocity constraint part is imposed in the same manner by applying the corresponding constraint condition to the velocity update, which gives another small linear system for the velocity Lagrange multipliers. In the rotationally constrained (RC) simulations, this procedure removes the three rotational degrees of freedom of each rotor anion while allowing its COM translation and internal vibration. In the roto-translationally constrained (RTC) simulations, the rotational constraints are combined with COM translational constraints, so that the three translational and three rotational degrees of freedom of each

rotor anion are constrained while internal vibrations remain active. Finally, in the roto-translational-vibrationally constrained (RTVC) simulations, the Cartesian positions of all atoms within each rotor anion were kept fixed at their initial values throughout the trajectory, thereby suppressing translational, rotational, and internal vibrational motions of the rotor anion.

We agree that the original methods description was too brief. With this clarification in mind, we have expanded the *Methods* section to explicitly cite the constraint algorithm reference and to clarify the description of each constraint condition:

Change (*Methods*, lines 653–657)

- Added: "The selective constraints on rotor anion motion modes were implemented following the constraint algorithm developed in our previous work.[72] In RC and RTC simulations, we explicitly constrain each rotor anion's 3 rotational degrees of freedom (DOF) and 3 translational+3 rotational DOF, respectively. In RTVC simulations, we constrain all DOF of each rotor anion."

The corresponding description in the Results section has been removed to avoid redundancy (lines 298–300).

4. I am pretty disappointed that they don't even attempt or define "correlated" center of mass diffusivity. Still, instead, they just used the tracer diffusivity in Eq. 1. Tracer diffusivity is the simplest definition of diffusivity and doesn't account for any correlation. These authors should revise some essential basics of ion transport in solids, e.g. 10.1103/PhysRevB.64.184307, or more recently 10.1021/acsmaterialsau.2c00057

We thank the reviewer for raising this important methodological point and for pointing us to two valuable references. We have added the two references:

Added citation (*Introduction*, lines 121–123):

"However, given the high concentration of charge carriers in superionic conductors, ion–ion correlations can play an important role[56,57,58] and lead to the failure of this approximation.[59]"

We agree that the conductivity calculated using Nernst–Einstein (NE) relation in our previous manuscript is based on the tracer diffusivity and does not include cross-correlations between mobile ions. The conductivity should instead be evaluated from the "correlated" center of mass diffusivity, which includes both self and cross terms and thereby captures ion–ion correlations. This distinction was neither recognized nor addressed in the original manuscript.

To capture these ion–ion correlations directly at the level of ionic conductivity, we have recomputed the conductivity using the Einstein–Helfand (EH) relation, which is equivalent to the Green–Kubo relation

$$\sigma = \frac{1}{3\Omega k_B T} \lim_{t\to\infty} \frac{\langle | \sum_i q_i(\mathbf{r}_i(t) - \mathbf{r}_i(0))|^2 \rangle}{2t}$$

where $q_i = +e$ for Na and Li ions and $\mathbf{r}_i$ is the position of the ith ion. It can be shown that, in solid crystalline electrolytes, when this equation is used to calculate the conductivity, the contributions associated with (nondiffusive) framework atoms vanish in the long time limit (see Appendix). Thus the summation can be restricted to the mobile charge carriers only.

To calculate both the EH and Nernst–Einstein (NE) conductivities, we ran MD simulations for a total of over 100 ns for each temperature and constraint. The EH conductivity and Haven ratio are shown in Figure 6 and Figure 7, respectively.

For each conductor, although the conductivities calculated using the EH relation differ substantially from those calculated using the NE approximation (Supplementary Fig.20), the dominant anion motion mode is the same whether the conductivity is evaluated using EH relation or NE approximation (Supplementary Fig.21–26). The anion dynamics facilitate cation diffusion, leading to higher conductivity. Specifically, anion rotation and translation dominate ionic conduction in $Na_2B_{10}H_{10}$ and $LiBH_4$, respectively. In Na_3OBH_4, $BH_4{}^-$ translation is the dominant anion motion mode affecting conductivity. In Li_3PS_4 and $Na_4P_2S_6$, vibration has a greater effect on ion migration than rotation and translation, whereas in $Na_{11}Sn_2PS_{12}$, vibration has only a minor effect and rotation contributes somewhat more to the conductivity. Note that Na vacancies are introduced into Na_3OBH_4 at a concentration of 1.17% to enable ion conduction.

Figure 6. Conductivities of six materials calculated using the EH relation at various temperatures under four constrained conditions: (a) $Na_2B_{10}H_{10}$, (b) $LiBH_4$, (c) Na_3OBH_4, (d) α-Li_3PS_4, (e) γ-$Na_4P_2S_6$, and (f) $Na_{11}Sn_2PS_{12}$. Some conductivities are absent because the ions do not exhibit sufficient collective diffusion to yield a valid linear fit. This figure also appears as Fig.2 in the revised manuscript.

Most of the Haven ratios shown in Figure 7 are smaller than 1, indicating the presence of interactions among charge carriers (*Solid State Ionics* **7**, 177–198, (1982)) and the resulting concerted motion (*Nat. Commun.* **8**, 15893, (2017)). For the three thiophosphates and Na_3OBH_4, the Haven ratio remains within a narrow range under all four constraint conditions and across the entire simulated temperature range. For $Na_2B_{10}H_{10}$ and $LiBH_4$, the Haven ratio shows weak temperature dependence, but tends to increase when anion rotation is constrained. This trend suggests that anion rotation may strengthen interionic correlations in these materials. Rotation affects not only the ionic conductivity itself, but also the relative contributions of the self and cross terms in equation (1). Overall, the three thiophosphates exhibit lower Haven ratios than the two borohydrides and the antiperovskite, indicating stronger interionic correlations.

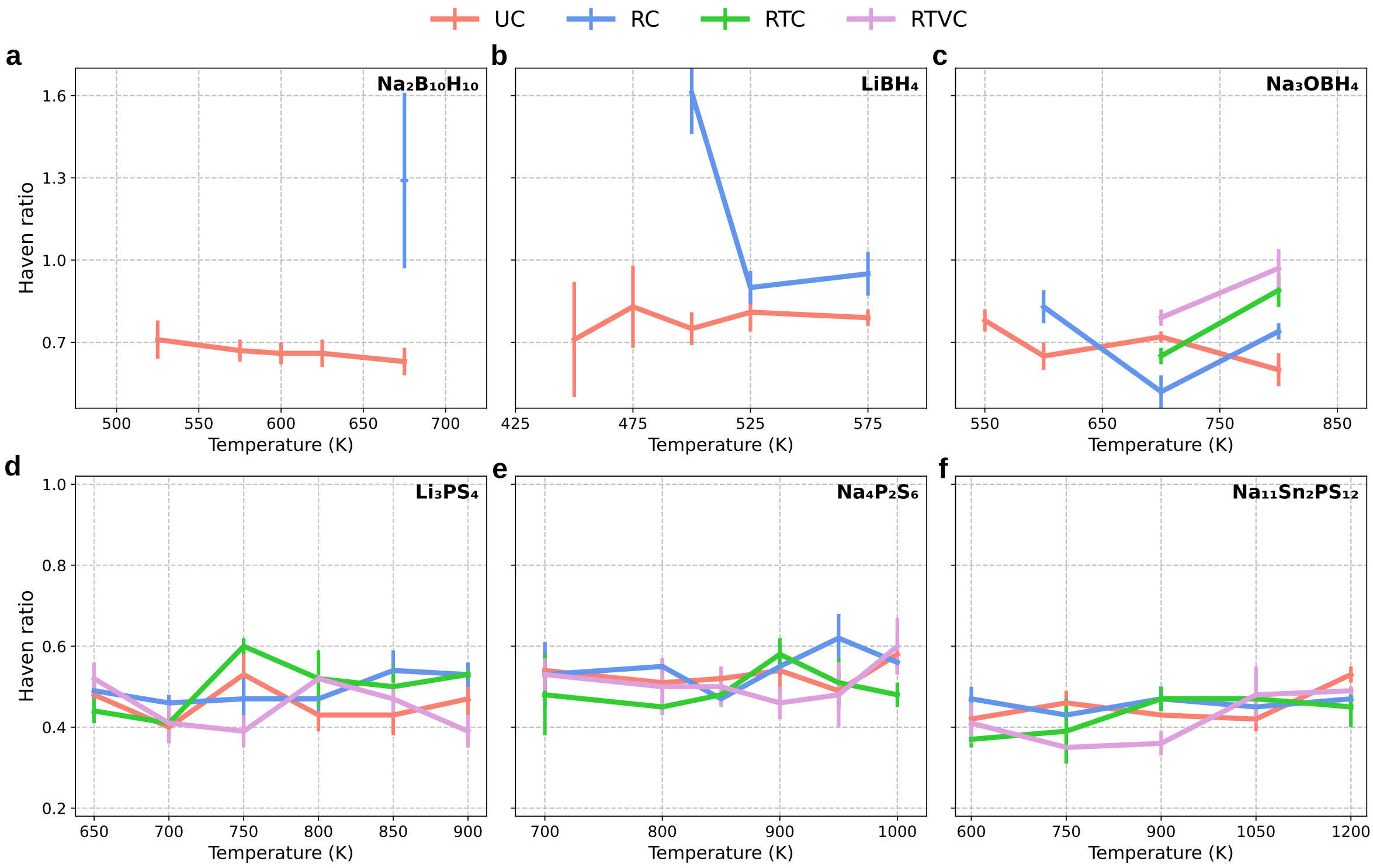

Figure 7. Temperature dependence of Haven ratio for six ionic conductors under four constrained conditions. Error bars computed from independent trajectories are displayed. This figure also appears as Fig.3 in the revised manuscript.

We thank the reviewer once again for raising this point, as addressing it has substantially strengthened the manuscript. The manuscript has been revised accordingly:

The *Abstract* now mentions the key finding:

Change 1 (*Abstract*, lines 28–29)

- Added: "rotation can further enhance cooperative cation transport."

The *Introduction* now provides the relevant background:

Change 2 (*Introduction*, lines 120–126)

- Added: "The Nernst–Einstein (NE) equation, which is an approximation to the Green–Kubo formula and converges faster, is often employed to estimate conductivity. However, given the high concentration of charge carriers in superionic conductors, ion–ion correlations can play an important role[56–58] and leads to the failure of this approximation.[59] These correlations may themselves be coupled to anion dynamics, an aspect that has rarely been examined. Capturing them through the Green–Kubo formula, however, requires substantially longer simulations."

The *Introduction*'s closing summary now incorporates these results:

Change 3 (*Introduction*, lines 147–149)

- Added: "For each material, we performed MLMD simulations for more than 100 ns at each temperature, in which specific polyanion motion modes were selectively constrained, and evaluated the ionic conductivity using the Einstein–Helfand (EH) relation."

Change 4 (*Introduction*, lines 162–164)

- Added: "Haven ratio analysis further shows cooperative cation transport across all systems. In the borohydrides, this cooperative motion is reinforced by anion rotation."

The *Results* section now introduces the Einstein–Helfand formula:

Change 5 (*Results*, lines 229–233)

- Added: "To quantify the contribution of polyanion translation, rotation and vibration to cation transport in SEs, we calculate the ionic conductivity using the Einstein–Helfand formula (eq 1, hereafter the EH conductivity), which is equivalent to the Green–Kubo formula and captures ion–ion cross-correlations."

The conductivity figure now uses the Einstein–Helfand formula:

Change 6 (*Results*, Fig.2, content & caption)

- Original: Figure 3 showed conductivities calculated using the Nernst–Einstein approximation.
- Revised: (1) Fig.2 now shows conductivities calculated using the Einstein–Helfand formula; the caption is updated accordingly.

  (2) Added in the main text (lines 238–240): "At each temperature, more than 100 ns of MD trajectories were used to compute the EH conductivity, and the results are shown in Fig. 2a–f."

  (3) All numerical values cited in the main text are updated accordingly.

A new subsection discussing the effect of cation–anion dynamic coupling on the Haven ratio was added:

Change 7 (*Results*, new subsection, lines 267–281)

A new subsection, "Anion rotation enhances cooperative transport in borohydrides," is added, together with a new Fig. 3 presenting the Haven ratios.

The *Discussion* now covers cooperative cation transport:

Change 8 (*Discussion*, lines 607–610)

- Added: "Beyond this single-ion picture, all six conductors exhibit Haven ratios below unity, indicating that cooperative cation motion is common across plastic-crystal SEs. In the borohydrides, anion rotation reinforces this cooperative motion, suggesting another way in which rotation can enhance ionic conductivity."

The *Methods* now describe the Einstein-Helfand relation and Haven ratio:

Change 9 (*Methods*, lines 665–670 and 674; equation (2) and (5))

- Added: "The conductivity is calculated through the Einstein-Helfand relation:[102]

$$\sigma_{HE} = \frac{1}{3\Omega k_B T} \lim_{t\to\infty} \frac{\langle | \sum_i q_i(\mathbf{r}_i(t) - \mathbf{r}_i(0))|^2\rangle}{2t}$$

  The Haven ratio is then defeined as $H_R = \frac{\sigma_{NE}}{\sigma_{EH}}$"

The *Methods* also specify the conductivity threshold applied to the EH relation:

Change 10 (*Methods*, lines 678–680)

- Added: "For the conductivity calculated from the EH relation, we also adopt 0.1 mS cm^{-1} as the lower threshold, based on a previous study that reported conductivities on the order of 0.1 mS cm^{-1}.[40]"

5. In the intro, they claimed they are doing "ultralong-time" simulations. This is very speculative and incorrect. First of all, their simulations fall short if compared to any classical molecular dynamics simulations. Therefore, they're unable to capture any correlation properties and even some basic ion transport properties. For example, they didn't report an Arrhenius plot in Figure 3f for LiBH4, because they could not converge their MSDs for this system. Kinetic Monte Carlo simulation as in 10.1038/s41467-022-32190-7 or 10.1098/rsos.170824, are the only methods that can reach "ultra-long" and spatially adequate simulations in the millisecond regime. These authors should comment on these publications as gold standards for ion transport.

We thank the reviewer for these careful and constructive comments. Each of the reviewer's points is addressed in detail below.

(1) We thank the reviewer for highlighting these two important studies employing kMC. We have cited them in the manuscript:

Added citation 1 (*Introduction*, lines 126–128):
"Significantly longer timescales, even in the millisecond range, can be accessed by employing kinetic Monte Carlo coupled with local cluster expansion; this approach can establish a robust link between experimental and theoretical diffusion results.[5,60]"

Furthermore, the *Nat. Commun.* study by Deng et al. is a notable example of how kMC can help address the long-standing problem of polyanion mixing in solid electrolytes. This work is also cited in:

Added citation 2 (*Introduction*, lines 41–43):
"From a static perspective, thermally activated cation hops occur by overcoming the energy barrier defined by the rigid host lattice; specific anion packing[4], polyanion mixing[5,6] and site disorder[7] can facilitate cation diffusion."

(2) We fully agree that the phrasing 'ultralong-time' overstated the actual capability of our simulations. Machine learning molecular dynamics (MLMD) (<100,000 atoms & ~10s of ns) are indeed shorter than those routinely achieved with classical MD (<1M atoms & ~100s of ns), and several orders of magnitude shorter than those accessible to kMC (>1M atoms & 10s of ms), as summarised in the review by Canepa (*ACS Mater. Au* **3** (2), 75–82 (2023)). We have therefore removed 'ultra-long' throughout the manuscript:

Remove 1 (*Introduction*, lines 145–148):
Original: "We performed ultralong-time, large-scale machine learning molecular dynamics (MLMD) simulations extensively to access results at near room and elevated temperatures."
Revised: "For each material, we performed MLMD simulations for more than 100 ns at each temperature, ..."

Remove 2 (*Discussion*, line 567–569):
Original: "Ultralong-time MLMD simulations were conducted for $Na_2B_{10}H_{10}$, $LiBH_4$, Na_3OBH_4, α-Li_3PS_4, γ-$Na_4P_2S_6$, and $Na_{11}Sn_2PS_{12}$ at near room temperature and elevated temperature."
Revised: Deleted.

(3) We respectfully take a different view on the reviewer's statement that our simulations are "unable to capture any correlation properties." Correlations can be included in equilibrium molecular dynamics through the Green–Kubo formula, or, equivalently, the Einstein–Helfand formula. Marcolongo and Marzari, for example, quantified such correlations in LGPS from first-principles molecular dynamics trajectories of only ∼500 ps, obtaining a Haven ratio deviating from unity (*Phys. Rev. Mater.* **1**, 025402 (2017)). More recently, Gigli et al. employed machine learning molecular dynamics of only a few nanoseconds (2–6 ns) on the α, β, and γ polymorphs of Li_3PS_4, using the Green–Kubo formula to evaluate ionic conductivities and Haven ratios (*Chem. Mater.* **36**, 1482–1496 (2024)). Notably, α-Li_3PS_4 is one of the systems also investigated in the present work.

Motivated by the reviewer's comment on the simulation length, we have substantially extended the MLMD sampling in the revised manuscript: whereas the original relied on trajectories of less than 10 ns per temperature, the revised manuscript relies on trajectories over 100 ns at every temperature. These trajectories are more than two orders of magnitude longer than the AIMD reference of Marcolongo and Marzari and more than an order of magnitude longer than the MLMD reference of Gigli et al. The conductivities calculated from the new trajectories using EH formula, which include correlation, are presented in the Comment 4.

Another point of clarification concerns original Figure 3f, which the reviewer interprets as an Arrhenius plot. It is not. For convenience, we reproduce the figure here:

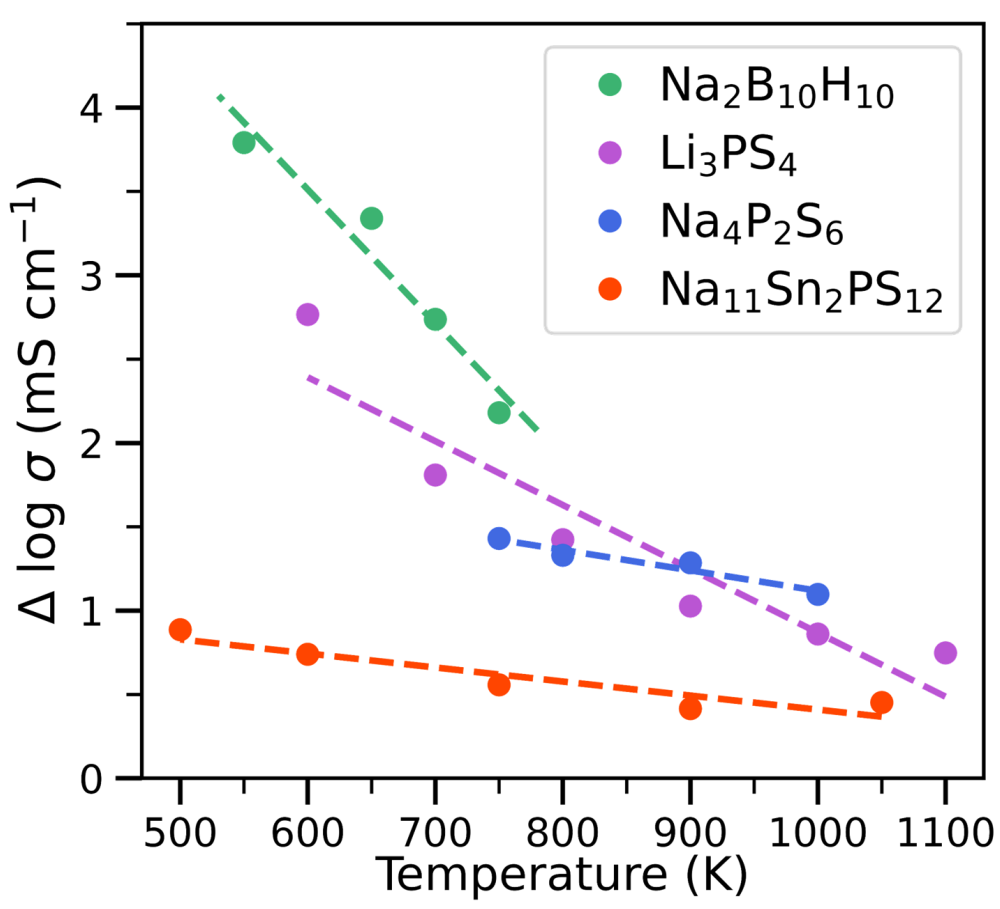

Figure 8. Reproduction of Figure 3f from the original manuscript.

The y-axis of this figure is the change of orders of magnitude in conductivity between constrained and unconstrained simulations versus temperature, rather than the $\log(\sigma T)$ versus $1/T$ of a conventional Arrhenius plot. $LiBH_4$ is absent because, once rotation and translation of $BH_4{}^-$ are constrained, its conductivity drops below the detection limit of our simulations, and the change therefore cannot be quantified. This does not, however, indicate that the MSDs of the unconstrained and rotationally constrained simulations are not converged; in fact, even their collective quantities are well converged (Figure 9).

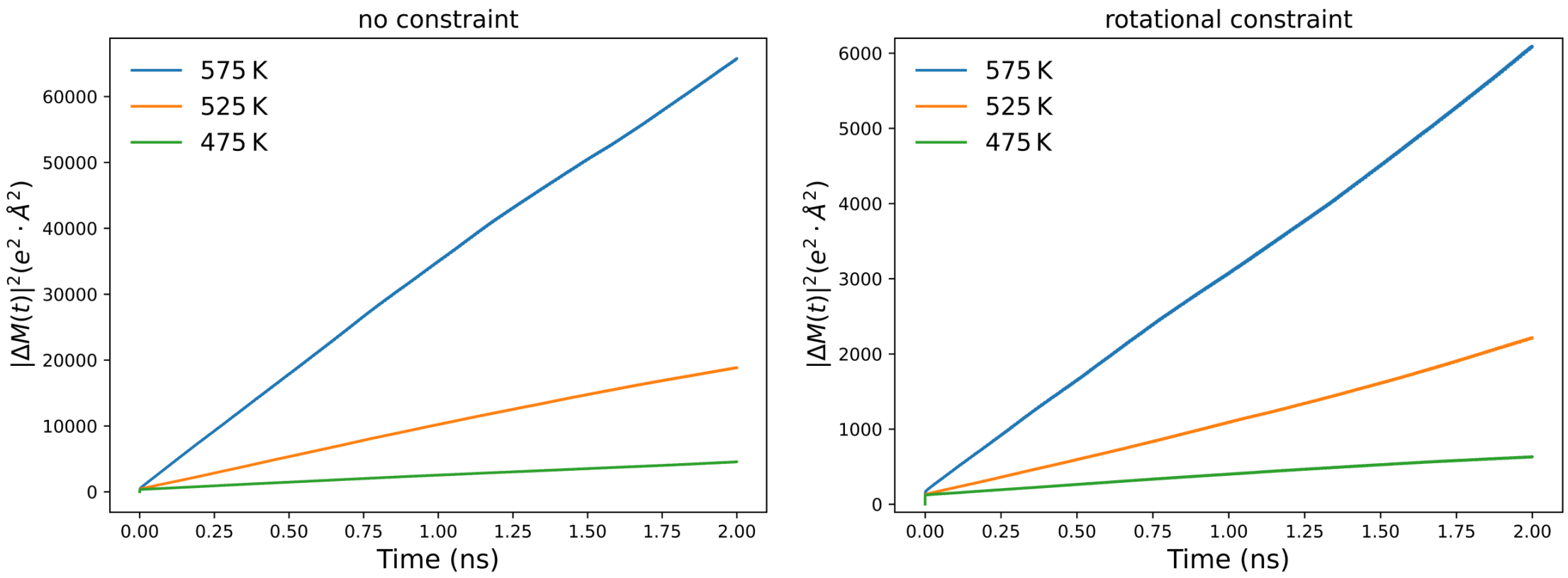

Figure 9. Mean square dipole displacement $|\Delta M(t)|^2$ as a function of time, where $\Delta M(t) = \sum_i q_i[\mathbf{r}_i(t) - \mathbf{r}_i(0)]$. Each curve is averaged over three independent 50 ns trajectories.

We must nevertheless acknowledge that the original Figure 3f is somewhat misleading and carries limited physical significance. Starting from $\sigma \propto \exp(-E_a/k_BT)$, or equivalently $\log\sigma \propto -E_a/k_BT$, and recalling our discussion in the *Section* "Correlating anion dynamics with migration enthalpy and prefactor" (renamed "Anion dynamics tune conductivity through barrier and prefactor" in the revised manuscript) that constraining a given motion mode raises E_a, we can write $\Delta\log\sigma = \log\sigma_1 - \log\sigma_2 \propto (-E_a/k_BT) - [-(E_a + \Delta E_a)/k_BT] = \Delta E_a/(k_BT)$. The change of orders of magnitude is therefore expected to shrink at higher temperature simply on thermodynamic grounds, and the original Figure 3f is no more than a minor corollary of the underlying increase in the migration barrier. The effect of anion motion modes on E_a itself is discussed explicitly in the *Section* "Anion dynamics tune conductivity through barrier and prefactor", so the present figure is largely redundant and has been removed from the revised manuscript.

Accordingly, we have removed the misleading statements associated with the original Figure 3f from both the *Abstract* and *Results* sections, as detailed below.

Change 1 (*Abstract*, lines 18)

- Original: "..., and their impact intensifies at lower temperatures"
- Revised: Deleted.

Change 2 (*Results*, lines 224–227)

- Original: "The temperature dependence of the order-of-magnitude decrease in conductivity caused by dynamic couplings is shown in Figure 3f. These anion dynamics are crucial for enabling superionicity, and become even more critical as the temperature decreases."
- Revised: Deleted.

That said, when both rotation and translation are constrained the mean square dipole displacement no longer exhibits diffusive behaviour (Figure 10a), and we therefore employ well-tempered metadynamics to quantify the contribution of polyanion translation to the migration free-energy barrier. This avoids the need to extract the barrier change from an Arrhenius plot, and has the additional advantage that the resulting free-energy barrier naturally incorporates migration entropy contribution. The results are shown in Figure 10b: when only rotation is constrained, the free-energy barrier changes by 0.03 eV, whereas further constraining translation raises the barrier by 0.52 eV to 0.83 eV—a value that is indeed difficult to converge in conventional MD.

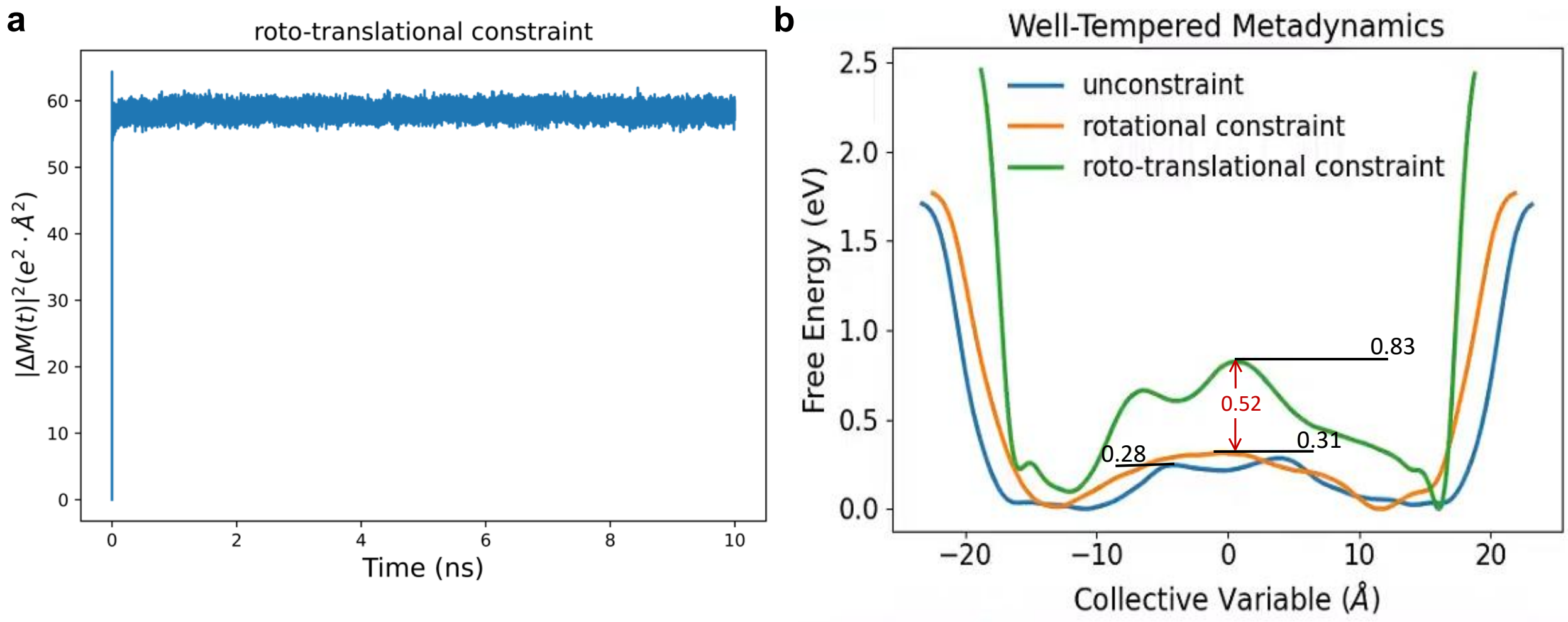

Figure 10. (a) Mean square dipole displacement $|\Delta M(t)|^2$ of $LiBH_4$ under simultaneous rotational and translational constraints at 575 K, with $\Delta M(t) = \sum_i q_i[\mathbf{r}_i(t) - \mathbf{r}_i(0)]$. The signal plateaus rather than growing linearly, confirming the absence of collective diffusion of Li^+. (b) Free-energy profiles of Li^+ migration in $LiBH_4$ obtained from well-tempered metadynamics at 300 K under three constraint conditions: unconstrained (blue), rotationally constrained (orange), and rotationally and translationally constrained (green). The migration barrier increases from 0.28 eV in the unconstrained case to 0.31 eV under rotational constraint, and further to 0.83 eV when translation is also constrained, corresponding to an additional 0.52 eV barrier increase from translational constraint alone. This figure also appears as Fig.6c in the revised manuscript.

(4) We would qualify, however, the reviewer's characterisation of kMC as the "gold standard" for ion transport. Below we discuss three specific reasons why this characterisation is, in our view, unwarranted in the context of the present work.

(i) *Reliance on the NEB–TST protocol*
From transition state theory, the rate constant reads

$$k = \frac{k_B T}{h} \exp\left[-\frac{\Delta G_m^\ddagger}{k_B T}\right] = \frac{k_B T}{h} \exp\left[\frac{\Delta S_m^\ddagger}{k_B}\right] \exp\left[-\frac{\Delta H_m^\ddagger}{k_B T}\right], \tag{4}$$

where $\Delta G_m^\ddagger$ is the migration Gibbs free energy, which decomposes into the migration entropy $\Delta S_m^\ddagger$ and the migration enthalpy $\Delta H_m^\ddagger$. For solids, this is commonly written as

$$\frac{k_B T}{h} \exp\left[\frac{\Delta S_m^\ddagger}{k_B}\right] \exp\left[-\frac{E_m}{k_B T}\right] = \nu^*(T) \exp\left[-\frac{E_m}{k_B T}\right], \tag{5}$$

with ν^* the attempt frequency (prefactor) and E_m the migration barrier. As in the two papers cited by the reviewer, lattice kMC studies typically adopt the protocol of assigning ν^* an empirical value and obtaining E_m from NEB calculations on a single migration pathway. This protocol introduces several drawbacks that, in our view, prevent kMC from serving as a gold standard.

First, in plastic crystals such as $LiBH_4$, computing E_m from a single NEB pathway can neglect the fact that a migrating Li^+ ion experiences dynamically reorienting ${BH_4}^-$ sublattice. In reality, a migrating Li^+ ion moves through a dynamically reorienting BH_4^-

environment, so the instantaneous migration barrier, and possibly also the hopping prefactor, can depend on the local orientational configuration encountered during the hopping event. The effective migration kinetics should therefore be viewed as an orientationally averaged process over the thermally accessible BH_4^- configurations at a given temperature. If the orientations of several neighbouring BH_4^- units affect the hopping barrier, the relevant average should involve their combined orientational configurations. A single static 0 K NEB pathway, even when all atoms are allowed to relax along the path, samples only a selected region of this high-dimensional orientational space and does not automatically represent the finite-temperature orientational average experienced by a migrating Li^+ ion. In $LiBH_4$, where BH_4^- units rotate at over $10^3\,\mathrm{ns}^{-1}$, the NEB-based kMC protocol is therefore intrinsically approximate relative to this more faithful description.

(ii) *Long times do not guarantee complete transport dynamics*
As is the case in the two studies cited by the reviewer, kMC applied to superionic conductors typically enumerates single-ion hops as the elementary events, whereas concerted migration can dominate transport in fast ion conductors (*Nat. Commun.* **8**, 15893 (2017)). A well-recognised source of error in kMC is precisely an incomplete event list (Voter, A. F. Introduction to the kinetic Monte Carlo method. In *Radiation Effects in Solids* (eds. Sickafus, K. E., Kotomin, E. A. & Uberuaga, B. P.) 1–23 (Springer, 2007)). Under such conditions, even though kMC can be propagated to arbitrarily long times, this long-time behaviour reflects the dynamics of a simplified event library rather than the full transport dynamics of the superionic conductor.

(iii) *A concrete example from the present work*
Our own analysis of $LiBH_4$ provides a direct example. When BH_4^- rotation is constrained, the activation energy changes by only about 0.02 eV (Figure 11b), whereas the conductivity decreases by roughly one order of magnitude (Figure 11a). Such a small change in E_a cannot account for the observed conductivity drop, indicating that the dominant change resides in the prefactor rather than in the migration barrier. Thus, even if an analogous rotational constraint were imposed in an NEB calculation, the resulting barrier would remain largely insensitive to this effect. A NEB-based kMC model using a E_m and an empirical ν^* would therefore miss this prefactor change and can yield quantitatively incorrect transport kinetics.

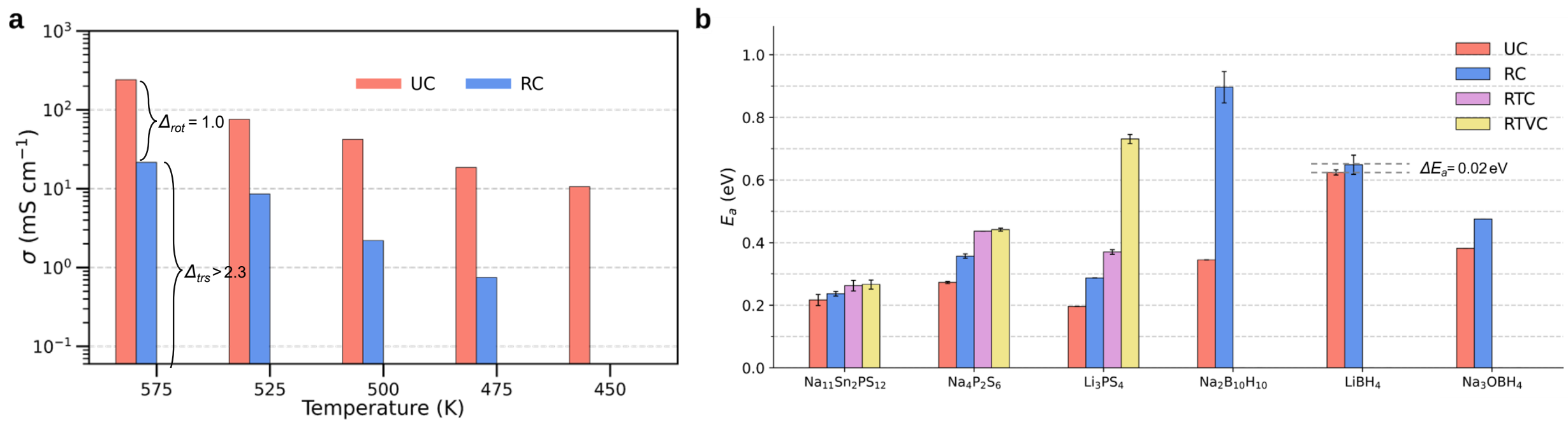

Figure 11. (a) Conductivity of $LiBH_4$ evaluated from the Einstein–Helfand relation in the unconstrained (UC, red) and rotationally constrained (RC, blue). Constraining rotation suppresses σ by approximately one order of magnitude. This figure also appears as Supplementary Fig.22 in the revised manuscript. (b) Migration barriers E_a extracted from Arrhenius fits for six materials under up to four constraint conditions: unconstrained (UC), rotationally constrained (RC), roto-translationally constrained (RTC), and roto-translational-vibrationally constrained (RTVC). For $LiBH_4$, rotational constraint raises E_a by only $\Delta E_a = 0.02\,\text{eV}$, despite the order-of-magnitude reduction in σ shown in panel (a), indicating that the effect of rotation resides predominantly in the prefactor. This figure also appears as Fig.5 in the revised manuscript.

For the reasons above, we regard kMC and MLMD as complementary rather than ranked: kMC is unrivalled for reaching millisecond timescales once a mechanism, a reliable rate catalogue, and correct attempt frequencies are established, while MLMD is the appropriate tool for discovering mechanisms and quantifying the coupling between cation transport and anion dynamics, which is the central scientific focus of the present work.

6. Regarding Figure 3f, some fits do not appear linear; for instance, Li3PS4. Are they seeing a phase transition? Is there a sudden change in slope at higher temperatures? They don't comment on this important aspect.

   This topic has been discussed in detail in the third and fourth paragraphs of Comment 5(3).

7. In Figure 2, the use of octants and the analysis of changes in angle projections over time was introduced in 10.1021/acsenergylett.1c02815, which they cite. It would be appropriate if they acknowledged this idea. In this context, a better treatment of anion rotations can be achieved through the use of quaternions and quaternion coordinates as in 10.1073/pnas.2316493121. Have these authors attempted the implementation of quaternion analysis for their data?

   We thank the reviewer for this comment, which prompted us to improve our analysis substantially. First, we have added a citation to *ACS Energy Lett.* **7**, 1403–1411 (2022) to acknowledge that the octant-based angle-projection analysis in Fig. 4a–c follows the approach used in that work:

   Added (*Results*, lines 348–350)
   "Following the angle-projection approach of Scholz et al.[14], we track the reorientation dynamics of each rotor by monitoring the polar and azimuthal angles (θ and ϕ) defined with respect to a reference frame parallel to the lattice vectors (insets of Fig. 4a–c)."

   In the original manuscript, reorientation events were counted directly from the angle trajectories in a rather ad hoc way. Motivated by this comment, we have replaced that treatment with a quantitative analysis based on Ivanov model, which yields both the reorientation frequency and the jump amplitude (Figure 12d,e).

   Regarding quaternions, we considered their use carefully but concluded that a quaternion-based treatment would provide little additional insight beyond our current framework, for three reasons.

   First, for the slow rotors, such as α-Li_3PS_4 and $Na_{11}Sn_2PS_{12}$, reorientation does proceed as discrete jumps, and a quaternion-based analysis could indeed be used to obtain the reorientation frequency. However, for such discrete jumps the Ivanov model is equally applicable, and it has the further advantage of also yielding the characteristic jump amplitude (angle). We have moreover verified, for α-Li_3PS_4, that the reorientation frequency from the Ivanov analysis ($0.17\,ns^{-1}$, $105.2°$ at 800 K) is of the same order of magnitude as the value reported in 10.1073/pnas.2316493121 ($0.5\,ns^{-1}$, $\sim100°$ at 800 K). Quaternions therefore offer little advantage over the Ivanov treatment for these systems.

   Second, for fast rotor BH_4^-, any choice of time window only yields the net orientational change between the two endpoints, i.e., a single equivalent rotation. The actual trajectory typically reaches this net orientation through a sequence of rotations about different axes by different angles, not through a single rotation about one axis. The net angle therefore does not correspond to any rotation that actually took place, and is physically meaningless as a "jump angle". This holds for any method, since the limitation lies in the net-displacement nature of the measurement, not in how orientations are represented.

   Finally, on the suggestion that quaternion coordinates could replace the angle projections in Figure 12a–c: we would emphasize that Figure 12a–c are intended only to give a qualitative, visual impression of how fast or slow each rotor reorients, and such projections are indeed sensitive to the choice of reference frame. The physically rigorous quantities in our analysis are not these projections but the reorientation frequencies in Figure 12d, obtained from the Ivanov-model fit and from equation (8) (marked-up manuscript), which are representation-independent. The role of Figure 12a–c is therefore illustrative, and the quantitative conclusions of the paper do not rest on them. For the same reason, we have also removed Figure S4, which showed angle projections only for illustrative purposes.

   For these reasons, we did not adopt quaternion analysis.

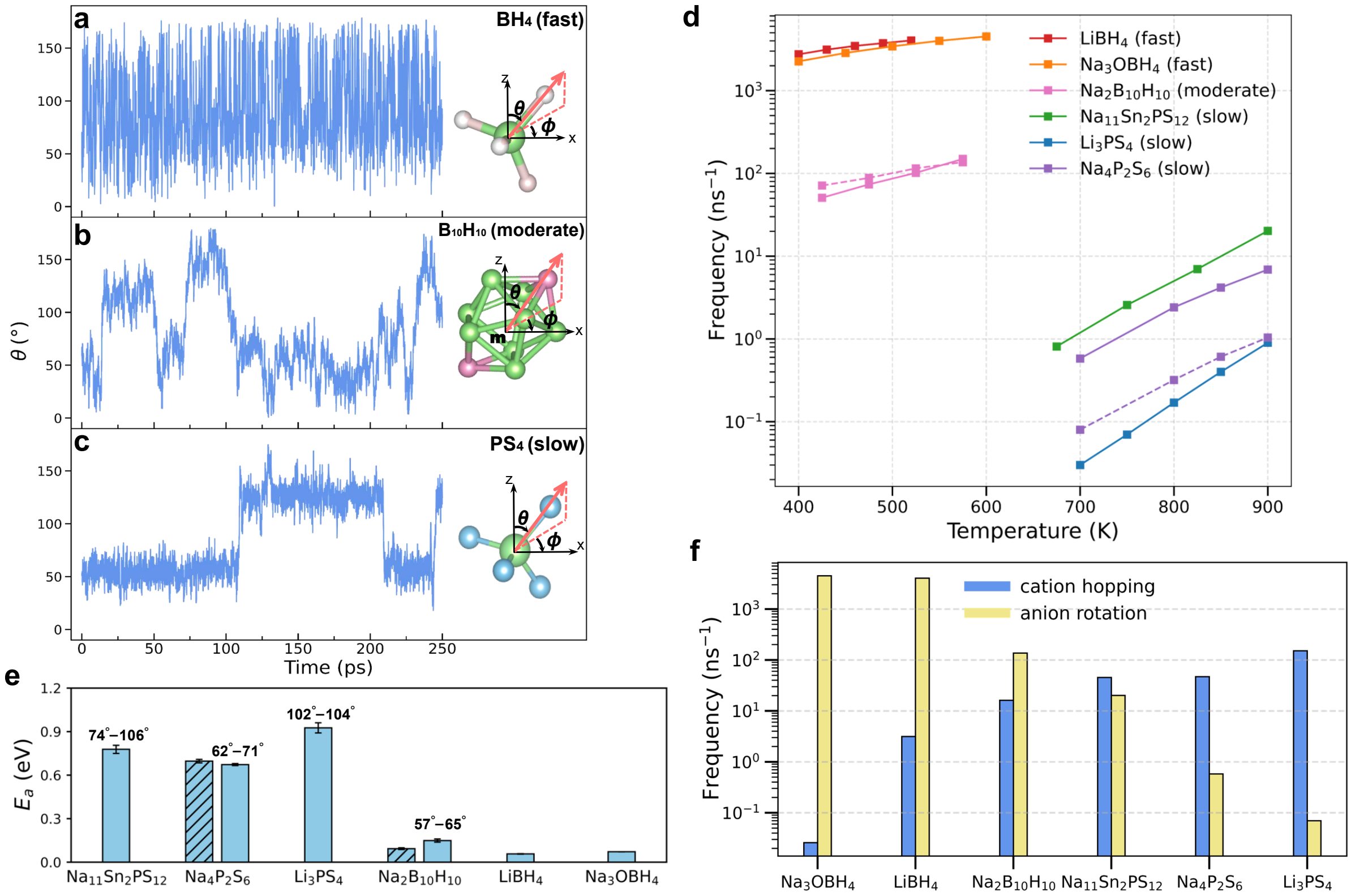

Figure 12. Polar angle of (a) BH_4^- in $LiBH_4$ at 500 K, (b) $B_{10}H_{10}^{2-}$ in $Na_2B_{10}H_{10}$ at 550 K, and (c) PS_4^{3-} in $Na_{11}Sn_2PS_{12}$ at 900 K, representing fast, moderate, and slow reorientational dynamics, respectively. Coordinates for the rotors are defined as shown in the insets. In (b), **m** denotes the center of mass of the $B_{10}H_{10}^{2-}$, and the apical and equatorial B atoms are represented by the pink and green spheres, respectively. (d) Anion reorientation frequencies in the six SEs at various temperatures. For $Na_4P_2S_6$ and $Na_2B_{10}H_{10}$, solid lines correspond to reorientation of the line connecting the two P atoms in $P_2S_6^{4-}$ and the two apical B atoms in $B_{10}H_{10}^{2-}$, respectively. Dashed lines correspond to the internal rotation of $P_2S_6^{4-}$ and rotation about the line connecting the two apical B atoms, respectively. (e) Activation energies (E_a) of anion reorientation, obtained from Arrhenius fits of the reorientation frequencies in (d). For $Na_4P_2S_6$ and $Na_2B_{10}H_{10}$, two reorientation modes are shown. Solid bars correspond to reorientation of the principal axis (P–P in $P_2S_6^{4-}$, apical B–B in $B_{10}H_{10}^{2-}$); hatched bars correspond to internal rotation of $P_2S_6^{4-}$ and rotation of $B_{10}H_{10}^{2-}$ about this axis. Numbers above the bars indicate the temperature-dependent ranges of reorientation angles fitted using the Ivanov model. No values are reported for $LiBH_4$ and Na_3OBH_4, because their reorientations are difficult to define as discrete jumps. (f) Cation hopping frequencies and anion reorientation frequencies for six ionic conductors. Frequencies were evaluated at 520–600 K for the borohydride and antiperovskite systems and at 700–900 K for the thiophosphates. This figure also appears as Fig.4 in the revised manuscript.

8. Another important layer of disorder that is not discussed but is undoubtedly present in Na11Sn2PS12 is the mixing of SnS4 and PS4 polyanion. This mixing certainly contributes to changes in ion transport. Polyanion mixing/disorder certainly changes the potential energy surface perceived by a traveling ion. This is central in NaSICON structure, where some of these authors have published before. However, molecular dynamics cannot describe these mixed anion species properly, and stochastic methods, such as Monte Carlo are paramount 10.1038/s41467-022-32190-7, and 10.1021/acs.chemmater.0c02695.

   We appreciate the reviewer's comments and the recommended references, which highlight the importance of stochastic methods for capturing polyanion mixing. We have cited these papers in the revised manuscript:
   Added citation (*Introduction*, line 42):
   "From a static perspective, thermally activated cation hops occur by overcoming the energy barrier defined by the rigid host lattice; specific anion packing[4], polyanion mixing[5,6] and site disorder[7] can facilitate cation diffusion."

   Indeed, polyanion mixing is an important factor that can modulate ionic conductivity. In the Na−Sn−P−S system, the conductivity can be tuned by varying the SnS_4^{4-}/PS_4^{3-} ratio, i.e., the composition (*Adv. Energy Mater.* **15**, 2500861, (2025)), and altering this ratio inevitably introduces a random distribution of SnS_4^{4-} and PS_4^{3-}. However, this compositional tuning and the accompanying SnS_4^{4-}/PS_4^{3-} random distribution are separate factors from the PS_4^{3-} orientational disorder studied here, and a comprehensive treatment of them is beyond the scope of the present work.

   To disentangle these effects, we fix the composition at $Na_{11}Sn_2PS_{12}$ and focus on the role of PS_4^{3-} orientational disorder. This choice is justified on two grounds:

   (1) This composition has been successfully synthesized as a stoichiometric compound (*Energy Environ. Sci.* **11**, 87-93, (2018)). DFT calculations across a series of related compositions ($Na_{15}Sn_3PS_{16}$, $Na_{11}Sn_2PS_{12}$, Na_7SnPS_8, $Na_{10}SnP_2S_{12}$, $Na_{13}SnP_3S_{16}$) identify $Na_{11}Sn_2PS_{12}$ as the most stable one (*Adv. Energy Mater.* **11**, 2003196, (2021)). Both experiment and theory therefore support $Na_{11}Sn_2PS_{12}$ as a representative composition.

   (2) Within this composition, single crystal diffraction shows that PS_4 and SnS_4 occupy two distinct Wyckoff sites ($8a$ and $16e$, respectively), each with full occupancy (*Energy Environ. Sci.* **11**, 87-93, (2018)), indicating that no significant PS_4/SnS_4 random distribution. Computationally, DFT calculations on various SnS_4/PS_4 orderings at this composition find that the configuration with PS_4 fully on $8a$ and SnS_4 fully on $16e$ is energetically the most stable (*Adv. Energy Mater.* **11**, 2003196, (2021)). Taken together, the $Na_{11}Sn_2PS_{12}$ model used in our MD simulations is a representative one.

9. I am utterly surprised to read that SnS4 units are immobile in Na11Sn2PS12. This could be because they had just chosen a convenient, perhaps unrepresentative, arrangement/ordering of the PS4/SnS4 in their MDs. As explained in point 7., this question can only be addressed by performing MD (or better Monte Carlo) on a significantly varied set of PS4/SnS4 arrangments.

We thank the reviewer for pointing this out. We agree that the original statement was imprecise. We have revised the wording to more accurate:

Change (*Results*, Fig.2 caption, lines 306–307)

- Original: "the SnS_4^{4-} typically does not exhibit any rotational mobility;[10]"
- Revised: "... previous study suggests that SnS_4 exhibit no orientational disorder."

Furthermore, the suggestion to test a significantly varied set of PS_4/SnS_4 arrangements is addressed in detail in our response to point 8(2).

10. In Figure 2c, it's unclear if P_2S_6 or PS_4. The inset is confusing. Particularly, what data are they currently showing?

We thank the reviewer for raising this. In the revised manuscript, we now show only PS_4 in $Na_{11}Sn_2PS_{12}$ in Fig.4c, and have updated the caption to specify the material and simulation temperature for each panel. The P_2S_6 inset has been removed from this panel to avoid confusion. For convenience, the revised figure is also shown above as Figure 12a–c.

11. On page 7 they bring about the concept of moment of inertia. This is important, and they should be more quantitative about this by computing the various moments of inertia of the various polyanion species investigated.

We thank the reviewer for this excellent suggestion. We have computed and compared the moments of inertia of $B_{10}H_{10}{}^{2-}$ and $BH_4{}^-$:

Change 1 (*Results*, lines 379–382)

- Added: "Notably, although $B_{10}H_{10}{}^{2-}$ has a reorientation barrier comparable to that of $BH_4{}^-$, its frequency is ∼1.6 orders of magnitude lower, in part due to larger moment of inertia ($I_A = 192$, $I_B = I_C = 249\,\mathrm{amu\,Å^2}$ for $B_{10}H_{10}{}^{2-}$, versus $4\,\mathrm{amu\,Å^2}$ for the spherical top $BH_4{}^-$)."

Building on this comparison, we further note that the moment of inertia, together with the reorientation barrier, leads to a concrete design principle, which we have also added:

Change 2 (*Results*, lines 382–385)

- Added: "This points to two design principles for maximizing the paddle-wheel effect's contribution: the cation hop and anion reorientation frequencies should be matched, which in turn requires balancing the reorientational barrier imposed by the host lattice and the moment of inertia of the rotor itself."
- Reason: During revision, we fitted the barriers of the large-amplitude reorientation jumps (Figure 12e) and found that $B_{10}H_{10}{}^{2-}$ (0.14 eV) has a barrier close to that of $BH_4{}^-$ (0.07 eV for Na_3OBH_4), yet its reorientation frequency is ∼1.6 orders of magnitude lower (Figure 12d). This demonstrates that the barrier alone does not set the reorientation frequency: the moment of inertia is a second key factor. Tuning a rotor's frequency—for example, matching it to the Na^+/Li^+ hop frequency to

maximize the paddle-wheel effect—therefore requires designing both the barrier and the moment of inertia.

12. The definition of plastic structures is not given.

    We thank the reviewer for pointing this out. Plastic crystals are central to this work, and a clear definition should indeed be provided. Following the description by Scholz et al. (*ACS Energy Lett.* **7**, 1403–1411 (2022)), we have refined the sentence:

    Change (*Results*, lines 286–287; 228–229)

    - Original: "In these plastic crystal structures, polyanion reorientations and partial occupancy of mobile cation are observed."
    - Revised: "Fig. 1 shows the crystal structures of the six plastic crystals, which feature polyanions with dynamic orientational disorder, translationally fixed on their lattice sites.[14]"

13. They used the terminology "cation local vibration frequencies". For me, it's extremely tough to visualize/understand "cation local vibration frequencies", as a vibrational mode should always include at least 2 entities. So, how can we define a cation-only local vibrational frequency? To me, this is an ill-defined term. They should revise this part as it doesn't make much sense.

    We thank the reviewer for pointing this out. We agree that the term "cation local vibration frequencies" is ambiguous. In our work, the quantity discussed is the phonon density of states (DOS) and the partial phonon DOS of Li/Na. In the classical harmonic limit, it is equivalent to the Fourier transform of the mass-weighted velocity correlation function (Dove, M. T. *Introduction to Lattice Dynamics* Appendix F (Cambridge University Press, 1993)). Accordingly, under this approximation, the partial phonon DOS of Li/Na refers to the sum over all phonon modes, with each mode weighted by the squared Li/Na components of its eigenvector. We have therefore revised the terminology in the manuscript as follows:

    Change (*Results*, lines 399–401)

    - Original: "It can also be seen from phonon DOS that the timescales of local potential fluctuations from anion motion aligh wtih cation local vibration frequencies (Figure S17)"
    - Revised: Deleted.

    We also corrected the PDOS formula (equation (10) → equation (11)), where a mass factor was inadvertently omitted. The calculations themselves used the correct mass-weighted form, so no results are affected.

14. Most of the DFT settings are not available, and therefore, any reader cannot reproduce these simulations. What is the plane-wave cut-off used? And how many valence electrons were treated explicitly with the PAW used? What DFT total energy convergence was used? What integrator did they use to integrate the equation of motion? All these details are vital to any interested reader. Why did they use DP-GEN as opposed to other existing machine learning interatomic potential methods? Likewise, no details are given about the MDs using CP2K, these are ab initio MDs, where they do not say how they choose the basis set and a functional. I had to dig out this information. In summary, these details

are extremely important. However, this part appears quite incomplete.

We thank the reviewer for pointing out that several important computational details were missing from the original manuscript. We agree that these parameters are essential for reproducibility.

Regarding the plane-wave cutoff, the reviewer is correct that the original manuscript only stated the convergence criterion (forces converged to within 1 meV/Å) without specifying the actual cutoff value adopted for each system. In our work, we followed the convergence-testing protocol recommended for DeePMD training-data generation. Based on these tests, we adopted a plane-wave cutoff of 650 eV for $Na_2B_{10}H_{10}$ and $LiBH_4$, 600 eV for Na_3OBH_4, 500 eV for Li_3PS_4 and $Na_4P_2S_6$, and 400 eV for $Na_{11}Sn_2PS_{12}$. The corresponding Γ-centered k-point meshes are 2×2×2 for $Na_2B_{10}H_{10}$, $LiBH_4$, and Li_3PS_4; 3×3×3 for Na_3OBH_4 and $Na_4P_2S_6$; and 2×2×1 for $Na_{11}Sn_2PS_{12}$. The full convergence-test data for both the plane-wave cutoff and k-point sampling across all six systems are shown in Figures 13 and 14 below, and have been added to the revised Supporting Information as Supplementary Fig.29 and 30.

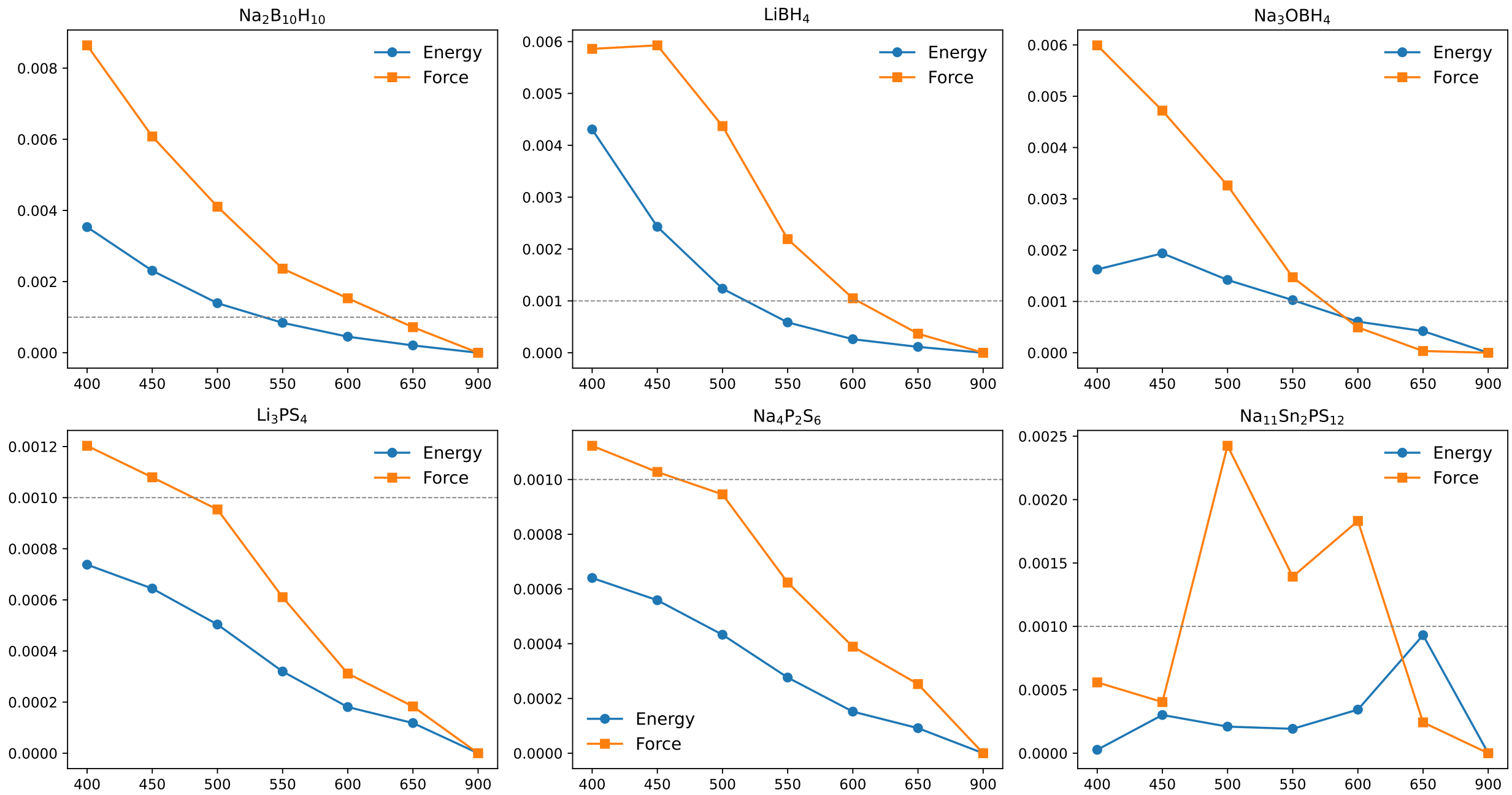

Figure 13. Plane-wave cutoff convergence tests for the six systems. The horizontal axis shows the plane-wave cutoff energy in eV, and the vertical axis shows the absolute changes in total energy and RMS force relative to the results obtained with the highest cutoff energy for each system. This figure also appears as Supplementary Fig.29 in the revised SI.

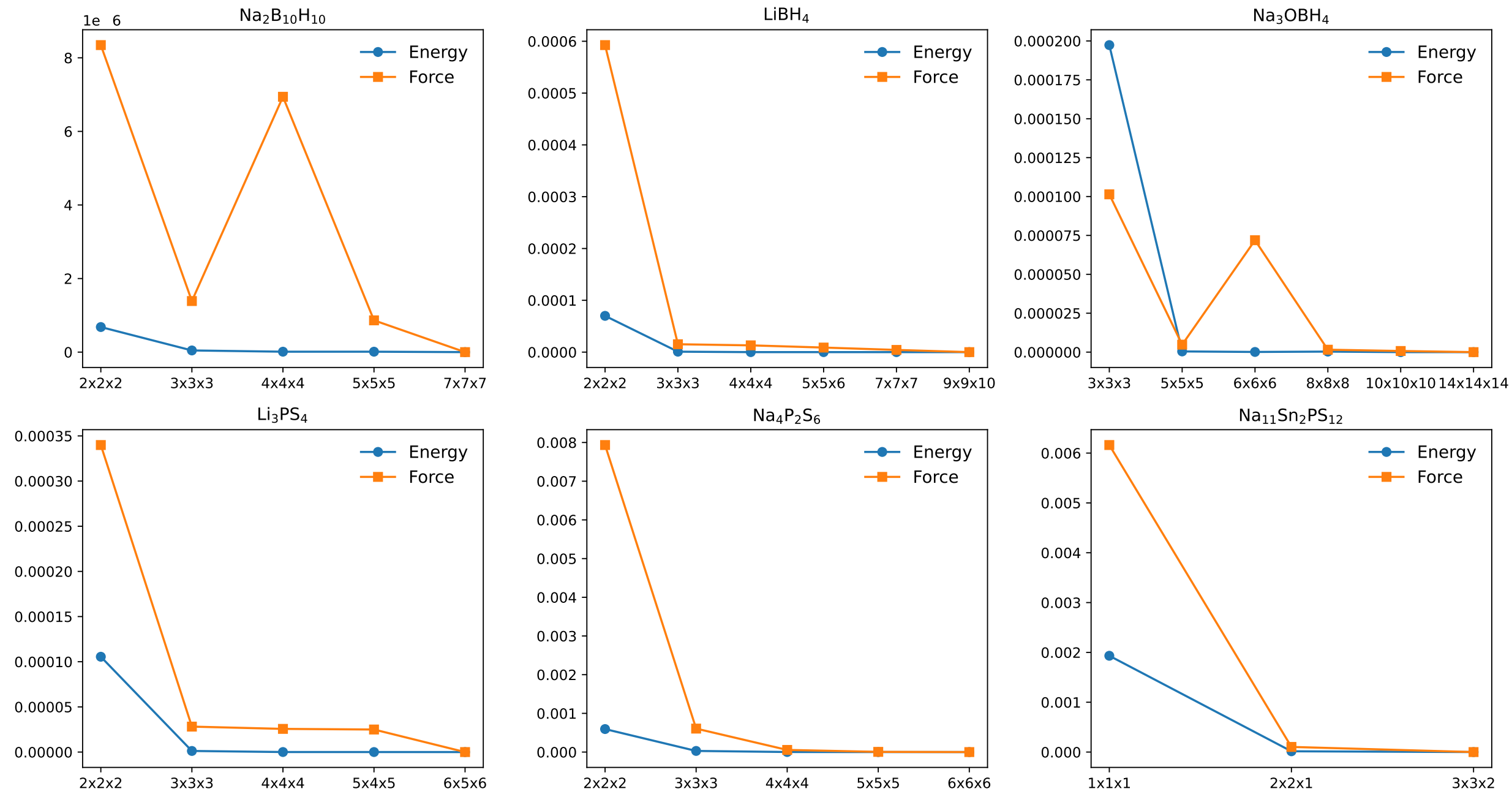

Figure 14. k-point convergence tests for the six systems. The horizontal axis shows the k-point mesh used for Brillouin-zone sampling, and the vertical axis shows the absolute changes in total energy and RMS force relative to the results obtained with the densest k-point mesh for each system. This figure also appears as Supplementary Fig.30 in the revised SI.

The revised *Methods* section has also been updated to include the cutoff and k-point values explicitly:

Added 1 (*Methods*, lines 625–628)

"Specifically, the plane-wave cutoffs are 650 eV for $Na_2B_{10}H_{10}$ and $LiBH_4$, 600 eV for Na_3OBH_4, 500 eV for Li_3PS_4 and $Na_4P_2S_6$, and 400 eV for $Na_{11}Sn_2PS_{12}$. The Γ-centered k-point meshes are 2×2×2 for $Na_2B_{10}H_{10}$, $LiBH_4$, and Li_3PS_4; 3×3×3 for Na_3OBH_4 and $Na_4P_2S_6$; and 2×2×1 for $Na_{11}Sn_2PS_{12}$."

Regarding the PAW valence electrons, this information was indeed missing from the original manuscript. The VASP PAW potentials used in this work treat 1, 1, 3, 1, 6, 5, 6, and 4 valence electrons for Li, Na, B, H, O, P, S, and Sn, respectively. This has now been added to the revised *Methods* section:

Added 2 (*Methods*, lines 621–622)

"The VASP PAW potentials treat 1, 1, 3, 1, 6, 5, 6, and 4 valence electrons for Li, Na, B, H, O, P, S, and Sn, respectively."

Regarding the total energy convergence, the electronic self-consistency loop was converged to 10^{-6} eV for all systems. This has now been stated in the revised *Methods* section:

Added 3 (*Methods*, lines 622–623)

"The electronic self-consistency convergence criterion was set to 10^{-6} eV."

Regarding the integrator, the equations of motion were integrated using the velocity Verlet algorithm in all MD simulations. This has now been stated in the revised *Methods* section:

Added 4 (*Methods*, lines 663–664)

"The equations of motion were integrated using the velocity Verlet algorithm."

Regarding the choice of DP-GEN, we adopted it because it has been successfully applied to solid-state electrolyte systems in previous work (*J. Chem. Phys.* **154**, 094703 (2021)). Its concurrent learning scheme automates the exploration–labeling–training loop, which is well suited for training potentials across multiple systems and a wide temperature range.

Regarding the MD simulations performed with CP2K, we would like to clarify that these are MD using the trained deep potential models, not ab initio MD. Therefore, no Gaussian basis set or exchange functional is involved at the MD stage. We have revised the *Methods* section to clarify this point:

Change (*Methods*, lines 650–652)

- Original: "MD simulations for production ... were performed with CP2K package."
- Revised: "MD simulations were performed with the trained DP models using a modified version of the CP2K package."

Additional revisions to the manuscript (not requested by either reviewer):

During the restructuring of the manuscript, some sentences were moved from their original sections to different sections in the revised manuscript without substantive modification. These organizational changes are not listed as separate items in the changelog below.

Abstract

We have made several revisions to the abstract. The original abstract exceeded the journal's 150-word limit, and the revised version has been reduced to within 150 words. Each change is listed below with the reasons for revision. All instances of "diffusion" or "migration" have been changed to "transport" because our central quantity is ionic conductivity, which describes field-driven ionic response.

Change 1 (*Abstract*, lines 3–9)

- Original: "Solid electrolytes (SEs) feature polyanion frameworks and mobile cations. Understanding and quantifying the impact of polyanion dynamics on cations will unravel the complex role of anions in SEs. However, no experimental or computational method can directly extract this information, as anion dynamics inherently couple with other factors affecting ion mobility."
- Revised: "Polyanion dynamics, notably rotation, have long been linked to fast cation transport in solid electrolytes, yet their contribution to ionic conductivity remains unquantified, and even rotation's role, disputed."
- Reason:
  (1) The original contained an abbreviation ("SEs"), disallowed by the journal's guidelines ("Do not include references, acronyms or abbreviations").

  (2) The original was verbose and buried the key question: polyanion dynamics are linked to fast cation transport, yet their contribution to conductivity remains unquantified.

Change 2 (*Abstract*, lines 9–15)

- Original: "Here, we present a pioneering study combining constraint algorithms, which selectively constrain each anion motion mode, and extended machine-learning molecular dynamics to quantitatively reveal the effects of polyanion translation, rotation, and vibration on ionic conductivity across various materials."
- Revised: "Here, we quantify the contributions of polyanion translation, rotation, and vibration to conductivity in plastic crystals by combining a constraint algorithm and machine learning molecular dynamics."
- Reason:
  (1) The original passage was verbose. "We present a pioneering study ... to quantitatively reveal ..." → "We quantify the ...".

  (2) "which selectively constrain each anion motion mode" was removed because methodological details of this kind belong in the main text, not the abstract, which should emphasize what the study achieves.

  (3) The word "extended" was vague and redundant.

  (4) Moving "quantify" to the front emphasizes the study's central contribution—quantitative decomposition of ionic conductivity by motion mode.

  (5) "Effects" → "contributions": the latter implies a quantifiable share of total conduc-

tivity, aligning with "quantify"; the former is more qualitative.

Change 3 (*Abstract*, lines 17–18)

- Original: "..., with one dominating per material class, ..."
- Revised: Deleted.
- Reason: This claim was erroneous: the dominant motion mode differs across the three thiophosphates studied, despite their belonging to the same material class.

Change 4 (*Abstract*, lines 24–25 and 27–28)

- Original: "Analyses uncover anion motion modes drive cation migration by inducing disorder in the cation sublattice."
- Revised: "We find that rotation and translation each can enhance conductivity by over two orders of magnitude by inducing cation disorder;"
- Reason: Additional analyses carried out during revision provide stronger evidence that rotation and translation, in particular, can induce cation disorder (lines 471–481, 511–517).

Change 5 (*Abstract*, lines 30–33)

- Original: "These findings highlight polyanion dynamics as key to superionicity and guide the development of advanced conductors."
- Revised: "These findings demonstrate that polyanion dynamics are crucial to superionicity and offer design principles for superionic conductors."
- Reason:
  (1) "highlight ... as key" → "demonstrate ... are crucial": rotation and translation can change the ionic conductivity by more than two orders of magnitude, which supports the stronger wording.

  (2) "guide the development" → "offer design principles": the *Discussion* articulates specific principles: considering rotation, translation, and vibration rather than focusing on rotation alone; targeting both the activation barrier and the prefactor rather than the barrier alone; and exploiting dynamic anion–cation couplings that induce dynamic cation disorder.

  (3) "advanced conductors" → "superionic conductors": specifies the material class actually addressed.

Introduction

- The original introduction established the background, highlighted key contradictions, and raised open questions in the field, but the concluding paragraph did not adequately tie these threads together. We have rewritten the final paragraph to explicitly address each point, bridging the motivation to our work (Change 7, Change 8, Change 9).
- The paddle-wheel debate is presented more neutrally, with supporting and challenging studies listed separately (Change 6, Change 11).
- Important background added (Change 10, Change 15).
- Less relevant passages deleted (Change 12, Change 13, Change 17).
- Imprecise or erroneous statements revised (Change 14, Change 16, Change 18).

Change 6 (key revision; *Introduction*, lines 55–64)

- Original: "A closely related and highly debated concept is the paddle-wheel effect, proposed over thirty years ago, suggesting that polyanion rotation enhances cation migration. While superionicity is believed to be coupled with rotation, and recent studies have leveraged this principle to search for superionic conductors,[30] the existence of the paddle-wheel effect remains controversial and is under renewed scrutiny.[10,17,31–34]"
- Revised: "Its role is most often described through the paddle-wheel effect[20,35], a hypothesis proposed over thirty years ago and since refined, that polyanion rotation facilitates cation migration.[38] Over the years, some studies have supported its role in enhancing cation conduction,[10,12,15,17,19,39] while a comparable body of work has challenged its existence.[18,33,34,40]"
- Reason:
  (1) We clarify that the paddle-wheel concept itself has evolved over time, and the definition adopted here follows the modern use in Ref. 38 (*Proc. Natl. Acad. Sci. U.S.A.* **122**, e2419892122 (2025).). This is reflected by the added phrase "and since refined" along with the citation of Ref. 38.

  (2) We expand the literature coverage by explicitly listing studies on both sides of the debate, supporting (Refs. 10, 12, 15, 17, 19, 39) and challenging (Refs. 18, 33, 34, 40) the paddlewheel effect. Presenting the two bodies of work in parallel makes the highly debated nature of this concept more evident than a generic statement of controversy.

Change 7 (*Introduction*, lines 149–151 and 153–157)

- Original: "Utilizing six superionic conductors from three distinct material classes, we identify key anion motion modes that critically influence conductivity."
- Revised: "The paddle-wheel effect dominates conductivity only when the polyanion reorientation frequency, quantified using the Ivanov model, matches the cation hopping frequency; when reorientation is much faster or much slower than cation hopping, the paddle-wheel effect persists but conductivity is instead dominated by polyanion translation or vibration, respectively."
- Reason: The original claim is vague. The revised sentence specifies which motion mode dominates and under what condition.

Change 8 (*Introduction*, lines 151–152)

- Added: "We quantify the contribution of the paddle-wheel effect to ionic conductivity and find it always operative."
- Reason: The original *Introduction* had set up the controversy surrounding the paddle-

wheel effect (lines 60–64) but did not clearly state our conclusion on it. This sentence now closes that loop by giving the specific result: a quantitative estimate of conductivity showing that the paddle-wheel effect is universally operative in plastic crystals.

Change 9 (*Introduction*, lines 152–153)

- Added: "Yet we show that no single polyanion motion mode universally dominates cation transport."
- Reason: The *Introduction* had noted that translation and vibration receive less attention than rotation and that their contributions to conductivity have not been quantified (lines 113–117), but the original closing paragraph did not address this setup. This sentence completes the narrative by stating the corresponding result.

Change 10 (*Introduction*, lines 43–53)

- Original: "Dynamic mechanisms have also been proposed, such as the cooperative motion of the host lattice and charge carriers.[8,9] Among these mechanisms, dynamic disorder arising from polyanion motion has been extensively observed, with its rotational disorder receiving significant attention.[10−15]"
- Revised: "In parallel, dynamic mechanisms of ion conduction have been actively investigated. From a phonon–ion coupling perspective, specific phonon modes have been shown to contribute directly to ion hopping, and their effects on ionic conductivity have been quantified by both theory and experiment.[16] From a local perspective, correlations between polyanion dynamics and cation hops have been identified in close spatiotemporal proximity.[10,17−19] However, in contrast to the phonon–ion coupling, their contribution to conductivity remains unquantified. Among polyanion dynamics, rotation has received the most attention."
- Reason:
  (1) It contrasts phonon-based and local-dynamics-based perspectives on ion conduction. The former has been quantitatively linked to conductivity, while the latter has not, which highlights the gap our study addresses.

  (2) Since the two perspectives are two distinct angles, this framing positions our work as offering an alternative viewpoint to the phonon–ion coupling picture.

Change 11 (*Introduction*, lines 65–68)

- Original: "Some typical materials have laid the foundation and provided templates for designing novel SEs that exploit the paddle-wheel effect."
- Revised: "Despite the ongoing debate, several inorganic plastic crystals have been reported in which fast superionic conduction is accompanied by polyanion rotation."
- Reason: The original wording presupposes the validity of the paddle-wheel effect by stating that these materials 'exploit' it, which is inconsistent with the neutral tone we established (lines 60–64), where both supporting and challenging studies are presented in parallel. The revised sentence instead reports only the empirical observation that fast ion conduction co-occurs with polyanion rotation, leaving the causal interpretation open.

Change 12 (*Introduction*, lines 104–105)

- Original: "The dynamic coupling between liquidlike sodium conduction and different modes of motion of this ethan-like anion remains elusive too."
- Revised: Deleted.

- Reason: The *Abstract* and the preceding paragraphs in the *Introduction* already establish that the contribution of polyanion dynamics to conductivity remains unquantified. Restating this for a single system is redundant.

Change 13 (*Introduction*, lines 106–109)

- Original: "Molecular dynamics (MD) simulations can detect paddle-wheel effect ... diffusion properties resulting from rotation."
- Revised: Deleted.
- Reason: Redundant, as the same point is now made earlier at lines 50–52.

Change 14 (*Introduction*, lines 109–113)

- Original: "Moreover, rotation typically manifests following phase transitions, making it experimentally challenging to isolate their effects from other variables."
- Revised: "Across these systems, the onset of polyanion rotation typically accompanies a phase transition, making it experimentally challenging to isolate the effects of rotation from structural changes."
- Reason: "rotation typically manifests following phase transitions" → "... rotation typically accompanies a phase transition, ..." We revised this sentence to avoid implying that polyanion rotation necessarily occurs after a phase transition. The updated wording better reflects that facile polyanion reorientation often occurs concurrently with phase transitions, and may even contribute to stabilizing the high-temperature phase (*Advanced Science* **11** (26), 2306488 (2024)).

Change 15 (*Introduction*, lines 117–120)

- Added: "Similarly, while both the migration barrier and the Arrhenius prefactor govern ionic conductivity, SE design has predominantly targeted the former, with the latter receiving comparatively less attention."
- Reason: This sentence provides the background for Section 4 in the *Results* ("Anion dynamics tune conductivity through barrier and prefactor") and for the related design principle discussed in the *Discussion* (lines 604–605).

Change 16 (*Introduction*, lines 128–132)

- Original: "Machine learning is a powerful technique for extending the spatiotemporal scales ..."
- Revised: "Similarly, machine learning interatomic potentials extend simulations to larger system sizes ..."
- Reason: "Machine learning" is vague; we now specify "machine learning interatomic potentials."

Change 17 (*Introduction*, lines 132–138)

- Original: "Lattice dynamics, proposed as a descriptor long ago ... remain poorly understood."
- Revised: Deleted.
- Reason: Phonon–ion coupling represents another perspective on dynamic coupling between framework and mobile ion different from the local perspective adopted in this work. The passage mainly presented specific examples of studies on phonon–ion

coupling. We removed this passage to keep the *Introduction* focused on the scope of the present study.

Change 18 (*Introduction*, lines 151–155 and 167–170)

- Original: "Our work ... offers robust support for the growing body of research employing dynamic coupling as a design principle, ..."
- Revised: "These findings provide a design principle for superionic conductors exploiting polyanion dynamics."
- Reason: Replaced "dynamic coupling" with "polyanion dynamics" as the latter is the specific focus of this work, whereas the former encompasses many other types of coupling beyond the scope of this study.

Results

Subsection 2: Quantifying each polyanion motion mode's contribution to conductivity

This section has been substantially revised:

– Two key findings are distilled and explicitly stated (Change 19).
– Subsection title revised (Change 20).
– Figure revised (Change 22).
– Statements deleted or moved to SI (Change 24, Change 25, Change 27, Change 28).
– Imprecise or erroneous statement revised (Change 21, Change 23, Change 26).

Change 19 (key revision; *Results*, lines 222–224 and 258–264)

- Original: "Here, we identify the dominant anion motion mode that most significantly affects conductivity in each superionic conductor."
- Revised:
  (1) "Notably, the rotational constraint reduces the conductivity in every one of the six SEs, demonstrating that the long-debated paddle-wheel effect is universally operative, even though its enhancement is not always the largest among the three motion modes."

  (2) "Across these six SEs, all three motion modes (rotation, translation, and vibration) emerge as dominant for cation transport. This reveals that, in plastic crystals, polyanion reorientation does not necessarily dominate ionic conductivity; no single polyanion motion mode universally dominates cation transport."
- Reason:
  (1) The *Introduction* frames the paddle-wheel controversy at length, but the original text never explicitly addresses whether the paddle-wheel effect operates. Based on calculated conductivities we now explicitly state the conclusion.

  (2) The revised wording further emphasizes the key physical implication that polyanion reorientation does not necessarily dominate ionic conductivity in plastic crystals.

Change 20 (*Results*, subsection title, lines 172–174)

- Original: "Quantifying cation-anion coupling dynamics"
- Revised: "Quantifying each polyanion motion mode's contribution to conductivity"
- Reason: The original title is vague. "coupling dynamics" does not specify what is being quantified. The revised title highlights the key methodological contribution: the separate quantification of each motion mode's contribution to conductivity, echoing the motivation stated in the *Abstract* and *Introduction*.

Change 21 (*Results*, Fig.1, caption)

- Original: "(b) Unit cell of liquid-like phase of $Na_2B_{10}H_{10}$ ..."
- Revised: "(b) Unit cell of disordered phase of $Na_2B_{10}H_{10}$ ..."
- Reason: "Liquid-like" typically refers to the cation sublattice, not the phase, so the original use is inappropriate; the high-temperature phase of $Na_2B_{10}H_{10}$ is conventionally termed "disordered" (*Adv. Mater.*, **26**, 7622–7626, (2014)).

Change 22 (*Results*, Fig.1, content & caption)

- Added: Distinct orientations of the same polyanion species are now labeled (I) and (II); the two Na sites (Td, Oh) in $Na_2B_{10}H_{10}$ are now explicitly shown. The previous figure used exclusively to show the partially occupied sites (Figure S26) has therefore been removed. The caption is updated accordingly.
- Reason: The polyanion orientations are directly relevant to the rotational dynamics analysis (see Supplementary Information Section 3); the Na site distinction is essential for the later discussion of Na disordered in $Na_2B_{10}H_{10}$ (lines 513–519). This also makes the presentation more compact by showing the crystal structure and partially occupied Na sites of $Na_2B_{10}H_{10}$ in the same figure rather than in two separate figures.

Change 23 (*Results*, lines 175–177 and 229–233)

- Original: "To quantify how different types of anion framework with distinct reorientational mobility dynamically affect cation transport, ..."
- Revised: "To quantify the contribution of polyanion translation, rotation and vibration to cation transport in SEs, ..."
- Reason: The same as Change 2 (5)

Change 24 (*Results*, lines 178–181)

- Original: "In some cases, no net jump events occurred, or only limited ion diffusion was captured, with a total mean-squared displacement (MSD) of just a few Angstrom within 10 ns; calculating reliable values for conductivity was not feasible. Their MSD, shown in Figure S9–S12, fluctuate around a constant value for a long period."
- Revised: Deleted.
- Reason: MSD and mean square dipole displacement of cation are now provided as part of the uploaded raw data deposit rather than in the Supplementary Information.

Change 25 (*Results*, lines 199–202)

- Original: "Simulations of $LiBH_4$ with BH_4^- under RTVC were also conducted. The conductivity cannot be calculated in this case due to drastic decrease in conductivity resulting from the absence of anion translation; therefore, the contribution of BH_4^- vibration cannot be estimated. "
- Revised: Deleted.
- Reason: Although the RTVC simulation for $LiBH_4$ was included in the original manuscript, it does not provide an independent quantitative estimate of the contribution of BH_4^- vibration. Under RTC, Li^+ migration is already suppressed, so the additional vibrational constraint provides no further information. We therefore removed this non-informative discussion from the main text.

Change 26 (*Results*, lines 210–211)

- Original: "In thiophosphates, cation migration is sensitive to the translation and rotation of anions, ..."
- Revised: Deleted.
- Reason:
  (1) The claim does not hold uniformly (e.g., $Na_{11}Sn_2PS_{12}$ shows little "sensitivity" to translation (~0.15 orders of magnitude change in conductivity)).

  (2) The word "sensitive" is subjective.

Change 27 (*Results*, lines 218–222)

- Original: "Several studies have performed MD simulations with the anion sublattice fully fixed to evaluate the effect of anion rotation on cation migration.[15,31] However, this approach is ambiguous, as it cannot attribute the effect to a specific motion mode and may even lead to an incorrect physical interpretation. For instance, BH_4^- translation dominates Li diffusion in $LiBH_4$, despite its fast rotation."
- Revised: Deleted.
- Reason: This critique of prior methods belongs in the *Introduction*, not the *Results*; the section now focuses on presenting our own quantitative findings.

Change 28 (*Results*, lines 237–238)

- Added: "The detailed validation of the machine learning potential and the constraint method is presented in Section 1 and 4 of the Supplementary Information."
- Reason: The machine learning interatomic potential accuracy discussion (RMSE table, fitting accuracy, overfitting assessment) originally in the main text is now in SI. Also see Change 36.

Subsection 3: The paddle-wheel effect dominates only under frequency matching

This section has been substantially revised:

- Reorientation analysis revised: frequencies recalculated using the Ivanov model in place of event-counting; angular coordinates changed from $(\theta_x, \theta_y, \theta_z)$ to (θ, φ) (Change 29, Change 31, Change 34, Change 38, Change 42).
- New analyses added (Change 30, Change 45, Change 46, Change 47, Change 48).
- Key finding distilled and explicitly stated (Change 32).
- Subsection title revised (Change 33).
- Statements deleted or moved to SI (Change 35, Change 36, Change 41).
- Imprecise or erroneous statements revised (Change 37, Change 39, Change 40, Change 43, Change 44, Change 49).

Change 29 (key revision; *Results*, Fig.4d, content & caption)

- Original: Figure 2e showed reorientation frequencies obtained by phenomenological event-counting of reorientation events.
- Revised:

  (1) Frequencies are now calculated using the Ivanov model or via equation (6) (depending on the rotor type), rather than by phenomenological counting.

  (2) For $B_{10}H_{10}^{2-}$, two distinct reorientation modes are now resolved separately; for $P_2S_6^{4-}$, the reorientation and the internal rotation about the P–P bond are shown separately (solid vs. dashed lines).

  (3) The y-axis is changed to a logarithmic scale with units of ns^{-1} in place of GHz.

  (4) Temperature ranges for the three thiophosphates ($Na_{11}Sn_2PS_{12}$, $Na_4P_2S_6$, Li_3PS_4) are aligned to facilitate direct comparison.
- Reason for (1): Phenomenological event-counting depends on arbitrary spatial and temporal thresholds (what counts as a jump, how long the new site must be occupied). The Ivanov model removes this dependence by extracting the jump frequency as a fit parameter from the orientational correlation function. For near-continuous reorientation (e.g., BH_4^- in $LiBH_4$), where the site picture breaks down, we instead obtain the

frequency directly from the inverse of the orientational correlation time (equation (6)), which is also threshold-free.

- Reason for (2): The two modes in each system correspond to physically distinct motions and should not be lumped into a single frequency.
- Reason for (3): The reorientation frequencies span five orders of magnitude across the six SEs, which a linear scale cannot accommodate without an axis break; a logarithmic scale shows all rotor types on the same plot without distortion. It also resolves frequency differences within the low-frequency region (e.g., among the three thiophosphates), which appeared comparable on the original linear scale. The unit ns^{-1} (numerically identical to GHz) matches the simulation timescale.

Change 30 (key revision; *Results*, Fig.4e, content & caption)

- Added: Fig. 4e shows the activation energies (E_a) of anion reorientation obtained from Arrhenius fits of the frequencies in Fig. 4d. Reorientation angles fitted using the Ivanov model are indicated above the bars.
- Reason: Provides a quantitative comparison of reorientation barriers across all six SEs, connects the fitted angles to the frequency data in Fig. 4d, and underpins the subsequent design-principle discussion linking barriers and moments of inertia to the paddle-wheel effect.

Change 31 (key revision; *Results*, Fig.4f, content & caption)

- Original: Figure 2f showed cation hopping and anion reorientation frequencies both obtained by phenomenological event-counting.
- Revised: Fig. 4f now uses reorientation frequencies from the Ivanov model or equation (6), and cation hop frequencies estimated via equation (7) (unit and scale follow Fig. 4d).
- Reason: Same rationale as Change 29(1); event-counting depends on arbitrary thresholds. Fig. 4f is brought in line with Fig. 4d so that cation and anion frequencies are estimated with consistent, model-based methods.

Change 32 (key revision; *Results*, lines 341–345 and 372–375)

- **Original:** "It has been suggested that for the paddle-wheel mechanism to make a significant contribution to cation hopping, a similar event frequency between rotational motion and translational motion would be required. The mismatches between anion reorientational frequencies and cation jump frequencies contradict the hypothesis that large-angle anion rotations directly facilitate cation hopping through the docking-undocking process."
- **Revised:** "In $Na_2B_{10}H_{10}$ and $Na_{11}Sn_2PS_{12}$, the large-amplitude reorientations of the rotors (57–65° for $B_{10}H_{10}$, 74–107° for PS_4) occur at frequencies comparable to those of cation hop (Fig. 4f), in line with the dominant role of rotation in cation transport in these SEs (Fig. 2a,f). The paddle-wheel effect thus dominates conductivity only when these two frequencies coincide."
- **Reason:** These original passage reflects the earlier view that frequency matching determines whether a paddle-wheel effect exists. However, the modern use of the paddle-wheel effect refers to polyanion rotation facilitating cation migration (*Proc. Natl. Acad. Sci. U.S.A.* **122**, e2419892122 (2025)), and the quantitative results of the *Section* 2 show that constraining anion rotation lowers the ionic conductivity in all six systems. Frequency matching therefore does not determine the existence of the

effect, but only explains why rotation is the dominant motion mode in $Na_2B_{10}H_{10}$ and $Na_{11}Sn_2PS_{12}$.

Change 33 (*Results*, subsection title, lines 281–282)

- Original: "Anion rotation and constraint algorithm"
- Revised: "The paddle-wheel effect dominates only under frequency matching"
- Reason: The original title merely listed a phenomenon and a methodological tool without conveying any conclusion; the revised version is a declarative statement of the key finding.

Change 34 (*Results*, Fig.4a–c, content & caption)

- Original: Figure 2a–c showed angle projections using θ_x, θ_y and θ_z coordinates; (b) $Na_2B_{10}H_{10}$ at 650 K; (c) $Na_{11}Sn_2PS_{12}$ at 1050 K.
- Revised:
  (1) Angular coordinates changed from (θ_x, θ_y, θ_z) to polar and azimuthal angles (θ, ϕ); the main text is updated accordingly (lines 301–303, 346–348).
  (2) Data updated: (b) $Na_2B_{10}H_{10}$ now at 550 K; (c) $Na_{11}Sn_2PS_{12}$ now at 900 K.
  (3) In (b), apical B atoms are now shown in a distinct color (pink) to differentiate them from equatorial B atoms (green).
  (4) Caption updated accordingly.
- Reason: (θ, ϕ) enables direct visualization of reorientation via angular distribution projections on the (θ, ϕ) sphere (see Supplementary Information Section 3); the color distinction between apical and equatorial B atoms in (b) is needed for the subsequent discussion of two distinct reorientation modes of $B_{10}H_{10}$ (rotation about the apical–apical axis vs. reorientation of the axis itself).

Change 35 (*Results*, Fig.4, content & caption)

- Original: Figure 2d showed the angle projection of BH_4^- under rotational constraint, demonstrating successful implementation of the constraint algorithm.
- Revised: Moved to Supplementary Fig. 17; the associated text (lines 322–324) is moved to SI accordingly.
- Reason: The panel is methodological validation; we keep the main-text figure focused on findings.

Change 36 (*Results*, lines 285–293)

- Original: "The machine learning interatomic potentials ... Section 1 of the supplementary information."
- Revised: Deleted from the main text.
- Reason: Moved to SI. Also see Change 28.

Change 37 (*Results*, lines 306–307)

- Original: "transitions between different staggered conformations were observed for $P_2S_6^{4-}$ in $Na_4P_2S_6$ (Figure S5), but typically at frequencies below 0.05 ns^{-1} per rotor."
- Revised: The sentence and Figure S5 have been removed.

- Reason:
  (1) There is no direct evidence supporting transitions between different staggered conformations. Moreover, during the revision we performed an additional analysis using the Ivanov model, which yields a jump amplitude of $\sim$105°, consistent with reorientational jumps within a single staggered conformation rather than between distinct ones.

  (2) Figure S5 was only an illustration showing that dihedral-angle jumps occur in the simulations. Since the Ivanov model now provides a quantitative characterization of the internal rotation (frequency ν and jump amplitude Δ, Supplementary Table 7), the figure is no longer necessary and has been removed.

  (3) The stated frequency ($<0.05\,\mathrm{ns}^{-1}$) contradicts the values computed from trajectories of more than 100 ns during revision (Fig. 4d), where the internal-rotation frequency of $P_2S_6{}^{4-}$ exceeds 0.1 ns^{-1} and reaches $\sim$1 ns^{-1} at elevated temperatures.

Change 38 (*Results*, lines 321–322)

- Original: "Free energy surfaces for the rotation of three types of rotors of representative materials show a consistent trend (Figure S6)."
- Revised: Replaced by the Arrhenius barriers in Fig. 4e.
- Reason:
  (1) A free energy surface is a single-temperature, entropy-containing quantity, and the six systems were simulated at different temperatures, so the surfaces are not directly comparable. The Arrhenius barriers in Fig. 4e are temperature-independent, decouple the barrier from the prefactor.

  (2) For the slow rotors ($PS_4{}^{3-}$, $P_2S_6{}^{4-}$), the saddle-point region of the surface is poorly sampled because the transition state is rarely visited, making the barrier read off the surface unreliable; the Arrhenius fit uses the reorientation frequency directly and does not depend on sampling the transition state.

  (3) The chosen reaction coordinates (θ_x and θ_z, the angles with the x- and z-axis) do not span the full orientational space: rotations perpendicular to the xz plane are not captured, so the original free energy surface is inherently incomplete as a description of the reorientational landscape.

Change 39 (*Results*, lines 330–333 and 355–356)

- Original: "Note that the anion orientation transition frequency of thiophosphate differs from its rotational frequency. Specifically, its hindered rotation exhibits a significantly higher frequency, corresponding to decorrelation within hundreds of femtoseconds (Figure S7)."
- Revised: "Here, reorientation refers to large-amplitude jumps between preferred orientations, not the fast libration within a single orientation." Figure S7 was deleted accordingly.
- Reason: The libration frequency quantified in the original manuscript is not relevant to this work, which concerns only the quasi-permanent hops between potential energy minima that are relevant to the paddle-wheel effect (*Proc. Natl. Acad. Sci. U.S.A.* **122**, e2419892122 (2025)).

Change 40 (*Results*, lines 339–341, 372–373)

- Original: "A comparable match between the two frequencies is observed only in $Na_2B_{10}H_{10}$, as indicated by a previous study."

- Revised: "In $Na_2B_{10}H_{10}$ and $Na_{11}Sn_2PS_{12}$, the large-amplitude reorientations of the rotors (57–65° for $B_{10}H_{10}$, 74–107° for PS_4) occur at frequencies comparable to those of cation hop (Fig. 4f), ..."
- Reason: The frequency match, previously noted only for $Na_2B_{10}H_{10}$, is now established for both $Na_2B_{10}H_{10}$ and $Na_{11}Sn_2PS_{12}$. This result follows from the revised methodology: cation hop frequencies are obtained from equation (7) and reorientation frequencies from the Ivanov model, both replacing the threshold-based event counting used in the original manuscript.

Change 41 (*Results*, lines 314–316)

- Original: "For the BH_4^- rotor in lithium hydride and antiperovskite, the rotations are relatively free, and its rotation frequency is highly susceptible to temperature changes."
- Revised: Deleted.
- Reason:
  (1) 'Lithium hydride' (LiH) should read lithium borohydride ($LiBH_4$).

  (2) The simultaneous claims of "relatively free" rotation and "highly susceptible to temperature changes" are physically inconsistent. Specifically, "relatively free" rotation implies a low energy barrier, which in turn implies weak sensitivity to temperature changes. The activation energies quantified during revision (0.057 eV for BH_4^- reorientation in $LiBH_4$ and 0.072 eV for BH_4^- reorientation in Na_3OBH_4) are both low, further supporting the removal of this statement.

Change 42 (*Results*, lines 325–327, 365, and 369–370)

- Original: "We performed a statistical ion hopping analysis on each superionic conductor under UC, and the results, along with the corresponding rotor anion orientation transition frequencies, are presented in Figure 2f. "
- Revised: "We also evaluate the cation hop frequencies in the six SEs (equation (7)). ... Hop and reorientation frequencies are shown in Fig. 4f."
- Reason: The methodology has changed: cation hop frequencies are now obtained from equation (7) and reorientation frequencies from the Ivanov model, rather than phenomenological event counting (see Change 31 for the corresponding update to Fig. 4f). Wording implying event counting (e.g., "statistical ion hopping analysis") is removed accordingly.

Change 43 (*Results*, lines 327–328, 372–373)

- Original: "All thiophosphates exhibit cation jump frequencies that exceed their anion orientation transition frequencies."
- Revised: "In $Na_2B_{10}H_{10}$ and $Na_{11}Sn_2PS_{12}$, the large-amplitude reorientations of the rotors (57–65° for $B_{10}H_{10}$, 74–107° for PS_4) occur at frequencies comparable to those of cation hop ..."
- Reason: Based on the Ivanov model and equation (7), the revised analysis shows that the polyanion reorientation frequencies are comparable to the cation hop frequencies in both $Na_2B_{10}H_{10}$ and $Na_{11}Sn_2PS_{12}$.

Change 44 (*Results*, lines 328–330 and 370–371)

- Original: "This difference is particularly pronounced in Li_3PS_4, where the cation jump frequency is approximately four orders of magnitude higher than the anion orientation transition frequency."

- Revised: "The two frequencies differ by more than three orders of magnitude in Na_3OBH_4 and $LiBH_4$, and by at least two in $Na_4P_2S_6$ and Li_3PS_4."
- Reason: The original manuscript estimated reorientation frequencies by threshold-dependent phenomenological event counting, which yielded a gap of approximately four orders of magnitude for Li_3PS_4. Analysis based on the Ivanov model, performed during revision, provides a more rigorous and unified determination of the reorientation frequency, resulting in a difference of ~3 orders of magnitude in Li_3PS_4.

Change 45 (*Results*, lines 357–359)

- Added: "The reorientation frequencies span five orders of magnitude across the six SEs, from $\sim 10^3\,ns^{-1}$ in the fast rotors ($LiBH_4$, Na_3OBH_4) down to $\sim 10^{-2}\,ns^{-1}$ in the slowest (α-Li_3PS_4), with $Na_2B_{10}H_{10}$ falling in between."
- Reason: This sentence, based on the Ivanov model analysis introduced during revision, highlights that reorientation frequencies can differ by five orders of magnitude even among plastic crystals, and provides a quantitative characterization of the fast, moderate, and slow dynamics visualized in Fig. 4a–c.

Change 46 (*Results*, lines 359–363)

- Added: "Two types of rotation in ${B_{10}H_{10}}^{2-}$ were considered separately: rotation about the axis connecting the apical B atoms and reorientation of the axis itself. Both occur at comparable frequencies (pink dashed and solid lines in Fig. 4d). For ${P_2S_6}^{4-}$ in $Na_4P_2S_6$, internal rotation occurs at a lower frequency than the P–P bond reorientation (purple dashed and solid lines in Fig. 4d)."
- Reason: These sentences describe the distinct reorientation modes resolved during revision (see Change 29(2) for the corresponding update to Fig. 4d).

Change 47 (*Results*, lines 363–364)

- Added: "The angular distribution and its analysis are provided in Section 3 of the Supplementary Information."
- Reason: The sentence directs the reader to Section 3 of the Supplementary Information, where the full angular distributions and the temperature-dependent frequencies and jump amplitudes obtained from the Ivanov model fits are now provided.

Change 48 (*Results*, lines 376–377)

- Added: "Fig. 4e shows that the anion reorientation barrier increases from ~0.06 eV for ${BH_4}^{-}$ to ~0.1 eV for ${B_{10}H_{10}}^{2-}$, and reaches 0.7–0.9 eV for the P/S-based rotors."
- Reason: The reorientation barriers in Fig. 4e were newly added during revision (see Change 29); this sentence provides a comparative description of these barriers across the rotors.

Change 49 (*Results*, lines 314–316)

- Original: "The slow rotors, ${PS_4}^{3-}$ and ${P_2S_6}^{4-}$ anion groups, become orientationally disordered only at elevated temperatures (approximately 900 K) and exhibit slow transitions among preferred orientations."
- Revised: Deleted.
- Reason: The original sentence conflated a dynamical observation with a thermodynamic claim. An rotor polyanion can be orientationally disordered in the thermody-

namic sense while exhibiting reorientation events too rare to be captured within a MD simulation.

Subsection 4: Anion dynamics tune conductivity through barrier and prefactor

This section has been revised:

- Subsection title revised (Change 50).
- Figure revised: activation energies recomputed with longer trajectories; free-energy profile panel removed (Change 51).
- Conclusions updated to reflect revised data (Change 54, Change 55).
- Statements deleted (Change 52, Change 53).

Change 50 (*Results*, subsection title, lines 384–385)

- Original: "Correlating anion dynamics with migration enthalpy and prefactor"
- Revised: "Anion dynamics tune conductivity through barrier and prefactor"
- Reason:
  (1) The original title used "migration enthalpy," which was inconsistent with the body text where only "activation energy" and "energy barrier" were used throughout, both before and after revision, so the revised title corrects this inconsistency by adopting "barrier" instead.

  (2) The shift from "correlating" to "tune" elevates anion dynamics from a passive correlation to an active governing role.

Change 51 (*Results*, Fig.5, content & caption)

- Original: Figure 4a: Activation energies derived from linear Arrhenius fits. Figure 4b: Free-energy profiles from the 900 K $Na_{11}Sn_2PS_{12}$ trajectory, plotted against the Na–PS_4 center-of-mass distance.
- Revised: (a) Recomputed using trajectories of over 100 ns produced during revision. (b) Deleted. The relevant content in the main text (line 387) is also removed.
- Reason: The activation energy of cation migration is a temperature-independent effective barrier and is more commonly reported and discussed in this field, both experimentally and theoretically. In constrast, a free-energy profile is a single-temperature quantity; because different systems were simulated at different temperatures, these profiles are not directly comparable and were removed.

Change 52 (*Results*, lines 391–394)

- Original: "The conductivity (or diffusion coefficient) reflects the collective ion diffusion characterized by Brownian motion, whereas ion migration in solids can be described by absolute rate theory, where activation energy governs both the formation and migration of mobile ions."
- Revised: Deleted.
- Reason: It states textbook-level concepts that do not belong in the *Results* section.

Change 53 (*Results*, lines 404–406)

- Original: "Note that the quantitative accuracy of the barriers should not be overemphasized;"
- Revised: Deleted.

- Reason: This sentence is more of a disclaimer than a scientifically grounded statement.

Change 54 (*Results*, lines 407–409 and 430–432)

- Original: "However, an exception is observed when the fast rotation of BH_4^- in $LiBH_4$ is constrained, reducing the conductivity by about one order of magnitude (Figure 3b), with a simultaneous lowering of activation energy."
- Revised: "In contrast, in $LiBH_4$, constraining BH_4^- rotation, decreases the conductivity by ∼1 order of magnitude, despite very small increases in E_a of only 0.0246 eV."
- Reason: Updated data from longer MD trajectories show that E_a slightly increases (by 0.0246 eV) rather than decreases upon constraining BH_4 rotation, reversing the original trend. The revised statement corrects this and replaces the qualitative description with an explicit value.

Change 55 (*Results*, lines 425–427)

- Original: "This is also consistent with the Meyer-Neldel rule, as the fast rotation of BH_4^- increases both the barrier accompanied by an increase in prefactor, and the combined effect results in higher conductivity."
- Revised: Deleted.
- Reason: As noted in Change 54, updated data show that BH_4^- rotation lowers rather than raises the activation barrier, contradicting the premise of the original Meyer-Neldel argument (that rotation increases both barrier and prefactor). This argument is therefore deleted.

Subsection 5: Anion dynamics drive superionicity via coupling and disorder

This section has been substantially revised:

- Figures streamlined and reorganized (Change 57, Change 59, Change 60, Change 61, Change 62).
- New analyses added: migration barrier from metadynamics, disorder analysis, and anharmonicity discussion (Change 56, Change 58, Change 64, Change 68).
- Statements removed or moved to SI (Change 63, Change 65, Change 66, Change 67, Change 69, Change 70, Change 71, Change 72).

Change 56 (key revision; *Results*, Fig.6c, content & caption)

- Original: No corresponding panel was included in the original Figure 6.
- Revised: A new Fig. 6c was added to show the free-energy profile of Li^+ migration in $LiBH_4$, obtained from well-tempered metadynamics under three constraint conditions at 300 K. The caption was added accordingly. A corresponding discussion of the migration barriers was also added in the main text (lines 484–492).
- Reason: This new panel provides direct quantitative evidence that BH_4^- translation lowers the Li^+ migration barrier, supporting the revised mechanistic discussion.

Change 57 (key revision; *Results*, Fig.6d, content & caption)

- Original: No corresponding panel was included in the original Figure 6.
- Revised: A new Fig. 6d was added to schematically illustrate the Li^+ migration energy landscape in $LiBH_4$ with BH_4^- translation constrained and unconstrained. The caption was correspondingly added. A corresponding description of the schematic energy landscape was also added in the main text (lines 490–492).

- Reason: This schematic visually summarizes the key mechanism established in Fig. 6a–c, helping readers quickly grasp how BH_4 translation enhances anharmonicity and lowers the migration barrier.

Change 58 (key revision; *Results*, Fig.6e, content & caption)

- Original: Figure 6d showed the Na^+–Na^+ PDF under different constraints. Figure 6e–f showed the Na^+ probability densities in $Na_2B_{10}H_{10}$ with and without $B_{10}H_{10}{}^{2-}$ rotation
- Revised: Figure 6d–f deleted. A new Fig.6e was added, showing the Oh site occupancy of Na^+ under UC and RC at different temperatures. The main text (lines 511–517) and caption are updated accordingly.
- Reason: The Oh site occupancy data directly and quantitatively demonstrate that $B_{10}H_{10}{}^{2-}$ rotation induces Na^+ sublattice disorder, whereas the original PDF and probability density plots were open to interpretation and could serve as auxiliary support for this conclusion at most.

Change 59 (*Results*, Fig.6a, content & caption)

- Original: Figure 6a showed the statistical distribution of Li ions along the c axis in $LiBH_4$ over a relatively broad coordinate range, where two repeated sets of split-site features were displayed and labeled as SS1 and SS2.
- Revised: Fig. 6a was simplified to focus on one representative pair of Li split sites along the c axis. The caption was also revised to explicitly define SS1 and SS2 as split site 1 and split site 2, respectively.
- Reason: This revision makes the key feature of Li-site splitting clearer and avoids redundant visual information.

Change 60 (*Results*, Fig.6b, content & caption)

- Original: Figures 6b,c showed the Li probability densities in $LiBH_4$ with and without $BH_4{}^-$ translation in a supercell.
- Revised: These two panels were combined into the revised Fig. 6b, and the Li probability densities are now shown in a unit cell.
- Reason: A unit cell is sufficient to show the contrast in Li probability densities, and the supercell view did not provide additional information.

Change 61 (*Results*, Fig.6, content & caption)

- Original: The original Figure 6g–i showed the Li^+–Li^+ PDF in Li_3PS_4 and the corresponding Li^+ probability densities with and without $PS_4{}^{3-}$ vibration.
- Revised: The original Figure 6g was moved to Supplementary Fig. 28, while the original Figure 6h,i were removed from the main figure. The caption was revised accordingly.
- Reason: The Li^+–Li^+ PDF is sufficient to show the gradual opening of Li^+ transport channels in Li_3PS_4, whereas the probability density plots mainly provide additional visual support. Removing these auxiliary panels makes Fig. 6 more focused on the direct mechanistic evidence in $LiBH_4$ and $Na_2B_{10}H_{10}$.

Change 62 (*Results*, Fig.7, content & caption)

- Original: The original Figure 5 showed the total phonon DOS and the partial phonon DOS of mobile cations in $LiBH_4$, $Na_2B_{10}H_{10}$, and Li_3PS_4.

- Revised: This figure was renumbered as Fig. 7. The $LiBH_4$ and $Na_2B_{10}H_{10}$ panels were retained, while the Li_3PS_4 panel was replaced by $Na_4P_2S_6$ at 800 K. The caption was revised accordingly, and the partial phonon DOS of mobile cations in Li_3PS_4 was moved to Supplementary Fig. 27a.
- Reason: The revised figure better supports the updated discussion on thiophosphate-based solid electrolytes by using $Na_4P_2S_6$ as a clearer main-text example showing the strong sensitivity of the partial phonon DOS of mobile cations to anion dynamics, while retaining Li_3PS_4 as supporting evidence in the Supplementary Information.

Change 63 (*Results*, lines 439–441)

- Original: " The phonon DOS calculated from MD trajectories, which naturally accounts for anharmonicity to all orders, can effectively capture the dynamics of the system or be atom-projected to extract cation dynamics."
- Revised: Deleted.
- Reason: Redundant.

Change 64 (*Results*, lines 477–481)

- Added: "This demonstrates that BH_4 translation enables Li to explore off-centered positions over a broader local region, giving rise to a flat-bottomed local free-energy landscape for Li. In contrast to the single-peaked distribution under RTC, the split distribution shows that BH_4 translation enhances the anharmonicity of the local potential experienced by Li."
- Reason: During revision, we revisited the split-site distribution of Li (Fig.6a,b) and extracted a clearer physical picture: the Li distribution changes from single-peaked to double-peaked when BH_4 translation is present, indicating a flat-bottomed local free-energy landscape and enhanced anharmonicity.

Change 65 (*Results*, lines 459–460)

- Original: "The influence of anion translation can also be interpreted by mechanism. MD is a good approach for detecting underlying ion transport mechanism."
- Revised: Deleted.
- Reason: This background was removed to make the discussion more direct.

Change 66 (*Results*, lines 467–468)

- Original: "Another piece of evidence is the pair distribution function (PDF) of Li^+–Li^+ (Figure S25a), where the SS1–SS2 distribution disappears under RTC."
- Revised: Deleted. The corresponding Li^+–Li^+ PDF was also removed from the SI.
- Reason: The disappearance of the double splitting under RTC is already directly evidenced by the Li^+ distribution in Fig. 6a and visualized by probability-density analysis in Fig. 6b. The PDF provided provides no additional information.

Change 67 (*Results*, lines 501–503)

- Original: "Similar to $LiBH_4$, the vibrations of the ${B_{10}H_{10}}^{2-}$ anion have minimal effect on the Na^+ phonon DOS in $Na_2B_{10}H_{10}$ (Figure 5b). Anion translation leads to more diffuse Na^+ phonon DOS."
- Revised: Deleted.

- Reason: The revised sentence focuses on anion rotation as the key motion mode and omits the discussion of other anion motion modes.

Change 68 (*Results*, lines 511–513)

- Original: No corresponding statement was included in the original text.
- Revised: "Partial occupancy of Na sites and the presence of multiple possible occupation patterns among the eight Td sites surrounding an occupied Oh site strongly indicate disorder in the Na sublattice."
- Reason: This sentence was added to provide a clearer basis for identifying the Na sublattice as disordered before presenting our occupancy analysis. It strengthens the logical transition from known site-occupancy features in $Na_2B_{10}H_{10}$ to our conclusion that anion rotation induces cation disorder.

Change 69 (*Results*, lines 528–529)

- Original: "Li^+ motion is coupled with all ${PS_4}^{3-}$ motion modes"
- Revised: Deleted.
- Reason: This conclusion was too broad and not very informative, since some degree of coupling is expected in such systems.

Change 70 (*Results*, lines 422–425)

- Original: "Here, applying constraints to anion dynamics dramatically affects the cation phonon DOS. In different materials, the anion motion mode that dominates the cation phonon DOS also has the most significant effect on conductivity."
- Revised: Deleted.
- Reason: This general claim does not hold for all materials studied (e.g., Li_3PS_4 and $Na_{11}Sn_2PS_{12}$), so it was removed to avoid overreaching.

Change 71 (*Results*, lines 416–422)

- Original: "Klerk et al. proposed that the attempt frequency can be approximated by the band center of the mobile cation's phonon density of states (DOS). The calculated phonon DOS and cation band center are shown in Figure 5a. The rotational constraint (absence of anion rotation) softens the Li^+ sublattice in $LiBH_4$, resulting in a decrease in the attempt frequency, which can lead to a decrease in prefactor; the Li^+ phonon band center exhibits a consistent trend across an extended temperature range, as illustrated in Figure S18b."
- Revised: Deleted.
- Reason: The band center of the cation phonon DOS reflects the overall spectral weight, but the attempt frequency corresponds to the frequency of mode(s) with projection along the migration pathway. A global softening of the spectrum does not establish that the relevant mode softens. The conclusion that the attempt frequency decreases therefore does not follow from the observed shift in band center.

Change 72 (*Results*, lines 551–563)

- Original: "Across the entire simulated temperature range, we find that anion dynamics lower the cation band center value, effectively softening the cation sublattice (Figure S18). The cation band center was correlated with migration enthalpy[90] and utilized as a descriptor to identify promising ionic conductors.[91] Here, the anion dynamics

facilitate a softer cation sublattice and thus lower migration enthalpy. We calculate the difference in cation band center value between UC and RTVC, as shown in Figure S28. However, ${B_{10}H_{10}}^{2-}$ dynamics have a much smaller softening effect on the Na^+ sublattice in $Na_2B_{10}H_{10}$, yet dominate ion migration (Figure 3a,f). On one hand, the cation band center represents an averaged lattice vibration property that characterizes the lattice softness, i.e., the interactions strength between atoms. On the other hand, certain anion motion modes, though not significantly softening the cation sublattice, couple with cation motions that are essential for migration. Identifying these anion dynamics, as the constraint algorithm enables us to do here, and selectively exciting them provide fundamental insights and enable high ion mobility."

- Revised: Deleted. Figure S28 was also deleted.
- Reason: The cation band center is not a sufficiently robust descriptor for ion transport: even in the original reference (*Energy Environ. Sci.* **11**, 850 (2018)), migration enthalpy varies from 0.15 to 0.6 eV within a narrow band center range (∼38–41 meV). Building a discussion around this descriptor therefore does not yield reliable conclusions, and the paragraph was removed.

Discussion

The original *Discussion* largely summarized material-specific observations. It has been substantially rewritten around the broader conclusions and design principles that emerge from the revised *Results*.

Change 73 (*Discussion*, lines 573–581 and 592–596)

- Original: "We find that anion rotation can enhance ionic conductivity, but not necessarily through the docking-undocking paddle-wheel mechanism, ... In contrast, in α-Li_3PS_4, anion vibration plays a significant role in cation diffusion."
- Revised: "Although the paddle-wheel effect is universally operative in plastic crystals, no single anion motion mode universally dominates cation transport. Anion translation and vibration can also dominate, and even govern, cation transport. This demands a broader view of cation–anion coupling, beyond the long-standing focus on rotation as the most visually compelling mechanism of cation hopping."
- Reason: During revision, the central conclusion was updated: the paddle-wheel effect is recognized as universally operative in plastic crystals, but rotation is not universally the dominant motion controlling cation transport. The *Discussion* was therefore rewritten to reflect this revised conclusion. The revised text distills the material-specific observations into a clearer *Discussion*-level conclusion.

Change 74 (*Discussion*, lines 569–572 and 599–605)

- Original: "We find that anion dynamics are of paramount importance for enabling superionicity, especially at lower temperatures, both by inducing fluctuations in the underlying energy landscape, thereby effectively lowering the energy barrier, and by altering local cation dynamics, which directly affects the prefactor."
- Revised: "To understand the origin of these conductivity changes, we examine the activation barrier and the prefactor. Lowering the activation barrier has long been a central strategy in the design of SEs, and we find that anion dynamics can substantially modify this barrier. However, anion dynamics can also alter conductivity substantially without significantly changing the activation barrier, indicating that the prefactor, likely through the attempt frequency and migration entropy, is comparably important. These findings call for SE design strategies that target both the activation barrier and the prefactor."
- Reason: The original sentence mentioned barrier lowering and prefactor changes only briefly. The revised text expands this point into a clearer *Discussion*-level conclusion: anion dynamics can regulate ion transport through both the activation barrier and the prefactor, so SE design should target both contributions rather than focusing only on barrier reduction.

Change 75 (*Discussion*, lines 582–589 and 609–614)

- Original: "These dominant anion motion modes influence cation motion by inducing disorder within the cation sublattice, and their effect on ion mobility becomes more prominent at lower temperatures. Besides the dynamic disorder quantified here, other effects, such as defects, are also likely to be crucial in affecting mobility, and their investigation is expected to be a focus of our future research. This study provides a fundamental understanding of anion-cation coupling dynamics ..."
- Revised: "Finally, anion dynamics can induce dynamic cation disorder, which alone can enhance conductivity by over two orders of magnitude. This disorder takes two

distinct forms: anharmonic broadening of cation positions around their lattice sites, and partial occupancy across multiple crystallographic sites. Both differ fundamentally from site disorder. This suggests that SE design should target the dynamic anion–cation couplings that activate such disorder, opening additional pathways for fast ion transport."

- Reason: The revised text reflects the updated *Results* section, where dynamic cation disorder became a central mechanism rather than a brief concluding statement. It also makes the conclusion more specific by distinguishing two forms of dynamic disorder and formulates a clearer design principle: SE design should target dynamic anion–cation couplings that activate such disorder. The previous caveat about defects was removed because a dedicated defect-physics analysis has been carried out and incorporated into the revised manuscript.

Methods & Supplementary Information

Change 76 (*Methods* & *SI*, Table S1; *Methods*, lines 657–661)

- Original: Table S1
- Revised: Table S1 is deleted. The MD settings are now specified in the *Methods* section: "For Na_3OBH_4, six 20 ns simulations with different initial velocities were performed for each temperature and constraint using a $7 \times 7 \times 7$ supercell containing 3087 atoms, in which 12 Na vacancies were introduced. For $Na_2B_{10}H_{10}$, $LiBH_4$, Li_3PS_4, $Na_4P_2S_6$, and $Na_{11}Sn_2PS_{12}$, three independent 50 ns simulations were performed in supercells containing 1056, 768, 864, 648 and 832 atoms, respectively."
- Reason: The main text focuses on findings instead of methodology.

Change 77 (*Methods*, lines 681–692)

- Added: "The average residence times and jump amplitudes associated with the reorientation of ${PS_4}^{3-}$ and ${P_2S_6}^{4-}$, as well as with the reorientation of the apical B–B axis of ${B_{10}H_{10}}^{2-}$, are extracted by fitting the Ivanov model.[103] Specifically, the first- and second-rank orientational correlation functions were fitted as

$$C_1(t) = \exp\left[-\frac{t}{\tau}\left(1 - \cos\Delta\right)\right], \tag{6}$$

and

$$C_2(t) = \exp\left[-\frac{3t}{2\tau}\left(1 - \cos^2\Delta\right)\right], \tag{7}$$

where τ is the average residence time between two jumps and Δ is the jump amplitude. The corresponding reorientation frequencies are then obtained as the inverse of the average residence time. The reorientation frequencies ν_{rot} of ${BH_4}^{-}$, and of ${B_{10}H_{10}}^{2-}$ about its apical B–B axis, are evaluated directly as the inverse of the orientational correlation time[104]

$$\nu_{rot} = \left(\int_0^\infty \langle P_1(\cos[\theta(t)])\rangle\, dt\right)^{-1}. \tag{8}$$

where P_1 is the first-order Legendre polynomial, and $\theta(t)$ is the angle between a given B–H bond at time t and at $t = 0$. The cation hop frequency ν_{hop} is estimated by

$$\nu_{hop} = \frac{6D_{tr}}{\lambda^2} \tag{9}$$

λ is the distance between nearest cations in the equilibrium structure."
- Reason: To clarify how the reorientation and cation-hop frequencies reported in the manuscript were defined and obtained.

Change 78 (*Methods*, lines 697–707)

- Added: "Well-tempered metadynamics[106] simulations were performed to obtain the free energy profile of Li-ion migration in $LiBH_4$. Three nearest-neighbor Li sites along the c-axis were considered and labeled I, II, and III. In the initial configuration, sites II and III are occupied by two Li ions, denoted Li(II) and Li(III) after their starting sites, while site I is vacant. The migration consists of a concerted displacement of Li(II) and Li(III) as a rigid pair, in which Li(II) moves into site I and Li(III) into site II, leaving site III vacant. The two end states are related by symmetry and the process is reversible, so the resulting free energy profile is symmetric. The surrounding ${BH_4}^{-}$

were used as an internal reference frame. The collective variable was defined as

$$d(\text{site(II)}, \text{Li(II)}) + d(\text{site(III)}, \text{Li(III)}) - d(\text{site(I)}, \text{Li(II)}) - d(\text{site(II)}, \text{Li(III)})$$

where $d(A, B)$ denotes the distance between A and B. The height of Gaussian is 0.0008 Ha, and the bias temperature ΔT is 4500 K."

- Reason: To provide a detailed description of the metadynamics settings in this study.

Change 79 (*SI*, lines 26–44)

- Added: First, the reorientation of these anions in six SEs was well reproduced. ... The very low BH_4 rotational barrier observed here is consistent with the nearly free BH_4 rotation reported by Sun et al. for Na_3OBH_4.[7]
- Reason:
- Reason: To validate the MLIPs for the six studied systems by comparing the calculated anion reorientation dynamics and rotational barriers with previously reported AIMD, QENS, and experimental results.

Change 80 (*SI*, lines 47–53; Supplementary Fig.2)

- Original: "For $Na_2B_{10}H_{10}$ and $LiBH_4$, conductivity measurements via impedance spectroscopy are available only below 423 K[4, 10], while those calculated by AIMD, typically at very high temperatures ($\geq$ 1000 K)[11], are of limited practical significance. Here, we compare their experimental conductivities with the theoretical conductivities obtained from our simulations at similar temperatures. The ionic conductivities of $Na_2B_{10}H_{10}$ and $LiBH_4$ are reported as 0.0259 $\mathrm{S\,cm^{-1}}$ at 423 K[4] and 0.005 $\mathrm{S\,cm^{-1}}$ at 423 K,[10] respectively. These values are in close agreement with our calculated values of 0.0263 $\mathrm{S\,cm^{-1}}$ at 450 K for $Na_2B_{10}H_{10}$ and 0.008 $\mathrm{S\,cm^{-1}}$ at 450 K for $LiBH_4$."
- Revised: "For $Na_2B_{10}H_{10}$ and $LiBH_4$, conductivity measurements via impedance spectroscopy are available below 423 K. The ionic conductivities of $Na_2B_{10}H_{10}$ and $LiBH_4$ are reported to be 0.0259 $\mathrm{S\,cm^{-1}}$ at 423 K and 0.005 $\mathrm{S\,cm^{-1}}$ at 423 K, respectively. These values are in agreement with our extrapolated/interpolated values (Supplementary Fig.2): 0.0206 $\mathrm{S\,cm^{-1}}$ for $Na_2B_{10}H_{10}$ and 0.0028 $\mathrm{S\,cm^{-1}}$ $LiBH_4$. For Na_3OBH_4, the experimental conductivity does not match the result obtained here, likely because the simulation uses a lower Na-vacancy concentration than that inferred from XRD."
- Reason: To validate the MLIPs by comparing the calculated conductivities of $Na_2B_{10}H_{10}$ and $LiBH_4$ with experimental values using extrapolated/interpolated results, and by comparing the calculated conductivity of Na_3OBH_4 with experiment.

Change 81 (*SI*, new section, pages 8–12)

- Added: A new section, "Rotation Dynamics Statistics", describing how angular distributions were computed by tracking the same individual bond in rotors with the same orientation. The section presents angular distributions for all six SEs, discusses the multiple preferred orientations and their consistency with crystal symmetry, and provides a complete list of the reorientation frequencies and jump amplitudes obtained by fitting the Ivanov model.
- Reason: To provide a complementary perspective on rotor dynamics beyond the reorientation frequencies discussed in the main text, by statistically characterizing the preferred orientational states and crystal-symmetry-dependent angular distributions of the rotors in the six studied systems.

Change 82 (*SI*, Figures S13–S14 and Supplementary Fig.S20–S26)

- Revised: The change of conductivity under different constraint conditions was updated by including conductivities calculated using both the Nernst–Einstein and Einstein–Helfand relations. The updated results are also based on longer MD trajectories.
- Reason: To provide a more reliable assessment of the conductivity changes under different constraint conditions by using longer trajectories and comparing two conductivity evaluation methods.

Change 83 (*SI*, Figure S16)

- Original: Figure S16 showed the Arrhenius plots of the calculated tracer diffusion coefficient under different constraints.
- Revised: Deleted.
- Reason: The figure was removed to avoid redundancy, as the underlying MSD data used to calculate the diffusion coefficients under different constraints are now provided directly.

Change 84 (*SI*, Figures S19–S24 and Supplementary Fig.S27)

- Original: Figures S19–S24 showed the phonon DOS of the six materials over selected temperature ranges, while the main text also included phonon DOS plots for three of these materials at representative temperatures.
- Revised: Only one representative-temperature phonon DOS is retained for each material, with three shown in the main text and three shown in the Supplementary Information.
- Reason: To avoid redundant phonon DOS plots at multiple temperatures and to make the presentation more concise while still providing representative phonon DOS results for all six materials.

Change 85 (*SI*, Figure S25)

- Original: Figure S25 showed the pair distribution functions for $LiBH_4$, $Na_4P_2S_6$, and $Na_{11}Sn_2PS_{12}$ under different constraint conditions.
- Revised: Deleted.
- Reason: The figure was removed because it did not provide substantial additional insight.

Appendix

In a solid electrolyte, mobile ions diffuse within a rigid lattice framework. Eq (3) can be partitioned into carrier and framework contributions.

$$
\begin{aligned}
\sigma &= \frac{1}{3\Omega k_B T}\lim_{t\to\infty}\frac{1}{2t}\left\langle \left[\sum_i^{N_C} q_i\Delta\mathbf{r}_i(t) + \sum_i^{N_F} q_i\Delta\mathbf{r}_i(t)\right]^2\right\rangle \\
&= \frac{1}{3\Omega k_B T}\lim_{t\to\infty}\frac{1}{2t}\left\langle \left(\sum_i^{N_C} q_i\Delta\mathbf{r}_i(t)\right)^2\right\rangle + \frac{1}{3\Omega k_B T}\lim_{t\to\infty}\frac{1}{2t}\left\langle \left(\sum_i^{N_F} q_i\Delta\mathbf{r}_i(t)\right)^2\right\rangle \\
&+ \frac{1}{3\Omega k_B T}\lim_{t\to\infty}\frac{1}{2t}\left\langle 2\left(\sum_i^{N_C} q_i\Delta\mathbf{r}_i(t)\right)\cdot\left(\sum_i^{N_F} q_i\Delta\mathbf{r}_i(t)\right)\right\rangle \\
&\equiv \sigma_C + \sigma_F + \sigma_{cross}
\end{aligned}
\tag{10}
$$

where N_C and N_F are the numbers of charge carrier and framework particles, respectively.
Carrier Term(σ_C): For the diffusing particles, the mean square displacement(MSD) is proportional to t in the long time limit (*npj Comput. Mater.* **4**, 18 (2018)). Thus, the time derivative converges to a non-zero constant, representing the carrier contribution to the total conductivity.
Framework Term(σ_F): The framework particles do not diffuse; their motion is vibrational and bounded. Therefore, the time derivative of their MSD vanishes (approaches zero) in the long-time limit.
Cross Term(σ_{cross}): This ensemble average of dot product of two observables can be formally treated as an inner product. Based on this, by applying Cauchy-Schwarz inequality, we get

$$
\left|\left\langle \left(\sum_i^{N_C} q_i\Delta\mathbf{r}_i(t)\right)\cdot\left(\sum_i^{N_F} q_i\Delta\mathbf{r}_i(t)\right)\right\rangle\right| \le \sqrt{\left\langle (\sum_i^{N_C} q_i\Delta\mathbf{r}_i(t))^2\right\rangle\left\langle (\sum_i^{N_F} q_i\Delta\mathbf{r}_i(t))^2\right\rangle} \sim t^{\frac{1}{2}}
$$

We conclude that the derivative of the cross term decays to zero in the long time limit.

Thus, the conductivity of a solid electrolyte calculated from the Einstein-Helfand relation, which encompasses ion-ion correlation, gives an exact result as long as the first term in eq (4) is evaluated. This is in consistent with the description of the phenomenological theory of irreversible processes (Helmut M. *Diffusion in solids: fundamentals, methods, materials, diffusion-controlled processes* pages 191–205 (Berlin, Heidelberg: Springer Berlin Heidelberg, 2007)). Given that the framework atoms are non-diffusive, the flux of the corresponding framework atoms must be zero under isothermal conditions. This indicates that any elements in the Onsager matrix related to framework atoms must be zero, which further implies that the thermodynamic forces from the framework atoms have no effect on the flux of mobile ions.

Reviewer #1 (Remarks to the Author):
We thank the Authors for their careful revision of the manuscript. In our view, the revised version has substantially improved with respect to the previous submission, both in terms of clarity and in the presentation of the numerical results. However, before we can suggest publication in *Nature Communications*, the author need to address satisfactorily the following remaining issues.

We are glad that the reviewer finds the revised manuscript improved, and we thank them for the detailed and constructive comments. In brief: while the reference frame was not clearly explained in the previous version, the conductivity was computed in the solid-matrix reference frame; we have now stated this explicitly. As suggested by the reviewer, we have also adopted the degree of anharmonicity to provide quantitative support for our conclusions. Finally, we have carried out a detailed analysis of the machine learning interatomic potential, confirmed its accuracy, and made the dataset available. We hope these revisions resolve the reviewer's remaining concerns.

1 Flaws in the Appendix of the Authors' response

We respectfully disagree with the statement that, in a crystalline solid electrolyte with a non-diffusive framework, the framework self term and the carrier–framework cross term in the Einstein–Helfand expression necessarily vanish in the long-time limit. The conclusion is correct only after transforming to the framework (i.e., solid-matrix) reference frame. It does not follow in the barycentric reference frame, which is the reference frame usually adopted in equilibrium MD simulations.

Let the Einstein–Helfand charge displacement be written, in the barycentric frame, as

$$\Delta\boldsymbol{\mu}^{\mathrm{bar}}(t) = \sum_{i\in C} q_i \Delta\mathbf{r}_i^{\mathrm{bar}}(t) + \sum_{j\in F} q_j \Delta\mathbf{r}_j^{\mathrm{bar}}(t) \equiv \mathbf{A}_C^{\mathrm{bar}}(t) + \mathbf{A}_F^{\mathrm{bar}}(t), \tag{1}$$

where C denotes the mobile carriers and F the framework atoms. The conductivity is then

$$\sigma = \frac{1}{3\Omega k_B T} \lim_{t\to\infty} \frac{1}{2t} \left\langle \left| \mathbf{A}_C^{\mathrm{bar}}(t) + \mathbf{A}_F^{\mathrm{bar}}(t) \right|^2 \right\rangle . \tag{2}$$

The argument in the manuscript assumes that $\mathbf{A}_F^{\mathrm{bar}}(t)$ is bounded because the framework atoms are non-diffusive. This is the step that is not generally valid.

Indeed, non-diffusivity of the framework implies bounded displacements only with respect to the solid matrix, *not necessarily in the barycentric frame.* Writing

$$\Delta\mathbf{r}_j^{\mathrm{bar}}(t) = \Delta\mathbf{R}_F^{\mathrm{bar}}(t) + \Delta\mathbf{u}_j(t), \tag{3}$$

where $\Delta\mathbf{R}_F^{\mathrm{bar}}(t)$ is the displacement of the framework center of mass and $\Delta\mathbf{u}_j(t)$ is the displacement of atom j relative to the framework, only $\Delta\mathbf{u}_j(t)$ is bounded for a non-diffusive framework. Therefore text

$$\mathbf{A}_F^{\mathrm{bar}}(t) = \sum_{j\in F} q_j \Delta\mathbf{r}_j^{\mathrm{bar}}(t) = Q_F \Delta\mathbf{R}_F^{\mathrm{bar}}(t) + \sum_{j\in F} q_j \Delta\mathbf{u}_j(t), \tag{4}$$

with

$$Q_F = \sum_{j \in F} q_j. \tag{5}$$

The second term in Eq. (4) is bounded, but the first one is not generally bounded. In a barycentric frame the total center of mass is fixed, so that

$$M_C \Delta \mathbf{R}_C^{\mathrm{bar}}(t) + M_F \Delta \mathbf{R}_F^{\mathrm{bar}}(t) = 0, \tag{6}$$

where M_C and M_F are the total masses of the carrier and framework subsystems, respectively. Hence

$$\Delta \mathbf{R}_F^{\mathrm{bar}}(t) = -\frac{M_C}{M_F} \Delta \mathbf{R}_C^{\mathrm{bar}}(t). \tag{7}$$

Thus, if the carrier subsystem has a diffusive collective displacement, the *framework center of mass recoils accordingly.* The framework atoms *remain non-diffusive relative to the matrix*, but the *collective barycentric displacement* of the framework is not generally bounded.

Consequently,

$$\left\langle \left| \mathbf{A}_F^{\mathrm{bar}}(t) \right|^2 \right\rangle \tag{8}$$

need not be $O(t^0)$ at long times. In particular, when $Q_F \neq 0$, as is the case when the framework carries the charge compensating that of the mobile carriers, the first term in Eq. (4) may have diffusive scaling. Therefore the Cauchy–Schwarz estimate used in the manuscript does not imply

$$\lim_{t \to \infty} \frac{1}{2t} \left\langle \mathbf{A}_C^{\mathrm{bar}}(t) \cdot \mathbf{A}_F^{\mathrm{bar}}(t) \right\rangle = 0. \tag{9}$$

The cross term can instead give a finite contribution, because both $\mathbf{A}_C^{\mathrm{bar}}(t)$ and the collective recoil part of $\mathbf{A}_F^{\mathrm{bar}}(t)$ may scale as $t^{1/2}$. This is indeed what was observed in Sec. III.E of Ref. [GTGC24], and detailed, for the cross-correlation term between the Li^+ carriers and the thiophosphate matrix, in Fig. S13 of its Supporting Information.

The correct statement is therefore reference-frame dependent. If one transforms to the solid-matrix frame,

$$\mathbf{v}_i^{\mathrm{mat}} = \mathbf{v}_i^{\mathrm{bar}} - \mathbf{w}_F^{\mathrm{bar}}, \qquad \mathbf{w}_F^{\mathrm{bar}} = \frac{\sum_{j \in F} m_j \mathbf{v}_j^{\mathrm{bar}}}{\sum_{j \in F} m_j}, \tag{10}$$

then the framework contribution is the time derivative of a bounded quantity and does not contribute to the conductivity. In that frame one may use the mobile-carrier current

$$\tilde{\mathbf{J}} = \frac{1}{\Omega} \sum_{i \in C} q_i \mathbf{v}_i^{\mathrm{mat}}. \tag{11}$$

Equivalently, in the barycentric frame, one may use the total charge current

$$\mathbf{J} = \frac{1}{\Omega} \sum_{i \in C \cup F} q_i \mathbf{v}_i^{\mathrm{bar}}. \tag{12}$$

These two formulations are equivalent, up to bounded gauge terms and using charge neutrality. What is not generally equivalent is to use only

$$\mathbf{J}_C^{\text{bar}} = \frac{1}{\Omega}\sum_{i\in C} q_i \mathbf{v}_i^{\text{bar}} \tag{13}$$

in the barycentric frame while discarding the framework and carrier–framework terms.

The non-diffusive nature of the framework does not by itself justify neglecting σ_F and σ_{cross} in the barycentric Einstein–Helfand expression. It only justifies doing so after the carrier displacements or velocities have been expressed relative to the solid framework.

The Authors should explicitly state which reference frame was used in the conductivity calculation and either compute the total barycentric charge current or transform the Li velocities/displacements to the solid-matrix frame before neglecting framework terms.

We are very grateful to the reviewer for the detailed and constructive comment, including the illustrative figure. We agree that our previous statement was not sufficiently rigorous. In the barycentric reference frame, the non-diffusive sublattice can recoil to compensate the net displacement of the diffusive species, and this recoil motion can itself exhibit diffusive scaling.

As a simple check of this recoil effect, we examined the mass-weighted center-of-mass (COM) displacements of the Li and PS_4 sublattices in Li_3PS_4. The trajectory was generated using machine learning interatomic potential in the NVT ensemble at 900 K. As shown in Figure 2, the x components of $M_{\text{Li}}\Delta\mathbf{R}_{\text{Li}}^{\text{bar}}$ and $M_{\text{PS}_4}\Delta\mathbf{R}_{\text{PS}_4}^{\text{bar}}$ exhibit an almost exact recoil relation. This observation is consistent with the reviewer's Eq. (6).

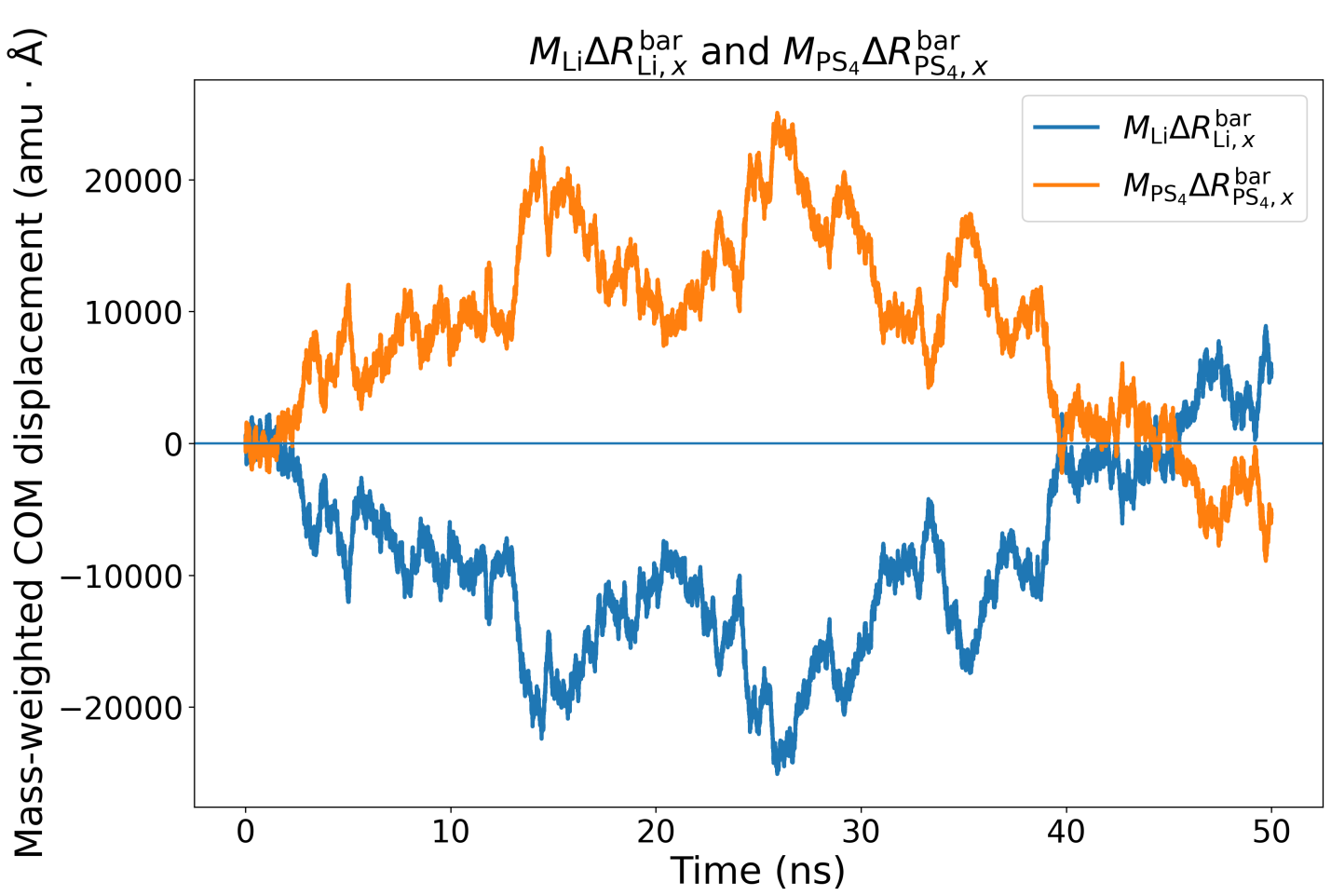

Figure 2. Time evolution of $M_{\text{Li}}\Delta R_{\text{Li},x}^{\text{bar}}$ and $M_{\text{PS}_4}\Delta R_{\text{PS}_4,x}^{\text{bar}}$ calculated from the trajectory in the barycentric reference frame, where x denotes the Cartesian x-direction. The nearly opposite motions show the collective recoil of the PS_4 framework against the net displacement of the diffusive Li species.

In our conductivity calculations, the Einstein–Helfand displacement was evaluated in the solid-matrix COM reference frame. This was indeed not explicitly stated in the previous manuscript. We have now modify the original description in the *Methods* section:

Change (*Methods*, lines 355–362)

- Original: The conductivity is calculated through the Einstein–Helfand relation:

$$\sigma_{HE} = \frac{1}{3\Omega k_B T} \lim_{t\to\infty} \frac{\langle |\sum_i q_i(\mathbf{r}_i(t) - \mathbf{r}_i(0))|^2 \rangle}{2t}$$

where $\langle\cdot\rangle$ denotes an equilibrium ensemble average, Ω and T are the system volume and temperature, k_B is the Boltzmann constant, $\mathbf{r}_i$ is the position of the ith ion and $q_i = +e$ for Na and Li ions.

- Revised: The conductivity is calculated through the Einstein–Helfand relation::

$$\sigma_{HE} = \frac{1}{3\Omega k_B T} \lim_{t\to\infty} \frac{\langle |\sum_{i\in C\cup F} q_i(\mathbf{r}_i^{bar}(t) - \mathbf{r}_i^{bar}(0))|^2 \rangle}{2t}$$

where $\langle\cdot\rangle$ denotes an equilibrium ensemble average, Ω and T are the system volume and temperature, k_B is the Boltzmann constant, C and F denote the mobile carrier sublattice and solid-matrix framework, $\mathbf{r}_i^{bar}$ denotes the position of the i-th particle in the barycentric reference frame, and q_i denotes the charge of the i-th particle. Thanks to gauge invariance, given that the system is charge-neutral, an equivalent way to evaluate the conductivity is

$$\sigma_{HE} = \frac{1}{3\Omega k_B T} \lim_{t\to\infty} \frac{\langle |\sum_{i\in C} q_i(\mathbf{r}_i^{mat}(t) - \mathbf{r}_i^{mat}(0))|^2 \rangle}{2t}$$

where $\mathbf{r}_i^{mat}$ denotes the position of particle in the solid-matrix reference frame.

2 Role of anharmonicity

A second point that remains unclear concerns the interpretation of the constrained metadynamics calculations in terms of anharmonicity. The comparison between the unconstrained case, the rotation-constrained case, and the translation-constrained case shows that allowing translational motion of the BH_4^- units substantially lowers the Li migration free-energy barrier. This is an important observation. However, it does not by itself constitute a direct measure of anharmonicity.

Indeed, a framework coordinate can strongly lower a migration barrier simply by relaxing or by dynamically gating the Li migration pathway, even if that coordinate is described by a purely harmonic potential. Similarly, the curvature of a free-energy minimum is not, by itself, a measure of anharmonicity. A shallow minimum corresponds to a soft harmonic mode if the free-energy profile remains quadratic. Anharmonicity requires evidence for deviations from a quadratic description, such as non-parabolic free-energy profiles, non-Gaussian displacement distributions, higher-order force constants, or strong temperature-dependent phonon renormalization. Broadening/softening of a finite-T velocity spectrum suggest strong coupling, but are not by themselves unique signatures of anharmonicity.

We therefore suggest that the Authors either provide a quantitative measure of anharmonicity supporting this interpretation, or rephrase the conclusion more conservatively. A quantitative proxy may be the degree of anharmonicity introduced by Knoop et al. in Ref. [KPSC20]: if $\mathbf{F}_I(t)$ is the force sampled along a finite-temperature trajectory for atom I and $\mathbf{F}_I^{(2)}(t)$ is its harmonic approximation, then the global

degree of anharmonicity at temperature T is

$$\text{deg. anharm.}\,(T) = \left[\frac{\sum_{I,\alpha}\left\langle\left(F_{I\alpha}(t) - F^{(2)}_{I\alpha}(t)\right)^2\right\rangle_T}{\sum_{I,\alpha}\langle F^2_{I\alpha}(t)\rangle_T}\right]^{1/2} \tag{14}$$

where α labels Cartesian components. This quantity directly measures the fraction of the finite-temperature force field that is not described by the harmonic approximation. It can also be resolved on selected atoms, species, or modes, which would be particularly useful here to assess whether the $BH_4{}^-$ translational degrees of freedom are genuinely anharmonic. The degree of anharmonicity was used on similar superionic conductor Li_3PS_4 in Ref. [TGGC24].

Alternatively, the Authors could quantify anharmonicity by explicitly fitting higher-order force constants, for example using packages such as hiPhive Ref. [EFE19], which allow the construction of force-constant models beyond second order. The relative importance of third-, fourth-, and higher-order force constants involving $BH_4{}^-$ translations would provide a direct perturbative measure of anharmonicity.

We thank the reviewer for this constructive comment and detailed explanation. We agree that neither this barrier lowering nor a shallow free-energy minimum is by itself a direct measure of anharmonicity. We are grateful for the references the reviewer pointed us to, which we found genuinely valuable. Following the degree-of-anharmonicity measure introduced by Knoop et al. [KPSC20], and its application to the closely related superionic conductor Li_3PS_4 in Ref. [TGGC24], we have quantified the anharmonicity of the Li sublattice in $LiBH_4$ directly. Since our study concerns Li migration, the relevant question is how anharmonic the potential-energy surface experienced by the Li ions is, which can be quantified by the degree of anharmonicity of the Li sublattice.

Specifically, the $LiBH_4$ unit cell was relaxed until the residual forces fell below 10^{-4} eV/Å, and the second-order force constants were obtained from calculations on a $4 \times 4 \times 4$ supercell using the finite displacement method implemented in Phonopy; both the relaxation and the force-constant calculation were performed with our deep potential model. We ran MD trajectories of approximately 1.6 ns at 525 K (sampling every 0.5 ps) under three constraint conditions: unconstrained, with $BH_4{}^-$ rotation constrained, and with both $BH_4{}^-$ translation and rotation constrained. From these trajectories, we evaluated the degree of anharmonicity of the Li sublattice via Eq 14.

The degree of anharmonicity of the Li sublattice is 2.17 for the unconstrained system. Constraining the $BH_4{}^-$ rotation lowers it to 1.05, and further constraining the $BH_4{}^-$ translation lowers it to 0.53. The reduction upon constraining the $BH_4{}^-$ translation supports the conclusion drawn in our manuscript that this translation enhances the anharmonicity of the Li sublattice. Note that a degree of anharmonicity greater than 1 is not unusual: a similar behavior was reported by Knoop et al. in noble metal halides, where it was attributed to the noble metal ions moving into metastable interstitial sites [KPSC20]. In $LiBH_4$, the Li ions likewise move into metastable interstitial sites, as shown in the manuscript and discussed previously in Ref. [ITM+11].

Manuscript revisions

We now incorporate the anharmonicity analysis discussed above into the *Results* section and cite the study

on Li_3PS_4 by Tisi *et al.* [TGGC24]:

Change 1 (*Results*, lines 238–243)

- Added: "To quantify this, we evaluate the degree of anharmonicity of the Li sublattice using equation (14), which has also been applied to an SE study[65], obtaining 2.17 for the unconstrained system, 1.05 under RC, and 0.53 under RTC. The pronounced drop upon constraining BH_4 translation ($1.05 \to 0.53$) quantitatively confirms that anion translation enhances the anharmonicity of the local potential experienced by Li."

The *Methods* section now describes how the degree of anharmonicity is calculated:

Change 2 (*Methods*, lines 400–412)

- Added: "We adopt the degree of anharmonicity introduced by Knoop et al[82]. For each atom I, the anharmonic contribution to the force is defined as the difference between the force F_I in finite-temperature MD and its harmonic approximation $F^{(2)}_{I,\alpha} = -\sum_{J,\beta} \Phi^{I,J}_{\alpha,\beta} \Delta R^{\beta}_{J}$, where $\Phi^{I,J}_{\alpha,\beta}$ are the second-order force constants and ΔR^{β}_{J} is the instantaneous displacement of atom J from its equilibrium position at direction β. The degree of anharmonicity at temperature T is then

$$\sigma^{\mathrm{A}}(T) = \sqrt{\frac{\sum_{I,\alpha} \left\langle \left(F_{I,\alpha}(t) - F^{(2)}_{I,\alpha}(t) \right)^2 \right\rangle_T}{\sum_{I,\alpha} \left\langle F^2_{I,\alpha}(t) \right\rangle_T}},$$

where $\langle \cdot \rangle_T$ denotes the ensemble average. Restricting the sums over I to the Li atoms yields the degree of anharmonicity of the Li sublattice reported here.
The second-order force constants $\Phi^{I,J}_{\alpha,\beta}$ were obtained from calculations on a $4 \times 4 \times 4$ supercell using the finite displacement method implemented in phonopy,[83] based on the equilibrium structure relaxed until the residual forces fell below 10^{-4} eV/Å. F_I were sampled from MD trajectories of approximately 1.6 ns at 525 K (sampled every 0.5 ps). All force-constant and force evaluations were performed with our deep potential model."

3 Dataset composition

We also have concerns regarding the dataset composition and the reported accuracy of the machine-learning interatomic potentials (MLIPs). First, the total number of structures included in the training and test sets is not clearly stated. Second, the manuscript does not describe how the structures are distributed among the different systems considered. It is therefore unclear whether all materials are equally represented in the dataset, or whether some systems are over- or under-represented. This point is particularly important for structures containing vacancies, whose representation in the training and test sets should be explicitly reported. In addition, in Supplementary Table 1, the RMSE for the forces on the test set is lower than that on the training set for several systems. The authors should explain this behaviour, since it may indicate that the training and test sets differ in composition.

We thank the reviewer for these important comments and fully appreciate the concerns regarding MLIP accuracy, which is a fundamental prerequisite underpinning the entire study. We address them in three parts below. First, we note that a separate MLIP is trained for each system rather than a single unified potential; accordingly, the question of whether the systems are equally represented does not arise, and we instead report the number of training and test configurations for each system individually. Second, since vacancy-containing structure is of particular concern, we explicitly validate the MLIP for Na_3OBH_4 in the

presence of Na vacancies. Third, we explain why the force RMSE on the test set is lower than that on the training set for several systems.

1. Number of data, composition, and distribution among systems

The number of configurations used for training separate model of each material is listed in the Table 1 below. In this study, for each material we considered only the phase exhibiting anion rotation and high ionic conductivity. Consequently, the MLIP for each material was trained on a single phase only. For $Na_2B_{10}H_{10}$, $LiBH_4$, α-Li_3PS_4 and γ-$Na_4P_2S_6$, since only a single phase is considered, the variation in data composition arises solely from the inclusion of supercells of different sizes. This approach of progressively scaling from smaller to larger supercells has been adopted in previous solid-state electrolyte study [HZW+21]. Smaller supercells impose the constraint that all periodic images are identical, whereas larger supercells relax this constraint, thereby accessing a broader and physically richer configuration space. The model trained on smaller (super)cells thus serves as a warm start for subsequent training on larger supercells. For $Na_{11}Sn_2PS_{12}$, since the unit cell is fairly large (208 atoms), the dataset consists of the unit cell only.

We briefly describe the DP-GEN scheme [ZWC+20] before discussing the composition of the training dataset for Na_3OBH_4. DP-GEN is an active learning scheme in which, after training an initial model on a small dataset, new configurations are not selected from a pre-existing pool but are instead generated on-the-fly via MD simulations driven by the current model—a step referred to as *exploration*. An error indicator, for configuration $\mathbf{R}$, is computed as

$$\epsilon = \max_i \sqrt{\langle ||\mathbf{F}_{w,i}(\mathbf{R}) - \langle F_{w,i}(\mathbf{R})\rangle||^2\rangle}, \tag{15}$$

where $\mathbf{F}_{w,i}$ is the force on atom i predicted by the w-th model, and $\langle \cdots \rangle$ denotes the ensemble average over the model ensemble. An upper (σ_{hi}) and lower (σ_{lo}) bound are then set to label the configuration as either *accurate* ($\epsilon < \sigma_{lo}$), *candidate* ($\sigma_{lo} < \epsilon < \sigma_{hi}$), or *failed* ($\epsilon > \sigma_{hi}$). DP-GEN considers the model to be converged if the proportion of configurations marked as *accurate* within the total pool of configurations reaches a threshold (here, 99.5%).

For Na_3OBH_4, one might expect the variation in data composition to arise not only from supercells of different sizes, but also from the inclusion of both host and defect structures. However, we found that when the model was trained exclusively on host structures, the subsequent DP-GEN *exploration* of configurations containing V_{Na} converged directly (with more than 99.5% labeled as *accurate*). Specifically, upon introducing one Na vacancy among 81 Na atoms in a 3×3×3 supercell. Using a model trained solely on the perfect crystal, the DP-GEN *exploration* converged without triggering any additional labeling. The *exploration* settings were as follows: temperatures of 50, 300, 550, 800, and 1100 K; 50 independently perturbed initial structures (250 in total); 12,000 MD steps per trajectory; a timestep of 0.5 fs; and a dump frequency of every 15 fs. This demonstrates that the model is capable of capturing the local symmetry breaking and distortions associated with sodium vacancy formation. Therefore, no additional labelled defect configurations were added to the training set.

We note that a separate MLIP was trained for each material system independently. The question of how structures are distributed among different systems therefore does not arise in our framework, as each model is trained and evaluated solely on data from its corresponding system.

2. Validation of the MLIP for Na_3OBH_4 in the presence of Na vacancies

To further validate the MLIP for Na_3OBH_4 in the presence of Na vacancies, we performed two additional

Table 1 Sizes and compositions of the training and testing datasets for the separate model trained for each material.

Material	Training data	Test data
$Na_2B_{10}H_{10}$	2666 ($1 \times 1 \times 1$); 9547 ($2 \times 1 \times 1$)	500 ($2 \times 1 \times 1$)
$LiBH_4$	7800 ($2 \times 2 \times 1$); 19661 ($3 \times 3 \times 2$)	500 ($3 \times 3 \times 2$)
Na_3OBH_4 (host)	6349 ($2 \times 2 \times 2$); 9804 ($3 \times 3 \times 3$)	500 ($3 \times 3 \times 3$)
Na_3OBH_4 (defect)	—	500 ($3 \times 3 \times 3$)
Li_3PS_4	4859 ($1 \times 1 \times 1$); 20024 ($2 \times 2 \times 2$)	500 ($2 \times 2 \times 2$)
$Na_4P_2S_6$	5105 ($1 \times 1 \times 1$); 3714 ($2 \times 2 \times 2$)	500 ($2 \times 2 \times 2$)
$Na_{11}Sn_2PS_{12}$	4917 ($1 \times 1 \times 1$)	500 ($1 \times 1 \times 1$)

checks beyond the DP-GEN convergence criterion described above. First, we compared the migration energy profiles obtained from MLIP-NEB and DFT-NEB calculations. Specifically, we first performed an IT-NEB calculation using the MLIP to obtain an initial path, followed by a CI-NEB calculation yielding 8 images including the endpoints. These images were then used as the starting configurations for a DFT CI-NEB calculation in VASP, using the same settings as those employed in the training set generation. As shown in Figure 3, the MLIP and DFT energy profiles are in close agreement, with pointwise deviations on the order of 0.02 eV, demonstrating that the model accurately captures the energetics of the Na migration event.

Second, we computed the model deviation, i.e., the error indicator ϵ, for each image along the NEB path. As shown in Figure 3 (the lower panel), all images fall within the accurate regime, confirming that these configurations are well within the domain of applicability of the current model.

Taken together, these results demonstrate that our MLIP is reliable for studying ion transport in Na_3OBH_4 containing Na vacancies.

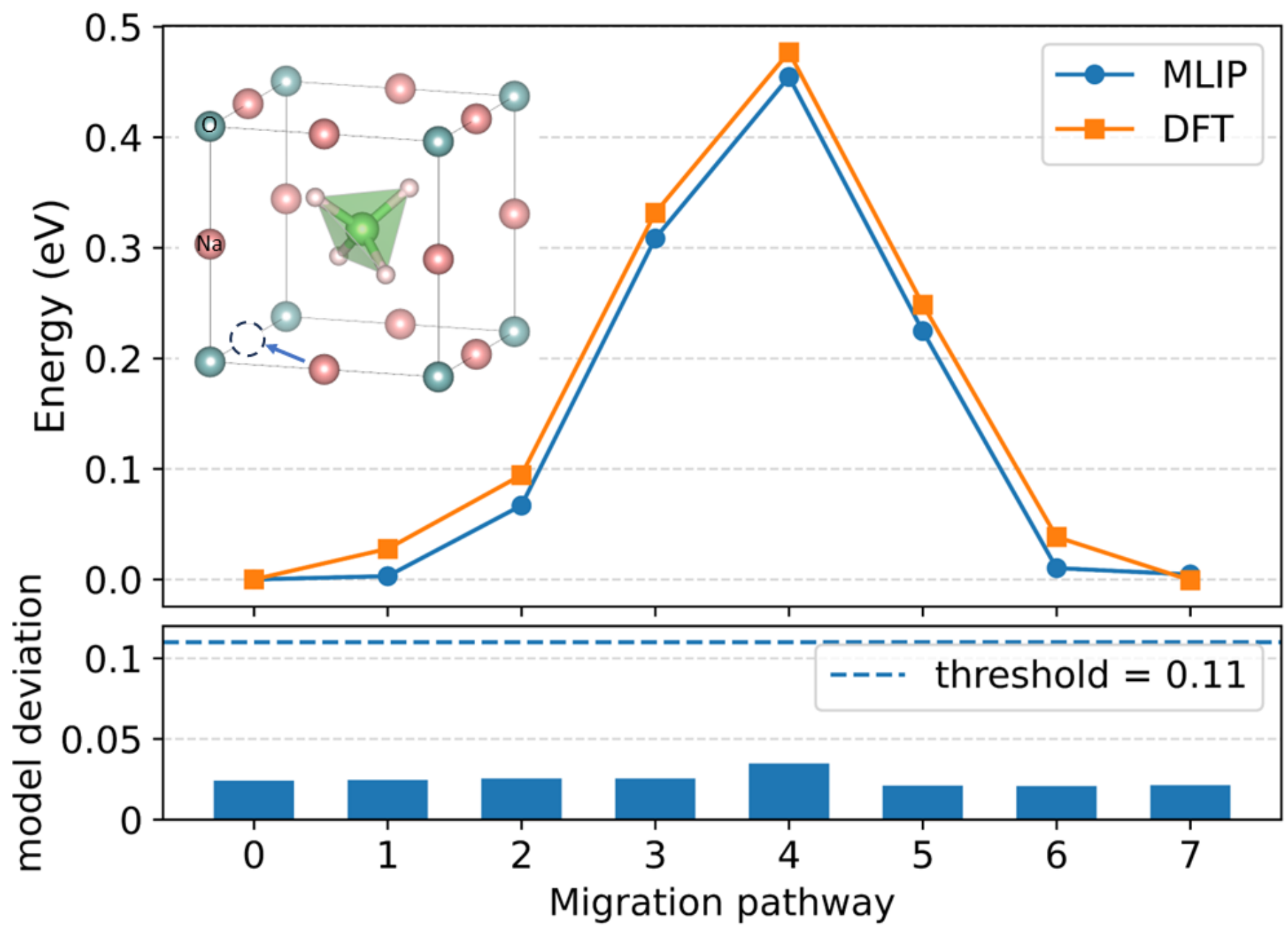

Figure 3. MLIP and DFT comparison for Na migration in $3 \times 3 \times 3$ Na_3OBH_4 supercell containing a Na vacancy. (Top) CI-NEB energy profiles computed with MLIP (blue) and DFT (orange) along the migration pathway, with 8 images including the endpoints. The migration barriers are 0.46 eV (MLIP) and 0.48 eV (DFT), with pointwise deviations on the order of 0.02 eV. The inset shows the migration pathway, where the dashed circle indicates the Na vacancy site and the arrow denotes the direction of Na migration. (Bottom) Model deviation for each image along the NEB path. All values fall well below the DP-GEN convergence threshold of 0.11 eV/Å, confirming that these configurations lie within the domain of applicability of the current MLIP.

3. Lower force RMSE of test set than training set

As the reviewer pointed out, the force RMSE being lower on the test set than on the training set stems from a difference in composition between the two sets. On one hand, our previous test set spanned only a narrow energy range, over which the forces are predicted accurately, which lowered its RMSE; with a newly constructed test set, the test-set RMSE increases, though it remains slightly lower than that on the training set. On the other hand, the training-set configurations were all generated through active learning and are thus intrinsically hard to fit, which raises the training-set RMSE. We elaborate on both points below.

3.1 Test set

Our analysis confirms that the original test set is compositionally non-uniform, and that this non-uniformity is responsible for its unexpectedly low force RMSE. Taking Na_3OBH_4 as a representative example, the test set consists of two subsets: 3021 configurations from a 3×3×3 supercell and 3768 configurations from a 2×2×2 supercell. The two subsets differ markedly in force accuracy: the 3×3×3 subset yields a force RMSE of only 5.94 meV/Å, whereas the 2×2×2 subset gives 21.90 meV/Å. This is also reflected in the force parity plots (Figure 4a,b), where the 2×2×2 predictions show a slightly wider scatter about the diagonal and a marginally broader range of reference forces. The low overall force RMSE therefore arises mainly

because this more accurately predicted 3×3×3 subset carries a large weight in the average and pulls it down.

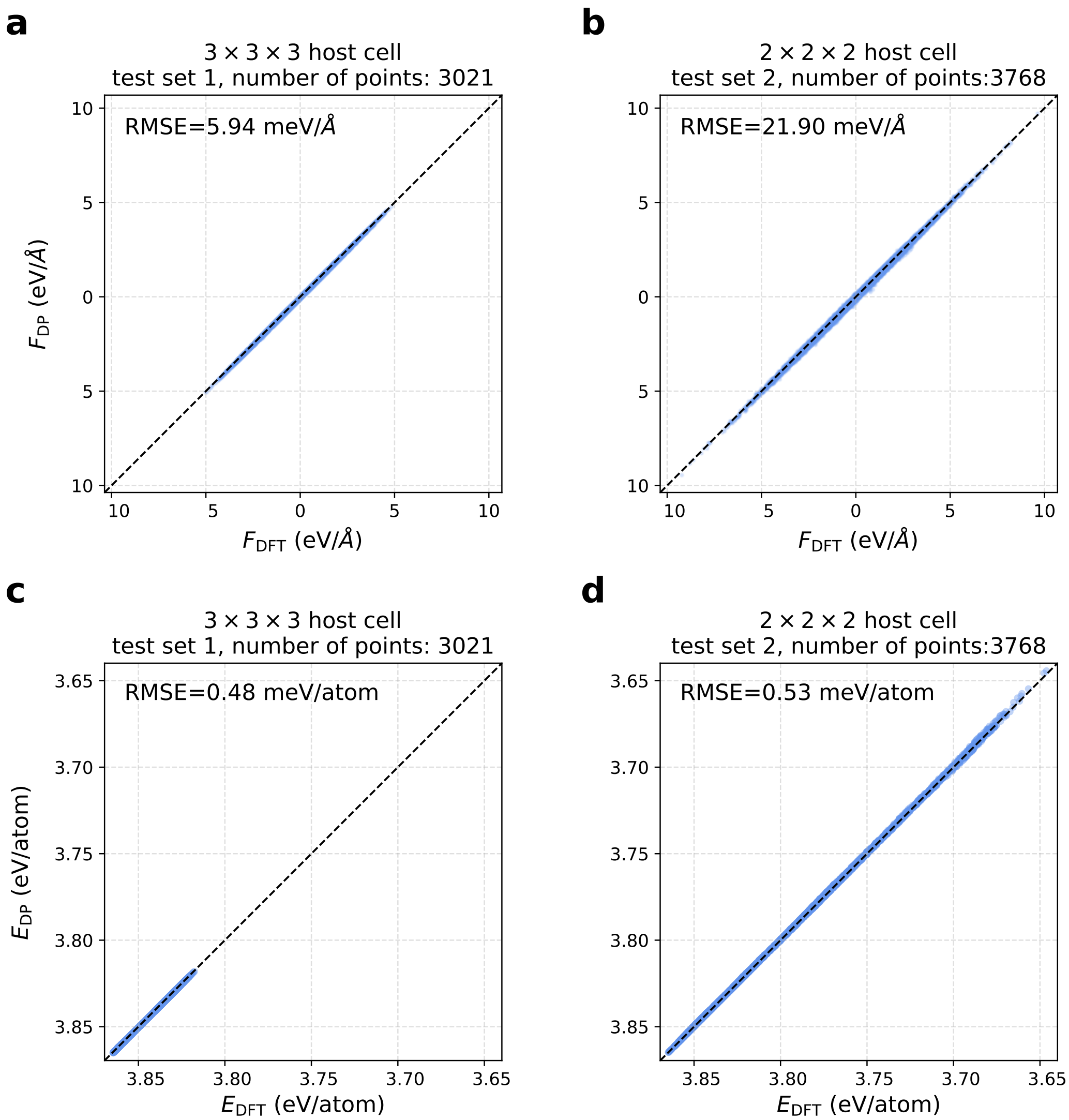

Figure 4. Parity plots of (a,b) force and (c,d) energy for the test set used in the original manuscript. The original test set consists of two subsets: test set 1 from a 3×3×3 supercell and test set 2 from a 2×2×2 supercell.

This can be verified quantitatively. Since the force RMSE is a root-mean-square over all force components, the two subsets should be combined with weights N_i equal to their number of force components, $N_i = N^i_{conf} \times N^i_{atom} \times 3$, where $i = 1$ and 2 denote the 3×3×3 and 2×2×2 subsets, respectively. For Na_3OBH_4, the 3×3×3 subset contains 3021 configurations of 243 atoms each (N_1 = 2,202,309) and the 2×2×2 subset contains 3768 configurations of 72 atoms each (N_2 = 813,888), so the two subsets carry

weights of about 73% and 27%. The combined force RMSE is then

$$\mathrm{RMSE}_{\mathrm{overall}} = \sqrt{\frac{N_1\,\mathrm{RMSE}_1^2 + N_2\,\mathrm{RMSE}_2^2}{N_1 + N_2}} = \sqrt{\frac{2202309 \times 5.94^2 + 813888 \times 21.90^2}{2202309 + 813888}} = 12.46\,\mathrm{meV/Å} \quad (16)$$

where 5.94 and 21.90 meV/Å are the subset force RMSEs of one deep potential (DP) model. As explained in the first point of this response, DP-GEN trains four models simultaneously to form a model ensemble. The other three models give $(\mathrm{RMSE}_1, \mathrm{RMSE}_2)$ of (5.80, 21.32), (5.58, 20.44), and (5.73, 20.94) meV/Å, corresponding to overall force RMSEs of 12.13, 11.64, and 11.93 meV/Å. Averaging over the four models gives 12.0 meV/Å, the value reported in our original manuscript.

The non-uniformity of the test set is also evident from its energy distribution. As shown in Figure 4c,d, the 3×3×3 subset is confined to a low-energy region and spans a much narrower energy range (−3.87 to −3.82 eV/atom) than the 2×2×2 subset (−3.87 to −3.65 eV/atom). The test set is thus energetically imbalanced.

Because of the issues discussed above, we no longer recommend using the original test sets, and we therefore constructed new test sets for all six systems. The new test sets differ from the original ones in two respects. First, the original test sets could contain supercells of different sizes. As Morrow et al. point out [MGD23], for per-cell properties such as the total energy, different normalization conventions yield different error values, and they recommend that, when comparing such properties, "datasets containing structures of the same size are used." Accordingly, for each material, the new test set uses a single supercell size, namely the largest supercell present in the corresponding training set.

Second, to cover a broad and physically representative region of configuration space, the configurations are sampled from molecular dynamics trajectories over a wide range of temperatures, combining ab initio molecular dynamics (AIMD) with longer MD runs driven by the DP model. The temperature ranges are chosen to encompass those used in the production simulations. The MD trajectories start from perturbed structures generated using DP-GEN. The cell is perturbed by drawing each element of a 3×3 matrix $\boldsymbol{P}$ uniformly from $[-0.02, 0.02]$ and transforming the cell matrix $\boldsymbol{L}$ (whose rows are the three lattice vectors) into $(\boldsymbol{I} + \boldsymbol{P})\boldsymbol{L}$; each Cartesian component of every atomic position is also independently displaced by a random value in $[-0.2, 0.2]$ Å.

We illustrate the construction using Na_3OBH_4 as an example; its test set is built on the 3×3×3 supercell and consists of two parts, an AIMD set and an MLIP-MD set. For the AIMD set, 200 perturbed structures were generated and each was run for 0.2 ps of AIMD, with temperatures ranging from 150 K to 1000 K. The final frame of each trajectory was retained, giving 200 DFT-labeled configurations. For the MLIP-MD set, 30 perturbed initial structures were each propagated for 100 ps using the current DP model, with temperatures ranging from 150 K to 1000 K. Structures were sampled every 1 ps from the trajectory, yielding 3000 configurations. Following the descriptor-based train set selection framework of Imbalzano et al. [IAG+18], for each structure a global fingerprint was constructed by summing the local fingerprints of all atomic environments—here obtained from our neural network descriptor. The global fingerprints were reduced by principal component analysis, and 300 representative structures were selected by farthest-point sampling. The final Na_3OBH_4 test set thus comprises 200 configurations obtained directly from AIMD and 300 configurations selected from the MLIP-MD trajectories, the latter subsequently labelled by DFT single-point calculations.

To compare the original test set with the new one generated by the procedure described above, we examined their nearest-neighbor distributions. As shown in Figure 5, the 3×3×3 subset of the original

test set (black) shows nearest-neighbour distances that are almost entirely confined below about 1.0, with a pronounced peak close to zero, that is, a large number of global fingerprints with near-zero nearest-neighbour distances. By contrast, the newly generated AIMD (blue) and MLIP-MD (orange) test sets exhibit markedly larger nearest-neighbor distances (mostly between 1.0 and 2.5) with broader distributions.

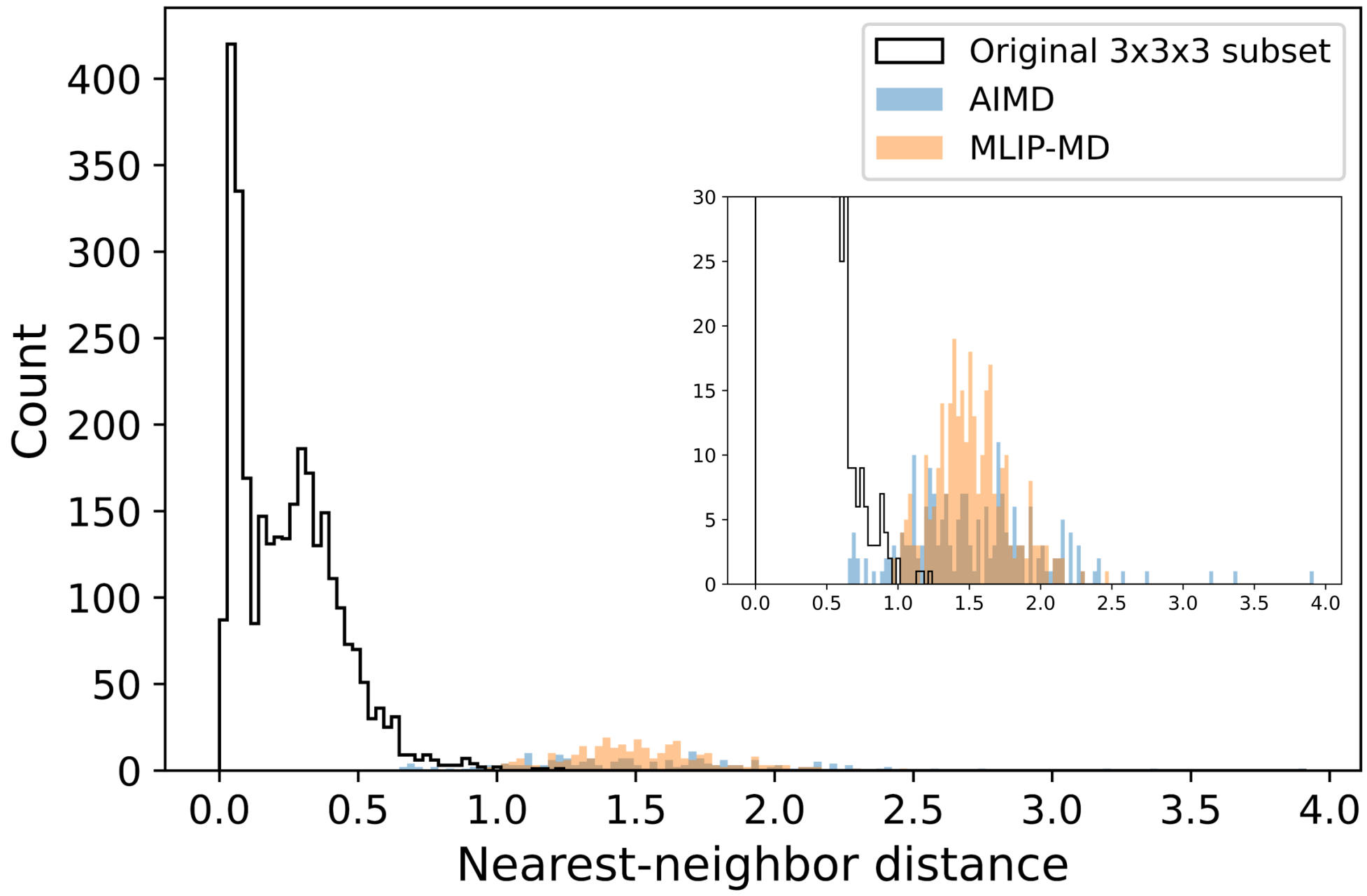

Figure 5. Distribution of nearest-neighbour distances for three test sets: the original 3×3×3 subset (black outline), the AIMD set (blue), and the MLIP-MD set (orange). The inset magnifies the low-count region.

Table 2 lists the energy and force RMSEs on the training and new test sets for all systems, evaluated with the models used in the production MD simulations. Table 3 lists the corresponding RMSEs on the training and original test sets for comparison. Two changes are worth noting:

(i) we have added a test set for Na_3OBH_4 containing a Na vacancy; and

(ii) the force RMSEs on the new MLIP-MD test sets are all higher than those reported previously.

Table 2 RMSE of energies per atom (meV/atom) and forces (meV/Å) on training and new test sets for the DP models of six superionic conductors. The test sets consist of configurations sampled from AIMD and MLIP-MD trajectories, respectively.

Materials	RMSE in energy			RMSE in force		
	Training	Test		Training	Test	
		AIMD	MLIP-MD		AIMD	MLIP-MD
$Na_{11}Sn_2PS_{12}$	0.68	1.88	2.22	48.3	45.5	48.6
$Na_4P_2S_6$	1.13	2.74	1.85	64.1	55.1	54.4
$Na_2B_{10}H_{10}$	1.23	0.92	0.99	81.5	56.7	70.2
Na_3OBH_4(perfect)	0.47	0.19	0.27	29.4	19.2	20.7
Na_3OBH_4(defect)	—	0.62	0.46	—	29.9	28.0
$LiBH_4$	1.28	0.75	0.67	57.4	32.6	38.1
Li_3PS_4	1.35	1.34	1.20	88.5	55.5	75.2

Table 3 RMSE of energies per atom (meV/atom) and forces (meV/Å) on training and original test sets for the DP models of six superionic conductors.

Materials	RMSE in energy		RMSE in force	
	Training	Test	Training	Test
$Na_{11}Sn_2PS_{12}$	0.68	0.70	48.3	41.8
$Na_4P_2S_6$	1.13	1.14	64.1	53.2
$Na_2B_{10}H_{10}$	1.23	1.11	81.5	55.7
Na_3OBH_4	0.47	0.51	29.4	12.5
$LiBH_4$	1.28	1.74	57.4	35.6
Li_3PS_4	1.35	1.11	88.5	71.3

3.2 Training set

Even though the newly constructed test sets are structurally more diverse than the original ones, the force RMSEs evaluated on them are still generally lower than those on the training set (Table 2). We first inspect the hyperparameters of the neural networks across the different systems (Table 4). One thing they share is that p_F^{limit}, the force prefactor at the infinite training step, is always set smaller than p_E^{limit}, the energy prefactor at infinite training step. We suspect that this is related to the force RMSE on the test set being lower than that on the training set. To see how this prefactor comes into play, recall that the loss function L in DP is written as [ZZL+23]

$$L = p_E L_E + p_F L_F + p_V L_V, \tag{17}$$

where p_η varies during training. For training step t, it is expressed as

$$p_\eta(t) = p_\eta^{limit}\left(1 - \frac{\gamma(t)}{\gamma(0)}\right) + p_\eta^{start}\frac{\gamma(t)}{\gamma(0)}, \tag{18}$$

where $\gamma(t)$ is the learning rate, which itself decays exponentially during training and can be written as

$$\gamma(t) = \gamma(0) r^{t/s}, \tag{19}$$

where the decay rate r is related to the decay steps s and total training steps t^{stop}.

$$r = \left(\frac{\gamma^{stop}}{\gamma(0)}\right)^{s/t^{stop}} \tag{20}$$

Table 4 Training parameters used for the DP models.

Material	p_E^{start}	p_E^{limit}	p_F^{start}	p_F^{limit}	p_V^{start}	p_V^{limit}
$LiBH_4$	1	10	1000	5	0	0
$Na_2B_{10}H_{10}$	1	10	1000	5	0	0
$Na_4P_2S_6$	1	20	1000	1	0	0
Li_3PS_4	1	20	1000	1	0	0
Na_3OBH_4	1	20	1000	5	0	0
$Na_{11}Sn_2PS_{12}$	1	20	1000	5	0	0
Descriptor type		`se_e2_a`				
Smoothing-start radius r_{smth_start}		3.5 Å				
Cutoff radius r_{cut}		7.0 Å				
Descriptor-network neurons		(25, 50, 100)				
Axis-neuron size		16				
Fitting-network neurons		(240, 240, 240)				
Initial learning rate $\gamma(0)$		1×10^{-3}				
Final learning rate γ^{stop}		1×10^{-8}				
Decay steps s		20,000				
Number of training steps t^{stop}		8×10^6				

Again taking Na_3OBH_4 as an example, we keep all other settings unchanged (including the random seed) and, in addition to the original prefactor setting, try two alternative ones. The specific settings and the corresponding RMSEs on the training and test sets are listed in Table 5. When we increase p_F^{limit} from 5 to 100 (from model0 to model1), the energy RMSE on the training set and on both test sets rises slightly, as expected. The force RMSE, however, does not decrease correspondingly—raising the force prefactor does not actually improve the force accuracy. Moreover, the force RMSE on the training set

remains larger than that on the test sets. In addition, we train another model (model2) using the prefactor settings commonly adopted in the literature [WWG+23, XLR+23, LLH22]. Model2 achieves lower absolute force and energy RMSEs on all datasets. Meanwhile, model2 still exhibits a higher force RMSE on the training set than on the test sets.

Table 5 Comparison of loss-function parameters and RMSEs for three DP models of Na_3OBH_4. Energy and force RMSEs are reported in meV/atom and meV/Å, respectively.

	**model0**[a]	**model1**	**model2**
p_E^{start}	1	1	0.02
p_E^{limit}	20	20	1
p_F^{start}	1000	1000	1000
p_F^{limit}	5	100	1
Force RMSE (full training set)	29.4	30.0	25.3
Energy RMSE (full training set)	0.47	0.51	0.39
Energy RMSE (test set AIMD)	0.19	0.21	0.17
Force RMSE (test set AIMD)	19.2	19.7	17.9
Energy RMSE (test set MLIP-MD)	0.27	0.30	0.27
Force RMSE (test set MLIP-MD)	20.7	21.1	16.5

[a] Model0 is the model used in the production MD simulations reported in the original manuscript.

Having ruled out the prefactor as the main cause, we now look inside the training set itself to see which configurations drive the largest force RMSE. The training set was built with DP-GEN, which iteratively explores configuration space (e.g., via MD), uses the model deviation (Eq. 15) to select configurations worth labeling, and labels a selected subset at each iteration. This iterative structure gives us a natural way to split the training set into per-iteration subsets and evaluate the force RMSE of each. Figure 6 shows these per-iteration force RMSEs for Na_3OBH_4, evaluated with the final model (model0). The initial data set from AIMD has a much lower force RMSE, whereas most subsets added during the DP-GEN active-learning iterations exhibit RMSEs comparable to or higher than the overall training-set value (29.4 meV/Å). As a result, the overall force RMSE on the training set is higher than that on the test set (20.7 meV/Å).

This behavior is consistent with the statistical bias introduced by active learning, as discussed by Farquhar et al. [FGR21]. We briefly summarize their reasoning here. In supervised learning, one seeks a decision rule f_θ that minimizes the population risk

$$r = \mathbb{E}_{\mathbf{x},y\sim p_{\text{data}}}\left[L\left(y, f_\theta(\mathbf{x})\right)\right], \tag{21}$$

where L is the loss function and $(\mathbf{x}, y)$ are inputs and outputs drawn from the population data distribution p_{data}. In practice, r is not directly accessible, and one instead works with the empirical distribution

of a dataset of N points drawn from the population. When these points are sampled identically and independently distributed (i.i.d.) from p_{data}, the resulting empirical risk

$$\hat{R} = \frac{1}{N}\sum_{n=1}^{N} L(y_n, f_\theta(\mathbf{x}_n)) \tag{22}$$

is an unbiased estimator of r. In active learning, however, the data are not drawn i.i.d.: points are selected from a pool of unlabeled data according to an acquisition strategy (e.g., the model deviation used in DP-GEN). If M such points are actively acquired and labeled, the sub-sample empirical risk is

$$\widetilde{R} = \frac{1}{M}\sum_{m=1}^{M} L(y_m, f_\theta(\mathbf{x}_m)) \,. \tag{23}$$

It is then a biased estimator of the population risk r. In particular, Farquhar et al. showed that this estimator can overestimate the risk because active learning samples the hardest points. We believe that DP-GEN introduces the same effect by preferentially selecting hard-to-fit configurations during active learning, which explains why the force RMSE on the training set exceeds that on the test sets. Meanwhile, active-learning bias may be actively helpful when training overparameterized neural networks, counteracting the tendency toward higher training RMSEs and leading to test RMSEs comparable to or slightly higher than the training RMSEs [FGR21].

Similar behavior has also been reported in other studies using DP models generated with the DP-GEN scheme. For example, in a recent study of high-pressure H_2O ice, Hou et al. [HTL+26] constructed a DP model and reported a larger force RMSE on the training set than on the test set (346.2 vs. 222.9 meV/Å), which they attributed to non-physical or extreme structures retained from the initial active sampling process. A similar trend was observed for the rutile TiO_2(110)–water interface by Zhuang et al. [ZBC22]: their five-trilayer training set gave a force RMSE of 102.3 meV/Å, whereas DFT validation on interface models with 3–12 trilayers yielded force RMSEs below 100 meV/Å. These examples indicate that a lower force RMSE on the test set than on the training set is not anomalous in DP-GEN-based MLIP construction.

Overall, all our models satisfy the accuracy criteria commonly expected for a reliable DP model on the test set—namely, energy and force RMSEs below 10 meV/atom and 100 meV/Å, respectively [WZW+22]. We therefore consider these models reliable for the MD simulations carried out in this work.

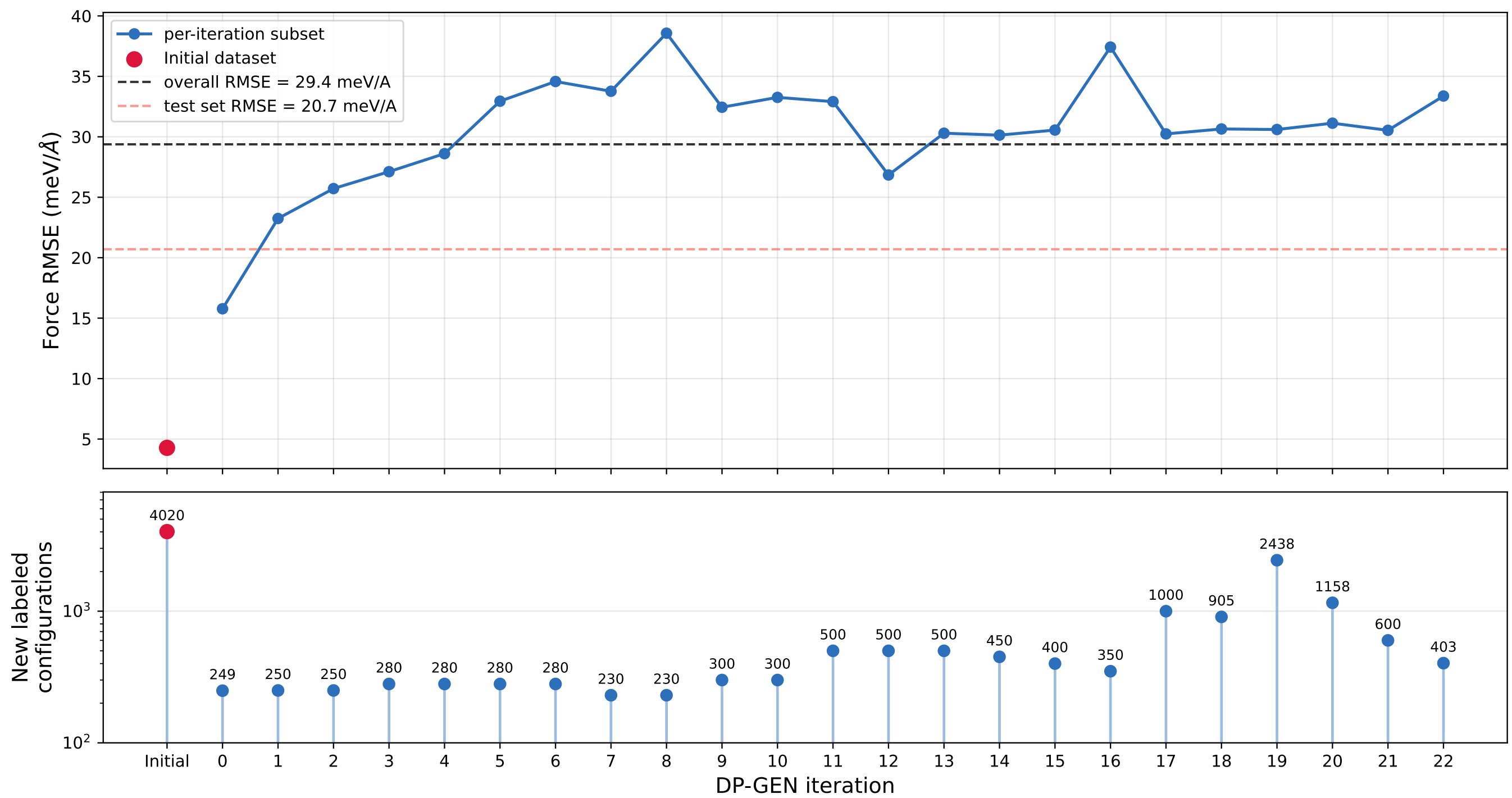

Figure 6. (top) Force RMSE of Na_3OBH_4 on the initial dataset and on each DP-GEN iteration's newly labeled subset evaluated with the final DP model, and (bottom) the number of configurations labeled in each iteration.

Manuscript revisions

Change 1 (*SI*, lines 40–44)

- Added: "We also observe that, for some systems, the force RMSE on the test set exceeds that on the training set. We attribute this to the active-learning scheme employed, which preferentially selects hard-to-fit configurations from the candidate pool[4]. Meanwhile, active-learning bias may be actively helpful when training overparameterized neural networks, counteracting the tendency toward higher training RMSEs and leading to test RMSEs comparable to or slightly higher than the training RMSEs[4]."
- Reason: Added an explanation of why the RMSE on the test set can be either higher or lower than that on the training set across different systems.

Change 2 (*Methods*, lines 336–338; *SI*, lines 15–17)

- Original: "For Na_3OBH_4 we first trained the models using the perfect crystal. We then introduced one Na vacancy into a $3 \times 3 \times 3$ supercell for further training, and finally obtained the converged model used in our simulations."
- Revised: "For Na_3OBH_4, we first carry out the DP-GEN iterations on the host supercell. After convergence, the MD exploration is performed on supercell containing one Na vacancy, which converges without triggering any additional labeling. Consequently, the training set consists solely of host supercells."

- Reason: Revised the previously misleading description of the Na_3OBH_4 training-set composition into a more detailed and accurate one.

Change 3 (*SI*, lines 18–30)

- Added: "To build the test set, configurations are sampled from MD trajectories spanning the temperature range of the production simulations, combining AIMD with longer DP-driven MD, so as to cover a broad and physically representative region of configuration space. All trajectories start from perturbed structures generated using DP-GEN, in which the cell matrix $\boldsymbol{L}$ is transformed into $(\boldsymbol{I} + \boldsymbol{P})\boldsymbol{L}$ with each element of $\boldsymbol{P}$ drawn uniformly from $[-0.02, 0.02]$, and every atomic coordinate is independently displaced by a random value in $[-0.2, 0.2]$ Å.

  Taking Na_3OBH_4 as an example, its test set is built on the $3 \times 3 \times 3$ supercell and combines an AIMD set with an MLIP-MD set. For the former, 200 perturbed structures were each run for 0.2 ps of AIMD (150–1000 K), and the final frame of each was retained, giving 200 DFT-labeled configurations. For the latter, 30 perturbed structures were each propagated for 100 ps with the current DP model over the same temperature range and sampled every 1 ps, yielding 3000 configurations; following Imbalzano et al.[2], a global fingerprint was obtained for each by summing the local fingerprints of all atomic environments, reduced by principal component analysis, and 300 representative structures were selected by farthest-point sampling and labeled by DFT single-point calculations. The final test set thus comprises 200 AIMD and 300 MLIP-MD configurations."
- Reason: We now describe how the new test set is constructed in detail.

Change 4 (*SI*, Supplementary Table 2)

- Original: Formerly Supplementary Table 1, which was based on the original test set and reported the standard deviation computed across the model ensemble.
- Revised: (i) Now Supplementary Table 2, updated with data from the newly generated test sets.

  (ii) The table now additionally includes results for the Na_3OBH_4 (defect) test set.

  (iii) The corresponding discussion has been updated (lines 39–40).

  (iv) We no longer report the standard deviation across the model ensemble, giving instead only the RMSE of the single model used for the production MD runs. Since the MD is driven by this single model, its accuracy is what matters most, and the ensemble standard deviation says little about the deployed model's actual prediction error.

Change 5 (*SI*, Supplementary Table 1; *Methods*, lines 322–324)

- Original: "For each material, a dataset containing 6,000 DFT energies and forces was used to train the initial model."
- Revised: "The sizes and compositions of the training and test datasets for each material are listed in Supplementary Table 1."
- Reason: The original description was ambiguous because each configuration has one DFT energy but multiple atomic force vectors. The revised text removes this ambiguity by referring to Supplementary Table 1, where the dataset size and composition are explicitly listed for each material.

Change 6 (*SI*, Supplementary Fig.3–9)

- Original: The parity plots in the former Supplementary Fig. 3–4 were based on the old test set.
- Revised: All parity plots (now Supplementary Fig. 3–9) have been updated using the new test set, and the distribution of the training-set data is no longer shown. In addition, a new parity plot for Na_3OBH_4 with a single Na vacancy has been added.

Change 7 (*SI*, Supplementary Fig.10; lines 77–89)

- Added: "To further assess the accuracy of the MLIP, we compared its migration barriers for the vacancy-mediated Na-ion pathway in Na_3OBH_4 with those from DFT using the nudged elastic band (NEB) method. First, we compared the migration energy barriers obtained from MLIP-NEB and DFT-NEB calculations. Specifically, we first performed an IT-NEB calculation using the MLIP to obtain an initial path, followed by a CI-NEB calculation yielding 8 images including the endpoints. These images were then used as the starting configurations for a DFT CI-NEB calculation in VASP, using the same settings as those employed in the training set generation. As shown in Supplementary Fig. 10, the MLIP and DFT energy profiles are in close agreement, with pointwise deviations on the order of 0.02 eV, demonstrating that the model accurately captures the energetics of the Na migration event.

  Second, we computed the model deviation[12], i.e., the error indicator ϵ, for each image along the NEB path. As shown in Supplementary Fig. 10 (the lower panel), all images fall within the accurate regime, confirming that these configurations are well within the domain of applicability of the current model."

  The figure showing the NEB and model-deviation results has been added to the SI as Supplementary Fig. 10.
- Reason: To further validate the accuracy of the MLIP for Na_3OBH_4 with defect.

4 Data availability

Before publication, we strongly recommend that the authors make the complete dataset publicly available through an established open repository, such as Zenodo or Materials Cloud [EO13, TKP+20]. This should include the training, validation, and test structures, together with the corresponding DFT energies, forces, and relevant metadata. Providing these data would improve the reproducibility of the work and allow the community to independently assess the quality and transferability of the reported MLIPs.

We thank the reviewer for this suggestion. We have deposited the complete training and test datasets, including the atomic coordinates, simulation cells, and the corresponding DFT energies and forces, in a public Zenodo repository (DOI: `10.5281/zenodo.21320506`). The repository description provides the metadata needed to reproduce the reference data, such as the DFT exchange-correlation functional and the plane-wave cutoffs used for each system. Our protocol does not employ a separate validation set.

Change (*Data availability*, lines 452–453)

- Added: "The training and test datasets used in this work are available in a public Zenodo repository `10.5281/zenodo.21320507`."

Reviewer #3 (Remarks to the Author):
I have read the extensive and carefully drafted reply by these authors. In my opinion this manuscript is ready for publication.

We thank the reviewer for their careful reading of our reply and for recommending the manuscript for publication. We greatly appreciate their time and support throughout the review process.

References

[EFE19] Fredrik Eriksson, Erik Fransson, and Paul Erhart. The Hiphive Package for the Extraction of High-Order Force Constants by Machine Learning. *Advanced Theory and Simulations*, 2(5):1800184, May 2019.

[EO13] European Organization For Nuclear Research and OpenAIRE. Zenodo, 2013.

[FGR21] Sebastian Farquhar, Yarin Gal, and Tom Rainforth. On statistical bias in active learning: How and when to fix it. In *International Conference on Learning Representations (ICLR)*, 2021.

[GTGC24] Lorenzo Gigli, Davide Tisi, Federico Grasselli, and Michele Ceriotti. Mechanism of Charge Transport in Lithium Thiophosphate. *Chemistry of Materials*, 36(3):1482–1496, February 2024.

[HTL+26] Pengfei Hou, Yumiao Tian, Zifeng Liu, Junwen Duan, Hanyu Liu, Xing Meng, Russell J. Hemley, and Yanming Ma. Interface-dependent phase transitions and ultrafast hydrogen superionic diffusion of H 2 O ice. *Physical Review B*, 113(18):184103, May 2026.

[HZW+21] Jianxing Huang, Linfeng Zhang, Han Wang, Jinbao Zhao, Jun Cheng, and Weinan E. Deep potential generation scheme and simulation protocol for the $Li_{10}GeP_2S_{12}$-type superionic conductors. *J. Chem. Phys.*, 154(9):094703, March 2021.

[IAG+18] Giulio Imbalzano, Andrea Anelli, Daniele Giofré, Sinja Klees, Jörg Behler, and Michele Ceriotti. Automatic selection of atomic fingerprints and reference configurations for machine-learning potentials. *The Journal of Chemical Physics*, 148(24):241730, June 2018.

[ITM+11] Tamio Ikeshoji, Eiji Tsuchida, Tetsuya Morishita, Kazutaka Ikeda, Motoaki Matsuo, Yoshiyuki Kawazoe, and Shin-ichi Orimo. Fast-ionic conductivity of Li^+ in $LiBH_4$. *Phys. Rev. B*, 83(14):144301, April 2011.

[KPSC20] Florian Knoop, Thomas A. R. Purcell, Matthias Scheffler, and Christian Carbogno. Anharmonicity measure for materials. *Physical Review Materials*, 4(8):083809, August 2020.

[LLH22] Jinfeng Liu, Jinggang Lan, and Xiao He. Toward High-level Machine Learning Potential for Water Based on Quantum Fragmentation and Neural Networks. *The Journal of Physical Chemistry A*, 126(24):3926–3936, June 2022.

[MGD23] Joe D. Morrow, John L. A. Gardner, and Volker L. Deringer. How to validate machine-learned interatomic potentials. *J. Chem. Phys.*, 158(12):121501, March 2023.

[TGGC24] Davide Tisi, Federico Grasselli, Lorenzo Gigli, and Michele Ceriotti. Thermal conductivity of Li 3 PS 4 solid electrolytes with *ab initio* accuracy. *Physical Review Materials*, 8(6):065403, June 2024.

[TKP+20] Leopold Talirz, Snehal Kumbhar, Elsa Passaro, Aliaksandr V. Yakutovich, Valeria Granata, Fernando Gargiulo, Marco Borelli, Martin Uhrin, Sebastiaan P. Huber, Spyros Zoupanos, Carl S. Adorf, Casper Welzel Andersen, Ole Schütt, Carlo A. Pignedoli, Daniele Passerone, Joost VandeVondele, Thomas C. Schulthess, Berend Smit, Giovanni Pizzi, and Nicola Marzari. Materials Cloud, a platform for open computational science. *Scientific Data*, 7(1):299, September 2020.

[WWG+23] Xiaoyang Wang, Zhenyu Wang, Pengyue Gao, Chengqian Zhang, Jian Lv, Han Wang, Haifeng Liu, Yanchao Wang, and Yanming Ma. Data-driven prediction of complex crystal structures of dense lithium. *Nature Communications*, 14(1):2924, May 2023.

[WZW+22] Tongqi Wen, Linfeng Zhang, Han Wang, Weinan E, and David J Srolovitz. Deep potentials

for materials science. *Materials Futures*, 1(2):022601, June 2022.

[XLR+23] R. L. Xiao, K. L. Liu, Y. Ruan, L. Hu, and B. Wei. Tutorial: Deep learning prediction of thermophysical properties for liquid multicomponent alloys. *Journal of Applied Physics*, 134(19):191101, November 2023.

[ZBC22] Yong-Bin Zhuang, Rui-Hao Bi, and Jun Cheng. Resolving the odd–even oscillation of water dissociation at rutile TiO2(110)–water interface by machine learning accelerated molecular dynamics. *The Journal of Chemical Physics*, 157(16):164701, October 2022.

[ZWC+20] Yuzhi Zhang, Haidi Wang, Weijie Chen, Jinzhe Zeng, Linfeng Zhang, Han Wang, and Weinan E. DP-GEN: A concurrent learning platform for the generation of reliable deep learning based potential energy models. *Comput. Phys. Commun.*, 253:107206, August 2020.

[ZZL+23] Jinzhe Zeng, Duo Zhang, Denghui Lu, Pinghui Mo, Zeyu Li, Yixiao Chen, Marián Rynik, Li'ang Huang, Ziyao Li, Shaochen Shi, Yingze Wang, Haotian Ye, Ping Tuo, Jiabin Yang, Ye Ding, Yifan Li, Davide Tisi, Qiyu Zeng, Han Bao, Yu Xia, Jiameng Huang, Koki Muraoka, Yibo Wang, Junhan Chang, Fengbo Yuan, Sigbjørn Løland Bore, Chun Cai, Yinnian Lin, Bo Wang, Jiayan Xu, Jia-Xin Zhu, Chenxing Luo, Yuzhi Zhang, Rhys E. A. Goodall, Wenshuo Liang, Anurag Kumar Singh, Sikai Yao, Jingchao Zhang, Renata Wentzcovitch, Jiequn Han, Jie Liu, Weile Jia, Darrin M. York, Weinan E, Roberto Car, Linfeng Zhang, and Han Wang. DeePMD-kit v2: A software package for deep potential models. *J. Chem. Phys.*, 159(5):054801, August 2023.

Reviewer #1 (Remarks to the Author):
The revised version and the Authors' replies are fully satisfactory and in our opinion the manuscript meets the publication standards of Nature Communications.

As a final remark, the Authors should explicitly state, as in the previous version, that the charges (q_i) employed in the formulae for the calculation of the conductivity are integer oxidation numbers. This assumption is not merely a convenient modelling choice, but is also supported by the modern ab initio theory of charge transport in electrolytes, according to which the dc electrical conductivity can be expressed in terms of integer (topological) charges, due to quantisation of charge transport.

We thank the Reviewer for the positive assessment of our revised manuscript, and for the time and effort devoted to reviewing our work throughout the process.

We have now added the following statement and cited corresponding reference in the manuscript: "It can be rigorously shown that q_i can be expressed in terms of integer-valued, scalar, and time-independent atomic oxidation numbers.[77]"

Review of manuscript NCOMMS-25-45033

The manuscript "Disentangling Cation-Polyanion Coupling in Solid Electrolytes: Which Anion Motion Dominates Cation Transport?" by K. Li and co-authors tackles the problem of understanding the correlation between the anion motion and the ionic conductivity in SSE. The problem is timely and very relevant to the community.

However, the work presents a major critical issue that impedes me from suggesting publication.

The Authors compute the conductivity via the Nernst-Einstein (NE) relation (see Eq. (2), p. 21).
However, this relation is an approximation that discards by construction the correlations:

- among the carriers (in this case, the cations)
- between the carriers and the solid matrix of the SSE.

In order to include these effects, one should compute the ionic conductivity via the full Green-Kubo (GK) relation:

$$\sigma_{GK} = \frac{\Omega}{3k_B T}\int_0^{\infty} < \vec{J}(t) \cdot \vec{J}(0) > dt$$

employing the full charge flux

$$\vec{J}(t) = \frac{e}{\Omega}\sum_{i=1}^{N} q_i \vec{v}_i(t)$$

where the sum is extended over all the N atoms of the simulation cell, of volume Ω.
The full GK expression is able to take into account the interactions among the ions. These correlation effects are at the basis of the so-called Haven ratio:

$$H_R = \frac{\sigma_{NE}}{\sigma_{GK}}$$

i.e. the ratio between the NE estimate for the ionic conductivity to its full value, which can indeed substantially deviate from 1. In SSEs it is often just a smaller fraction, since the concerted motion of ions is able to facilitate the motion of charge carriers at an effectively decreased energetic cost. This is also evidenced in energy landscape analysis in concerted migration of charge carriers, such as in He, X., Zhu, Y. & Mo, Y. “Origin of fast ion diffusion in super-ionic conductors.” Nat Commun 8, 15893 (2017). https://doi.org/10.1038/ncomms15893.

Methods that are solely based on atomic diffusivities cannot capture correlations.
See, e.g., A. Marcolongo, N. Marzari, Phys. Rev. Materials 1, 025402, DOI: https://doi.org/10.1103/PhysRevMaterials.1.025402 for more information and examples about this crucial drawback of NE approximation, compared with full GK relation, in SSEs.

Therefore, most of the results the Authors present aim at clarifying the role of interionic correlations, but are based on calculations of the ionic conductivity that neglect these correlations by constructions, which is a severe inconsistency that flaws the reliability of the conclusions drawn.
Notice that freezing or constraining the motion of the anionic groups does not resolve these issues, since interionic correlations among charge carriers may still be present, and dependent on the constraints. Once again, the latter cannot be captured by atomic-diffusion

based estimates, like the NE relation. The use of carriers' atomic diffusivity alone can depend on interionic correlations only in "mean field", but the explicit, dynamical, time dependence of the correlations (which is what defines, e.g., the paddlewheel effects) are not captured.

Second revision of manuscript NCOMMS-25-45033A

June 20, 2026

We thank the Authors for their careful revision of the manuscript. In our view, the revised version has substantially improved with respect to the previous submission, both in terms of clarity and in the presentation of the numerical results. However, before we can suggest publication in *Nature Communications*, the author need to address satisfactorily the following remaining issues.

1 Flaws in the Appendix of the Authors' response

We respectfully disagree with the statement that, in a crystalline solid electrolyte with a non-diffusive framework, the framework self term and the carrier–framework cross term in the Einstein–Helfand expression necessarily vanish in the long-time limit. The conclusion is correct only after transforming to the framework (i.e., solid-matrix) reference frame. It does not follow in the barycentric reference frame, which is the reference frame usually adopted in equilibrium MD simulations.

Let the Einstein–Helfand charge displacement be written, in the barycentric frame, as

$$\Delta\boldsymbol{\mu}^{\mathrm{bar}}(t) = \sum_{i\in C} q_i \Delta\mathbf{r}_i^{\mathrm{bar}}(t) + \sum_{j\in F} q_j \Delta\mathbf{r}_j^{\mathrm{bar}}(t) \equiv \mathbf{A}_C^{\mathrm{bar}}(t) + \mathbf{A}_F^{\mathrm{bar}}(t), \tag{1}$$

where C denotes the mobile carriers and F the framework atoms. The conductivity is then

$$\sigma = \frac{1}{3\Omega k_{\mathrm{B}}T} \lim_{t\to\infty} \frac{1}{2t} \left\langle \left|\mathbf{A}_C^{\mathrm{bar}}(t) + \mathbf{A}_F^{\mathrm{bar}}(t)\right|^2 \right\rangle. \tag{2}$$

The argument in the manuscript assumes that $\mathbf{A}_F^{\mathrm{bar}}(t)$ is bounded because the framework atoms are non-diffusive. This is the step that is not generally valid.

Indeed, non-diffusivity of the framework implies bounded displacements only with respect to the solid matrix, *not necessarily in the barycentric frame.* Writing

$$\Delta\mathbf{r}_j^{\mathrm{bar}}(t) = \Delta\mathbf{R}_F^{\mathrm{bar}}(t) + \Delta\mathbf{u}_j(t), \tag{3}$$

where $\Delta\mathbf{R}_F^{\mathrm{bar}}(t)$ is the displacement of the framework center of mass and $\Delta\mathbf{u}_j(t)$ is the displacement of atom j relative to the framework, only $\Delta\mathbf{u}_j(t)$ is bounded for a non-diffusive framework. Therefore

$$\mathbf{A}_F^{\mathrm{bar}}(t) = \sum_{j\in F} q_j \Delta\mathbf{r}_j^{\mathrm{bar}}(t) = Q_F \Delta\mathbf{R}_F^{\mathrm{bar}}(t) + \sum_{j\in F} q_j \Delta\mathbf{u}_j(t), \tag{4}$$

with

$$Q_F = \sum_{j\in F} q_j. \tag{5}$$

The second term in Eq. (4) is bounded, but the first one is not generally bounded. In a barycentric frame the total center of mass is fixed, so that

$$M_C \Delta\mathbf{R}_C^{\mathrm{bar}}(t) + M_F \Delta\mathbf{R}_F^{\mathrm{bar}}(t) = 0, \tag{6}$$

where M_C and M_F are the total masses of the carrier and framework subsystems, respectively. Hence

$$\Delta\mathbf{R}_F^{\mathrm{bar}}(t) = -\frac{M_C}{M_F} \Delta\mathbf{R}_C^{\mathrm{bar}}(t). \tag{7}$$

Redacted

Thus, if the carrier subsystem has a diffusive collective displacement, *the framework center of mass recoils accordingly*. The framework atoms *remain non-diffusive relative to the matrix*, but the *collective barycentric displacement* of the framework is **not** generally bounded.

Consequently,

$$\left\langle \left| \mathbf{A}_F^{\mathrm{bar}}(t) \right|^2 \right\rangle \tag{8}$$

need not be $O(t^0)$ at long times. In particular, when $Q_F \neq 0$, as is the case when the framework carries the charge compensating that of the mobile carriers, the first term in Eq. (4) may have diffusive scaling. Therefore the Cauchy–Schwarz estimate used in the manuscript does not imply

$$\lim_{t\to\infty} \frac{1}{2t} \left\langle \mathbf{A}_C^{\mathrm{bar}}(t) \cdot \mathbf{A}_F^{\mathrm{bar}}(t) \right\rangle = 0. \tag{9}$$

The cross term can instead give a finite contribution, because both $\mathbf{A}_C^{\mathrm{bar}}(t)$ and the collective recoil part of $\mathbf{A}_F^{\mathrm{bar}}(t)$ may scale as $t^{1/2}$. This is indeed what was observed in Sec. III.E of Ref. [GTGC24], and detailed, for the cross-correlation term between the Li^+ carriers and the thiophosphate matrix, in Fig. S13 of its Supporting Information, reported for convenience here as Fig. 1.

The correct statement is therefore reference-frame dependent. If one transforms to the solid-matrix frame,

$$\mathbf{v}_i^{\mathrm{mat}} = \mathbf{v}_i^{\mathrm{bar}} - \mathbf{w}_F^{\mathrm{bar}}, \qquad \mathbf{w}_F^{\mathrm{bar}} = \frac{\sum_{j\in F} m_j \mathbf{v}_j^{\mathrm{bar}}}{\sum_{j\in F} m_j}, \tag{10}$$

then the framework contribution is the time derivative of a bounded quantity and does not contribute to the conductivity. In that frame one may use the mobile-carrier current

$$\widetilde{\mathbf{J}} = \frac{1}{\Omega} \sum_{i\in C} q_i \mathbf{v}_i^{\mathrm{mat}}. \tag{11}$$

Equivalently, in the barycentric frame, one may use the total charge current

$$\mathbf{J} = \frac{1}{\Omega} \sum_{i\in C\cup F} q_i \mathbf{v}_i^{\mathrm{bar}}. \tag{12}$$

These two formulations are equivalent, up to bounded gauge terms and using charge neutrality. What is not generally equivalent is to use only

$$\mathbf{J}_C^{\mathrm{bar}} = \frac{1}{\Omega} \sum_{i\in C} q_i \mathbf{v}_i^{\mathrm{bar}} \tag{13}$$

in the barycentric frame while discarding the framework and carrier–framework terms.

The non-diffusive nature of the framework does not by itself justify neglecting σ_F and σ_{cross} in the barycentric Einstein–Helfand expression. It only justifies doing so after the carrier displacements or velocities have been expressed relative to the solid framework.

The Authors should explicitly state which reference frame was used in the conductivity calculation and either compute the total barycentric charge current or transform the Li velocities/displacements to the solid-matrix frame before neglecting framework terms.

2 Role of anharmonicity

A second point that remains unclear concerns the interpretation of the constrained metadynamics calculations in terms of anharmonicity. The comparison between the unconstrained case, the rotation-constrained case, and the translation-constrained case shows that allowing translational motion of the BH_4^- units substantially lowers the Li migration free-energy barrier. This is an important observation. However, it does not by itself constitute a direct measure of anharmonicity.

Indeed, a framework coordinate can strongly lower a migration barrier simply by relaxing or by dynamically gating the Li migration pathway, even if that coordinate is described by a purely harmonic potential. Similarly, the curvature of a free-energy minimum is not, by itself, a measure of anharmonicity. A shallow minimum corresponds to a soft harmonic mode if the free-energy profile remains quadratic. Anharmonicity requires evidence for deviations from a quadratic description, such as non-parabolic free-energy profiles, non-Gaussian displacement distributions, higher-order force constants, or strong temperature-dependent phonon renormalization. Broadening/softening of a finite-T velocity spectrum suggest strong coupling, but are not by themselves unique signatures of anharmonicity.

We therefore suggest that the Authors either provide a quantitative measure of anharmonicity supporting this interpretation, or rephrase the conclusion more conservatively. A quantitative proxy may be the *degree of anharmonicity* introduced by Knoop *et al.* in Ref. [KPSC20]: if $\mathbf{F}_I(t)$ is the force sampled along a finite-temperature trajectory for atom I and $\mathbf{F}_I^{(2)}(t)$ is its harmonic approximation, then the global degree of anharmonicity at temperature T is

$$\text{deg. anharm. } (T) = \left[\frac{\sum_{I,\alpha} \left\langle \left(F_{I\alpha}(t) - F_{I\alpha}^{(2)}(t) \right)^2 \right\rangle_T}{\sum_{I,\alpha} \langle F_{I\alpha}^2(t) \rangle_T} \right]^{1/2} \tag{14}$$

where α labels Cartesian components. This quantity directly measures the fraction of the finite-temperature force field that is not described by the harmonic approximation. It can also be resolved on selected atoms, species, or modes, which would be particularly useful here to assess whether the BH_4^- translational degrees of freedom are genuinely anharmonic. The degree of anharmonicity was used on similar superionic conductor Li_3PS_4 in Ref. [TGGC24].

Alternatively, the Authors could quantify anharmonicity by explicitly fitting higher-order force constants, for example using packages such as HIPHIVE [EFE19], which allow the construction of force-constant models beyond second order. The relative importance of third-, fourth-, and higher-order force constants involving BH_4^- translations would provide a direct perturbative measure of anharmonicity.

3 Dataset composition

We also have concerns regarding the dataset composition and the reported accuracy of the machine-learning interatomic potentials (MLIPs). First, the total number of structures included in the training and test sets is not clearly stated. Second, the manuscript does not describe how the structures are distributed among the different systems considered. It is therefore unclear whether all materials are equally represented in the dataset, or whether some systems are over- or under-represented. This point is particularly important for structures containing vacancies, whose representation in the training and test sets should be explicitly reported. In addition, in Supplementary Table 1, the RMSE for the forces on the test set is lower than that on the training set for several systems. The authors should explain this behaviour, since it may indicate that the training and test sets differ in composition.

4 Data availability

Before publication, we strongly recommend that the authors make the complete dataset publicly available through an established open repository, such as Zenodo or Materials Cloud [EO13, TKP+20]. This should include the training, validation, and test structures, together with the corresponding DFT energies, forces, and relevant metadata. Providing these data would improve the reproducibility of the work and allow the community to independently assess the quality and transferability of the reported MLIPs.

References

[EFE19] Fredrik Eriksson, Erik Fransson, and Paul Erhart. The Hiphive Package for the extraction of high-order force constants by machine learning. *Advanced Theory and Simulations*, 2(5):1800184, 2019.

[EO13] European Organization For Nuclear Research and OpenAIRE. Zenodo, 2013.

[GTGC24] Lorenzo Gigli, Davide Tisi, Federico Grasselli, and Michele Ceriotti. Mechanism of charge transport in lithium thiophosphate. *Chemistry of Materials*, 36(3):1482–1496, 2024.

[KPSC20] Florian Knoop, Thomas A. R. Purcell, Matthias Scheffler, and Christian Carbogno. Anharmonicity measure for materials. *Phys. Rev. Mater.*, 4:083809, Aug 2020.

[TGGC24] Davide Tisi, Federico Grasselli, Lorenzo Gigli, and Michele Ceriotti. Thermal conductivity of Li_3PS_4 solid electrolytes with ab initio accuracy. *Phys. Rev. Mater.*, 8:065403, Jun 2024.

[TKP+20] Leopold Talirz, Snehal Kumbhar, Elsa Passaro, Aliaksandr V. Yakutovich, Valeria Granata, Fernando Gargiulo, Marco Borelli, Martin Uhrin, Sebastiaan P. Huber, Spyros Zoupanos, Carl S. Adorf, Casper Welzel Andersen, Ole Schütt, Carlo A. Pignedoli, Daniele Passerone, Joost VandeVondele, Thomas C. Schulthess, Berend Smit, Giovanni Pizzi, and Nicola Marzari. Materials cloud, a platform for open computational science. *Scientific Data*, 7(1):299, 2020.
